# Direct sulfonylation of anilines mediated by visible light†
†Electronic supplementary information (ESI) available. See DOI: 10.1039/c7sc03891g


**DOI:** 10.1039/c7sc03891g

**Published:** 2017-11-16

**Authors:** Tarn C. Johnson, Bryony L. Elbert, Alistair J. M. Farley, Timothy W. Gorman, Christophe Genicot, Bénédicte Lallemand, Patrick Pasau, Jakub Flasz, José L. Castro, Malcolm MacCoss, Darren J. Dixon, Robert S. Paton, Christopher J. Schofield, Martin D. Smith, Michael C. Willis

**Affiliations:** a Department of Chemistry , University of Oxford , Chemical Research Laboratory , Mansfield Road , Oxford , OX1 3TA , UK . Email: darren.dixon@chem.ox.ac.uk ; Email: robert.paton@chem.ox.ac.uk ; Email: christopher.schofield@chem.ox.ac.uk ; Email: martin.smith@chem.ox.ac.uk ; Email: michael.willis@chem.ox.ac.uk; b Global Chemistry , UCB New Medicines , UCB BioPharma sprl , 1420 Braine-L'Alleud , Belgium; c Global Chemistry , UCB , 261 Bath Road, Slough , SL1 3WE , UK; d Bohicket Pharma Consulting LLC , 2556 Seabrook Island Road , Seabrook Island , South Carolina 29455 , USA

## Abstract

Sulfones feature prominently in biologically active molecules and are key functional groups for organic synthesis. We report a mild, photoredox-catalyzed reaction for sulfonylation of aniline derivatives with sulfinate salts, and demonstrate the utility of the method by the late-stage functionalization of drugs. Key features of the method are the straightforward generation of sulfonyl radicals from bench-stable sulfinate salts and the use of simple aniline derivatives as convenient readily available coupling partners.

## Abstract 2

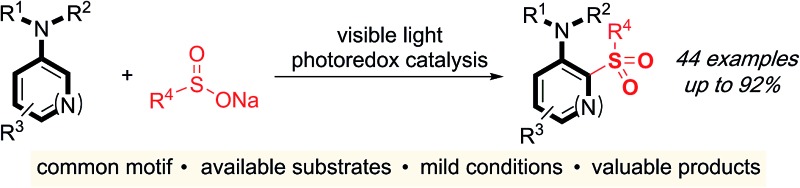
Visible light photocatalysis allows the introduction of the sulfone functional group to anilines under mild reaction conditions, without the need for pre-functionalization.

## Introduction

**Fig. 1:**
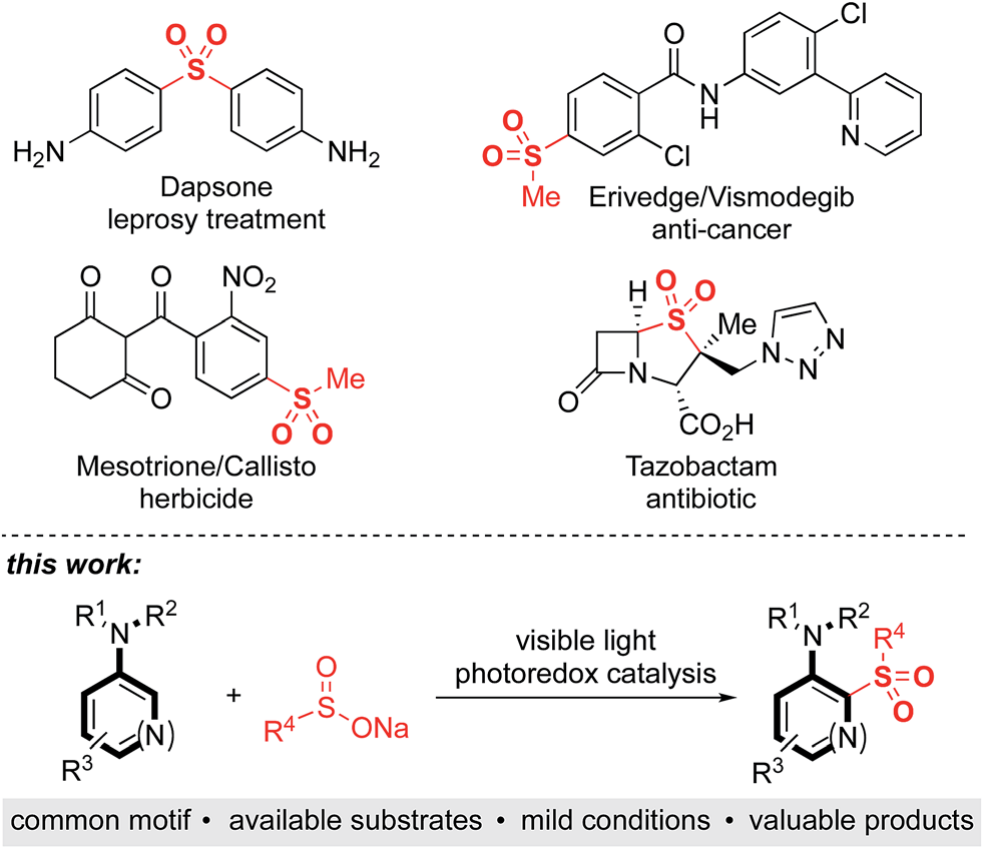
Biologically active sulfones, and the reaction targeted in this work.

Sulfones are an important class of organosulfur compounds that have uses ranging from polymers and solvents to synthetic intermediates.^1^ Sulfones display diverse biological activities, making them of major interest in the pharmaceutical and agrochemical industries; they have been used for the treatment of a variety of conditions, ranging from leprosy to skin cancer, and are included in antibiotic and herbicidal treatments (Fig. 1). The most common method for preparing sulfones is by oxidation of sulfides; this often requires the use of odorous thiols and strongly oxidizing conditions which limits functional group compatibility.^2^ Electrophilic aromatic substitution of (hetero)arenes with sulfonyl chlorides or sulfonic acids are other classical methods for sulfone synthesis. However, these methods normally employ harsh conditions, such as the use of stoichiometric Lewis or Brønsted acids and high temperatures, and the (hetero)arene reaction partner is often required in large excess.^3^ More recent methods for sulfone synthesis include transition-metal-catalyzed coupling reactions between sulfinate salts and aryl halides,^4^ three-component methods utilizing SO_2_ surrogates,^5^ and C–H activation approaches employing a suitable directing group.^6^ There remains, however, an unmet need for new methods that enable access to complex, highly functionalized aryl-sulfones, that employ mild conditions, have wide functional group tolerance, and which are broad in scope. We proposed that a photoredox-catalyzed process, utilizing the redox chemistry of an aromatic substrate in combination with a sulfonyl radical, would enable us to achieve this goal.

Sulfonyl radicals are transient intermediates that undergo a range of reactions, including with alkenes^7^ and alkynes.^8^ However, examples of their reactivity with aromatic rings are scarce:^1^ the groups of Deng and Kuhakarn have independently reported sulfonylation of indoles at the C-2 position using sodium sulfinate salts under oxidative conditions,^9^ Li and coworkers have reported on hypervalent iodine-mediated sulfonylation of anilides with aryl sulfonyl chlorides,^10^ and sulfonyl radicals have been implicated in transition-metal-catalyzed directed C–H sulfonylations.^11^ Here we report on the development of a mild and robust sulfonylation protocol that is applicable to the late-stage functionalization of aniline containing drug-molecules. To the best of our knowledge the reaction described herein represents the first example of a photoredox-catalyzed aryl C–H sulfonylation process (Fig. 1).

## Results and discussion

Anilines were targeted as substrates because they are present in many pharmaceuticals and natural products, and possess an accessible oxidation potential (*N*,*N*-dimethylaniline 0.74 V *vs.* SCE).^12^*N*,*N*-Dimethyl-*p*-toluidine and sodium methanesulfinate were chosen as a test system, with selected optimization experiments presented in Table 1 (see ESI† for details). Evaluation of commercially available photoredox catalysts (entries 1–5) identified [Ir(dF(CF_3_)ppy)_2_(dtbpy)]PF_6_ as the optimal catalyst with potassium persulfate serving as the oxidant to drive the desired reaction under irradiation with visible light (blue LEDs).

**Table 1:** Selected optimization studies for the preparation of sulfone **1**^*a*^

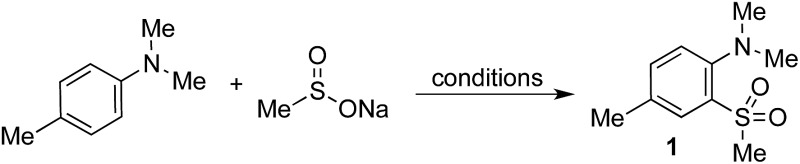			
Entry	Catalyst	Solvent	Yield^*b*^ (%)
1	**2**	10 : 1 MeCN/H_2_O	22
2	**3**	10 : 1 MeCN/H_2_O	12
3	**4**	10 : 1 MeCN/H_2_O	60
4	**5**	10 : 1 MeCN/H_2_O	67
5	**6**	10 : 1 MeCN/H_2_O	60
6^*c*^	**5**	10 : 1 MeCN/H_2_O	60
7	**5**	5 : 1 MeCN/H_2_O	59
8	**5**	MeCN	27
9^*c*^	**5**	10 : 1 MeCN/TFA	58
10	**5**	10 : 1 CH_2_Cl_2_/H_2_O	52
11	**5**	10 : 1 acetone/H_2_O	63
12^*d*^	**5**	10 : 1 MeCN/H_2_O	76
13^*d*^ ^,^^*e*^	**5**	10 : 1 MeCN/H_2_O	85(85)
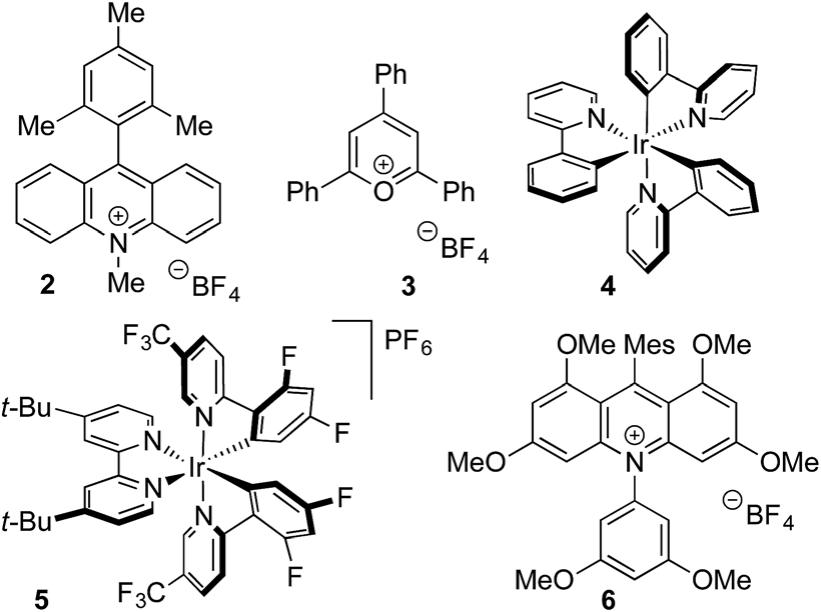			

^*a*^Reaction conditions: catalyst (1 mol%), sodium methanesulfinate (3 equiv.), K_2_S_2_O_8_ (2 equiv.), Bu_4_NHSO_4_ (20 mol%), 0.2 M, blue LEDs, 24 h.

^*b*^ ^1^H NMR yield *vs.* trimethoxybenzene internal standard. Isolated yield in parentheses.

^*c*^No Bu_4_NHSO_4_.

^*d*^5 equiv. sodium methanesulfinate, 3 equiv. K_2_S_2_O_8_, 0.1 M.

^*e*^72 h.

The addition of water or trifluoroacetic acid (TFA) as a co-solvent (entries 4, 7–11) enabled high yields, with 10 : 1 acetonitrile/water providing the optimal solvent combination. The optimized conditions employ a modest excess of the sulfinate salt and oxidant, and a reaction time of 72 h to reach full conversion with the expected sulfone **1** being isolated in 85% yield (entry 13). By way of control reactions, three different protocols to achieve the formation of sulfone **1** from *N*,*N*-dimethyl-*p*-toluidine *via* electrophilic aromatic sulfonylation were performed; all three were unsuccessful, with no trace of sulfone **1** being observed (see the ESI† for details).3a,3b,^13^

With optimised conditions established, the scope of the reaction with respect to the aniline substrates was investigated (Table 2a). We found that both alkyl and aryl substituents on the aromatic ring were well tolerated, affording the corresponding sulfones in high yields (**1**, **7–12**). Electron-rich substrates performed well, with methoxy groups in the *ortho*-, *meta*- and *para*-positions all proceeding in high yields (**13–15**). Pleasingly, a substrate bearing an unprotected hydroxyl group underwent sulfonylation in high yield to give sulfone **16** featuring a *meta* relationship with the dimethylamino group.^14^ Diamine-containing sulfones could be accessed in moderate to good yields from the corresponding phenylene diamine derivatives (**17**, **18**). Halide substituents were also tolerated under the photoredox conditions (**19–21**). More complex anilines preferentially underwent sulfonylation at the least hindered *ortho* position, delivering sulfones **22** and **23**. Pyridines and fused heterocycles were also compatible substrates (**24–27**). A survey of aniline derivatives with differing substituents at the nitrogen atom revealed that although aromatic groups performed very well, different alkyl groups were prone to competing dealkylation processes, resulting in lower yields (**28–34**). It was observed that, in most cases, sulfonylation occurs predictably at the *ortho*- and *para*-positions with respect to the amino substituent.

**Table 2:** Scope of the direct sulfonylation of aniline derivatives^*a*^

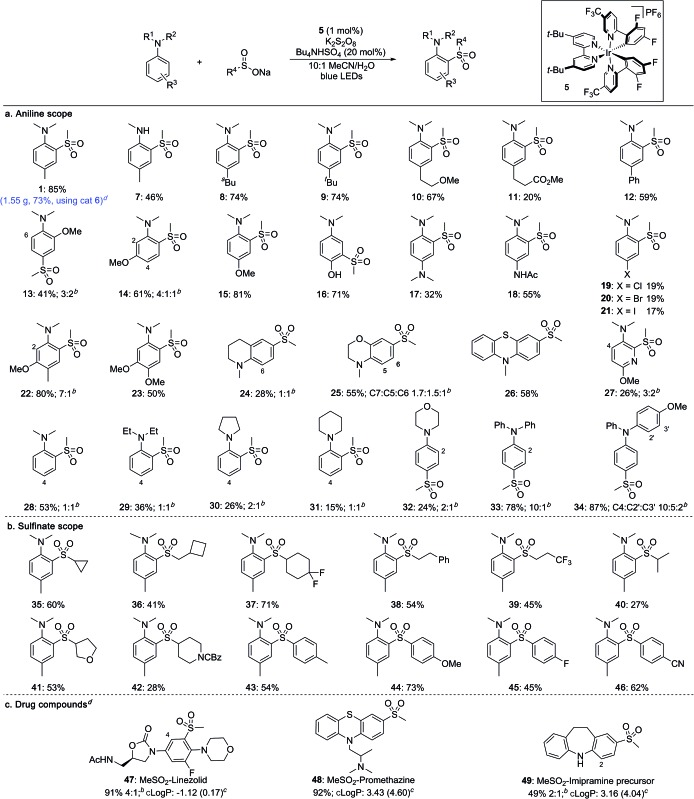

^*a*^Reaction conditions: **5** (1 mol%), sodium methanesulfinate (5 equiv.), K_2_S_2_O_8_ (3 equiv.), Bu_4_NHSO_4_ (20 mol%), 10 : 1 MeCN/H_2_O, 0.1 M, blue LEDs, 72 h.

^*b*^Minor regioisomer labelled with the corresponding carbon number.

^*c*^cLog *P* values of starting materials in parentheses.

^*d*^40 W blue LED, 40–56 h.

Sulfinate salts were also evaluated under the reaction conditions and a broad range of substituents were found to be tolerated (Table 2b). Thus, cycloalkyl (**35–37**), as well as linear (**38**, **39**) and branched alkyl (**40**) sulfinates provided the corresponding sulfones in good to moderate yields. Sulfinates featuring both saturated O- and N-heterocycles were compatible (**41**, **42**). Aryl sulfinates were also suitable substrates, and a range of electron-donating and electron-withdrawing substituents could be included on the aromatic rings (**43–46**).

To investigate the robustness of the reaction we carried out the synthesis of sulfone **1** on a preparative scale (10 mmol). Due to the high cost of iridium catalyst **5** we sought a suitable metal-free replacement. Recently, DiRocco and coworkers^15^ disclosed the acridinium photocatalyst **6** (Table 1) and achieved a comparable performance to **5** in a decarboxylative conjugate addition. Replacing iridium catalyst **5** with acridinium **6** under our optimal conditions provided the expected sulfonylation product in 76% isolated yield. Performing the reaction on a 10 mmol scale provided 1.55 g of sulfone **1** in a 73% yield.

The broad applicability of this process to various anilines and sulfinate salts has potential for introducing diversity at a late stage; to demonstrate this we examined functionalization of drugs (Table 2c). Initial studies were encouraging, with sulfonylation being observed in all cases. Changing the light source from blue LED strips (approximately 6 W, see ESI†) to a 40 W LED lamp was used to achieve complete conversion. The antibiotic linezolid was fully converted after 40 h providing two regioisomeric sulfones in 91% combined yield (**47**). Promethazine, a neuroleptic medication, underwent sulfonylation with high regioselectivity providing **48** in 92% yield. The precursor to the antidepressant imipramine was also sulfonylated, giving products of reaction at the 2- and 4-positions in 16% and 33% yield, respectively (**49**). The incorporation of a methylsulfone (MeSO_2_–) group can substantially lower the lipophilicity of a molecule and provide a potential site for biological interaction as a hydrogen-bond acceptor. The successful late-stage sulfonylation of these complex molecules bearing sensitive and reactive functionalities demonstrates the potential utility of the method for medicinal chemistry.

**Scheme 1:**
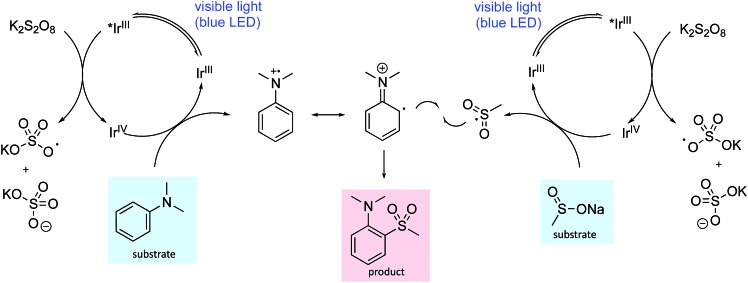
A plausible reaction mechanism for photoredox mediated aryl sulfonylation showing the iridium ion enabled oxidative quenching cycle.

One plausible mechanistic proposal is illustrated in Scheme 1. The oxidation potentials of the aniline (*N*,*N*-dimethylaniline 0.74 V *vs.* SCE)^12^ and the sulfinate salt (sodium methanesulfinate 0.46 V *vs.* SCE)7*a* are both accessible by the iridium catalyst such that both oxidative and reductive quenching cycles can operate simultaneously (Ir(iii)*/Ir(ii) 1.21 V *vs.* SCE, Ir(iv)/Ir(iii) 1.69 V *vs.* SCE).^16^ Oxidation of the aniline to the radical cation and its reaction with the sulfonyl radical, generated by oxidation of the sulfinate salt, provides the sulfone product following proton loss. The addition of neutral and anionic nucleophiles to arene radical cations has been demonstrated,^17^ so nucleophilic attack of the sulfinate anion cannot be ruled out; however, addition of 1-phenylstyrene as a sulfonyl radical trap ablates the reaction making this pathway seem less likely (see ESI† for details). In addition, application of reported sulfonyl-radical generating conditions (I_2_, MeOH) to our test substrate combination, failed to deliver any sulfone product. We also explored the possibility of a thiosulfonate intermediate; however, the use of *S*-methyl methanethiosulfonate in place of sodium methanesulfinate, did not deliver the sulfone product. Unsuccessful aromatic substrates include 1,4-dimethoxybenzene, 1-methylindole and 1-methylimidazole, with no reaction occurring in each case. These observations make an electrophilic aromatic substitution pathway involving a sulfur-based electrophile unlikely. In addition, substrates bearing electron-withdrawing groups were generally unreactive, consistent with a mechanism requiring oxidation of the aniline to the radical cation, as electron-withdrawing groups will raise the oxidation potential. A detailed mechanistic investigation is ongoing.

**Scheme 2:**
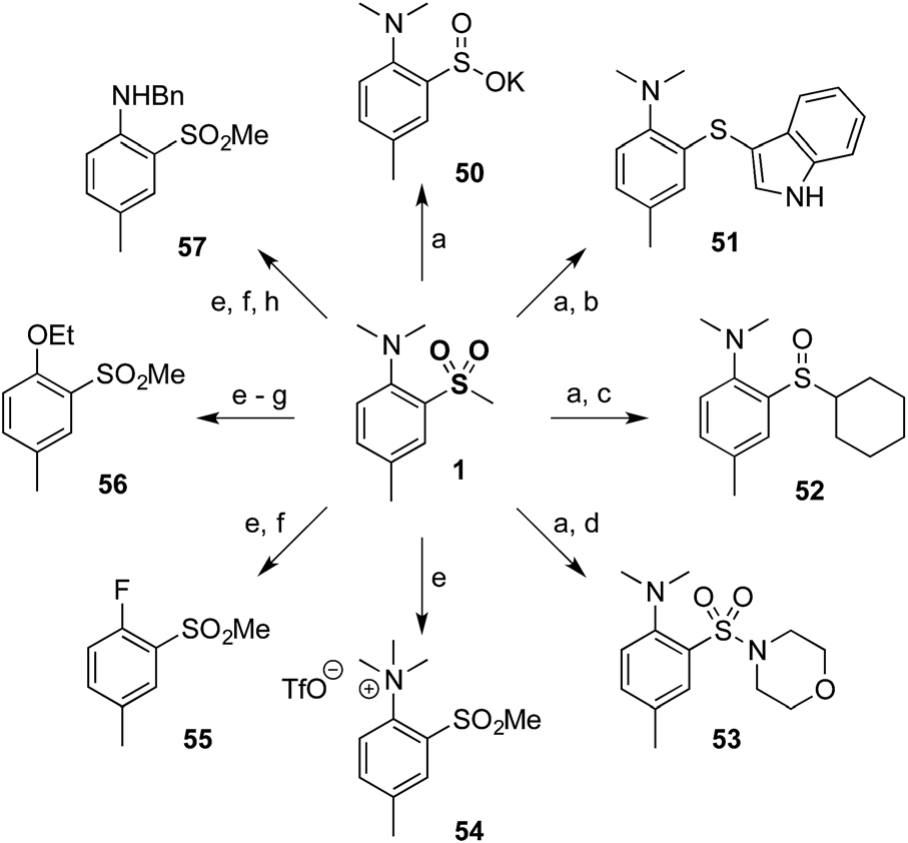
Efficient and flexible derivatization of sulfone **1**. (a) **1**, BnBr, KO^*t*^Bu, Et_2_O, 62%. (b) **50**, indole, I_2_, PPh_3_, EtOH, 78 °C, 83%. (c) **50**, SOCl_2_, MeOH, then CyMgCl, THF, 71%. (d) **50**, *N*-chloromorpholine, ^*i*^PrOH, 70%. (e) **1**, MeOTf, 79%. (f) **54**, TBAF, NMP, 200 °C, 60%. (g) **55**, NaOEt, EtOH, 78 °C, quant. (h) **55**, BnNH_2_, DMSO, 130 °C, 78%.

Finally, to demonstrate the utility of our methodology a diverse range of derivatives of sulfone **1** were prepared (Scheme 2). Thus, minor modification of a literature procedure^18^ led to the generation of sulfinate salt **50** (62%). Further transformations were straightforward, providing access to products at the sulfide (**51**) and sulfoxide (**52**) oxidation levels, in addition to the sulfonamide (**53**). The dimethylamino group could be modified by methylation to provide quaternary ammonium salt **54**, which could be further converted to aryl fluoride **55** and subsequently to ether **56** or protected aniline **57** by nucleophilic aromatic substitution.

## Conclusions

In conclusion, we have developed a new and scalable method for the introduction of the sulfone functional group to anilines under mild conditions, without the need for pre-functionalization of the aromatic ring. The mild reaction conditions and consequent excellent functional group tolerance are exemplified by the late-stage functionalization of important biologically active compounds, suggesting the method will be a valuable tool in discovery chemistry.

## Conflicts of interest

There are no conflicts to declare.

## Supplementary Material

Supplementary information
