# Supplementary material for: Direct sulfonylation of anilines mediated by visible light
Source: Chem Sci. 2017 Nov 16;9(3):629–33. doi: 10.1039/c7sc03891g (PMC5868301; doi:10.1039/c7sc03891g)
Supplement: Supplementary file 1 [file SC-009-C7SC03891G-s001.pdf]

Electronic Supplementary Material (ESI) for Chemical Science.
This journal is © The Royal Society of Chemistry 2017

Supplementary Information

Direct Sulfonylation of Anilines Mediated by Visible Light

Tarn C. Johnson,[a] Bryony L. Elbert,+[a] Alistair J.M. Farley,+[a] Timothy W. Gorman,+[a] Christophe Genicot,[b] Bénédicte Lallemand,[b] Patrick Pasau,[b] Jakub Flasz,[c] José L. Castro,[c] Malcolm MacCoss,[d] Darren J. Dixon,*[a] Robert S. Paton,*[a] Christopher J. Schofield,*[a] Martin D. Smith*[a] and Michael C. Willis*[a]

[a] Dr T.C. Johnson, Dr B. L. Elbert+, Dr. A.J.M. Farley+, Dr. T.W. Gorman+, Prof. D. J. Dixon, Dr. R.S. Paton, Prof. C. J. Schofield, Prof. M. D. Smith, Prof. M. C. Willis
Department of Chemistry, Chemistry Research Laboratory
University of Oxford, 12 Mansfield Road, Oxford OX1 3TA (UK)
E-mail: michael.willis@chem.ox.ac.uk
[+] These authors contributed equally to this work.

[b] Dr C.Genicot, Dr B. Lallemand, Dr P. Pasau,
Global Chemistry, UCB New Medicines, UCB Biopharma sprl
1420 Braine-L'Alleud (Belgium)

[c] Dr J. Flasz, Dr J.L. Castro
Global Chemistry, UCB, 216 Bath Road, Slough, SL1 3WE, U.K.

[d] Dr M. MacCoss
Bohicket Pharma Consulting LLC, 2556 Seabrook Island Road, Seabrook Island, South Carolina 29455, United States

Supplementary Information

**General Experimental**	S3
**Additional Details of Optimization Studies**	S4
**Control Experiments**	S6
**Synthesis and Characterization of Non-Commercial Starting Materials**	S8
**Synthesis and Characterization of Sulfonylation Products**	S11
**10 mmol Scale Reaction**	S29
**Derivatization Reactions**	S29
**NMR Spectra of Novel Compounds**	S33
**References**	S110

General Experimental

Solvents and Reagents. Tetrahydrofuran and diethylether were obtained anhydrous from MBRAUN SPS5 solvent dispenser units having been passed through an activated alumina column under argon. Dimethylsulfoxide and 1-methylpyrrolidinone were purchased as anhydrous solvent in a Sure/Seal™ bottles from Sigma Aldrich and used as received. 2-Propanol was dried over 4 Å molecular sieves. Petroleum ether refers to the fraction which boils in the range 40-60 ºC.

Reactions. All reactions were carried out under argon or nitrogen unless otherwise stated.

Chromatography. Thin-layer chromatography was performed on Merck aluminium-backed DC 60 F254 0.2 mm precoated plates, which were visualised with UV fluorescence and staining with potassium permanganate or vanillin. Flash column chromatography was performed on a Biotage Isolera with Biotage ZIP or ZIP Sphere® Silica cartridges.

Melting Points. Melting points were determined using a Griffin melting point apparatus and are uncorrected.

Infrared Spectroscopy. Infrared spectra were recorder on a Bruker Tensor 27 Fourier transform spectrometer, as a thin film on a diamond ATR module.

NMR Spectroscopy. ^{1}H NMR spectra were recorded at 400 or 500 MHz on a Bruker AVIIIHD 400, Bruker AVII 500, or Bruker AVIIIHD 500 respectively. ^{13}C NMR spectra were recorded at 100 MHz or 125 MHz on a Bruker AVIIIHD 400, Bruker AVIIIHD 500 or a Bruker AVII 500 with ^{13}C cryoprobe, respectively. Chemical shifts (δ_H and δ_C) are expressed in parts per million (ppm), referenced to tetramethylsilane or the residual solvent peak of $CDCl_3$. Coupling constants (*J*) are reported to the nearest 0.1 Hz. Spectra are assigned based on chemical shift, coupling constants, COSY, HSQC, HMBC and NOESY data and comparison with similar compounds. Splitting patterns are described using the following abbreviations: s (singlet), d (doublet), t (triplet), q (quartet), quin. (quintet), sept. (septet), app. (apparent). (Q) indicated a quaternary carbon. Atoms are generally numbered according to IUPAC conventions, unless otherwise indicated. Deviation from IUPAC numbering is employed to facilitate consistent assignment of compounds.

Mass Spectroscopy. Low-resolution mass spectra (*m/z*) were performed on a Micromass LCT Premier Open Access. High-resolution mass spectra were recorded under ESI or EI conditions on a Bruker MicroTOF.

Light Sources. For photoredox experiments a 6 W, 2.5 M strip of Tingkam® 5050 SMD RGB LEDs set to blue was used as shown in Figure S1. For the late-stage functionalization of drug compounds and 10 mmol scale experiment a Kessil 40 W A160WE Tuna Blue LED lamp was used with the reaction vessel placed ~5 cm from the lamp. Reactions remained at ~28 °C and no additional cooling was required.

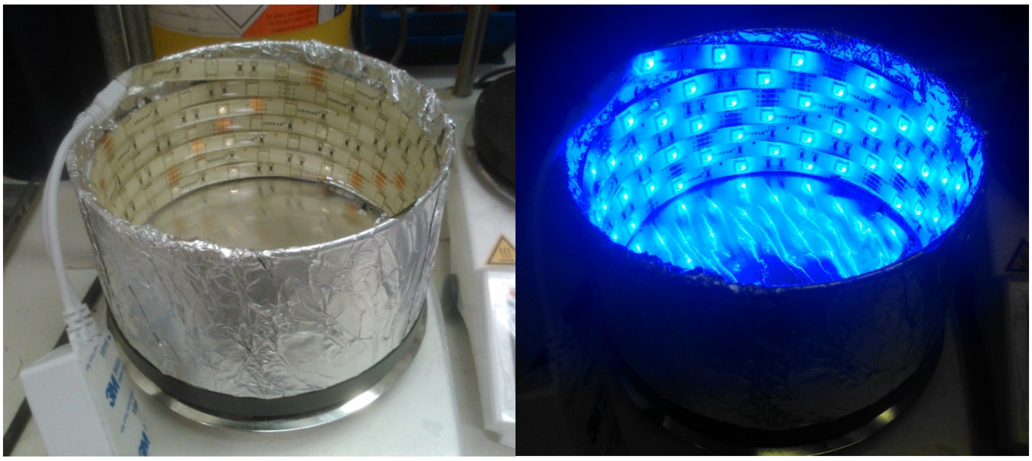

Figure S1. Photoredox apparatus.

Additional Details of Optimization Studies

Table S1. Oxidant screen.

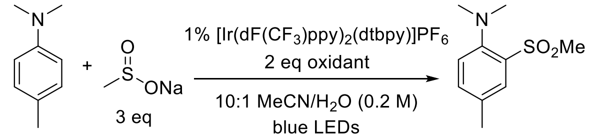

Oxidant	Additive	Time (h)	Yield (%)[a]
H_2O_2	-	16	0[b]
O_2	-	16	0[b]
$K_2S_2O_8$	-	16	60
$K_2S_2O_8$	2 eq K_2CO_3	16	0
$K_2S_2O_8$	-	24	69 (67)[c]
$K_2S_2O_8$	-	24	10[d]
$K_2S_2O_8$	-	24	6[e]
$Na_2S_2O_8$	-	24	62
$(H_4N)_2S_2O_8$	-	24	54
$(Bu_4N)_2S_2O_8$	-	24	26

Reaction conditions: 1 mol% catalyst, 3 eq sodium methanesulfinate, 2 eq oxidant, 0.2 M, blue LEDs, 24 h. [a] ^{1}H NMR yield *vs* trimethoxybenzene internal standard. Isolated yield in brackets. [b] Mono-*N*-demethylation of *N*,*N*-dimethyl-*p*-toluidine observed. [c] Poor solubility of the sodium methanesulfinate and $K_2S_2O_8$ led to inconsistencies in mixing and poor reproducibility. The addition of 20 mol% Bu_4NHSO_4 alleviated this issue. [d] No iridium. [e] No light.

Table S2. Solvent screen.

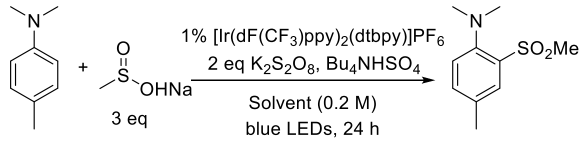

Solvent	Bu_4NHSO_4 eq	Yield (%)[a]
10:1 $MeCN/H_2O$	0.2	67
5:1 $MeCN/H_2O$	0.2	59
Dry MeCN	0.2	27
10:1 CH_2Cl_2/H_2O	0.2	52
10:1 $HFIP/H_2O$	0.2	13
10:1 $Acetone/H_2O$	0.2	63
10:1 MeCN/AcOH	0	26
10:1 MeCN/TFA	0	58

Reaction conditions: 1 mol% catalyst, 3 eq sodium methanesulfinate, 2 eq oxidant, 0.2 M, blue LEDs, 24 h. HFIP = hexafluoroisopropanol, TFA = trifluoroacetic acid. [a] ^{1}H NMR yield *vs* trimethoxybenzene internal standard. Isolated yield in brackets.

Table S3. Catalyst screen.

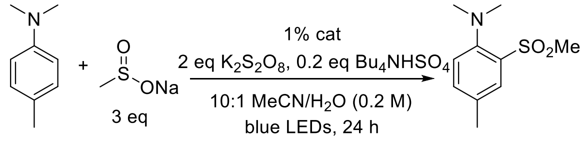

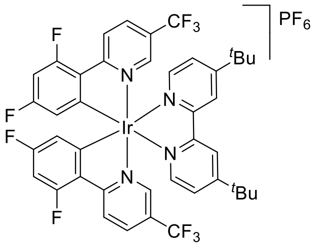

67% NMR yield

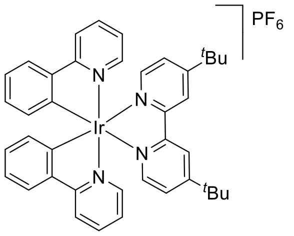

38% NMR yield

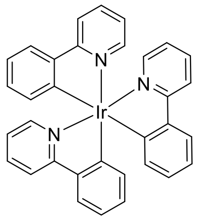

60% NMR yield

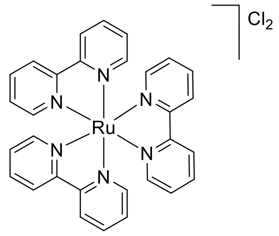

69% NMR yield

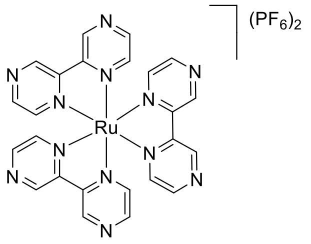

27% NMR yield

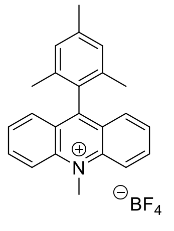

22% NMR yield

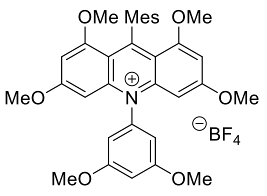

60% NMR yield

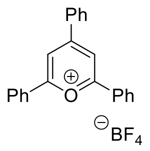

15% NMR yield

Reaction conditions: 1 mol% catalyst, 3 eq sodium methanesulfinate, 2 eq oxidant, 0.2 eq Bu_4NHSO_4, 0.2 M, blue LEDs, 24 h. Although RuBpy gave a promising result after 24 h, higher yields could not be obtained over longer reaction times. ^{1}H NMR yield *vs* trimethoxybenzene internal standard.

Table S4. Other parameters.

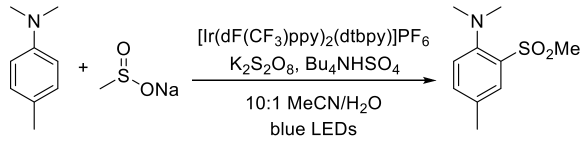

Ir (%)	Conc (M)	Sulfinate eq	$K_2S_2O_8$ eq	Bu_4NHSO_4 eq	Time (h)	Yield (%)[a]
1	0.2	3	2	0.2	24	67
1	0.5	3	2	0.2	24	0
1	0.2	1	2	0.2	24	29
1	0.2	3	1	0.2	24	15
2	0.2	3	2	0.2	24	72
1	0.1	5	3	0.2	72	85

[a] ^{1}H NMR yield *vs* trimethoxybenzene internal standard.

Control Experiments

Scheme S1. Attempted electrophilic aromatic sulfonylation.

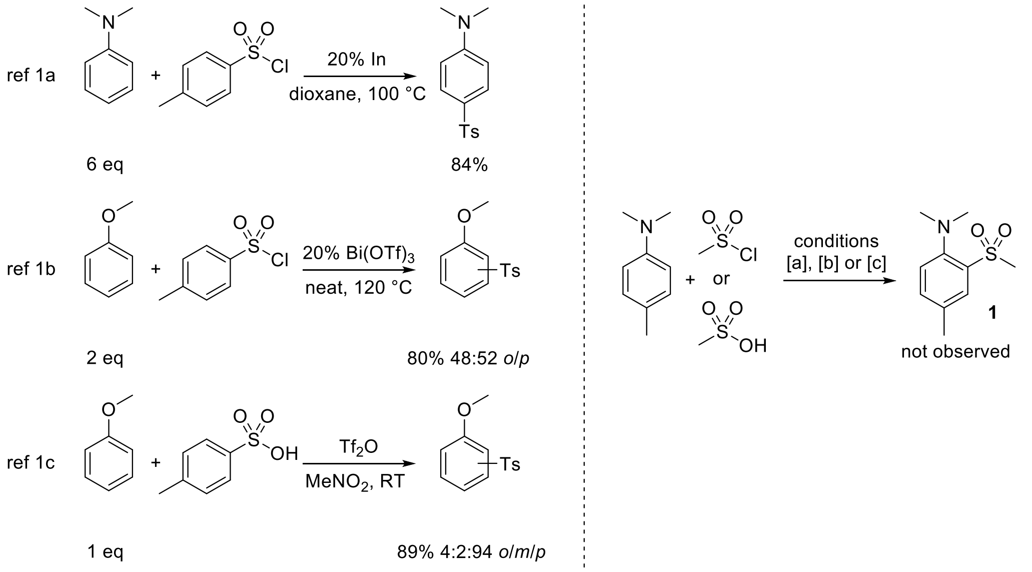

[a] MsCl, 20% In, dioxane, 100 °C. [b] MsCl, 5% Bi(OTf)$_3$, neat, 120 °C. [c] MsOH, Tf$_2$O, MeNO$_2$, room temperature.

After conducting a survey of literature reports for electrophilic aromatic sulfonylation reactions, three exemplar conditions[1] from the current state of the art were selected and applied to the methylsulfonylation of *N*,*N*-dimethyl-*p*-toluidine (Scheme S1). No trace of **1** was observed in all cases.

Scheme S2. Testing a thiosulfonate.

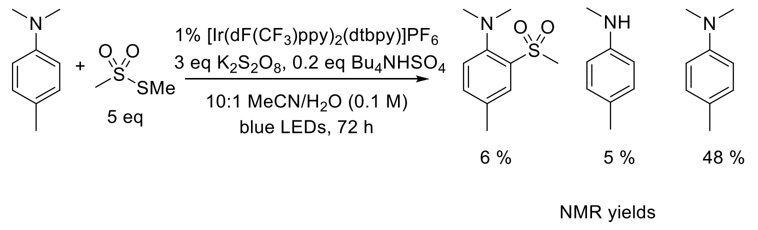

In some reactions thiosulfonates were isolated. To probe their possible role in the reaction *S*-methyl methanethiosulfonate was used in place of sodium methanesulfinate in the methanesulfonylation of *N*,*N*-dimethyl-*p*-toluidine following general procedure A (Scheme S2). The sulfonylation product was observed in only 6% NMR yield suggesting that thiosulfonates do not play a significant role.

Scheme S3. Radical trapping experiments.

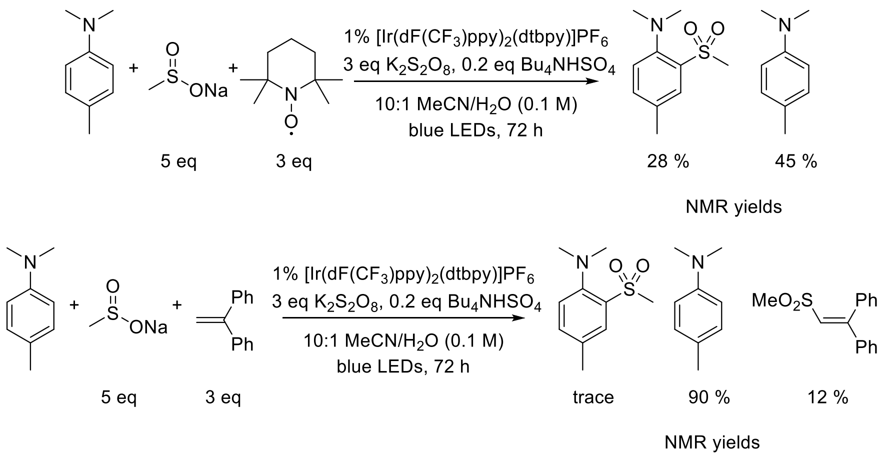

A sulfonylation according to general procedure A was performed in the presence of 3 equivalents of TEMPO (Scheme S3) resulting in a much lower yield than would be otherwise expected (28% vs 85% without TEMPO) indicating that a mechanism involving radical intermediates is likely in operation. The same experiment instead utilizing 1,1-diphenylethene as a trapping agent resulted in the formation of aryl sulfone in only trace quantities. The known compound (2-methanesulfonyl-1-phenylethenyl)benzene was observed in 12% NMR yield, indicating that sulfonyl radicals are likely formed under the reaction conditions.

Scheme S4. Unsuccessful substrates.

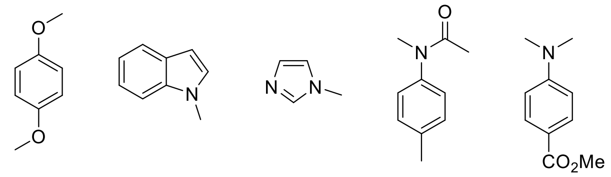

The sulfonylation of other electron-rich aromatics including 1,4-dimethoxybenzene, 1-methylindole and 1-methylimidazole was attempted with no reaction occurring in each case. This makes an electrophilic aromatic substitution pathway involving a sulfur-based electrophile seem unlikely. Substrates bearing electron-withdrawing groups were generally unreactive, this is consistent with a

mechanism requiring oxidation of the aniline to the radical cation as electron-withdrawing groups will raise the oxidation potential.

Synthesis and Characterisation of Non-Commerical Starting Materials

3-Methoxy-*N*,*N*,4-trimethylaniline

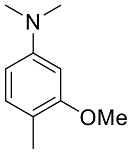

Sodium cyanoborohydride (377 mg, 6.00 mmol) was added to a solution of 3-methoxy-4-methylaniline (274 mg, 2.00 mmol), paraformaldehyde (300 mg, 10.0 mmol) and glacial acetic acid (0.12 mL, 2.00 mmol) in methanol (4 mL, 0.5 M) at 0 °C. The mixture was allowed to warm to room temperature and stir overnight. A saturated $NaHCO_3$ solution (10 mL) was added and the mixture was extracted with ethyl acetate (3 x 10 mL). The combined organic extracts were dried ($MgSO_4$), filtered and the volatiles were removed under reduced pressure. Column chromatography on silica (0-40% ethyl acetate in petroleum ether), afforded the expected product as a colourless oil (260 mg, 1.57 mmol, 79%). ***R*f** 0.25 (10% ethyl acetate in petroleum ether); **^{1}H NMR (400 MHz, $CDCl_3$)** δ 6.98 (1H, d, *J* 7.8, Ar*H*), 6.30-6.25 (2H, m, Ar*H*), 3.82 (3H, s, OCH_3), 2.91 (6H, s, N(CH_3)$_3$), 2.12 (3H, s, ArCH_3); **^{13}C NMR (100 MHz, $CDCl_3$)** δ 158.3 (Q), 150.5 (Q), 130.7, 115.0 (Q), 104.8, 96.6, 55.1, 41.1, 15.2; **IR ν_{max}/cm^{-1} (neat)** 2980, 1618, 1517, 1238, 1123, 812; **HRMS (ESI)** found *m/z* 166.1226 $[M+H]^+$, $C_{10}H_{16}NO$ requires *m/z* 166.1226.

4-(Butan-2-yl)-*N*,*N*-dimethylaniline

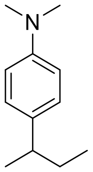

Sodium cyanoborohydride (377 mg, 6.00 mmol) was added to a solution of 4-(butan-2-yl)aniline (299 mg, 2.00 mmol), paraformaldehyde (300 mg, 10.0 mmol) and glacial acetic acid (0.12 mL, 2.00 mmol) in methanol (4 mL, 0.5 M) at 0 °C. The mixture was allowed to warm to room temperature and stir overnight. A saturated $NaHCO_3$ solution (10 mL) was added and the mixture was extracted with ethyl acetate (3 x 10 mL). The combined organic extracts were dried ($MgSO_4$), filtered and the volatiles were removed under reduced pressure. Column chromatography on silica (0-20% ethyl acetate in petroleum ether), afforded the expected product as a colourless oil (239 mg, 1.35 mmol, 67%). ***R*f** 0.45 (10% ethyl acetate in petroleum ether); **^{1}H NMR (400 MHz, $CDCl_3$)** δ 7.09-7.03 (2H, m, Ar*H*), 6.74-6.68 (2H, m, Ar*H*), 2.91 (6H, s, N(CH_3)$_2$), 2.55-2.45 (1H, m, ArC*H*CH$_3$), 1.60-1.51 (2H, m, CH_2), 1.20 (3H, d, *J* 6.9, ArCHCH_3), 0.82 (3H, t, *J*, 7.4, CH$_2$CH_3); **^{13}C NMR (100 MHz, $CDCl_3$)** δ 148.9 (Q), 135.9 (Q), 127.5, 112.8, 40.9, 40.6, 31.3 (CH_2), 22.0, 12.3; **IR ν_{max}/cm^{-1} (neat)** 2959, 1683, 1614, 1519, 1342, 815; **HRMS (ESI)** found *m/z* 178.1590 $[M+H]^+$, $C_{12}H_{20}N$ requires *m/z* 178.1590.

6-Methoxy-*N*,*N*-dimethylpyridin-3-amine

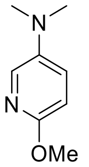

Sodium cyanoborohydride (377 mg, 6.00 mmol) was added to a solution of 6-methoxypyridin-3-amine (248 mg, 2.00 mmol), paraformaldehyde (300 mg, 10.0 mmol) and glacial acetic acid (0.12 mL, 2.00 mmol) in methanol (4 mL, 0.5 M) at 0 °C. The mixture was allowed to warm to room temperature and stir overnight. A saturated $NaHCO_3$ solution (10 mL) was added and the mixture was extracted with ethyl acetate (3 x 10 mL). The combined organic extracts were dried ($MgSO_4$), filtered and the volatiles were removed under reduced pressure. Column chromatography on silica (20-100% ethyl acetate in petroleum ether), afforded an oil which was dissolved in ethyl acetate (10 mL), washed with 2 M NaOH solution (10 mL), dried ($MgSO_4$), filtered and the volatiles were removed under reduced pressure, to yield the expected product as a red oil (218 mg, 1.43 mmol, 72%). $\boldsymbol{R_f}$ 0.40 (40% ethyl acetate in petroleum ether); **^{1}H NMR (400 MHz, $CDCl_3$)** δ 7.68-7.66 (1H, d, *J* 3.1, Ar*H*), 7.17 (1H, dd, *J* 9.0, 3.1, Ar*H*), 6.67 (1H, d, *J* 9.0, Ar*H*), 3.88 (3H, s, OC*H*$_3$), 2.86 (6H, s, N(C*H*$_3$)$_3$); **^{13}C NMR (100 MHz, $CDCl_3$)** δ 157.1 (Q), 142.3 (Q), 131.1, 126.2, 110.3, 53.3, 41.5; **IR ν_{max}/cm^{-1} (neat)** 2945, 1496, 1444, 1283, 1253, 1057, 820, 704; **HRMS (ESI)** found *m/z* 153.1021 $[M+H]^+$, $C_8H_{13}N_2O$ requires *m/z* 153.1022.

2-Cyclobutylmethanesulfanylpyridine

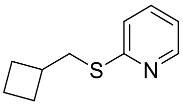

2-Mercaptopyridine (278 mg, 2.50 mmol) in THF (2 mL) was added to a suspension of 60% NaH in mineral oil (300 mg, 3.00 mmol) in dry tetrahydrofuran (3 mL) at 0 °C. After stirring at this temperature for 30 mins bromomethylcyclobutane (0.14 mL, 3.00 mmol) was added. The mixture was allowed to warm to room temperature and stir overnight. A saturated $NaHCO_3$ solution (10 mL) was added and the mixture was extracted with ethyl acetate (3 x 10 mL). The combined organic extracts were dried ($MgSO_4$), filtered and the volatiles were removed under reduced pressure. Column chromatography on silica (0-20% ethyl acetate in petroleum ether), afforded the expected product as a pale yellow oil (260 mg, 1.45 mmol, 58%). $\boldsymbol{R_f}$ 0.34 (20% ethyl acetate in petroleum ether); **^{1}H NMR (400 MHz, $CDCl_3$)** δ 8.48-8.38 (1H, m, Ar*H*), 7.49-7.40 (1H, m, Ar*H*), 7.19-7.12 (1H, m, Ar*H*), 6.98-6.92 (1H, m, Ar*H*), 3.25 (2H, d, *J* 7.6, SC*H*$_2$), 2.70-2.57 (1H, m, C*H*), 2.17-2.08 (2H, m, C*H*$_2$), 1.91-1.71 (4H, m, C*H*$_2$); **^{13}C NMR (100 MHz, $CDCl_3$)** δ 159.5 (Q), 149.3, 135.7, 122.2, 119.1, 36.4, 34.9, 27.8, 18.0; **IR ν_{max}/cm^{-1} (neat)** 2971, 1578, 1556, 1453, 1412, 1123, 754, 726; **HRMS (ESI)** found *m/z* 180.0841 $[M+H]^+$, $C_{10}H_{14}NS$ requires *m/z* 180.0842.

2-Cyclobutylmethanesulfonylpyridine

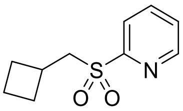

Prepared according to a modified literature procedure.[2] Sodium periodate (1.65 g, 7.73 mmol) was added portionwise to a solution of 2-cyclobutylmethanesulfanylpyridine (231 mg, 1.29 mmol) and

$RuCl_3.xH_2O$ (1.3 mg, 6.50 µmol, 0.5 mol%) in 1:1 ethyl acetate/water (6.5 mL, 0.2 M). The mixture was allowed to stir at room temperature overnight. Water (10 mL) was added and the mixture was extracted with ethyl acetate (3 x 10 mL). The combined organic extracts were dried ($MgSO_4$), filtered and the volatiles were removed under reduced pressure. The residue was passed through a pad of silica (30% ethyl acetate in petroleum ether), affording the expected product as a colourless oil (249 mg, 1.18 mmol, 91%). **^{1}H NMR (400 MHz, CDCl$_3$)** δ 8.79-8.73 (1H, m, Ar*H*), 8.11-8.15 (1H, m, Ar*H*), 8.00-7.92 (1H, m, Ar*H*), 7.58-7.52 (1H, m, Ar*H*), 3.50 (2H, d, *J* 7.3, SC*H*$_2$), 2.85-2.71 (1H, m, C*H*), 2.09-1.99 (2H, m, C*H*$_2$), 1.96-1.70 (4H, m, C*H*$_2$); **^{13}C NMR (100 MHz, CDCl$_3$)** δ 157.6 (Q), 150.2, 138.1, 127.3, 122.1, 57.7, 29.4, 28.3, 19.2; **IR ν_{max}/cm^{-1} (neat)** 2972, 1578, 1427, 1306, 1157, 1110, 767; **HRMS (ESI)** found *m/z* 212.0740 $[M+H]^+$, $C_{10}H_{14}NO_2S$ requires *m/z* 212.0740.

Sodium cyclobutylmethanesulfinate

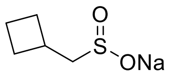

Sodium ethoxide (80 mg, 1.18 mmol) was added to a solution of 2-cyclobutylmethanesulfonylpyridine (227 mg, 1.07 mmol) in ethanol (2 mL, 0.5 M) and heated at 80 °C for 24 h. After cooling to room temperature the volatiles were removed under reduced pressure. A saturated $NaHCO_3$ solution (2 mL) was added the mixture was washed with ethyl acetate (2 x 2 mL). The aqueous extract was dried *in vacuo*, after which the solid was repeatedly washed with ethanol. The combined ethanol washes were evaporated under reduced pressure to yield the titled sulfinate as a yellow, amorphous solid (146 mg, 0.93 mmol, 87%). **^{1}H NMR (400 MHz, CD$_3$OD)** δ 2.63-2.54 (1H, m, C*H*), 2.30 (2H, d, *J* 7.6, SC*H*$_2$), 2.09-2.01 (2H, m, C*H*$_2$), 1.87-1.69 (4H, m, C*H*$_2$); **^{13}C NMR (100 MHz, CD$_3$OD)** δ 70.5, 32.0, 29.7, 20.2; **IR ν_{max}/cm^{-1} (neat)** 2971, 1574, 1393, 1023, 957.

Sodium cyclopropanesulfinate

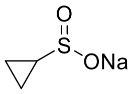

Prepared according to a modified literature procedure.[3] Cyclopropanesulfonyl chloride (0.51 mL, 5.00 mmol) was added to a solution of sodium sulfite (1.26 g, 10.0 mmol) and sodium bicarbonate (840 mg, 10.0 mmol) in water (5 mL, 1 M) and heated at 80 °C for 3 h. After cooling to room temperature the volatiles were removed *in vacuo*. The resultant solid was repeatedly washed with ethanol. The combined ethanol washes were evaporated under reduced pressure to yield the titled sulfinate as a colourless, amorphous solid (472 mg, 3.68 mmol, 74%). **^{1}H NMR (400 MHz, CD$_3$OD)** δ 1.82-1.73 (1H, m, C*H*), 0.68-0.62 (2H, m, C*H*$_2$), 0.55-0.49 (2H, m, C*H*$_2$); **^{13}C NMR (100 MHz, CD$_3$OD)** δ 37.1, 1.1; **IR ν_{max}/cm^{-1} (neat)** 2980, 1382, 1016, 967, 880.

Sodium 4-cyanobenzene-1-sulfinate

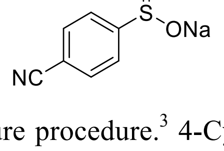

Prepared according to a modified literature procedure.[3] 4-Cyanobenzene-1-sulfonyl chloride (1.00 g, 4.96 mmol) was added to a solution of sodium sulfite (1.26 g, 10.0 mmol) and sodium bicarbonate

(840 mg, 10.0 mmol) in water (5 mL, 1 M) and heated at 80 °C for 3 h. After cooling to room temperature the volatiles were removed *in vacuo*. The resultant solid was repeatedly washed with ethanol. The combined ethanol washes were evaporated under reduced pressure to yield the titled sulfinate as a colourless, amorphous solid (802 mg, 4.24 mmol, 86%). **^{1}H NMR (400 MHz, CD_3OD)** δ 7.70 (4H, s, Ar*H*); **^{13}C NMR (100 MHz, CD_3OD)** δ 162.3 (Q), 133.5, 126.3, 119.5 (Q), 113.9 (Q); **IR ν_{max}/cm^{-1} (neat)** 2980, 2234, 1382, 1251, 1152, 1074, 966.

Synthesis and Characterisation of Sulfonylation Products

In the following general procedures the solvent mixture was sparged with nitrogen for 10 mins prior to use. The reactions can be carried out in air but some *N*-dealkylation of the aniline substrate may be observed.

General procedure A: Table 2, aniline scope.

To a round-bottomed flask charged with [Ir(dF(CF_3)ppy)$_2$(dtbpy)]PF_6 (5.6 mg, 5.00 μmol, 1 mol %), potassium persulfate (405 mg, 1.50 mmol, 3 equiv), tetrabutylammonium hydrogensulfate (34 mg, 0.10 mmol, 0.20 equiv) and sodium methanesulfinate (255 mg, 2.50 mmol, 5 equiv) was added a solution of aniline derivative (0.50 mmol) in 10:1 MeCN/H_2O (5 mL, 0.1 M) under an inert atmosphere. The mixture was stirred for 72 h under irradiation with blue LEDs (see Figure S1). The mixture was neutralised with saturated sodium bicarbonate (5 mL) followed by extraction with EtOAc (3 x 5 mL). The combined organic extracts were dried ($MgSO_4$), filtered and the volatiles were removed under reduced pressure.

General procedure B: Table 3, sulfinate scope.

To a round-bottomed flask charged with [Ir(dF(CF_3)ppy)$_2$(dtbpy)]PF_6 (2.2 mg, 2.00 μmol, 1 mol %), potassium persulfate (162 mg, 0.60 mmol, 3 equiv), tetrabutylammonium hydrogensulfate (14 mg, 0.04 mmol, 0.20 equiv) and sulfinate salt (1.00 mmol, 5 equiv) was added a solution of *N*,*N*-4-trimethylaniline (27 mg, 0.20 mmol) in 10:1 MeCN/H_2O (2 mL, 0.1 M) under an inert atmosphere. The mixture was stirred for 72 h under irradiation with blue LEDs (see Figure S1). The mixture was neutralised with saturated sodium bicarbonate (5 mL) followed by extraction with EtOAc (3 x 5 mL). The combined organic extracts were dried ($MgSO_4$), filtered and the volatiles were removed under reduced pressure.

General procedure C: Scheme 1, late-stage functionalization.

To a round-bottomed flask charged with [Ir(dF(CF_3)ppy)$_2$(dtbpy)]PF_6 (2.2 mg, 2.00 μmol, 1 mol %), potassium persulfate (162 mg, 0.60 mmol, 3 equiv), tetrabutylammonium hydrogensulfate (14 mg, 0.04 mmol, 0.20 equiv) and sodium methanesulfinate (102 mg, 1.00 mmol, 5 equiv) was added a solution of the drug compound (0.20 mmol) in 10:1 MeCN/H_2O (2 mL, 0.1 M) under an inert atmosphere. The mixture was stirred for 40 h under irradiation with a 40 W blue LED. The mixture was neutralised with saturated sodium bicarbonate (5 mL) followed by extraction with EtOAc (3 x 5 mL). The combined organic extracts were dried ($MgSO_4$), filtered and the volatiles were removed under reduced pressure.

2-Methanesulfonyl-*N*,*N*-4-trimethylaniline (1)

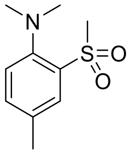

Prepared according to general procedure A. Column chromatography on silica (0-60% ethyl acetate in petroleum ether) afforded the titled sulfone as an off-white, crystalline solid (91 mg, 0.43 mmol, 85%); ***R***$\boldsymbol{R_f}$ 0.20 (20% ethyl acetate in petroleum ether); **mp** 53-54 °C (CH_2Cl_2); **^{1}H NMR (400 MHz, $CDCl_3$)** δ 7.86 (1H, d, *J* 1.8, Ar*H*), 7.41 (1H, dd, *J* 8.1, 1.8, Ar*H*), 7.34 (1H, d, *J* 8.1, Ar*H*), 3.32 (3H, s, SO_2CH_3), 2.76 (6H, s, $N(CH_3)_2$), 2.38 (3H, s, ArCH_3); **^{13}C NMR (100 MHz, $CDCl_3$)** δ 151.3 (Q), 136.2 (Q), 135.2 (Q), 135.1, 129.4, 123.4, 46.1, 42.5, 20.7; **IR ν_{max}/cm^{-1} (neat)** 2980, 1521, 1285, 1133, 1059, 956, 838, 767; **HRMS (ESI)** found *m/z* 214.0896 $[M+H]^+$, $C_{10}H_{16}NO_2S$ requires *m/z* 214.0896.

2-Methanesulfonyl-*N*,4-dimethylaniline (7)

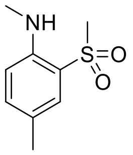

Prepared according to general procedure A. Column chromatography on silica (5-60% ethyl acetate in petroleum ether) afforded the titled sulfone as a pale orange, crystalline solid (46 mg, 0.23 mmol, 46%); $\boldsymbol{R_f}$ 0.24 (20% ethyl acetate in petroleum ether); **mp** 69-70 °C (CH_2Cl_2); **^{1}H NMR (400 MHz, $CDCl_3$)** δ 7.57 (1H, d, *J* 1.6, Ar*H*), 7.29 (1H, dd, *J* 8.4, 1.6, Ar*H*), 6.68 (1H, d, *J* 8.1, Ar*H*), 5.58 (1H, app. broad s, N*H*), 3.03 (3H, s, SO_2CH_3), 2.87 (3H, d, *J* 5.0, NCH_3), 2.27 (3H, s, ArCH_3); **^{13}C NMR (100 MHz, $CDCl_3$)** δ 145.7 (Q), 136.3, 129.5, 125.5 (Q), 121.1 (Q), 111.9, 42.1, 30.1, 20.0; **IR ν_{max}/cm^{-1} (neat)** 3402, 2923, 1616, 1520, 1314, 1284, 1132, 955; **HRMS (ESI)** found *m/z* 200.0749 $[M+H]^+$, $C_9H_{14}NO_2S$ requires *m/z* 200.0745.

4-(Butan-2-yl)-2-methanesulfonyl-*N*,*N*-dimethylaniline (8)

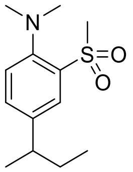

Prepared according to general procedure A. Column chromatography on silica (10-60% ethyl acetate in petroleum ether) afforded the titled sulfone as a pale orange, crystalline solid (95 mg, 0.37 mmol, 74%); $\boldsymbol{R_f}$ 0.32 (20% ethyl acetate in petroleum ether); **mp** 61-62 °C (CH_2Cl_2); **^{1}H NMR (400 MHz, $CDCl_3$)** δ 7.86 (1H, d, *J* 2.1, Ar*H*), 7.42 (1H, dd, *J* 8.2, 2.1, Ar*H*), 7.37 (1H, d, *J* 8.2, Ar*H*), 3.34 (3H, s, SO_2CH_3), 2.77 (6H, s, $N(CH_3)_2$), 2.66 (1H, q, *J* 7.0, ArC*H*), 1.65-1.55 (2H, m, CH_2), 1.24 (3H, d, *J* 7.0, CHCH_3), 0.82 (3H, t, *J* 7.4, CH_2CH_3); **^{13}C NMR (100 MHz, $CDCl_3$)** δ 151.5 (Q), 145.0 (Q), 136.3 (Q), 133.1, 127.5, 123.4, 46.1, 42.5, 41.1, 30.8 (CH_2), 21.5, 12.1; **IR ν_{max}/cm^{-1} (neat)** 2961, 1489, 1452, 1291, 1134, 937, 767; **HRMS (ESI)** found *m/z* 256.1365 $[M+H]^+$, $C_{13}H_{22}NO_2S$ requires *m/z* 256.1366.

4-*tert*-Butyl-2-methanesulfonyl-*N*,*N*-dimethylaniline (9)

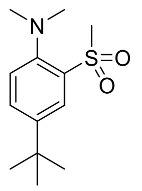

Prepared according to general procedure A. Column chromatography on silica (0-10% ethyl acetate in petroleum ether) afforded the titled sulfone as a pale orange oil (95 mg, 0.37 mmol, 74%); ***R*f** 0.35 (20% ethyl acetate in petroleum ether); **^{1}H NMR (400 MHz, $CDCl_3$)** δ 8.04 (1H, d, *J* 2.4, Ar*H*), 7.60 (1H, dd, *J* 8.4, 2.4, Ar*H*), 7.35 (1H, d, *J* 8.4, Ar*H*), 3.32 (3H, s, SO_2CH_3), 2.76 (6H, s, $N(CH_3)_2$), 1.32 (9H, s, $C(CH_3)_3$); **^{13}C NMR (100 MHz, $CDCl_3$)** δ 151.2 (Q), 148.5 (Q), 136.1 (Q), 131.5, 126.0, 123.2, 46.2, 42.6, 34.7 (Q), 31.3; **IR ν_{max}/cm^{-1} (neat)** 2960, 2861, 1492, 1457, 1298, 1139; **HRMS (ESI)** found *m/z* 256.1360 $[M+H]^+$, $C_{13}H_{22}NO_2S$ requires *m/z* 256.1366.

2-Methanesulfonyl-4-(2-methoxyethyl)-*N*,*N*-dimethylaniline (10)

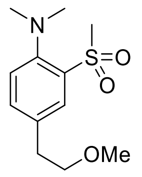

Prepared according to general procedure A. Column chromatography on silica (0-30% ethyl acetate in petroleum ether) afforded the titled sulfone as a pale pink oil (86 mg, 0.33 mmol, 67%); ***R*f** 0.21 (30% ethyl acetate in petroleum ether); **^{1}H NMR (400 MHz, $CDCl_3$)** δ 7.89 (1H, d, *J* 2.3, Ar*H*), 7.46 (1H, dd, *J* 8.3, 2.3, Ar*H*), 7.35 (1H, d, *J* 8.3, Ar*H*), 3.60 (2H, t, *J* 6.7, CH_2OCH_3), 3.34 (3H, s, SO_2CH_3), 3.31 (3H, s, CH_2OCH_3), 2.90 (2H, t, *J* 6.7, $ArCH_2$), 2.75 (6H, s, $N(CH_3)_2$); **^{13}C NMR (100 MHz, $CDCl_3$)** δ 152.1 (Q), 136.6 (Q), 136.5 (Q), 135.2, 129.4, 123.6, 72.8, 58.7, 46.3, 42.6, 35.4; **IR ν_{max}/cm^{-1} (neat)** 2934, 2830, 1491, 1456, 1270, 1140, 1112; **HRMS (EI)** found *m/z* 257.1086 $[M]^+$, $C_{12}H_{19}NO_3S$ requires *m/z* 257.1085.

Methyl 3-[4-(dimethylamino)-3-methanesulfonylphenyl]propanoate (11)

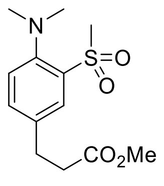

Prepared according to general procedure A. Column chromatography on silica (5-100% ethyl acetate in petroleum ether) afforded the titled sulfone as a white solid (29 mg, 0.10 mmol, 20%); ***R*f** 0.23 (40% ethyl acetate in petroleum ether); **mp** 81-83 °C (CH_2Cl_2); **^{1}H NMR (500 MHz, $CDCl_3$)** δ 7.87 (1H, d, *J* 2.1, Ar*H*), 7.44 (1H, dd, *J* 8.1, 2.1, Ar*H*), 7.34 (1H, d, *J* 8.1, Ar*H*), 3.67 (3H, s, CO_2CH_3), 3.30 (3H, s, SO_2CH_3), 2.97 (2H, t, *J* = 7.8, $ArCH_2$), 2.75 (6H, s, $N(CH_3)_2$), 2.64 (2H, t, *J* 7.8, $CH_2CO_2CH_3$); **^{13}C NMR (125 MHz, $CDCl_3$)** δ 172.9 (Q), 152.2 (Q), 137.9 (Q), 136.7 (Q), 134.6, 129.0, 123.7, 51.8, 46.2, 42.6, 35.2, 30.1; **IR ν_{max}/cm^{-1} (neat)** 2950, 2790, 1736, 1491, 1438, 1299, 1141; **HRMS (ESI)** found *m/z* 286.1107 $[M+H]^+$, $C_{13}H_{20}NO_4S$ requires *m/z* 286.1108.

2-Methanesulfonyl-*N*,*N*-dimethyl-4-phenylaniline (12)

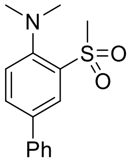

Prepared according to general procedure A. Column chromatography on silica (10-80% ethyl acetate in petroleum ether) afforded the titled sulfone as a colourless, crystalline solid (81 mg, 0.29 mmol, 59%); $\boldsymbol{R}_f$ 0.47 (40% ethyl acetate in petroleum ether); **mp** 125-126 °C (CH_2Cl_2); **^{1}H NMR (400 MHz, $CDCl_3$)** δ 8.29 (1H, d, *J* 2.3, Ar*H*), 7.81 (1H, dd, *J* 8.3, 2.3, Ar*H*), 7.61-7.56 (2H, m, Ar*H*), 7.49 (1H, d, *J* 8.3, Ar*H*), 7.47-7.42 (2H, m, Ar*H*), 7.39-7.34 (1H, m, Ar*H*), 3.35 (3H, s, SO_2CH_3), 2.83 (6H, s, $N(CH_3)_2$); **^{13}C NMR (100 MHz, $CDCl_3$)** δ 152.8 (Q), 138.9 (Q), 138.1 (Q), 136.8 (Q), 132.8, 128.9, 127.9, 127.8, 126.9, 123.9, 46.2, 42.4; **IR ν_{max}/cm^{-1} (neat)** 2981, 1478, 1387, 1301, 1135, 957, 773, 703; **HRMS (ESI)** found *m/z* 276.1061 $[M+H]^+$, $C_{15}H_{18}NO_2S$ requires *m/z* 276.1058.

2-Methanesulfonyl-6-methoxy-*N*,*N*-dimethylaniline (13a) and 4-methanesulfonyl-2-methoxy-*N*,*N*-dimethylaniline (13b)

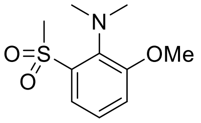

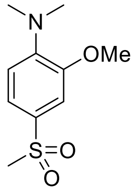

Prepared according to general procedure A. Column chromatography on silica (10-100% ethyl acetate in petroleum ether) afforded **13a** as a yellow amorphous solid (18 mg, 0.08 mmol, 16%) and **13b** as a pale yellow oil (28 mg, 0.12 mmol, 25%); **13a** $\boldsymbol{R}_f$ 0.25 (20% ethyl acetate in petroleum ether); **^{1}H NMR (400 MHz, $CDCl_3$)** δ 7.62 (1H, dd, *J* 8.0, 1.4, Ar*H*), 7.33-7.28 (1H, m, Ar*H*), 7.13 (1H, dd, *J* 8.3, 1.4 Hz, Ar*H*), 3.90 (3H, s, OCH_3), 3.32 (3H, s, SO_2CH_3), 2.81 (6H, s, $N(CH_3)_2$); **^{13}C NMR (100 MHz, $CDCl_3$)** δ 160.2 (Q), 140.9, (Q) 140.3 (Q), 126.9, 120.0, 117.1, 55.7, 43.4, 43.1; **IR ν_{max}/cm^{-1} (neat)** 3399, 2933, 1583, 1468, 1383, 1297, 1128, 959, 768; **HRMS (ESI)** found *m/z* 230.0847 $[M+H]^+$, $C_{10}H_{16}NO_3S$ requires *m/z* 230.0845. **13b** $\boldsymbol{R}_f$ 0.47 (50% ethyl acetate in petroleum ether); **^{1}H NMR (400 MHz, $CDCl_3$)** 7.47 (1H, dd, *J* 8.4, 2.1, Ar*H*), 7.31 (1H, d, *J* 2.1, Ar*H*), 6.93 (1H, d, *J* 8.4 Hz, Ar*H*), 3.93 (3H, s, OCH_3), 3.03 (3H, s, SO_2CH_3), 2.89 (s, 6H, $N(CH_3)_2$); **^{13}C NMR (100 MHz, $CDCl_3$)** δ 151.4 (Q), 147.1 (Q), 131.8 (Q), 121.2, 117.1, 109.6, 55.9, 44.9, 42.6; **IR ν_{max}/cm^{-1} (neat)** 3411, 2944, 1681, 1586, 1508, 1296, 1241, 1121, 959, 760; found *m/z* 230.0847 $[M+H]^+$, $C_{10}H_{16}NO_3S$ requires *m/z* 230.0845.

2-Methanesulfonyl-5-methoxy-*N*,*N*-dimethylaniline (14a), 2-methanesulfonyl-3-methoxy-*N*,*N*-dimethylaniline (14b) and 4-methanesulfonyl-3-methoxy-*N*,*N*-dimethylaniline (14c)

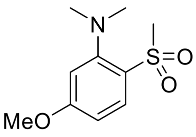

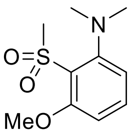

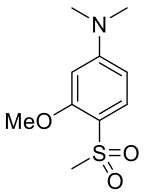

Prepared according to general procedure A. Column chromatography on silica (0-100% ethyl acetate in petroleum ether) afforded **14a** as a brown oil (13 mg, 0.06 mmol, 11%), **14b** as a red oil (10 mg,

0.05 mmol, 9%) and **14c** as a brown oil (47 mg, 0.21 mmol, 41%); **14a** $\boldsymbol{R_f}$ 0.09 (20% ethyl acetate in petroleum ether); **^{1}H NMR (400 MHz, $CDCl_3$)** δ 7.98 (1H, d, *J* 8.9, Ar*H*), 7.87 (1H, d, *J* 2.5, Ar*H*), 7.76 (1H, dd, *J* 8.9, 2.5, Ar*H*), 3.86 (3H, s, SO_2CH_3), 3.25, (3H, s, OCH_3), 2.77 (6H, s, $N(CH_3)_2$); **^{13}C NMR (100 MHz, $CDCl_3$)** δ 164.6 (Q), 156.1 (Q), 131.9, 128.8 (Q), 109.8, 109.3, 55.8, 46.4, 42.8; **IR ν_{max}/cm^{-1} (neat)** 3419, 3201, 1671, 1484, 1380, 1333, 1113; **HRMS (ESI)** found *m/z* 230.08472 $[M+H]^+$, $C_{10}H_{16}NO_3S$ requires *m/z* 230.08454.**14b** $\boldsymbol{R_f}$ 0.18 (50% ethyl acetate in petroleum ether); **^{1}H NMR (400 MHz, $CDCl_3$)** δ 7.36-7.31 (1H, m, Ar*H*), 6.71 (1H, dd, *J* 8.5, 0.9, Ar*H*), 6.53 (1H, dd, *J* 8.3, 0.9, Ar*H*), 3.94 (3H, s, SO_2CH_3), 3.28 (3H, s, OCH_3),2.93 (6H, s, $N(CH_3)_2$); **^{13}C NMR (100 MHz, $CDCl_3$)** δ 159.6 (Q), 154.8 (Q), 133.6, 118.8 (Q), 112.2, 103.8, 56.4, 46.4, 46.1; **IR ν_{max}/cm^{-1} (neat)** 2954, 2889, 2812, 1452, 1288, 1255, 1153, 1114; **HRMS (ESI)** found *m/z* 230.08467 $[M+H]^+$, $C_{10}H_{16}NO_3S$ requires *m/z* 230.08454. **14c** $\boldsymbol{R_f}$ 0.12 (50% ethyl acetate in petroleum ether); **^{1}H NMR (400 MHz, $CDCl_3$)** δ 7.73 (1H, d, *J* 9.0, Ar*H*), 6.27 (1H, dd, *J* 9.0, 2.3, Ar*H*), 6.12 (1H, d, *J* 2.3, Ar*H*), 3.96 (3H, s, SO_2CH_3), 3.15 (3H, s, OCH_3), 3.05 (6H, s, $N(CH_3)_2$); **^{13}C NMR (100 MHz, $CDCl_3$)** δ 158.9 (Q), 155.5 (Q), 131.1, 114.8 (Q), 103.4, 94.4, 56.0, 43.5, 40.4; **IR ν_{max}/cm^{-1} (neat)** 2944, 2788, 1591, 1567, 1289, 1231, 1142, 1122; **HRMS (ESI)** found *m/z* 230.08474 $[M+H]^+$, $C_{10}H_{16}NO_3S$ requires *m/z* 230.08474.

2-Methanesulfonyl-4-methoxy-*N*,*N*-dimethylaniline (15)

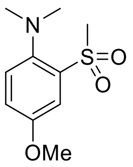

Prepared according to general procedure A. Following column chromatography on silica (0-60% ethyl acetate in petroleum ether), the material was redissolved in acetonitrile (1 mL) to which was added 2 M NaOH solution (1 mL). The mixture was stirred at room temperature for 3 h before extracting with ethyl acetate (3 x 1 mL). The combined organic extracts were dried ($MgSO_4$), filtered and the volatiles were removed under reduced pressure to afford the titled sulfone as a pale yellow, crystalline solid (93 mg, 0.41 mmol, 81%); $\boldsymbol{R_f}$ 0.17 (20% ethyl acetate in petroleum ether); **mp** 75-76 °C (CH_2Cl_2); **^{1}H NMR (400 MHz, $CDCl_3$)** δ 7.57-7.53 (1H, m, Ar*H*), 7.42-7.36 (1H, m, Ar*H*), 7.16-7.10 (1H, m, Ar*H*), 3.84 (3H, s, OCH_3), 3.34 (3H, s, SO_2CH_3), 2.73 (6H, s, $N(CH_3)_2$); **^{13}C NMR (100 MHz, $CDCl_3$)** δ 156.6 (Q), 146.5 (Q), 137.6 (Q), 124.9, 121.0, 112.7, 55.7, 46.2, 42.5; **IR ν_{max}/cm^{-1} (neat)** 2824, 2778, 1487, 1288, 1231, 1141, 1021, 767; **HRMS (ESI)** found *m/z* 230.0845 $[M+H]^+$, $C_{10}H_{16}NO_3S$ requires *m/z* 230.0845.

4-(Dimethylamino)-2-methanesulfonylphenol (16)

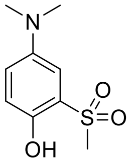

Prepared according to general procedure A. Column chromatography on silica (0-40% ethyl acetate in petroleum ether) afforded the titled sulfone as a colourless, crystalline solid (76 mg, 0.35 mmol, 71%); $\boldsymbol{R_f}$ 0.16 (40% ethyl acetate in petroleum ether); **mp** 110-112 °C (CH_2Cl_2); **^{1}H NMR (400 MHz, $CDCl_3$)** δ 8.07 (1H, br s, O*H*), 7.00-6.88 (3H, m, Ar*H*), 3.10 (3H, s, SO_2CH_3), 2.89 (6H, s, $N(CH_3)_2$); **^{13}C NMR (100 MHz, $CDCl_3$)** δ 146.8 (Q), 145.1 (Q), 122.9 (Q), 122.1, 119.7, 110.6, 44.6, 41.2; **IR**

ν_{max}/cm^{-1} (neat) 3356, 2926, 2801, 1502, 1423, 1287, 1125; **HRMS (ESI)** found *m/z* 214.0545 $[M+H]^+$, $C_9H_{12}NO_3S$ requires *m/z* 214.0543.

2-Methanesulfonyl-1-*N*,*N*,4-*N*,*N*-tetramethylbenzene-1,4-diamine (17)

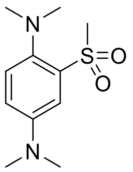

Prepared according to general procedure A. Column chromatography on silica (10-80% ethyl acetate in petroleum ether) afforded the titled sulfone as a colourless, crystalline solid (39 mg, 0.16 mmol, 32%); R_f 0.40 (50% ethyl acetate in petroleum ether); **mp** 134-136 °C (petroleum ether/ diethyl ether); **^{1}H NMR (400 MHz, CDCl$_3$)** δ 7.33-7.31 (2H, m, Ar*H*), 6.88 (1H, dd, *J* 8.8, 3.1, Ar*H*), 3.33 (3H, s, SO_2CH_3), 2.97 (6H, s, $N(CH_3)_2$), 2.70 (6H, s, $N(CH_3)_2$); **^{13}C NMR (100 MHz, CDCl$_3$)** δ 148.1 (Q), 142.2 (Q), 137.4 (Q), 124.7, 117.7, 111.8, 46.5, 42.9, 40.7; **IR ν_{max}/cm^{-1} (neat)** 2404, 2943, 2854, 2820, 1591, 1505, 1293, 1138, 1094; **HRMS (ESI)** found *m/z* 243.1162 $[M+H]^+$, $C_{11}H_{19}N_2O_2S$ requires *m/z* 243.1162.

***N*-[4-(Dimethylamino)-3-methanesulfonylphenyl]acetamide (18)**

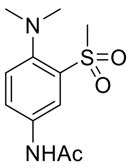

Prepared according to general procedure A. Column chromatography on silica (0-100% ethyl acetate in petroleum ether) afforded the titled sulfone as a yellow oil (70 mg, 0.28 mmol, 55%); R_f 0.1 (50% ethyl acetate in petroleum ether); **^{1}H NMR (400 MHz, CDCl$_3$)** δ 8.85 (1H, s (br), N*H*), 8.30 (1H, dd, *J* 8.9, 2.6, Ar*H*), 7.84 (1H, d, *J* 2.6, Ar*H*), 7.38 (1H, d, *J* 8.9, Ar*H*), 3.32 (3H, s, SO_2CH_3), 2.71 (3H, s, $COCH_3$), 2.18 (6H, s, $N(CH_3)_2$); **^{13}C NMR (100 MHz, CDCl$_3$)** δ 169.5 (Q), 149.2 (Q), 136.4 (Q), 136.1, 126.5, 124.7, 120.1 (Q), 46.3, 42.8, 24.4; **IR ν_{max}/cm^{-1} (neat)** 3329, 2833, 2790, 1671, 1525, 1490, 1294, 1095; **HRMS (ESI)** found *m/z* 257.09539 $[M+H]^+$, $C_{11}H_{17}N_2O_3S$ requires *m/z* 257.09544.

4-Chloro-2-methanesulfonyl-*N*,*N*-dimethylaniline (19)

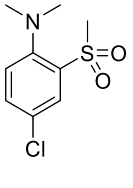

Prepared according to general procedure A. Column chromatography on silica (5-40% ethyl acetate in petroleum ether) afforded the titled sulfone as a yellow oil (22 mg, 0.09 mmol, 19%); R_f 0.12 (10% ethyl acetate in petroleum ether); **^{1}H NMR (500 MHz, CDCl$_3$)** δ 8.04 (1H, d, *J* 2.5, Ar*H*), 7.55 (1H, dd, *J* 8.4, 2.5, Ar*H*), 7.36 (1H, d, *J* 8.4, Ar*H*), 3.31 (3H, s, SO_2CH_3), 2.77 (6H, s, $N(CH_3)_2$); **^{13}C NMR (125 MHz, CDCl$_3$)** δ 152.6 (Q), 138.1 (Q), 134.6, 130.8 (Q), 129.6, 125.0, 46.2, 42.4; **IR ν_{max}/cm^{-1} (neat)** 2947, 2792, 1482, 1301, 1143, 1056; **HRMS (ESI)** found *m/z* 234.0351 $[M+H]^+$, $C_9H_{13}NO_2{}^{35}ClS$ requires *m/z* 234.0350.

4-Bromo-2-methanesulfonyl-*N*,*N*-dimethylaniline (20)

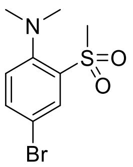

Prepared according to general procedure A. Column chromatography on silica (0-20% ethyl acetate in petroleum ether) afforded the titled sulfone as a pale yellow solid (26 mg, 0.10 mmol, 19%); $\boldsymbol{R}_f$ 0.12 (10% ethyl acetate in petroleum ether); **mp** 103-104 °C (CH_2Cl_2); **^{1}H NMR (400 MHz, $CDCl_3$)** δ 8.17 (1H, d, *J* 2.5, Ar*H*), 7.69 (1H, dd, *J* 8.4, 2.5, Ar*H*), 7.29 (1H, d, *J* 8.4, Ar*H*), 3.30 (3H, s, SO_2CH_3), 2.77 (6H, s, $N(CH_3)_2$); **^{13}C NMR (100 MHz, $CDCl_3$)** δ 153.0 (Q), 138.3 (Q), 137.5, 132.5, 125.3, 118.1 (Q), 46.1, 42.4; **IR ν_{max}/cm^{-1} (neat)** 2946, 2834, 1477, 1457, 1300, 1142; **HRMS (ESI)** found *m/z* 277.9846 $[M+H]^+$, $C_9H_{13}NO_2{}^{79}BrS$ requires *m/z* 277.9845.

4-Iodo-2-methanesulfonyl-*N*,*N*-dimethylaniline (21)

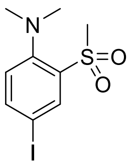

Prepared according to general procedure A. Column chromatography on silica (0-100% ethyl acetate in petroleum ether) afforded the titled sulfone as a yellow, crystalline solid (27 mg, 0.08 mmol, 17%); $\boldsymbol{R}_f$ 0.44 (40% ethyl acetate in petroleum ether); **mp** 121-123 °C (CH_2Cl_2); **^{1}H NMR (400 MHz, $CDCl_3$)** δ 8.35 (1H, d, *J* 2.2, Ar*H*), 7.90 (1H, dd, *J* 8.4, 2.2, Ar*H*), 7.17 (1H, d, *J* 8.4, Ar*H*), 3.30 (3H, s, SO_2CH_3), 2.78 (6H, s, $N(CH_3)_2$); **^{13}C NMR (100 MHz, $CDCl_3$)** δ 153.6 (Q), 143.4, 138.2 (Q), 138.1, 125.5, 88.3 (Q), 46.1, 42.3; **IR ν_{max}/cm^{-1} (neat)** 2980, 1474, 1375, 1295, 1137, 1056, 965; **HRMS (ESI)** found *m/z* 325.9718 $[M+H]^+$, $C_9H_{13}NO_2SI$ requires *m/z* 325.9712.

2-Methanesulfonyl-5-methoxy-*N*,*N*,4-trimethylaniline (22a) and 2-Methanesulfonyl-3-methoxy-*N*,*N*,4-trimethylaniline (22b)

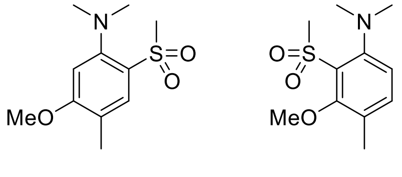

Prepared according to general procedure A. Following column chromatography on silica (10-60% ethyl acetate in petroleum ether), the material was redissolved in acetonitrile (1 mL) to which was added 2 M NaOH solution (1 mL). The mixture was stirred at room temperature for 3 h before extracting with ethyl acetate (3 x 1 mL). The combined organic extracts were dried ($MgSO_4$), filtered and the volatiles were removed under reduced pressure to afford a mixture of the titled sulfones as a colourless, crystalline solid (98 mg, 0.40 mmol, 80%, **22a**/**22b** 7:1); $\boldsymbol{R}_f$ 0.32 (40% ethyl acetate in petroleum ether); **^{1}H NMR (400 MHz, $CDCl_3$)** δ **22a** 7.78 (1H, s, Ar*H*), 6.84 (1H, s, Ar*H*), 3.90 (3H, s, OCH_3), 3.27 (3H, s, SO_2CH_3), 2.79 (6H, s, $N(CH_3)_2$), 2.20 (3H, s, $ArCH_3$), **22b** 7.29 (1H, d, *J* 8.5, Ar*H*), 6.88 (1H, d, *J* 8.5, Ar*H*), 3.89 (3H, s, OCH_3), 3.33 (3H, s, SO_2CH_3), 2.86 (6H, s, $N(CH_3)_2$), 2.26 (3H, s, $ArCH_3$); **^{13}C NMR (100 MHz, $CDCl_3$)** δ **22a** 162.2 (Q), 153.7 (Q), 130.8, 127.5 (Q), 123.6 (Q), 104.3, 55.5, 46.1, 42.7, 15.5, **22b** 158.0 (Q), 152.6 (Q), 135.7, 125.3 (Q), 115.6, 93.2 (Q), 62.4, 46.2, 30.0, 15.3; **HRMS (ESI)** found *m/z* 244.1016 $[M+H]^+$, $C_{11}H_{18}NO_3S$ requires *m/z* 244.1007.

2-Methanesulfonyl-4,5-dimethoxy-*N*,*N*-dimethylaniline (23)

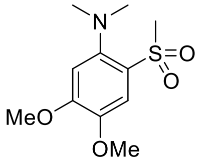

Prepared according to general procedure A. Column chromatography on silica (0-100% ethyl acetate in petroleum ether) afforded the titled sulfone as a pale brown solid (65 mg, 0.25 mmol, 50%); $\boldsymbol{R}_{\mathbf{f}}$ 0.13 (20% ethyl acetate in petroleum ether); **mp** 98-102 °C (Et_2O); **^{1}H NMR (400 MHz, $CDCl_3$)** δ 7.46 (1H, s, Ar*H*), 6.87 (1H, s, Ar*H*), 3.91 (3H, s, OCH_3), 3.88 (3H, s, OCH_3), 3.27 (3H, s, SO_2CH_3), 2.71 (6H, s, $N(CH_3)_2$); **^{13}C NMR (100 MHz, $CDCl_3$)** δ 153.5 (Q), 147.8 (Q), 145.9 (Q), 128.4 (Q), 110.8, 105.8, 56.2, 56.1, 46.2, 42.8; **IR ν_{max}/cm^{-1} (neat)** 2937, 1505, 1291, 1252, 1217, 1126, 1048, 989, 760; **HRMS (ESI)** found *m/z* 260.0953 $[M+H]^+$, $C_{11}H_{18}NO_4S$ requires *m/z* 260.0951.

8-Methanesulfonyl-1-methyl-1,2,3,4-tetrahydroquinoline (24a) and 6-methanesulfonyl-1-methyl-1,2,3,4-tetrahydroquinoline (24b)

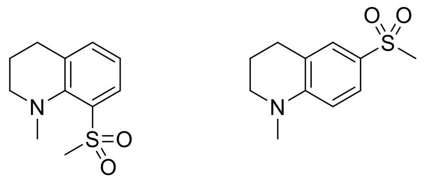

Prepared according to general procedure A. Column chromatography on silica (5-60% ethyl acetate in petroleum ether) afforded **24a** as a pale yellow oil (15 mg, 0.07 mmol, 13%) and **24b** as a colourless, amorphous solid (17 mg, 0.08 mmol, 15%); **24a** $\boldsymbol{R}_{\mathbf{f}}$ 0.22 (20% ethyl acetate in petroleum ether); **^{1}H NMR (400 MHz, $CDCl_3$)** δ 7.91-7.87 (1H, m, Ar*H*), 7.30-7.26 (1H, m, Ar*H*), 7.14-7.08 (1H, m, Ar*H*), 3.28 (3H, s, SO_2CH_3), 3.12-3.07 (2H, m, NCH_2), 2.96 (3H, s, NCH_3), 2.89 (2H, t, *J* 7.0, $ArCH_2$), 2.01-1.93 (2H, m, $ArCH_2CH_2$); **^{13}C NMR (125 MHz, $CDCl_3$)** δ 149.0 (Q), 135.0, 134.7 (Q), 132.6 (Q), 128.0, 122.9, 50.4, 46.0, 41.2, 26.7, 15.5; **IR ν_{max}/cm^{-1} (neat)** 2946, 1461, 1297, 1153, 1125, 820; **HRMS (ESI)** found *m/z* 226.0897 $[M+H]^+$, $C_{11}H_{16}NO_2S$ requires *m/z* 226.0896. **24b** $\boldsymbol{R}_{\mathbf{f}}$ 0.06 (20% ethyl acetate in petroleum ether); **^{1}H NMR (400 MHz, $CDCl_3$)** δ 7.58 (1H, dd, *J* 8.7, 2.4, Ar*H*), 7.45-7.42 (1H, m, Ar*H*), 6.55 (1H, d, *J* 8.7, Ar*H*), 3.39-3.34 (2H, m, NCH_2), 3.00 (3H, s, SO_2CH_3), 2.98 (3H, s, NCH_3), 2.78 (2H, t, *J* 6.3, $ArCH_2$), 2.01-1.93 (2H, m, $ArCH_2CH_2$); **^{13}C NMR (125 MHz, $CDCl_3$)** δ 150.1 (Q), 127.5, 127.3, 125.2 (Q), 122.1 (Q), 109.3, 50.9, 45.1, 38.7, 27.7, 21.4; **IR ν_{max}/cm^{-1} (neat)** 2980, 1597, 1519, 1295, 1135; **HRMS (ESI)** found *m/z* 226.0897 $[M+H]^+$, $C_{11}H_{16}NO_2S$ requires *m/z* 226.0896.

5-Methanesulfonyl-4-methyl-3,4-dihydro-2*H*-1,4-benzoxazine (25a), 7-methanesulfonyl-4-methyl-3,4-dihydro-2*H*-1,4-benzoxazine (25b) and 6-methanesulfonyl-4-methyl-3,4-dihydro-2*H*-1,4-benzoxazine (25c)

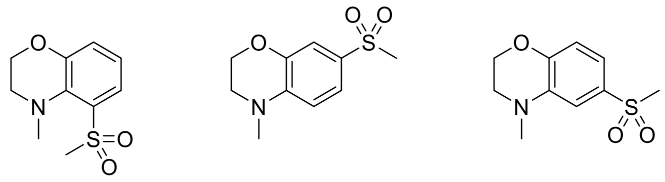

Prepared according to general procedure A. Column chromatography on silica (0-100% ethyl acetate in petroleum ether) afforded **25a** as a pale yellow oil (22 mg, 0.1 mmol, 20%) and **25b** and **25c** as a red oil as an inseparable mixture (40 mg, 0.18 mmol, 35%, **25b**/**25c** 1.8:1); **25a** ***R*f** 0.32 (50% ethyl acetate in petroleum ether); **^{1}H NMR (400 MHz, CDCl$_3$)** δ 7.68 (1H, dd, *J* 7.4 2.0, Ar*H*), 7.68 (2H, m, Ar*H*), 4.36-4.32 (2H, m, C*H$_2$*), 3.27 (3H, s, SO$_2$C*H$_3$*), 3.14-3.09 (2H, m, C*H$_2$*), 3.01 (3H, s, NC*H$_3$*), **^{13}C NMR (100 MHz, CDCl$_3$)** δ 149.8 (Q), 135.9 (Q), 134.9 (Q), 124.5, 123.0, 122.1, 58.9, 48.5, 46.5, 46.5, 42.1; **IR ν_{max}/cm^{-1} (neat)** 2954, 2888, 1812, 1452, 1347, 1233, 1134; **HRMS (ESI)** found *m/z* 228.0689 [M+H]$^+$, $C_{10}H_{14}O_3NS$ requires *m/z* 228.0689. **25b and 25c** ***R*f** 0.18 (50% ethyl acetate in petroleum ether); **^{1}H NMR (400 MHz, CDCl$_3$)** δ **25b** 7.39 (1H, dd, *J* 8.5, 2.2, Ar*H*), 7.26 (1H, d, *J* 2.2, Ar*H*), 6.65 (1H, d, *J* 8.5, Ar*H*), 4.28-4.25 (2H, m, C*H$_2$*), 3.42-3.38 (2H, m, C*H$_2$*), 2.99 (3H, s, SO$_2$C*H$_3$*) 2.98 (3H, s, NC*H$_3$*), **25c** 7.20 (1H, dd, *J* 8.3, 2.2, Ar*H*), 7.12 (1H, d, *J* 2.2, Ar*H*), 6.85 (1H, d, *J* 8.3, Ar*H*), 4.36-4.32 (2H, m, C*H$_2$*), 3.34-3.30 (2H, m, C*H$_2$*), 3.02 (3H, s, SO$_2$C*H$_3$*), 2.94 (3H, s, NC*H$_3$*); **^{13}C NMR (100 MHz, CDCl$_3$)** δ **25b** 143.3 (Q), 140.8 (Q), 127.5 (Q), 121.9, 114.6, 110.6, 64.2, 48.5, 45.0, 38.3, **25c** 148.4 (Q), 136.9 (Q), 132.9 (Q), 117.7, 116.2, 110.5, 65.1, 48.2, 44.9, 38.6; **IR ν_{max}/cm^{-1} (neat)** 2927, 2884, 1598, 1510, 1295, 1212, 1134; **HRMS (ESI)** found *m/z* 228.0691 [M+H]$^+$, $C_{10}H_{14}O_3NS$ requires *m/z* 228.0689.

3-Methanesulfonyl-10-methyl-10H-phenoxazine (26)

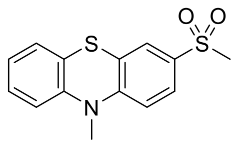

Prepared according to general procedure A. Column chromatography on silica (0-100% ethyl acetate in petroleum ether) afforded the titled sulfone as a pale yellow, crystalline solid (85 mg, 0.29 mmol, 58%); ***R*f** 0.10 (20% ethyl acetate in petroleum ether); **mp** 134-136 °C (Et$_2$O); **^{1}H NMR (400 MHz, CDCl$_3$)** δ 7.70 (1H, dd, *J* 8.6, 2.2, Ar*H*), 7.63 (1H, d, *J* 2.2, Ar*H*), 7.25-7.16 (1H, m, Ar*H*), 7.13 (1H, dd, *J* 7.7, 1.5, Ar*H*), 7.04-6.95 (1H, m, Ar*H*), 6.89-6.82 (2H, m, Ar*H*), 3.42 (3H, s, NC*H$_3$*), 3.01 (3H, s, SO$_2$C*H$_3$*); **^{13}C NMR (100 MHz, CDCl$_3$)** δ 150.5 (Q), 144.1 (Q), 133.6 (Q), 128.0, 127.4, 127.4, 126.0, 124.8 (Q), 123.8, 122.2 (Q), 114.8, 113.7, 44.9, 35.8; **IR ν_{max}/cm^{-1} (neat)** 3060, 2924, 1566, 1459, 1299, 1150, 1138, 957, 751, 728; **HRMS (ESI)** found *m/z* 292.0461 [M+H]$^+$, $C_{14}H_{14}NO_2S_2$ requires *m/z* 292.0461.

2-Methanesulfonyl-6-methoxy-*N*,*N*-dimethylpyridin-3-amine (27a) and 4-methanesulfonyl-6-methoxy-*N*,*N*-dimethylpyridin-3-amine (27b)

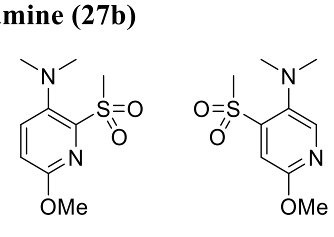

Prepared according to general procedure A. Column chromatography on silica (0-100% ethyl acetate in petroleum ether) afforded **27a** as a pale yellow, crystalline solid (18 mg, 0.08 mmol, 16%) and **27b** as a yellow, amorphous solid (12 mg, 0.05 mmol, 10%); **27a** ***R*f** 0.23 (40% ethyl acetate in petroleum ether); **mp** 102-103 °C (CH$_2$Cl$_2$); **^{1}H NMR (400 MHz, CDCl$_3$)** δ 7.68 (1H, d, *J* 8.9, Ar*H*), 6.94 (1H, d, *J* 8.9, Ar*H*), 3.93 (3H, s, OC*H$_3$*), 3.34 (3H, s, SO$_2$C*H$_3$*), 2.83 (6H, s, N(C*H$_3$*)$_2$); **^{13}C NMR (125 MHz, CDCl$_3$)** δ 158.2 (Q), 147.4 (Q), 142.9 (Q), 134.7, 115.9, 54.0, 45.6, 41.3; **IR ν_{max}/cm^{-1} (neat)** 2980, 1672, 1484, 1296, 1269, 1148, 1123, 1021, 957, 756; **HRMS (ESI)** found *m/z* 253.0624

[M+Na]$^+$, $C_9H_{14}NaNO_3S$ requires *m/z* 253.0623. **27b** ***R*$_f$** 0.11 (40% ethyl acetate in petroleum ether); **^{1}H NMR (400 MHz, $CDCl_3$)** δ 7.89 (1H, d, *J* 3.1, Ar*H*), 7.70 (1H, d, *J* 3.1, Ar*H*), 4.05 (3H, s, OC*H*$_3$), 3.24 (3H, s, SO_2C*H*$_3$), 2.96 (6H, s, N(C*H*$_3$)$_2$); **^{13}C NMR (125 MHz, $CDCl_3$)** δ 153.0 (Q), 141.2 (Q), 137.1, 124.6, 122.6 (Q), 54.4, 42.3, 41.6; **IR ν_{max}/cm^{-1} (neat)** 2980, 1573, 1395, 1146, 958; **HRMS (ESI)** found *m/z* 231.0809 [M+H]$^+$, $C_9H_{15}N_2O_3S$ requires *m/z* 231.0803.

2-Methanesulfonyl-*N*,*N*-dimethylaniline (28a) and 4-methanesulfonyl-*N*,*N*-dimethylaniline (28b)

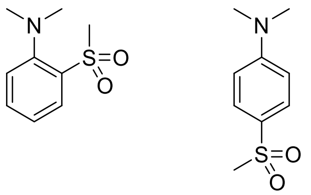

Prepared according to general procedure A. Column chromatography on silica (0-100% ethyl acetate in petroleum ether) afforded **28a** as a colourless solid (29 mg, 0.15 mmol, 29%) and **28b** as a colourless, crystalline solid (24 mg, 0.12 mmol, 24%); **28a** ***R*$_f$** 0.25 (20% ethyl acetate in petroleum ether); **mp** 92-95 °C (Et_2O), lit. 94-95 °C; **^{1}H NMR (400 MHz, $CDCl_3$)** δ 8.06 (1H , dd, *J* 8.0, 1.6, Ar*H*), 7.62-7.58 (1H, m, Ar*H*), 7.43 (1H, dd, *J* 8.0, 0.9, Ar*H*), 7.34 – 7.27 (1H, m, Ar*H*), 3.31 (3H, s, SO_2C*H*$_3$), 2.79 (6H, s, N(C*H*$_3$)$_2$); **^{13}C NMR (100 MHz, $CDCl_3$)** δ 154.1 (Q), 136.7 (Q), 134.7, 129.6, 125.2, 123.6, 46.3, 42.5; **LRMS** found *m/z* 200.1 [M+H]$^+$, $C_9H_{14}NO_2S$ requires *m/z* 200.1. Data is consistent with that given in the literature.[4] **28b** ***R*$_f$** 0.27 (50% ethyl acetate in petroleum ether); **mp** 152-154 °C (Et_2O), lit. 163-166 °C; **^{1}H NMR (400 MHz, $CDCl_3$)** δ 7.76 – 7.70 (2H, m, Ar*H*), 6.72 – 6.67 (2H, m, Ar*H*), 3.06 (6H, s, N(C*H*$_3$)$_2$), 3.00 (3H, s, SO_2C*H*$_3$); **^{13}C NMR (100 MHz, $CDCl_3$)** δ 153.4 (Q), 129.1, 125.8 (Q), 111.0, 45.1, 40.1; **LRMS** found *m/z* 200.1 [M+H]$^+$, $C_9H_{14}NO_2S$ requires *m/z* 200.1. Data is consistent with that given in the literature.[5]

2-Methanesulfonyl-*N*,*N*-diethylaniline (29a) and 4-methanesulfonyl-*N*,*N*-diethylaniline (29b)

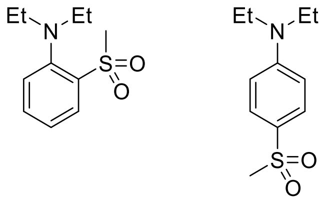

Prepared according to general procedure A. Column chromatography on silica (0-100% ethyl acetate in petroleum ether) afforded **29a** as a pale yellow oil (17 mg, 0.08 mmol, 15%) and **29b** as a pale yellow oil (24 mg, 0.11 mmol, 21%); **29a** ***R*$_f$** 0.44 (20% ethyl acetate in petroleum ether); **^{1}H NMR (500 MHz, $CDCl_3$)** δ 8.12 (1H, dd, *J* 8.0, 1.7, Ar*H*), 7.60-7.56 (1H, m, Ar*H*), 7.34 (1H, d, *J* 8.0, Ar*H*), 7.31-7.27 (1H, m, Ar*H*), 3.37 (3H, s, SO_2C*H*$_3$), 3.14 (4H, q, *J* 7.1, N(C*H*$_2$CH$_3$)$_2$), 1.07 (6H, t, *J* 7.1, N(CH$_2$C*H*$_3$)$_2$); **^{13}C NMR (125 MHz, $CDCl_3$)** δ 151.1 (Q), 137.8 (Q), 133.9, 129.9, 125.2, 124.9, 48.4, 42.7, 12.1; **IR ν_{max}/cm^{-1} (neat)** 3385, 2973, 2933, 2820, 1472, 1298, 1143, 955, 776, 735; **HRMS (ESI)** found *m/z* 228.1053 [M+H]$^+$, $C_{11}H_{18}NO_2S$ requires *m/z* 228.1053. **29b** ***R*$_f$** 0.45 (50% ethyl acetate in petroleum ether); **^{1}H NMR (500 MHz, $CDCl_3$)** δ 7.70 (2H, d, *J* 9.1, Ar*H*), 6.67 (2H, d, *J* 9.1, Ar*H*), 3.42 (4H, q, *J* 7.1, N(C*H*$_2$CH$_3$)$_2$), 3.00 (3H, s, SO_2C*H*$_3$), 1.20 (6H, t, *J* 7.1, N(CH$_2$C*H*$_3$)$_2$); **^{13}C NMR (125 MHz, $CDCl_3$)** δ 151.2 (Q), 129.3, 125.0 (Q), 110.5, 45.2, 44.6 (CH$_2$, 12.3; **IR ν_{max}/cm^{-1} (neat)** 2965, 2851, 1593, 1300, 1140, 955, 774; **HRMS (ESI)** found *m/z* 228.1053 [M+H]$^+$, $C_{11}H_{18}NO_2S$ requires *m/z* 228.1053.

1-(2-Methanesulfonylphenyl)pyrrolidine (30a) and 1-(4-methanesulfonylphenyl)pyrrolidine (30b)

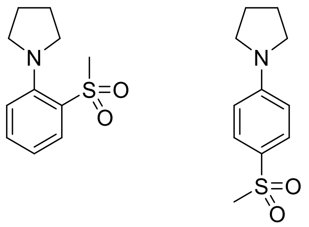

Prepared according to general procedure A. Column chromatography on silica (0-100% ethyl acetate in petroleum ether) afforded **30a** as a colourless oil (19 mg, 0.08 mmol, 17%) and **30b** as a white solid (11 mg, 0.05 mmol, 9%); **30a** R_f 0.13 (10% ethyl acetate in petroleum ether; **^{1}H NMR (500 MHz, $CDCl_3$)** δ 8.06 (1H, dd, *J* 7.9, 1.5, Ar*H*), 7.57-7.53 (1H, m, Ar*H*), 7.36 (1H, dd, *J* 7.9, 1.0, Ar*H*), 7.24-7.21 (1H, m, Ar*H*), 3.29 (3H, s, SO_2CH_3), 3.26-3.23 (4H, m, $N(CH_2)_2$), 1.98-1.95 (4H, m, (CH_2CH_2); **^{13}C NMR (125 MHz, $CDCl_3$)** δ 150.6 (Q), 136.4 (Q), 134.4, 129.7, 123.9, 123.1, 54.4, 42.7, 24.9; **IR ν_{max}/cm^{-1} (neat)** 2967, 2825, 1589, 1473, 1297, 1146; **HRMS (ESI)** found *m/z* 226.0896 $[M+H]^+$, $C_{11}H_{16}NO_2S$ requires *m/z* 226.0896. **30b** R_f 0.26 (20% ethyl acetate in petroleum ether); **mp** 167-171 °C (CH_2Cl_2); **^{1}H NMR (500 MHz, $CDCl_3$)** δ 7.72-7.70 (2H, m, Ar*H*), 6.57-6.54 (2H, m, Ar*H*), 3.36-3.33 (4H, m, $N(CH_2)_2$), 2.99 (3H, s, SO_2CH_3), 2.06-2.03 (4H, m, (CH_2CH_2); **^{13}C NMR (125 MHz, $CDCl_3$)** δ 151.0 (Q), 129.1, 125.2 (Q), 111.1, 47.6, 45.2, 24.9; **IR ν_{max}/cm^{-1} (neat)** 2980, 2856, 1599, 1511, 1390, 1304, 1141; **HRMS (ESI)** found *m/z* 226.0897 $[M+H]^+$, $C_{11}H_{16}NO_2S$ requires *m/z* 226.0896.

1-(2-Methanesulfonylphenyl)piperidine (31a) and 1-(4-methanesulfonylphenyl)piperidine (31b)

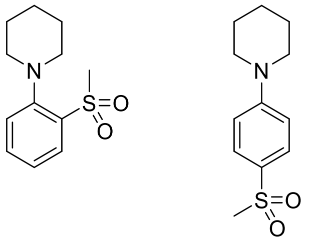

Prepared according to general procedure A. Column chromatography on silica (0-50% ethyl acetate in petroleum ether) afforded **31a** as a colourless oil (8 mg, 0.03 mmol, 6%) and **31b** as a pale yellow solid (11 mg, 0.05 mmol, 9%); **31a** R_f 0.16 (10% ethyl acetate in petroleum ether); **^{1}H NMR (500 MHz, $CDCl_3$)** δ 8.06 (1H, dd, *J* 8.1, 1.1, Ar*H*), 7.63-7.60 (1H, m, Ar*H*), 7.40 (1H, dd, *J* 8.1, 1.0, Ar*H*), 7.34-7.30 (1H, m, Ar*H*), 3.39 (3H, s, SO_2CH_3), 3.09-2.88 (4H, m, $N(CH_2)_2$), 1.95-1.18 (4H, m, ($CH_2CH_3CH_2$), 1.67-1.60 (2H, m, ($CH_2CH_3CH_2$); **^{13}C NMR (125 MHz, $CDCl_3$)** δ 153.9 (Q), 137.6 (Q), 134.5, 129.7, 125.3, 123.9, 55.3, 42.6, 26.4, 24.0; **IR ν_{max}/cm^{-1} (neat)** 2933, 2852, 1587, 1475, 1301, 1148; **HRMS (ESI)** found *m/z* 240.1053 $[M+H]^+$, $C_{12}H_{18}NO_2S$ requires *m/z* 240.1053. **31b** R_f 0.41 (10% ethyl acetate in petroleum ether); **mp** 112-114 °C (CH_2Cl_2); **^{1}H NMR (500 MHz, $CDCl_3$)** δ 7.74-7.71 (2H, m, Ar*H*), 6.92-6.89 (2H, m, Ar*H*), 3.38-3.33 (4H, m, $N(CH_2)_2$), 3.01 (3H, s, SO_2CH_3), 1.70-1.64 (6H, m, ($CH_2CH_2CH_2$); **^{13}C NMR (125 MHz, $CDCl_3$)** δ 154.5 (Q), 129.1, 127.4 (Q), 113.6, 48.6, 45.0, 25.2, 24.3; **IR ν_{max}/cm^{-1} (neat)** 2932, 2853, 1590, 1508, 1294, 1144, 1124; **HRMS (ESI)** found *m/z* 240.1053 $[M+H]^+$, $C_{12}H_{18}NO_2S$ requires *m/z* 240.1055.

1-(2-Methanesulfonylphenyl)morpholine (32a) and 1-(4-methanesulfonylphenyl)morpholine (32b)

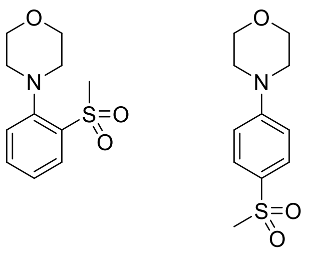

Prepared according to general procedure A. Column chromatography on silica (0-50% ethyl acetate in petroleum ether) afforded **32a** as a pale orange solid (10 mg, 0.04 mmol, 8%) and **32b** as a white solid (20 mg, 0.08 mmol, 17%); **32a** ***R*f** 0.24 (40% ethyl acetate in petroleum ether); **mp** 178-180 °C (CH_2Cl_2); **^{1}H NMR (500 MHz, $CDCl_3$)** δ 8.11 (1H, dd, *J* 7.9, 1.5, Ar*H*), 7.68-7.64 (1H, m, Ar*H*), 7.43 (1H, dd, *J* 7.9, 1.0, Ar*H*), 7.39-7.36 (1H, m, Ar*H*), 3.93-3.90 (4H, m, O(C*H*$_2$)$_2$), 3.39 (3H, s, SO_2C*H*$_3$), 3.11-3.09 (4H, m, N(C*H*$_2$)$_2$); **^{13}C NMR (125 MHz, $CDCl_3$)** δ 152.1 (Q), 137.0 (Q), 134.9, 130.1, 125.8, 123.8, 67.4, 54.1, 42.8; **IR ν_{max}/cm^{-1} (neat)** 2959, 2853, 1588, 1474, 1450, 1298, 1145, 1112; **HRMS (ESI)** found *m/z* 242.0846 $[M+H]^+$, $C_{11}H_{16}NO_3S$ requires *m/z* 242.0845. **32b** ***R*f** 0.10 (40% ethyl acetate in petroleum ether); **mp** 170-172 °C (CH_2Cl_2); **^{1}H NMR (500 MHz, $CDCl_3$)** δ 7.80-7.77 (2H, m, Ar*H*), 6.95-6.92 (2H, m, Ar*H*), 3.87-3.84 (4H, m, O(C*H*$_2$)$_2$), 3.32-3.29 (4H, m, N(C*H*$_2$)$_2$), 3.01 (3H, s, SO_2C*H*$_3$); **^{13}C NMR (125 MHz, $CDCl_3$)** δ 154.4 (Q), 129.4 (Q), 129.1, 113.8, 66.5, 47.5, 45.0; **IR ν_{max}/cm^{-1} (neat)** 2921, 2858, 1594, 1293, 1249, 1141, 1122; **HRMS (ESI)** found *m/z* 242.0847 $[M+H]^+$, $C_{11}H_{16}NO_3S$ requires *m/z* 242.0845.

2-Methanesulfonyl-*N*,*N*-diphenylaniline (33a) and 4-methanesulfonyl-*N*,*N*-diphenylaniline (33b)

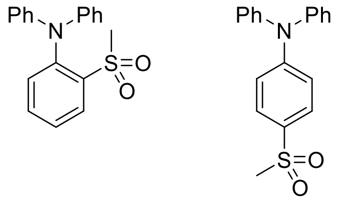

Prepared according to general procedure A. Column chromatography on silica (0-100% ethyl acetate in petroleum ether) afforded **33a** as an amorphous brown solid (12 mg, 0.04 mmol, 7%) and **33b** as an off-white, amorphous solid (115 mg, 0.36 mmol, 71%); **33a** ***R*f** 0.74 (40% ethyl acetate in petroleum ether); **^{1}H NMR (400 MHz, $CDCl_3$)** δ 8.19 (1H, dd, *J* 8.0, 1.6, Ar*H*), 7.70-7.65 (1H, m, Ar*H*), 7.50-7.45 (1H, m, Ar*H*), 7.37 (1H, dd, *J* 8.0, 1.3, Ar*H*), 7.30-7.22 (4H, m, Ar*H*), 7.06-6.98 (6H, m, Ar*H*), 2.48 (3H, s, SO_2C*H*$_3$); **^{13}C NMR (100 MHz, $CDCl_3$)** δ 147.8 (Q), 146.2 (Q), 138.8 (Q), 135.3, 133.2, 131.8, 129.2, 126.8, 122.9, 122.7, 42.6; **IR ν_{max}/cm^{-1} (neat)** 3061, 3035, 1585, 1489, 1470, 1310, 1146, 754, 695; **HRMS (ESI)** found *m/z* 324.1054 $[M+H]^+$, $C_{19}H_{18}NO_2S$ requires *m/z* 324.1053. **33b** ***R*f** 0.59 (40% ethyl acetate in petroleum ether); **^{1}H NMR (400 MHz, $CDCl_3$)** δ 7.67-7.55 (2H, m, Ar*H*), 7.31-7.22 (4H, m, Ar*H*), 7.12-7.04 (6H, m, Ar*H*), 6.98-6.93 (2H, m, Ar*H*), 2.96 (3H, s, SO_2C*H*$_3$); **^{13}C NMR (100 MHz, $CDCl_3$)** δ 152.6 (Q), 146.1 (Q), 130.9 (Q), 129.8, 128.8, 126.2, 125.2, 119.5, 44.8; **IR ν_{max}/cm^{-1} (neat)** 3062, 1582, 1488, 1299, 1142, 956, 697; **HRMS (ESI)** found *m/z* 324.1054 $[M+H]^+$, $C_{19}H_{18}NO_2S$ requires *m/z* 324.1053.

2-Methanesulfonyl-4-methoxy-*N*,*N*-diphenylaniline (34a), 4-methanesulfonyl-*N*-(4-methoxyphenyl)-*N*-phenylaniline (34b), and 3-methanesulfonyl-4-methoxy-*N*,*N*-diphenylaniline (34c)

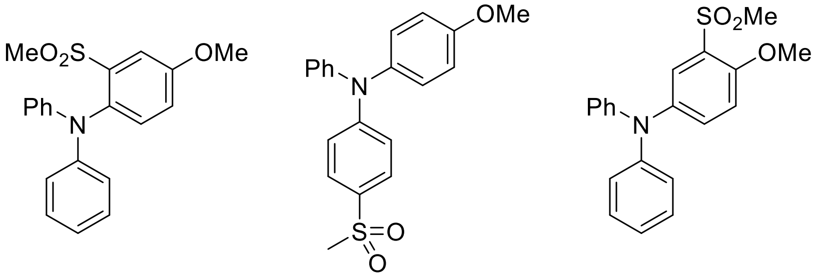

Prepared according to general procedure A. Column chromatography on silica (0-100% ethyl acetate in petroleum ether) afforded **34a** as a pale yellow oil (42 mg, 0.12 mmol, 24%), **34b** as a colourless solid (94 mg, 0.27 mmol, 53%) and **34c** as an off-white solid (17 mg, 0.05 mmol, 10%); **34a** $\boldsymbol{R}_\mathbf{f}$ 0.71 (20% ethyl acetate in petroleum ether); **^{1}H NMR (400 MHz, CDCl$_3$)** δ 7.70 (1H, d, *J* 3.0 Ar*H*), 7.32-7.17 (6H, m, Ar*H*), 7.07-6.94 (6H, m, Ar*H*), 3.92 (3H, s OC*H$_3$*), 2.55 (3H, s, SO$_2$C*H$_3$*); **^{13}C NMR (100 MHz, CDCl$_3$)** δ 158.0 (Q), 147.8 (Q), 139.8 (Q), 138.2 (Q), 134.6, 129.1, 122.4, 122.3, 121.9, 115.1, 56.0, 42.7; **IR ν_{max}/cm^{-1} (neat)** 3035, 2839, 1587, 1488, 1310, 1147, 758; **HRMS (ESI)** found *m/z* 354.1159 [M+H]$^+$, $C_{20}H_{20}NO_3S$ requires *m/z* 354.1158. **34b** $\boldsymbol{R}_\mathbf{f}$ 0.48 (40% ethyl acetate in petroleum ether); **mp** 105-108 °C (Et$_2$O); **^{1}H NMR (400 MHz, CDCl$_3$)** δ 7.69-7.62 (2H, m, Ar*H*), 7.36-7.29 (2H, m, Ar*H*), 7.18-7.08 (5H, m, Ar*H*), 6.99-6.86 (4H, m, Ar*H*), 3.82 (3H, s OC*H$_3$*), 3.03 (3H, s, SO$_2$C*H$_3$*); **^{13}C NMR (100 MHz, CDCl$_3$)** δ 157.5 (Q), 152.9 (Q), 146.1 (Q), 138.7 (Q), 130.0, 129.7, 128.7, 128.4, 125.7, 124.9, 118.2, 115.2, 55.5, 44.9; **IR ν_{max}/cm^{-1} (neat)** 3038, 2930, 2837, 1583, 1506, 1489, 1296, 1242, 1139, 1092, 829, 772, 726; **HRMS (ESI)** found *m/z* 354.1159 [M+H]$^+$, $C_{20}H_{20}NO_3S$ requires *m/z* 354.1158. **34c** $\boldsymbol{R}_\mathbf{f}$ 0.37 (40% ethyl acetate in petroleum ether); **mp** 186-188 °C (CH_2Cl_2); **^{1}H NMR (400 MHz, CDCl$_3$)** δ 7.75 (1H, d, *J* 2.8, Ar*H*), 7.33 (1H, dd, *J* 8.9, 2.9, Ar*H*), 7.28-7.22 (4H, m, Ar*H*), 7.06-6.99 (6H, m, Ar*H*), 6.97 (1H, d, *J* 8.9, Ar*H*), 3.99 (3H, s, OC*H$_3$*), 3.24 (3H, s, SO$_2$C*H$_3$*); **^{13}C NMR (100 MHz, CDCl$_3$)** δ 152.8 (Q), 147.4 (Q), 141.4 (Q), 131.7, 129.4, 129.3 (Q), 125.7, 123.6, 123.0, 113.6, 56.7, 43.0; **IR ν_{max}/cm^{-1} (neat)** 3035, 2942, 1588, 1489, 1306, 1279, 1142, 728, 697; **HRMS (ESI)** found *m/z* 354.1159 [M+H]$^+$, $C_{20}H_{20}NO_3S$ requires *m/z* 354.1158.

2-(Cyclopropanesulfonyl)-*N*,*N*,4-trimethylaniline (35)

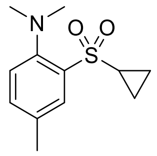

Prepared according to general procedure B. Column chromatography on silica (0-10% ethyl acetate in petroleum ether) afforded the titled sulfone as a yellow oil (29 mg, 0.12 mmol, 60%); $\boldsymbol{R}_\mathbf{f}$ 0.21 (20% ethyl acetate in petroleum ether); **^{1}H NMR (400 MHz, CDCl$_3$)** δ 7.72 (1H, d, *J* 1.6, Ar*H*), 7.38 (1H, dd, *J* 8.1, 1.6, Ar*H*), 7.34 (1H, d, *J* 8.1, Ar*H*), 3.41-3.35 (1H, m, SO$_2$C*H*), 2.75 (3H, s, ArC*H$_3$*), 2.36 (6H, s, N(C*H$_3$*)$_2$), 1.26-1.22 (2H, m, C*H$_2$*), 0.96-0.91 (2H, m, C*H$_2$*); **^{13}C NMR (100 MHz, CDCl$_3$)** δ 151.5 (Q), 136.9 (Q), 135.3 (Q), 134.9, 129.3, 123.7, 43.3, 31.4, 20.9, 5.4; **IR ν_{max}/cm^{-1} (neat)** 2944, 2787, 1494, 1309, 1290, 1134, 1040; **HRMS (ESI)** found *m/z* 240.1060 [M+H]$^+$, $C_{12}H_{18}NO_2S$ requires *m/z* 240.1058

2-Cyclobutylmethanesulfonyl-*N*,*N*,4-trimethylaniline (36)

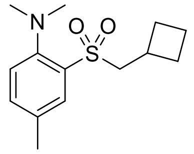

Prepared according to general procedure B. Following column chromatography on silica (0-20% ethyl acetate in petroleum ether), the material was redissolved in acetonitrile (1 mL) to which was added 2 M NaOH solution (1 mL). The mixture was stirred at room temperature for 3 h before extracting with ethyl acetate (3 x 1 mL). The combined organic extracts were dried ($MgSO_4$), filtered and the volatiles were removed under reduced pressure to afford the titled sulfone as a colourless oil (22 mg, 0.08 mmol, 41%); $\boldsymbol{R}_f$ 0.41 (20% ethyl acetate in petroleum ether); **^{1}H NMR (400 MHz, CDCl$_3$)** δ 7.81 (1H, d, *J* 1.9, Ar*H*), 7.38 (1H, dd, *J* 8.1, 1.9, Ar*H*), 7.29 (1H, d, *J* 8.1, Ar*H*), 3.62 (2H, d, *J* 7.3, SO_2CH_2), 2.75 (6H, s, $N(CH_3)_2$), 2.68-2.57 (1H, m, C*H*), 2.37 (3H, s, ArCH_3), 2.0-1.67 (6H, m, CH_2); **^{13}C NMR (100 MHz, CDCl$_3$)** δ 151.5 (Q), 135.2 (Q), 135.1 (Q), 135.0, 130.5, 123.1, 59.5, 46.3, 30.0, 28.4, 20.8, 19.2; **IR ν_{max}/cm^{-1} (neat)** 2980, 1493, 1383, 1300, 1133, 941; **HRMS (ESI)** found *m/z* 268.1375 $[M+H]^+$, $C_{14}H_{22}NO_2S$ requires *m/z* 268.1371.

2-(2-Phenylethanesulfonyl)-*N*,*N*,4-trimethylaniline (37)

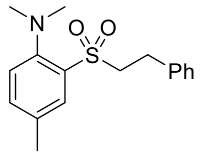

Prepared according to general procedure B. Column chromatography on silica (0-100% ethyl acetate in petroleum ether) afforded the titled sulfone as a colourless amorphous solid (33 mg, 0.11 mmol, 54%); $\boldsymbol{R}_f$ 0.55 (20% ethyl acetate in petroleum ether); **^{1}H NMR (400 MHz, CDCl$_3$)** δ 7.86 (1H, d, *J* 2.1, Ar*H*), 7.39 (1H, dd, *J* 8.2, 2.1 Hz, Ar*H*), 7.32 – 7.22 (3H, m, Ar*H*), 7.20 – 7.15 (1H, m, Ar*H*), 7.11 – 7.07 (2H, m, Ar*H*), 3.92 – 3.74 (2H, m, SO_2CH_2), 2.97 – 2.79 (2H, m, $SO_2CH_2CH_2$), 2.73 (6H, s, $N(CH_3)_2$), 2.38 (3H, s, CH_3); **^{13}C NMR (100 MHz, CDCl$_3$)** δ 151.6 (Q), 138.1 (Q), 135.4, 135.3 (Q), 134.3 (Q), 130.7, 128.7, 128.3, 126.7, 123.2, 55.0 (CH_2), 46.4, 29.2 (CH_2), 20.9; **IR ν_{max}/cm^{-1} (neat)** 2943, 2831, 2789, 1493, 1455, 1302, 1141, 1124, 879, 833, 753, 698; **HRMS (ESI)** found *m/z* 304.1366 $[M+H]^+$, $C_{17}H_{22}NO_2S$ requires *m/z* 304.1366.

2-(3,3,3-Trifluoropropanesulfonyl)-*N*,*N*,4-trimethylaniline (38)

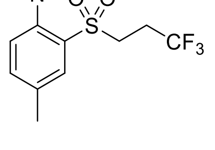

Prepared according to general procedure B. Column chromatography on silica (0-100% ethyl acetate in petroleum ether) afforded the titled sulfone as a yellow oil (26 mg, 0.09 mmol, 45%); $\boldsymbol{R}_f$ 0.36 (20% ethyl acetate in petroleum ether); **^{1}H NMR (400 MHz, CDCl$_3$)** δ 7.82 (1H, d, *J* 1.8, Ar*H*), 7.44 (1H, dd, *J* 8.2 1.8, Ar*H*), 7.34 (1H, d, *J* 8.2, Ar*H*), 3.83-3.77 (4H, m, $SO_2CH_2CH_2CF_3$), 2.75 (6H, s, $N(CH_3)_2$), 2.39 (3H, s, ArCH_3); **^{13}C NMR (100 MHz, CDCl$_3$)** δ 151.7 (Q), 136.0, 135.8 (Q), 133.5 (Q), 130.7, 123.6, 55.5, 47.0 (q, *J* 2.88), 46.4, 28.8 (q, *J* 30.8), 21.0; **IR ν_{max}/cm^{-1} (neat)** 2947, 2836,

2793, 1603, 1495, 1380, 1298, 1268, 1133, 1087; **HRMS (ESI)** found *m/z* 296.09254 $[M+H]^+$, $C_{12}H_{17}F_3NO_2S$ requires *m/z* 296.09266.

2-(2-Propanesulfonyl)-*N*,*N*,4-trimethylaniline (39)

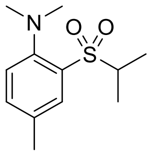

Prepared according to general procedure B. Column chromatography on silica (0-10% ethyl acetate in petroleum ether) afforded the titled sulfone as a colourless oil (13 mg, 0.06 mmol, 27%); $\boldsymbol{R}_f$ 0.20 (10% ethyl acetate in petroleum ether); **^{1}H NMR (400 MHz, CDCl$_3$)** δ 7.83 (1H, d, *J* 1.8, Ar*H*), 7.37 (1H, dd, *J* 8.1, 1.8, Ar*H*), 7.29 (1H, d, *J* 8.1, Ar*H*), 4.15 (1H, sept, *J* 7.6, $SO_2CH(CH_3)_2$), 2.72 (6H, s, $N(CH_3)_2$), 2.37 (3H, s, ArCH_3), 1.24 (6H, d, *J* 7.6, $SO_2CH(CH_3)_2$); **^{13}C NMR (100 MHz, CDCl$_3$)** δ 151.7 (Q), 135.3 (Q), 135.0, 133.6 (Q), 131.3, 123.2, 52.5, 46.3, 20.8, 15.1; **IR ν_{max}/cm^{-1} (neat)** 2941, 2830, 1493, 1456, 1299, 1132, 1054; **HRMS (ESI)** found *m/z* 242.1209 $[M+H]^+$, $C_{12}H_{20}NO_2S$ requires *m/z* 242.1209.

2-(Oxolane-3-sulfonyl)-*N*,*N*,4-trimethylaniline (40)

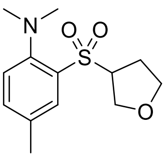

Prepared according to general procedure B. Column chromatography on silica (0-100% ethyl acetate in petroleum ether) afforded the titled sulfone as a colourless liquid (28 mg, 0.11 mmol, 53%); $\boldsymbol{R}_f$ 0.34 (50% ethyl acetate in petroleum ether); **^{1}H NMR (400 MHz, CDCl$_3$)** δ 7.86 (1H, d, *J* 1.8, Ar*H*), 7.40 (1H, dd, *J* 8.1, 1.8, Ar*H*), 7.31 (1H, d, *J* 8.1, Ar*H*), 4.79-4.72 (1H, m, $(CH_2)_2CH$), 4.04-3.81 (4H, m, $(CH_2)_2O$), 2.73 (6H, s, $N(CH_3)_2$), 2.39-2.32 (1H, m, CH_2), 2.38 (3H, m, SO_2CH_3), 2.39-2.32 (1H, m, CH_2); **^{13}C NMR (100 MHz, CDCl$_3$)** δ 151.3 (Q), 135.5, 134.3 (Q), 130.9, 123.4, 68.6, 67.7, 60.9, 46.4, 27.3, 20.8; **IR ν_{max}/cm^{-1} (neat)** 3003, 2938, 2837, 1604, 1521, 1456, 1205, 1151; **HRMS (ESI)** found *m/z* 270.11578 $[M+H]^+$, $C_{13}H_{19}NO_3S$ requires *m/z* 270.11584.

Benzyl 4-[2-(dimethylamino)-5-methylbenzenesulfonyl]piperidine-1-carboxylate (41)

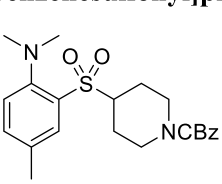

Prepared according to general procedure B. Column chromatography on silica (0-100% ethyl acetate in petroleum ether) afforded the titled sulfone as a yellow, amorphous solid (23 mg, 0.06 mmol, 28%); $\boldsymbol{R}_f$ 0.37 (50% ethyl acetate in petroleum ether); **^{1}H NMR (400 MHz, CDCl$_3$)** δ 7.80 (1H, d *J* 1.8, Ar*H*), 7.41-7.30 (7H, m, Ar*H*), 5.11 (2H, s, ArCH_2), 4.29 (2H, br s, CH_2), 4.14-4.02 (1H, m, C*H*), 2.87-2.77 (2H, m, CH_2), 2.72 (6H, s, $N(CH_3)_2$), 2.37 (3H, s, ArCH_3), 1.84-1.71 (4H, m, -$(CH_2)_2$NCBz); **^{13}C NMR (100 MHz, CDCl$_3$)** δ 155.1 (Q), 151.8 (Q), 136.6 (Q), 135.7, (Q), 135.6, 133.1, (Q), 131.4, 128.7, 128.2, 128.1, 123.4, 100.1, 67.5, 58.7, 46.5, 43.0, 24.6, 21.0; **IR ν_{max}/cm^{-1} (neat)** 3032, 2939, 2862, 2788, 1697, 1561, 1364, 1302, 1134; **HRMS (ESI)** found *m/z* 417.18389 $[M+H]^+$, $C_{22}H_{28}N_2O_4S$ requires *m/z* 417.18425.

2-[(4,4-Difluorocyclohexyl)sulfonyl]-*N*,*N*,4-trimethylaniline (42)

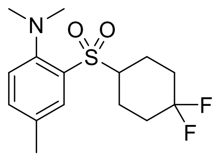

Prepared according to general procedure B. Column chromatography on silica (0-20% ethyl acetate in petroleum ether) afforded the titled sulfone as a pale pink oil (45 mg, 0.14 mmol, 71%); R_f 0.24 (20% ethyl acetate in petroleum ether); **^{1}H NMR (500 MHz, CDCl$_3$)** δ 7.81 (1H, d, *J* 1.7, Ar*H*), 7.40 (1H, dd, *J* 8.3, 1.7, Ar*H*), 7.32 (1H, d, *J* 8.3, Ar*H*), 4.43-3.92 (1H, m, SO_2CH), 2.72 (6H, s, $N(CH_3)_2$), 2.38 (3H, s, ArCH_3), 2.28-2.18 (2H, m, CH_2), 1.96-1.90 (4H, m, CH_2), 1.80-1.63 (2H, m, CH_2); **^{13}C NMR (125 MHz, CDCl$_3$)** δ 151.6 (Q), 135.6 (Q), 135.5, 133.2 (Q), 131.1, 123.3, 122.1 (Q, t, *J* 240.0), 57.8, 46.3, 32.3 (t, *J* 25.9), 21.8 (t, *J* 7.3), 20.8; **^{19}F NMR (470 MHz, CDCl$_3$)** -94.1 (d, *J* 239), -101.1 (d, *J* 239); **IR ν_{max}/cm^{-1} (neat)** 2947, 2831, 1494, 1452, 1261, 1127, 1103; **HRMS (ESI)** found *m/z* 318.1333 $[M+H]^+$, $C_{15}H_{22}NO_2F_2S$ requires *m/z* 318.1334.

2-(4-Methylbenzenesulfonyl)-*N*,*N*,4-trimethylaniline (43)

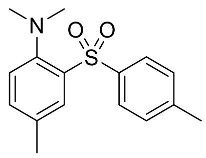

Prepared according to general procedure B. Column chromatography on silica (0-10% ethyl acetate in petroleum ether) afforded the titled sulfone as a colourless oil (31 mg, 0.11 mmol, 54%); R_f 0.31 (20% ethyl acetate in petroleum ether); **^{1}H NMR (400 MHz, CDCl$_3$)** δ 8.06 (1H, d, *J* 2.0, Ar*H*), 7.77 (2H, d, *J* 8.0, Ar*H*), 7.34 (1H, dd, *J* 8.1, 2.0, Ar*H*), 7.22 (2H, d, *J* 8.0, Ar*H*), 7.15 (1H, d, *J* 8.1, Ar*H*), 2.40 (3H, s, ArCH_3), 2.38 (3H, s, ArCH_3), 2.36 (6H, s, $N(CH_3)_2$); **^{13}C NMR (100 MHz, CDCl$_3$)** δ 151.2 (Q), 143.0 (Q), 139.5 (Q), 137.9 (Q), 135.3, 129.7, 128.6, 128.2, 126.8 (Q), 124.2, 45.4, 21.6, 20.9; **IR ν_{max}/cm^{-1} (neat)** 2939, 2786, 1494, 1453, 1309, 1300, 1147, 1092; **HRMS (ESI)** found *m/z* 290.1199 $[M+H]^+$, $C_{16}H_{20}NO_2S$ requires *m/z* 290.1215

2-(4-Methoxybenzenesulfonyl)-*N*,*N*,4-trimethylaniline (44)

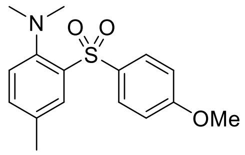

Prepared according to general procedure B. Column chromatography on silica (0-100% ethyl acetate in petroleum ether) afforded the titled sulfone as a colourless liquid (45 mg, 0.15 mmol, 73%); R_f 0.15 (20% ethyl acetate in petroleum ether); **^{1}H NMR (400 MHz, CDCl$_3$)** δ 8.03 (1H, d, *J* 1.6, Ar*H*), 7.84 (2H, d, *J* 9.0, Ar*H*), 7.33 (1H, dd, *J* 8.1, 2.1, Ar*H*), 7.15 (1H, d, *J* 8.1, Ar*H*), 6.90 (2H, d, *J* 9.0, Ar*H*), 3.83 (3H, s, SO_2CH_3), 2.40 (9H, s, $N(CH_3)_2$, ArCH_3); **^{13}C NMR (100 MHz, CDCl$_3$)** δ 162.9 (Q), 151.2 (Q), 138.3 (Q), 135.3 (Q), 135.2, 134.2 (Q), 130.6, 129.7, 124.3, 113.3, 55.7, 45.7, 21.1; **IR ν_{max}/cm^{-1} (neat)** 3024, 2940, 2829, 2786, 1594, 1495, 1293, 1256, 1142, 1092; **HRMS (ESI)** found *m/z* 306.11573 $[M+H]^+$, $C_{16}H_{20}NO_3S$ requires *m/z* 306.11584.

2-(4-Fluorobenzenesulfonyl)-*N*,*N*,4-trimethylaniline (45)

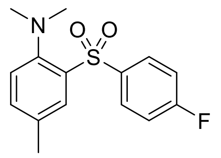

Prepared according to general procedure B. Column chromatography on silica (0-100% ethyl acetate in petroleum ether) afforded the titled sulfone as a colourless liquid (26 mg, 0.09 mmol, 45%); $\boldsymbol{R}_\mathbf{f}$ 0.14 (20% ethyl acetate in petroleum ether); **^{1}H NMR (400 MHz, $CDCl_3$)** δ 7.75 (1H, s, Ar*H*), 7.70-7.67 (2H, m, Ar*H*), 7.21-7.19 (1H, m, Ar*H*), 7.10-7.02 (3H, m, ArH), 2.54 (6H, s, $N(CH_3)_2$), 2.39 (3H, s, $ArCH_3$); **^{13}C NMR (100 MHz, $CDCl_3$)** δ 165.4 (d, *J* 255.5, Q), 151.1 (Q), 138.0 (d, *J* 3.0, Q), 135.6 (Q), 131.0 (d, *J* 9.4), 129.7, 124.3, 115.2 (d, *J* 22.5), 100.0 (Q), 45.4, 20.9; ^{19}F NMR (376.5 MHz, $CDCl_3$) δ 109.6; **IR ν_{max}/cm^{-1} (neat)** 3072, 2939, 2863, 2830, 1590, 1493, 1310, 1287, 1234, 1146, 1090; **HRMS (ESI)** found *m/z* 294.08391 $[M+H]^+$, $C_{15}H_{17}FNO_2S$ requires *m/z* 294.09585.

4-[2-(Dimethylamino)-5-methylbenzenesulfonyl]benzonitrile (46)

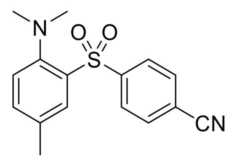

Prepared according to general procedure B. Column chromatography on silica (0-100% ethyl acetate in petroleum ether) afforded the titled sulfone as a yellow, crystalline solid (37 mg, 0.12 mmol, 62%); $\boldsymbol{R}_\mathbf{f}$ 0.21 (20% ethyl acetate in petroleum ether); **mp** 174-175 °C (Petrol ether 40-60 ˚C : Et_2O); **^{1}H NMR (400 MHz, $CDCl_3$)** δ 8.04 (1H, d, *J* 1.7, Ar*H*), 7.97 (2H, d, *J* 8.7, Ar*H*), 7.74 (2H, d, *J* 8.7, Ar*H*), 7.41 (1H, dd, *J* 10.2, 1.7, Ar*H*), 7.17 (1H, d, *J* 10.2, Ar*H*), 3.43 (3H, s, SO_2CH_3), 2.33 (6H, s, $N(CH_3)_2$); **^{13}C NMR (100 MHz, $CDCl_3$)** δ 151.1 (Q), 147.0 (Q), 136.6 (Q), 136.3, 136.0 (Q), 131.9, 130.1, 128.7, 124.5, 116.0 (Q), 100.1 (Q), 45.4, 21.1; **IR ν_{max}/cm^{-1} (neat)** 3093, 2939, 2864, 2831, 2789, 2232, 1454, 1286, 1189, 1148; **HRMS (ESI)** found *m/z* 301.10053 $[M+H]^+$, $C_{16}H_{17}N_2O_2S$ requires *m/z* 301.10053.

N-{[(5-*S*)-3-[3-Fluoro-5-methanesulfonyl-4-(morpholin-4-yl)phenyl]-2-oxo-1,3-oxazolidin-5-yl]methyl}acetamide (47a) and *N*-{[(5-*S*)-3-[5-fluoro-2-methanesulfonyl-4-(morpholin-4-yl)phenyl]-2-oxo-1,3-oxazolidin-5-yl]methyl}acetamide (47b)

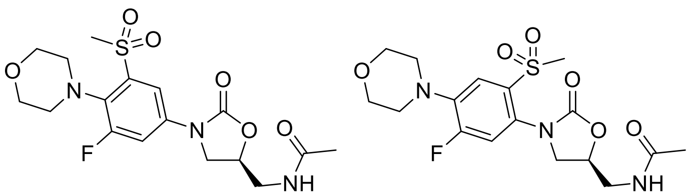

Prepared according to general procedure C. Column chromatography on silica (0-10% methanol in CH_2Cl_2) afforded **47a** as a pale yellow oil (61 mg, 0.15 mmol, 73%) and **47b** as a colourless oil (15 mg, 0.04 mmol, 18%); **47a** $\boldsymbol{R}_\mathbf{f}$ 0.12 (5% methanol in CH_2Cl_2); **^{1}H NMR (500 MHz, $CDCl_3$)** δ 8.00 (1H, dd, *J* 13.9, 2.9, Ar*H*), 7.62 (1H, d, *J* 2.9 Ar*H*), 6.25 (1H, t, *J* 6.2, N*H*), 4.88-4.80 (1H, m, CO_2C^5H), 4.12 (1H, t, *J* 8.9, C^4HH), 3.94-3.89 (2H, m, NCH_2CH_2O), 3.82 (1H, dd, *J* 8.9, 6.3, C^4HH),

3.79-3.69 (3H, m, NCH_2CH_2O and $CHHNHCOCH_3$), 3.64-3.58 (1H, m, $CHHNHCOCH_3$), 3.48-3.41 (2H, m, NCH_2CH_2O), 3.39 (3H, s, SO_2CH_3), 2.92-2.87 (2H, m, NCH_2CH_2O), 2.02 (3H, s, $NHCOCH_3$); **^{13}C NMR (125 MHz, $CDCl_3$)** δ 170.9 (Q), 162.8 (Q, d, *J* 256), 153.8 (Q), 141.2 (Q, d, *J* 6.4), 137.1 (Q, d, *J* 10.4), 133.1 (Q, d, *J* 13.3), 113.1 (d, *J* 1.9), 112.5 (d, *J* 27.0), 72.0, 67.3, 51.5, 47.7, 43.2, 42.0, 23.2; **IR ν_{max}/cm^{-1} (neat)** 3310, 2963, 2858, 1756, 1671, 1477, 1301, 1243, 1136; **HRMS (ESI)** found *m/z* 416.1285 $[M+H]^+$, $C_{17}H_{23}FN_3O_6S$ requires *m/z* 416.1286. **47b** ***R*$_f$** 0.23 (5% methanol in CH_2Cl_2); **^{1}H NMR (500 MHz, $CDCl_3$)** δ 7.56 (1H, d, *J* 9.0, Ar*H*), 7.07 (1H, d, *J* 12.7 Ar*H*), 6.18-2.89 (1H, m, N*H*), 4.93-4.87 (1H, m, CO_2C^5H), 4.04-3.93 (1H, m, C^4HH), 3.89-3.86 (4H, m, NCH_2CH_2O), 3.77-3.62 (3H, m, C^4HH and $CH_2NHCOCH_3$), 3.21 (3H, s, SO_2CH_3), 3.20-3.17 (4H, m, NCH_2CH_2O), 2.06 (3H, s, $NHCOCH_3$); **^{13}C NMR (125 MHz, $CDCl_3$)** δ 171.1 (Q), 157.6 (Q, d, *J* 259), 156.3 (Q), 141.2 (Q, d, *J* 8.1), 129.4 (Q, d, *J* 10.4), 129.3 (Q, d, *J* 10.2), 120.7 (Q, d, *J* 5.4), 118.7 (d, *J* 20.4), 66.6, 50.2, 50.1, 44.0, 41.7, 23.2, one C not found; **IR ν_{max}/cm^{-1} (neat)** 3315, 2964, 2856, 1750, 1671, 1505, 1541, 1309, 1237, 1150, 1117; **HRMS (ESI)** found *m/z* 438.1103 $[M+Na]^+$, $C_{17}H_{22}FNaN_3O_6S$ requires *m/z* 438.1106.

[1-(3-Methanesulfonyl-10*H*-phenothiazin-10-yl)propan-2-yl]dimethylamine (48)

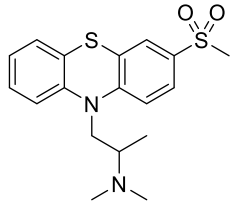

Prepared according to general procedure C. Column chromatography on silica (0-20% methanol in dichloromethane) afforded the titled sulfone as a red, amorphous solid (67 mg, 0.19 mmol, 92%); ***R*$_f$** 0.12 (5% methanol in dichloromethane); **^{1}H NMR (400 MHz, $CDCl_3$)** δ 7.76-7.70 (1H, m, Ar*H*), 7.70-7.67 (1H, m, Ar*H*), 7.27-7.21 (1H, m, Ar*H*), 7.20-7.11 (2H, m, Ar*H*), 7.09-7.01 (2H, m, Ar*H*), 4.43 (1H, dd, *J* 13.6, 3.7, C*H*H), 3.82 (1H, dd, *J* 13.6, 8.9, CH*H*), 3.31-3.23 (1H, m, $CHCH_3$), 3.03 (3H, s, SO_2CH_3), 2.51 (6H, s, $N(CH_3)_2$) 1.21 (3H, d, *J* 6.6, $CHCH_3$); **^{13}C NMR (100 MHz, $CDCl_3$)** δ 150.0 (Q), 143.4 (Q), 134.3 (Q), 128.1, 128.0, 127.4 (Q), 127.3, 127.2, 126.6, 124.8 (Q), 124.3, 116.7, 115.9, 56.8, 50.1, 44.7, 40.3, 11.7; **IR ν_{max}/cm^{-1} (neat)** 2972, 1458, 1309, 1147, 958, 753, 730; **HRMS (ESI)** found *m/z* 363.1195 $[M+H]^+$, $C_{18}H_{23}N_2O_2S_2$ requires *m/z* 363.1196.

10,11-Dihydro-4-methanesulfonyl-5*H*-dibenz[*b,f*]azepine (49a) and 10,11-Dihydro-2-methanesulfonyl-5*H*-dibenz[*b,f*]azepine (49b)

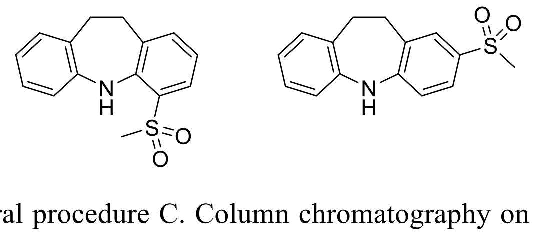

Prepared according to general procedure C. Column chromatography on silica (0-100% ethyl acetate in petroleum ether) afforded **49a** as a colourless film (9 mg, 0.03 mmol, 16%) and **49b** as a yellow, crystalline solid (18 mg, 0.07 mmol, 33%); **49a** ***R*$_f$** 0.64 (40% ethyl acetate in petroleum ether); **^{1}H NMR (400 MHz, $CDCl_3$)** δ 8.67 (1H, broad s, N*H*), 7.77 (1H, dd, *J* 8.0, 1.6, Ar*H*), 7.32-7.28 (1H, m, Ar*H*), 7.16-7.11 (1H, m, Ar*H*), 7.09-7.05 (1H, m, Ar*H*), 6.92-6.87 (1H, m, Ar*H*), 6.87-6.81 (1H, m, Ar*H*), 3.17-3.08 (4H, m, CH_2), 3.08 (3H, s, SO_2CH_3); **^{13}C NMR (125 MHz, $CDCl_3$)** δ 142.1 (Q), 140.8 (Q), 136.2, 133.5 (Q), 130.9, 128.0, 127.9 (Q), 127.2, 125.2 (Q), 120.4, 119.3, 119.2, 43.7,

35.1, 34.8; **IR ν_{max}/cm^{-1} (neat)** 3343, 1585, 1464, 1106, 961, 761, 734; **HRMS (ESI)** found *m/z* 274.0897 $[M+H]^+$, $C_{15}H_{16}NO_2S$ requires *m/z* 274.0896. **49b *R*$_f$** 0.25 (40% ethyl acetate in petroleum ether); **mp** 183-185 °C (CH_2Cl_2); **^{1}H NMR (400 MHz, CDCl$_3$)** δ 7.62-7.57 (2H, m, Ar*H*), 7.16-7.11 (1H, m, Ar*H*), 7.11-7.06 (1H, m, Ar*H*), 6.91-6.85 (1H, m, Ar*H*), 6.85-6.80 (2H, m, Ar*H*), 6.51 (1H, broad s, N*H*), 3.14-3.06 (4H, m, C*H*$_2$), 3.04 (3H, s, SO_2CH_3); **^{13}C NMR (100 MHz, CDCl$_3$)** δ 146.9 (Q), 140.6 (Q), 130.6, 130.5, 129.7 (Q), 129.2 (Q), 127.5 (Q), 127.1, 126.4, 121.2, 118.7, 117.9, 45.0, 35.4, 34.5; **IR ν_{max}/cm^{-1} (neat)** 3346, 2981, 1584, 1532, 1304, 1150, 1125, 1093, 969, 753; **HRMS (ESI)** found *m/z* 274.0897 $[M+H]^+$, $C_{15}H_{16}NO_2S$ requires *m/z* 274.0896.

10 mmol Scale Reaction

2-Methanesulfonyl-*N*,*N*-4-trimethylaniline (1)

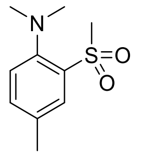

To a round-bottomed flask charged with acridinium **6** (64 mg, 0.10 mmol, 1 mol %), potassium persulfate (8.11 g, 30.0 mmol, 3 equiv), tetrabutylammonium hydrogensulfate (0.679 g, 2.00 mmol, 0.20 equiv) and sodium methanesulfinate (5.10 g, 50.0 mmol, 5 equiv) was added a solution of *N*,*N*-4-trimethylaniline (1.35 g, 10.0 mmol) in degassed 10:1 MeCN/H_2O (100 mL, 0.1 M) under an argon atmosphere. The mixture was stirred for 40 h under irradiation with a 40 W blue LED. The mixture was neutralised with saturated sodium bicarbonate (100 mL) followed by extraction with EtOAc (3 x 100 mL). The combined organic extracts were dried ($MgSO_4$), filtered and the volatiles were removed under reduced pressure. Column chromatography on silica (0-60% ethyl acetate in petroleum ether) afforded the titled sulfone as an off-white, crystalline solid (1.548 g, 7.26 mmol, 73%).

Derivatization Reactions

Potassium 2-(dimethylamino)-5-methylbenzene-1-sulfinate (50)

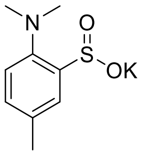

Prepared according to a modified literature procedure.[6] Sulfone **1** (320 mg, 1.50 mmol) in dry diethyl ether (5 mL) was added to a suspension of potassium *tert*-butoxide (673 mg, 6.0 mmol) in dry diethyl ether (10 mL) at room temperature. This was followed by the dropwise addition of benzyl bromide (0.21 mL, 1.80 mmol). After stirring for 16 h the mixture was diluted with water (10 mL) and the phases were separated. The aqueous phase was further washed with diethyl ether (2 x 10 mL) before being acidified with 3 M HCl solution and subsequently neutralized with solid K_2CO_3. The volatiles were removed *in vacuo* and the resultant solid was washed repeatedly with ethanol. The ethanol was removed under reduced pressure to yield the titled sulfinate as a colourless, amorphous solid (220 mg, 0.93 mmol, 62%). **^{1}H NMR (400 MHz, CD$_3$OD)** δ 7.57 (1H, d, *J* 2.2, Ar*H*), 7.01 (1H, dd, *J* 8.1, 2.2,

Ar*H*), 6.90 (1H, d, *J* 8.1, Ar*H*), 2.67 (6H, s, N(CH_3)$_2$), 2.22 (3H, s, ArCH_3); **^{13}C NMR (100 MHz, CD_3OD)** δ 151.1 (Q), 150.4 (Q), 134.3 (Q), 131.9, 124.3, 120.0, 46.4, 20.9; **IR ν_{max}/cm^{-1} (neat)** 3373, 2981, 1486, 1030, 968, 814; **HRMS (ESI)** found *m/z* 283.0300 $[M+H]^+$, $C_9H_{13}KNO_2S$ requires *m/z* 283.0299.

2-(1H-Indol-3-ylsulfanyl)-*N*,*N*,4-trimethylaniline (51)

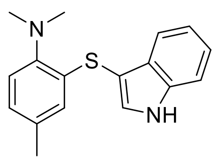

Prepared according to a modified literature procedure.[7] Iodine (53 mg, 0.21 mmol) was added to a solution of **50** (59 mg, 0.25 mmol), indole (25 mg, 0.21 mmol) and triphenylphosphine (66 mg, 0.21 mmol) in ethanol (0.8 mL). The mixture was stirred at reflux for 2 h after which the mixture was allowed to cool to room temperature. A saturated aqueous $Na_2S_2O_3$ solution (5 mL) was added and the mixture was extracted with ethyl acetate (3 x 5 mL). The combined organic extracts were dried ($MgSO_4$), filtered and the volatiles were removed under reduced pressure. Column chromatography on silica (0-60% ethyl acetate in petroleum ether) afforded the titled sulfide as a colourless oil (49 mg, 0.17 mmol, 83%). ***R*$_f$** 0.32 (20% ethyl acetate in petroleum ether); **^{1}H NMR (400 MHz, $CDCl_3$)** δ 8.49 (1H, broad s, N*H*), 7.60 (1H, d, *J* 8.0, Ar*H*), 7.43 (1H, s, Ar*H*), 7.42 (1H, d, *J* 5.2, Ar*H*), 7.28-7.22 (1H, m, Ar*H*), 7.18-7.12 (1H, m, Ar*H*), 7.01 (1H, d, *J* 8.0, Ar*H*), 6.83 (1H, dd, *J* 8.0, 1.4, Ar*H*), 6.46-6.43 (1H, m, Ar*H*), 2.85 (6H, s, N(CH_3)$_2$), 1.99 (3H, s, ArCH_3); **^{13}C NMR (100 MHz, $CDCl_3$)** δ 147.6 (Q), 136.6 (Q), 135.1 (Q), 133.6 (Q), 131.0, 129.5 (Q), 125.8, 125.6, 122.8, 120.7, 119.8, 119.1, 111.6, 102.2 (Q), 44.8, 20.9; **IR ν_{max}/cm^{-1} (neat)** 3399, 2980, 1485, 1452, 1391, 1158, 943, 738; **HRMS (ESI)** found *m/z* 283.1262 $[M+H]^+$, $C_{17}H_{19}N_2S$ requires *m/z* 283.1264.

2-(Cyclohexanesulfinyl)-*N*,*N*,4-trimethylaniline (52)

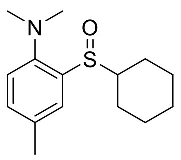

Thionyl chloride (0.05 mL, 0.75 mmol) was added dropwise to a solution of sulfinate **50** (59 mg, 0.25 mmol) in methanol (1.25 mL) at room temperature. After stirring for 3 h a saturated aqueous $NaHCO_3$ solution (5 mL) was added and the mixture was extracted with ethyl acetate (3 x 5 mL). The combined organic phase was dried ($MgSO_4$), filtered and the volatiles were removed under reduced pressure to yield the sulfinate ester as a colourless oil (42 mg). The sulfinate ester was dissolved in dry tetrahydrofuran (0.5 mL) and a 1 M solution of cyclohexylmagnesium chloride in 2-methyltetrahydrofuran (0.3 mL, 0.30 mmol) was added dropwise at room temperature. After stirring for 3 h ethanol (1 mL) was added and the volatiles were removed under reduced pressure. Column chromatography on silica (0-100% ethyl acetate in petroleum ether) afforded the titled sulfoxide as a colourless oil (47 mg, 0.18 mmol, 71%). ***R*$_f$** 0.16 (20% ethyl acetate in petroleum ether); **^{1}H NMR (400 MHz, $CDCl_3$)** δ 7.56 (1H, d, *J* 1.6, Ar*H*), 7.20 (1H, dd, *J* 8.0, 1.6, Ar*H*), 7.06 (1H, d, *J* 8.0, Ar*H*), 2.92-2.83 (1H, m, S(O)C*H*) 2.67 (6H, s, N(CH_3)$_2$), 2.37 (3H, s, ArCH_3), 2.06-1.97 (1H, m, CH_2), 1.92-1.85 (1H, m, CH_2), 1.82-1.75 (1H, m, CH_2), 1.72-1.60 (2H, m, CH_2), 1.52-1.40 (1H, m, CH_2), 1.35-1.25 (2H, m, CH_2), 1.23-1.12 (2H, m, CH_2); **^{13}C NMR (100 MHz, $CDCl_3$)** δ 148.9 (Q), 136.2 (Q), 134.3 (Q), 131.8, 126.1, 120.0, 59.9, 44.9, 27.7, 26.1, 25.5, 25.4, 22.3, 20.8; **IR ν_{max}/cm^{-1}**

(neat) 2980, 1493, 1451, 1383, 1154, 1063, 1029, 941; **HRMS (ESI)** found *m/z* 266.1576 $[M+H]^+$, $C_{15}H_{24}NOS$ requires *m/z* 266.1579.

_N,N_,4-Trimethyl-2-(morpholine-4-sulfonyl)aniline (53)

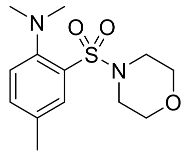

Prepared according to a modified literature procedure.[8] To a dry 2-propanol (1.5 mL) solution of sulfinate **50** (45 mg, 0.19 mmol) was added *N*-chloromorpholine (91 mg, 0.75 mmol). After stirring for 16 h the volatiles were removed under reduced pressure. Column chromatography on silica (0-100% ethyl acetate in petroleum ether) afforded the titled sulfone as a colourless, crystalline solid (38 mg, 0.13 mmol, 70%). $\boldsymbol{R_f}$ 0.23 (20% ethyl acetate in petroleum ether); **mp** 125-127 °C (CH_2Cl_2); **^{1}H NMR (400 MHz, $CDCl_3$)** δ 7.76 (1H, d, *J* 1.6, Ar*H*), 7.33 (1H, dd, *J* 8.1, 1.6, Ar*H*), 7.26 (1H, d, *J* 8.1, Ar*H*), 3.75-3.69 (4H, m, OCH_2), 3.21-3.15 (4H, m, NCH_2), 2.72 (6H, s, $N(CH_3)_2$), 2.35 (3H, s, $ArCH_3$); **^{13}C NMR (100 MHz, $CDCl_3$)** δ 151.4 (Q), 134.5, 134.3 (Q), 133.0 (Q), 132.1, 122.7, 66.9, 46.5, 46.3, 20.7; **IR ν_{max}/cm^{-1} (neat)** 2980, 1383, 1255, 1153, 1074, 954; **HRMS (ESI)** found *m/z* 285.1266 $[M+H]^+$, $C_{13}H_{21}N_2O_3S$ requires *m/z* 285.1267.

2-Methanesulfonyl-_N,N,N_,4-tetramethylanilinium trifluoromethanesulfonate (54)

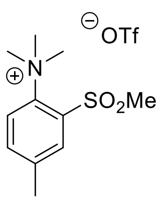

Compound **1** (250 mg, 1.17 mmol) was dissolved in methyl trifluoromethanesulfonate (1.33 mL, 11.7 mmol, 10 eq) and allowed to stir at room temperature for 20 h. The mixture was filtered and the precipitate was washed with dichloromethane to give a beige powder (152 mg). The filtrate was subjected to column chromatography on silica (0-20% methanol in dichloromethane), yielding a beige powder (196 mg) which was combined with the above precipitate to yield the titled compound (348 mg, 0.92 mmol, 79%). $\boldsymbol{R_f}$ 0.14 (10% methanol in dichloromethane); **^{1}H NMR (400 MHz, CD_3OD)** δ 8.35 (1H, d, *J* 1.7, Ar*H*), 8.07 (1H, d, *J* 8.7, Ar*H*), 7.78 (1H, dd, *J* 8.7, 1.7, Ar*H*), 3.99 (9H, s, $N(CH_3)_3$), 3.50 (3H, s, SO_2CH_3), 2.56 (3H, s, $ArCH_3$); **^{13}C NMR (100 MHz, CD_3OD)** δ 144.7 (Q), 142.6 (Q), 137.5 (Q), 137.3, 137.1, 124.0, 59.6, 47.0, 20.6; **IR ν_{max}/cm^{-1} (neat)** 2980, 1319, 1252, 1146, 1026, 632; **HRMS (ESI)** found *m/z* 228.1054 $[M]^+$, $C_{11}H_{18}NO_2S$ requires *m/z* 228.1053.

1-Fluoro-2-methanesulfonyl-4-methylbenzene (55)

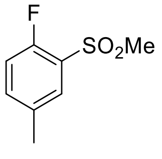

Prepared according to modified literature procedure.[5] A solution of **54** (94 mg, 0.25 mmol) and tetrabutylammonium fluoride (394 mg, 1.25 mmol, 5 eq) in 1-methylpyrrolidinone (0.5 mL, 0.5 M)

was heated at 200 °C for 2 h. The mixture was cooled to room temperature and diluted with brine (3 mL). The mixture was extracted with diethyl ether (3 x 3 mL) and the combined organic extracts were washed with 3 M HCl solution (2 x 10 mL). The organic extract was dried ($MgSO_4$), filtered and the volatiles were removed under reduced pressure to yield the titled compound as a pale yellow oil (28 mg, 0.15 mmol, 60%). $\boldsymbol{R}_f$ 0.39 (40% ethyl acetate in petroleum ether); **^{1}H NMR (400 MHz, $CDCl_3$)** δ 7.77-7.73 (1H, m, Ar*H*), 7.46-7.41 (1H, m, Ar*H*), 7.17-7.11 (1H, m, Ar*H*), 3.22 (3H, s, SO_2CH_3), 2.41 (3H, s, ArCH_3); **^{13}C NMR (100 MHz, $CDCl_3$)** δ 157.5 (d, *J* 252.5, Q), 136.5 (d, *J* 8.0), 134.8 (d, *J* 3.9, Q), 129.5, 127.6 (d, *J* 14.8, Q), 116.8 (d, *J* 21.2), 43.8 (d, *J* 3.2), 20.5; **^{19}F NMR (376.5 MHz, $CDCl_3$)** δ -115.2; **IR ν_{max}/cm^{-1} (neat)** 2980, 1491, 1393, 1310, 1145, 1069, 958, 747; **HRMS (CI)** found *m/z* 189.0389 $[M+H]^+$, $C_8H_{10}FO_2S$ requires *m/z* 189.0380.

1-Ethoxy-2-methanesulfonyl-4-methylbenzene (56)

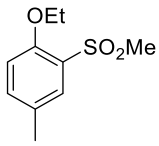

A solution of **55** (28 mg, 0.15 mmol) and sodium ethoxide (51 mg, 0.75 mmol, 5 eq) in ethanol (0.6 mL, 0.25 M) was heated at 78 °C for 48 h. The mixture was cooled to room temperature and diluted with a saturated $NaHCO_3$ solution (3 mL). The mixture was extracted with diethyl ether (3 x 3 mL) and the combined organic extracts were dried ($MgSO_4$), filtered and the volatiles were removed under reduced pressure. Column chromatography on silica (0-100% ethyl acetate in petroleum ether) yielded the titled compound as a yellow oil (32 mg, 0.15 mmol, quant). $\boldsymbol{R}_f$ 0.13 (20% ethyl acetate in petroleum ether); **^{1}H NMR (400 MHz, $CDCl_3$)** δ 7.77 (1H, d, *J* 2.0, Ar*H*), 7.35 (1H, dd, *J* 8.4, 2.0, Ar*H*), 6.93 (1H, d, *J* 8.4, Ar*H*), 4.19 (2H, q, *J* 7.0, CH_2CH_3), 3.23 (3H, s, SO_2CH_3), 2.34 (3H, s, ArCH_3), 1.50 (3H, t, *J* 7.0, CH_2CH_3); **^{13}C NMR (100 MHz, $CDCl_3$)** δ 154.4 (Q), 135.7, 130.2 (Q), 129.5, 128.1 (Q), 113.2, 65.0, 42.9, 20.3, 14.6; **IR ν_{max}/cm^{-1} (neat)** 2929, 1496, 1301, 1142, 907, 727; **HRMS (ESI)** found *m/z* 215.0737 $[M+H]^+$, $C_{10}H_{15}O_3S$ requires *m/z* 215.0736.

***N*-Benzyl-2-methanesulfonyl-4-methylaniline (57)**

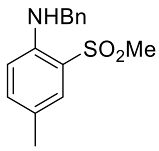

A solution of **55** (28 mg, 0.15 mmol) and benzylamine (50 µL, 0.45 mmol, 3 eq) in dry dimethylsulfoxide (0.6 mL, 0.25 M) was heated at 130 °C for 48 h. The mixture was cooled to room temperature and diluted with a saturated $NaHCO_3$ solution (1 mL) and brine (2 mL). The mixture was extracted with diethyl ether (3 x 3 mL) and the combined organic extracts were dried ($MgSO_4$), filtered and the volatiles were removed under reduced pressure. Column chromatography on silica (0-100% ethyl acetate in petroleum ether) yielded the titled compound as a colourless oil (32 mg, 0.12 mmol, 78%). $\boldsymbol{R}_f$ 0.32 (20% ethyl acetate in petroleum ether); **^{1}H NMR (400 MHz, $CDCl_3$)** δ 7.59 (1H, d, *J* 2.1, Ar*H*), 7.37-7.31 (4H, m, Ar*H*), 7.30-7.25 (1H, m, Ar*H*), 7.19 (1H, dd, *J* 8.4, 2.1, Ar*H*), 6.64 (1H, d, *J* 8.4, Ar*H*), 6.44-6.38 (1H, broad m, N*H*), 4.40 (2H, d, *J* 5.5, CH_2), 3.07 (3H, s, SO_2CH_3), 2.25 (3H, s, ArCH_3); **^{13}C NMR (100 MHz, $CDCl_3$)** δ 144.5 (Q), 138.2 (Q), 136.2, 129.6, 128.8, 127.4, 127.0, 126.2 (Q), 121.5 (Q), 113.0, 47.4, 42.3, 20.0; **IR ν_{max}/cm^{-1} (neat)** 3388, 2922, 1615, 1517, 1287, 1130, 955, 750; **HRMS (ESI)** found *m/z* 276.1053 $[M+H]^+$, $C_{15}H_{18}NO_2S$ requires *m/z* 276.1053.

NMR Spectra of Novel Compounds

400 MHz ^{1}H NMR of **3-methoxy-*N*,*N*,4-trimethylaniline** in $CDCl_3$

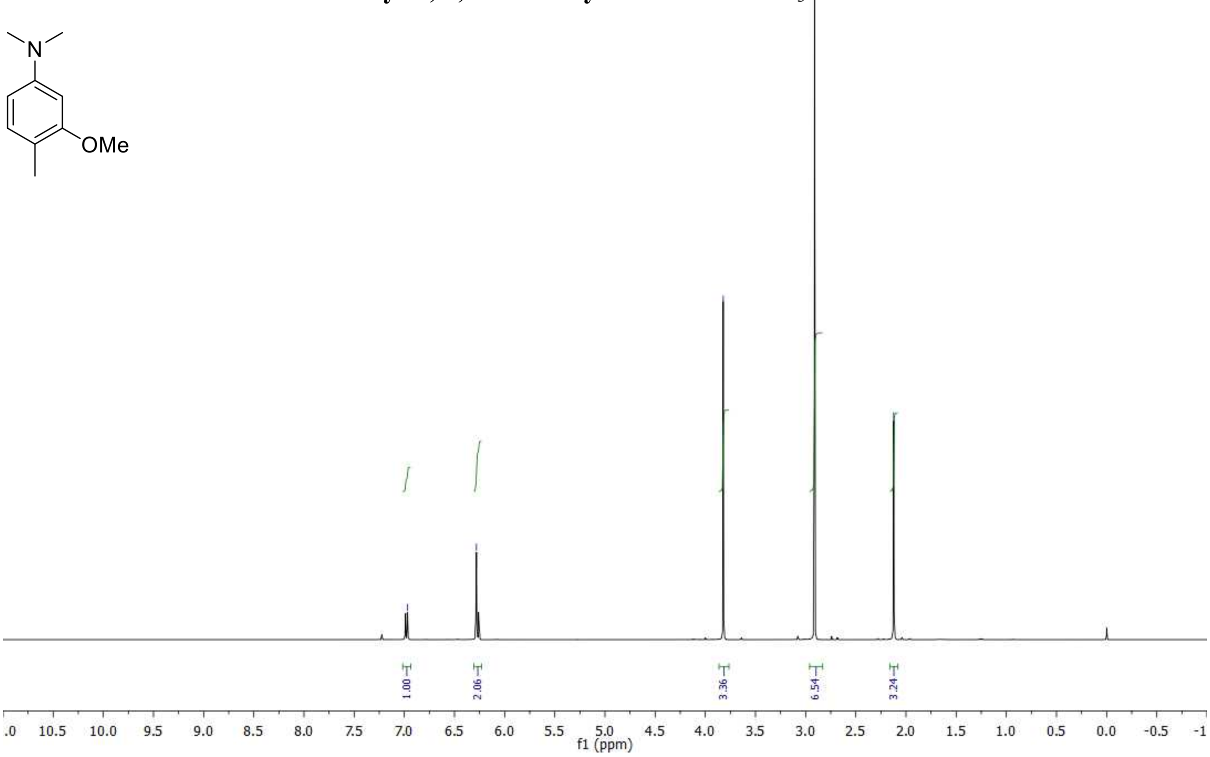

100 MHz ^{13}C NMR of **3-methoxy-*N*,*N*,4-trimethylaniline** in $CDCl_3$

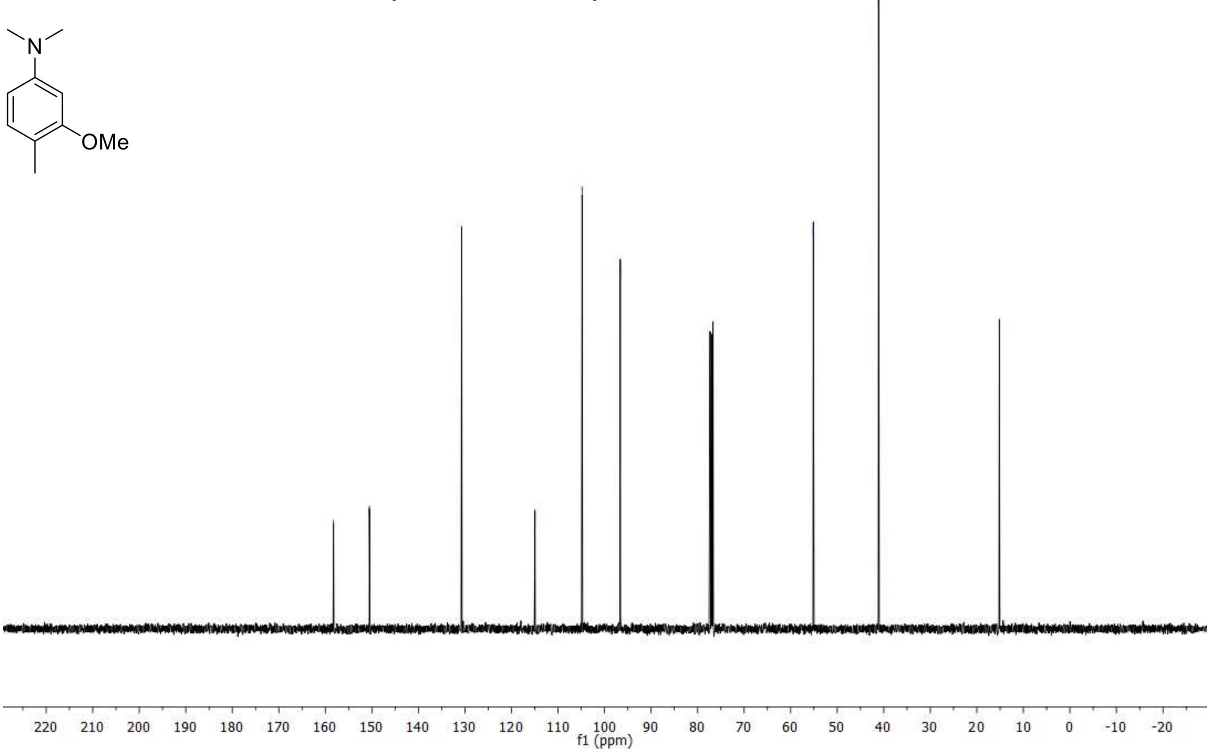

400 MHz ^{1}H NMR of **4-(butan-2-yl)-*N*,*N*-dimethylaniline** in $CDCl_3$

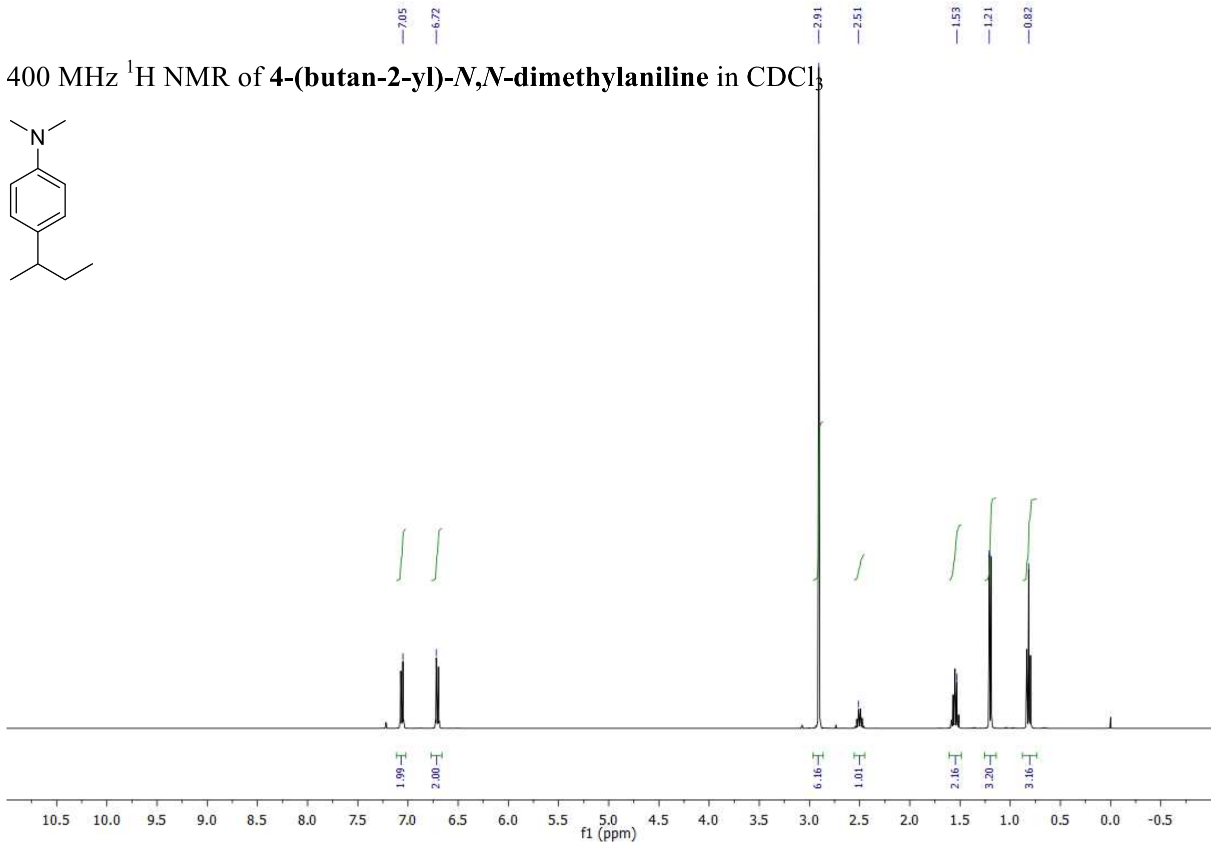

100 MHz ^{13}C NMR of **4-(butan-2-yl)-*N*,*N*-dimethylaniline** in $CDCl_3$

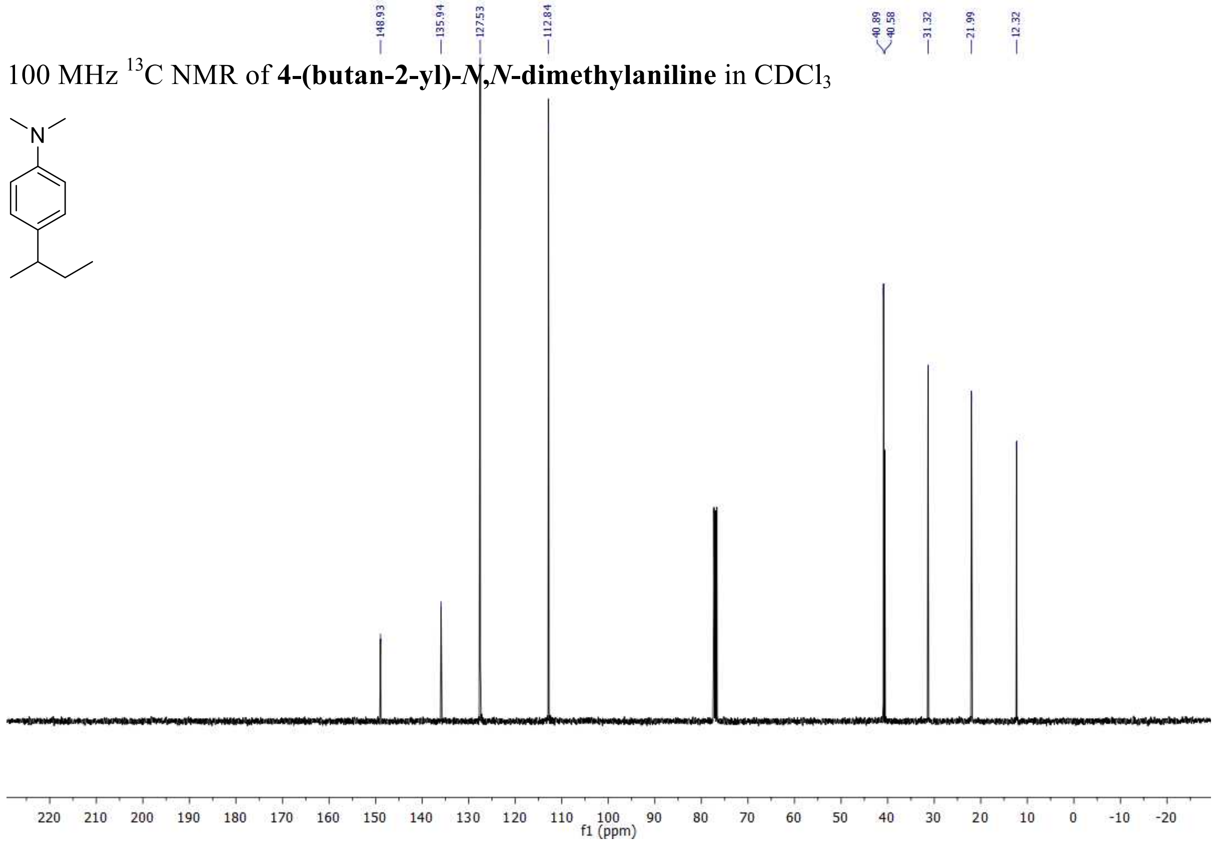

400 MHz ^{1}H NMR of **6-methoxy-*N*,*N*-dimethylpyridin-3-amine** in $CDCl_3$

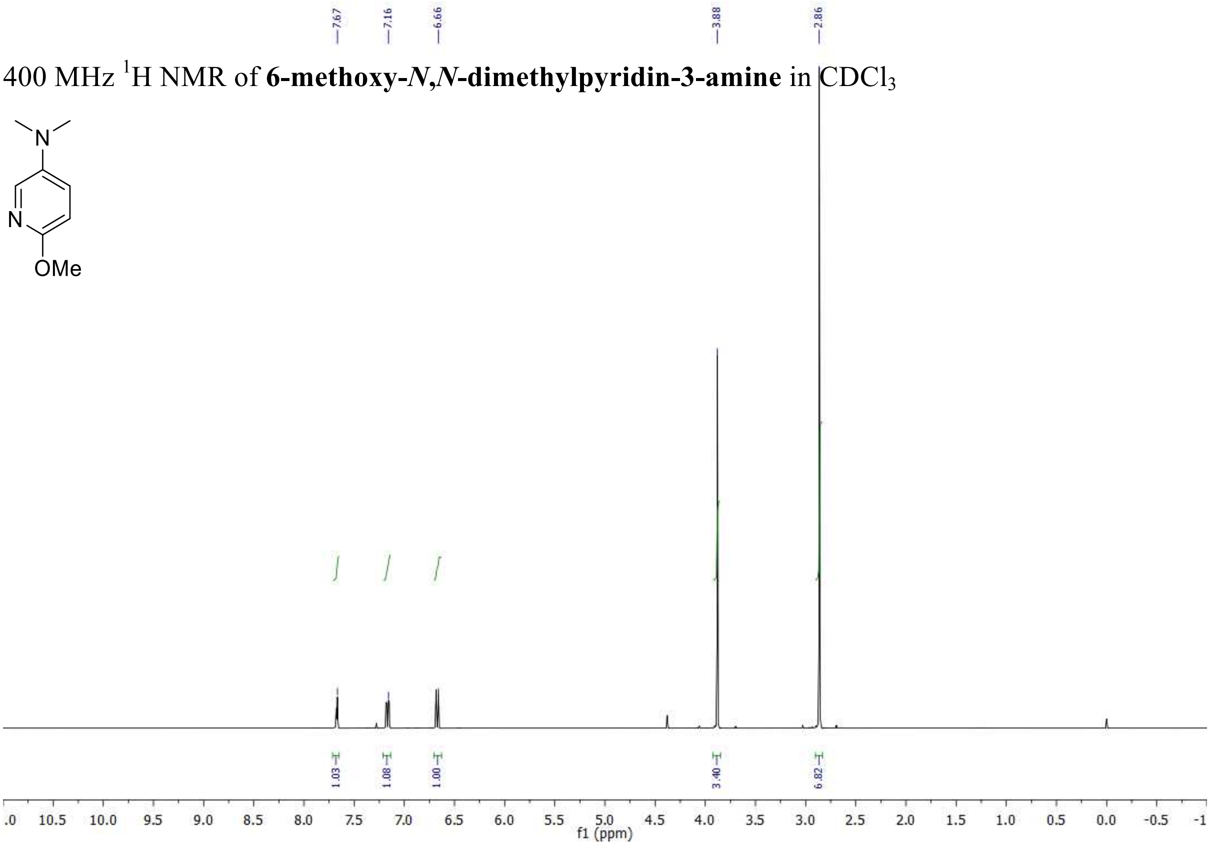

100 MHz ^{13}C NMR of **6-methoxy-*N*,*N*-dimethylpyridin-3-amine** in $CDCl_3$

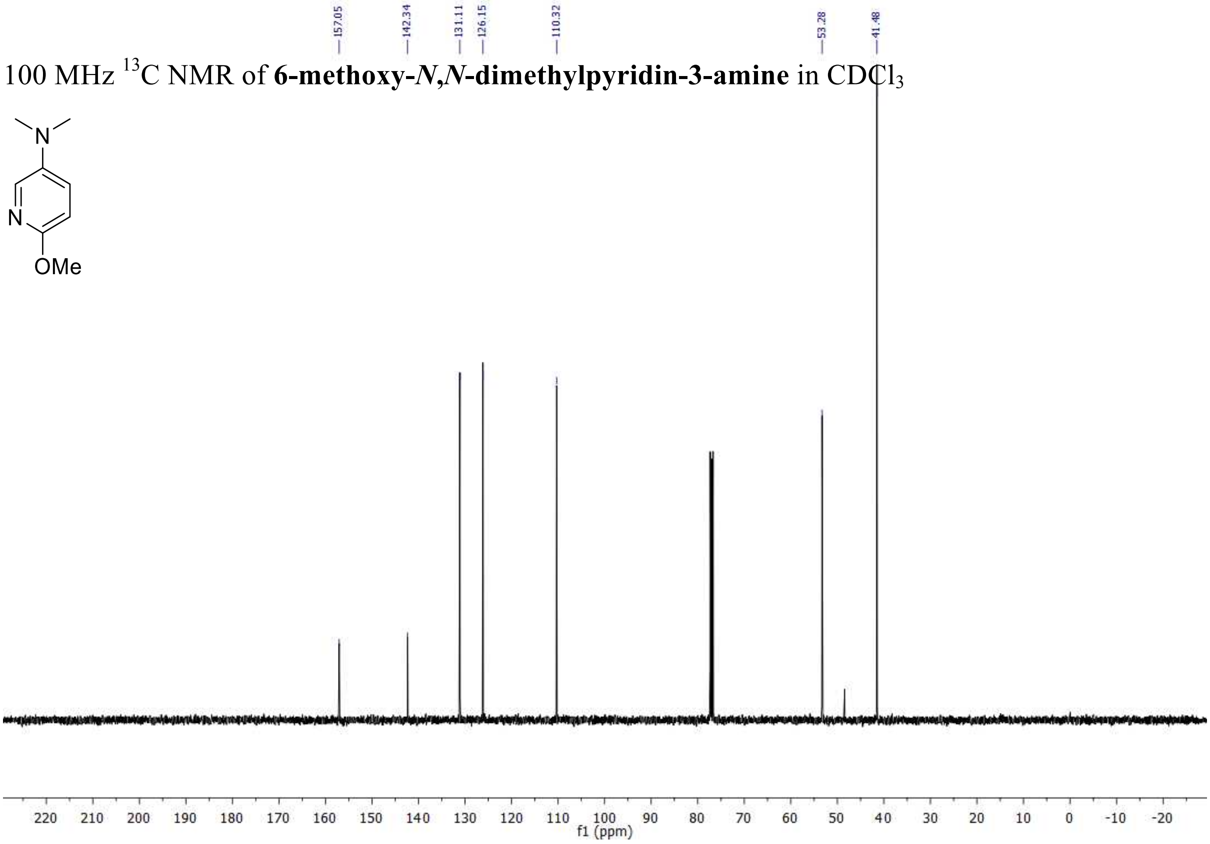

400 MHz ^{1}H NMR of **2-cyclobutylmethanesulfanylpyridine** in $CDCl_3$

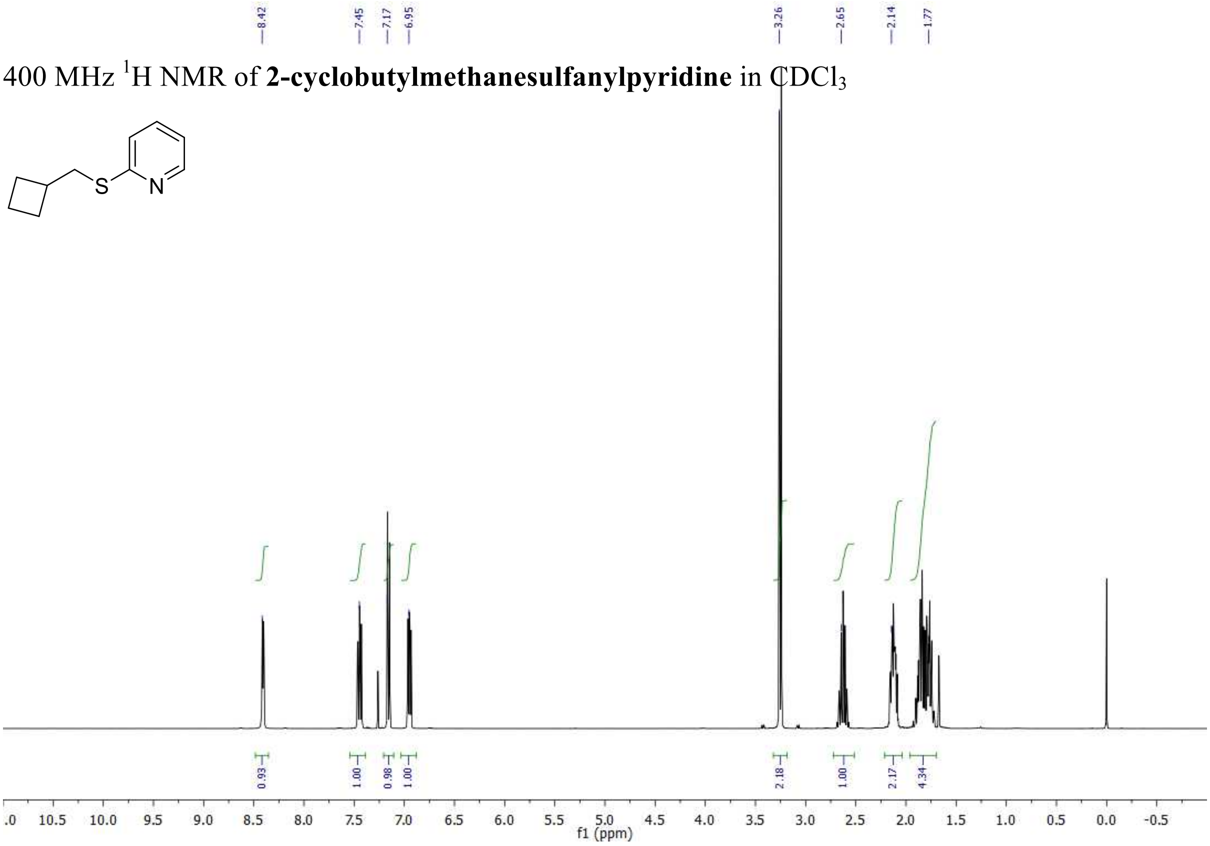

100 MHz ^{13}C NMR of **2-cyclobutylmethanesulfanylpyridine** in $CDCl_3$

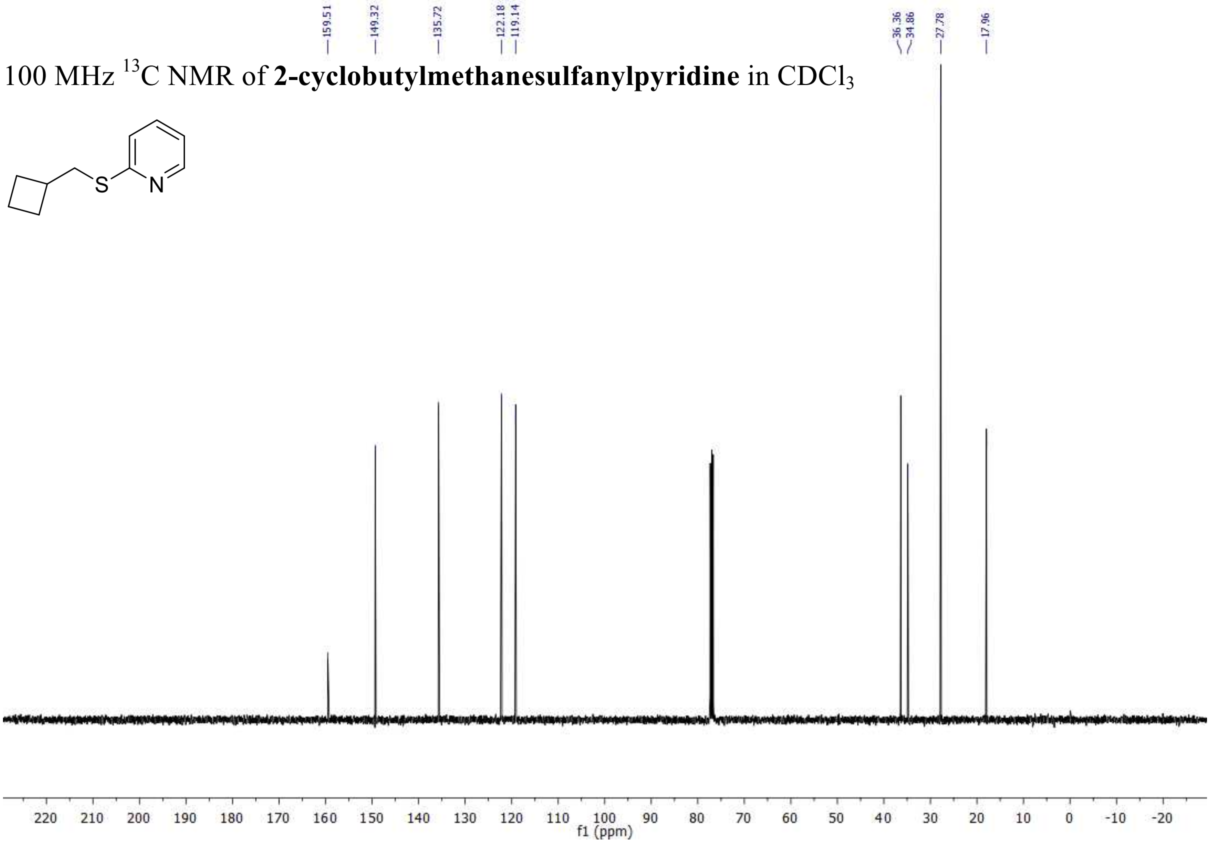

400 MHz ^{1}H NMR of **2-cyclobutylmethanesulfonylpyridine** in $CDCl_3$

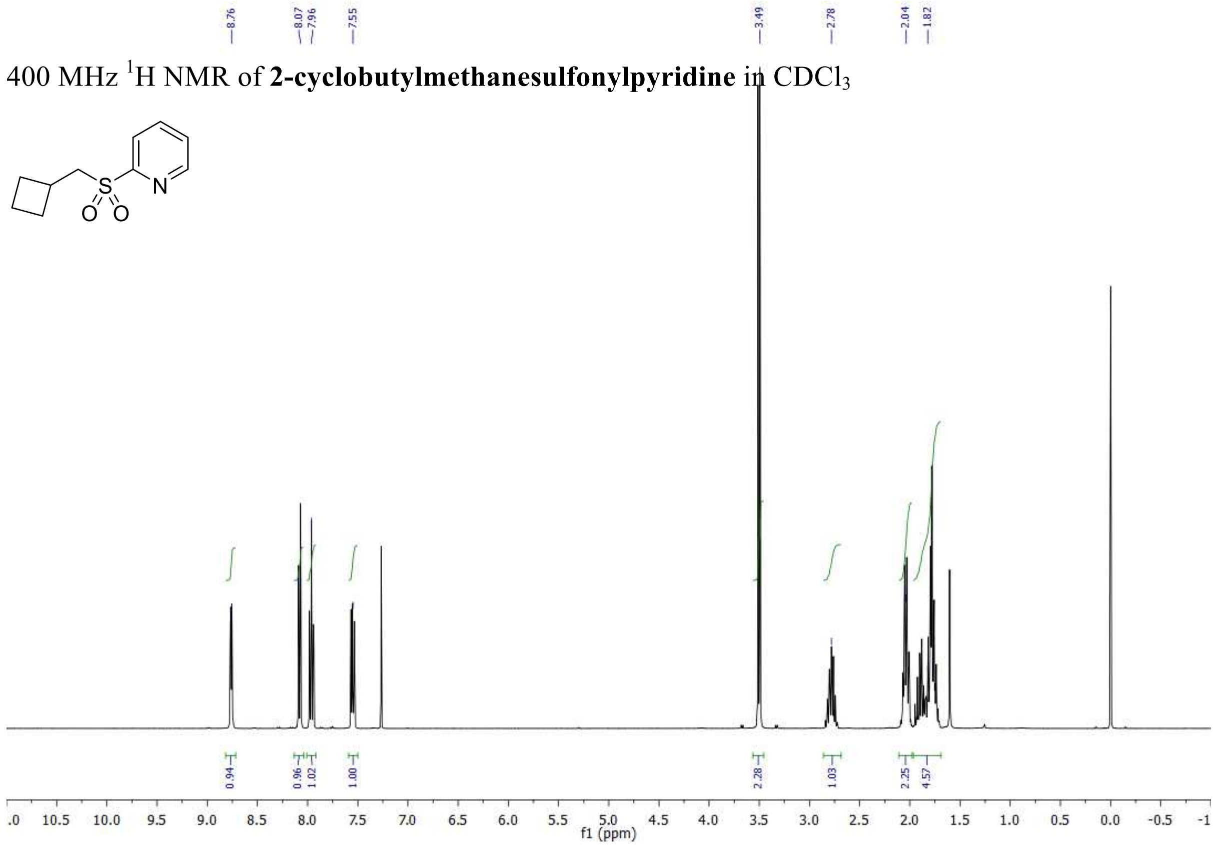

100 MHz ^{13}C NMR of **2-cyclobutylmethanesulfonylpyridine** in $CDCl_3$

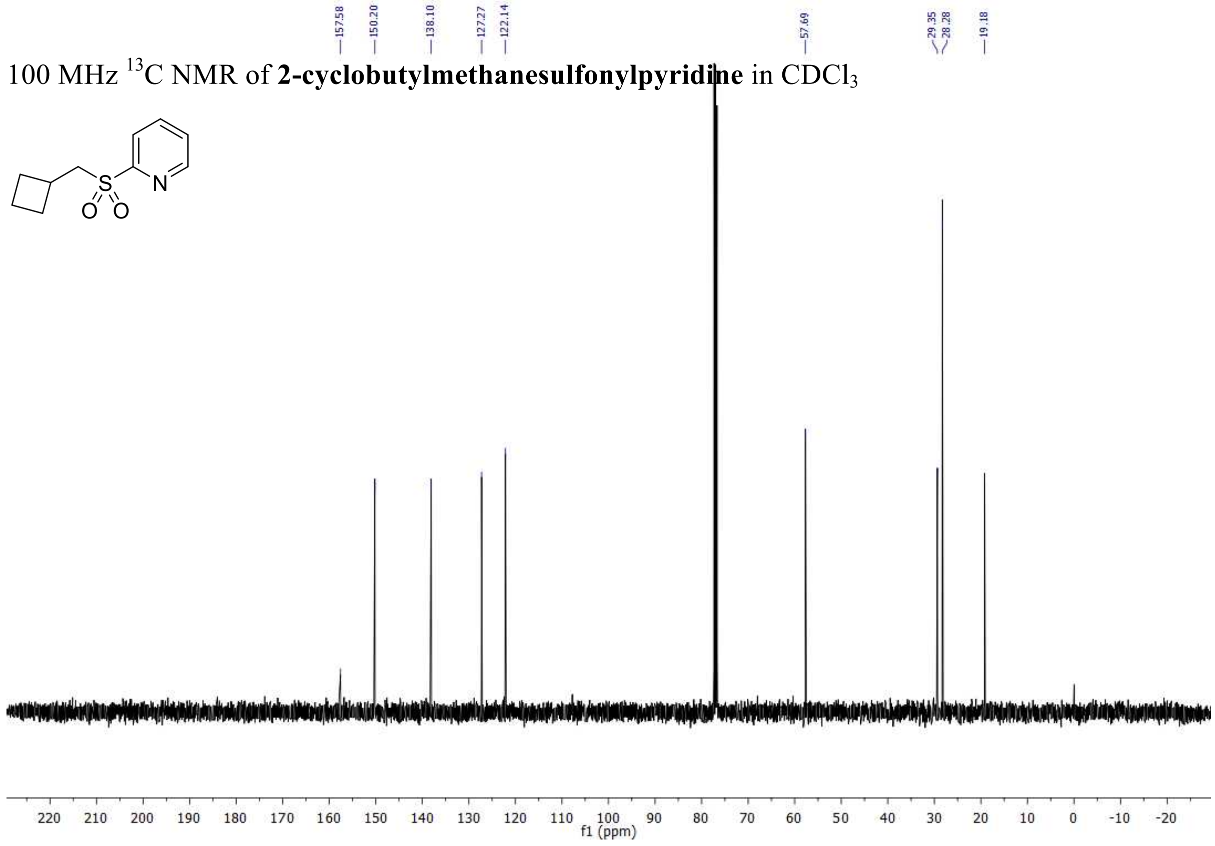

400 MHz ^{1}H NMR of **sodium cyclobutylmethanesulfinate** in CD_3OD

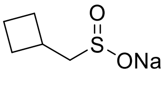

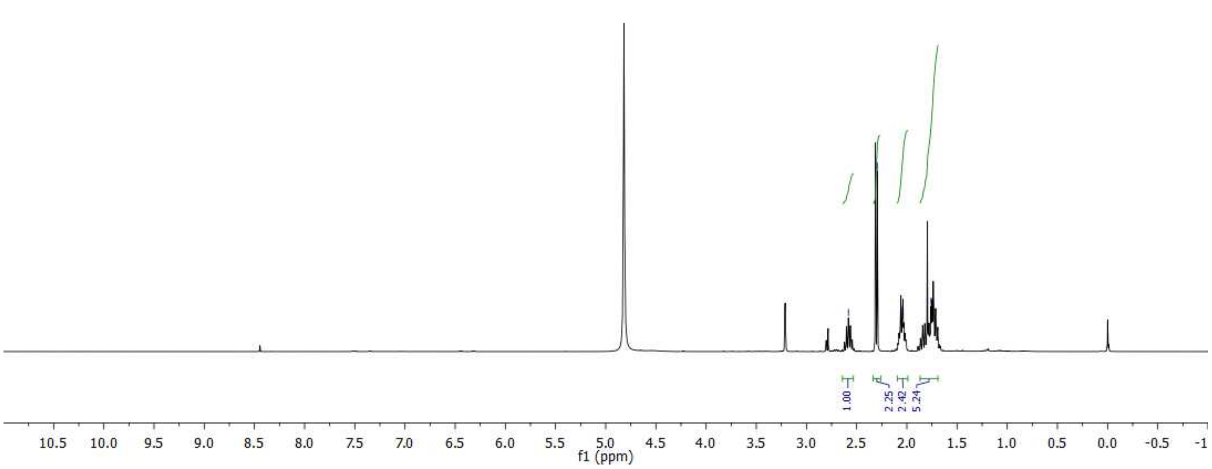

100 MHz ^{13}C NMR of **sodium cyclobutylmethanesulfinate** in CD_3OD

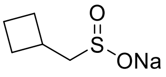

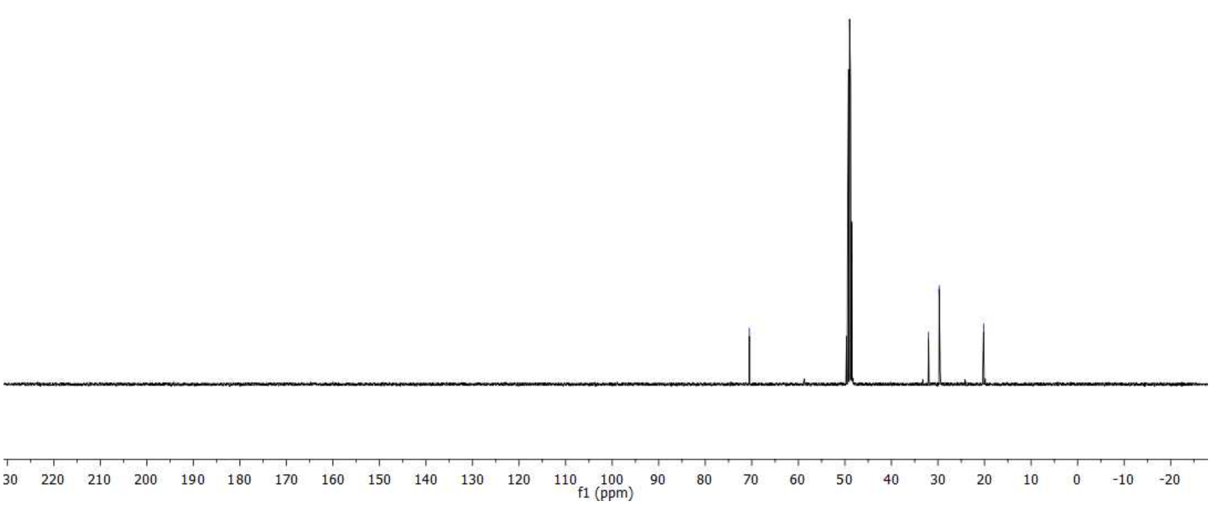

400 MHz ^{1}H NMR of **sodium cyclopropanesulfinate** in CD_3OD

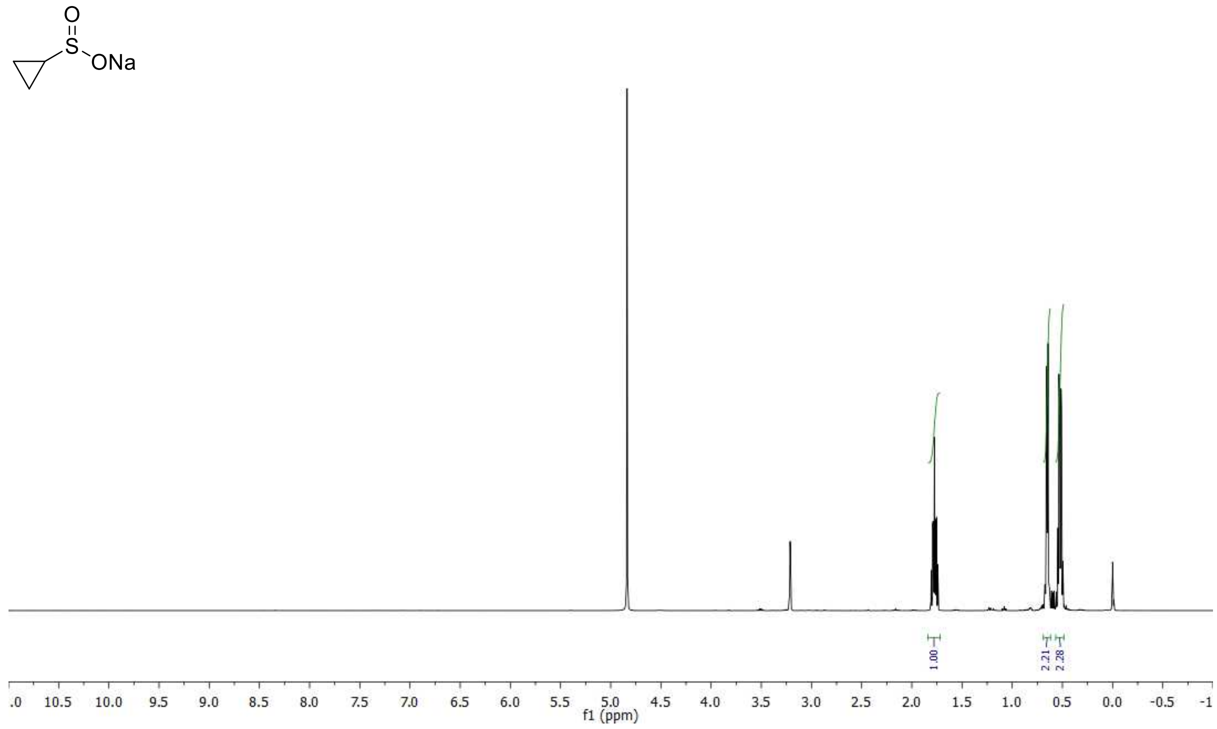

100 MHz ^{13}C NMR of **sodium cyclopropanesulfinate** in CD_3OD

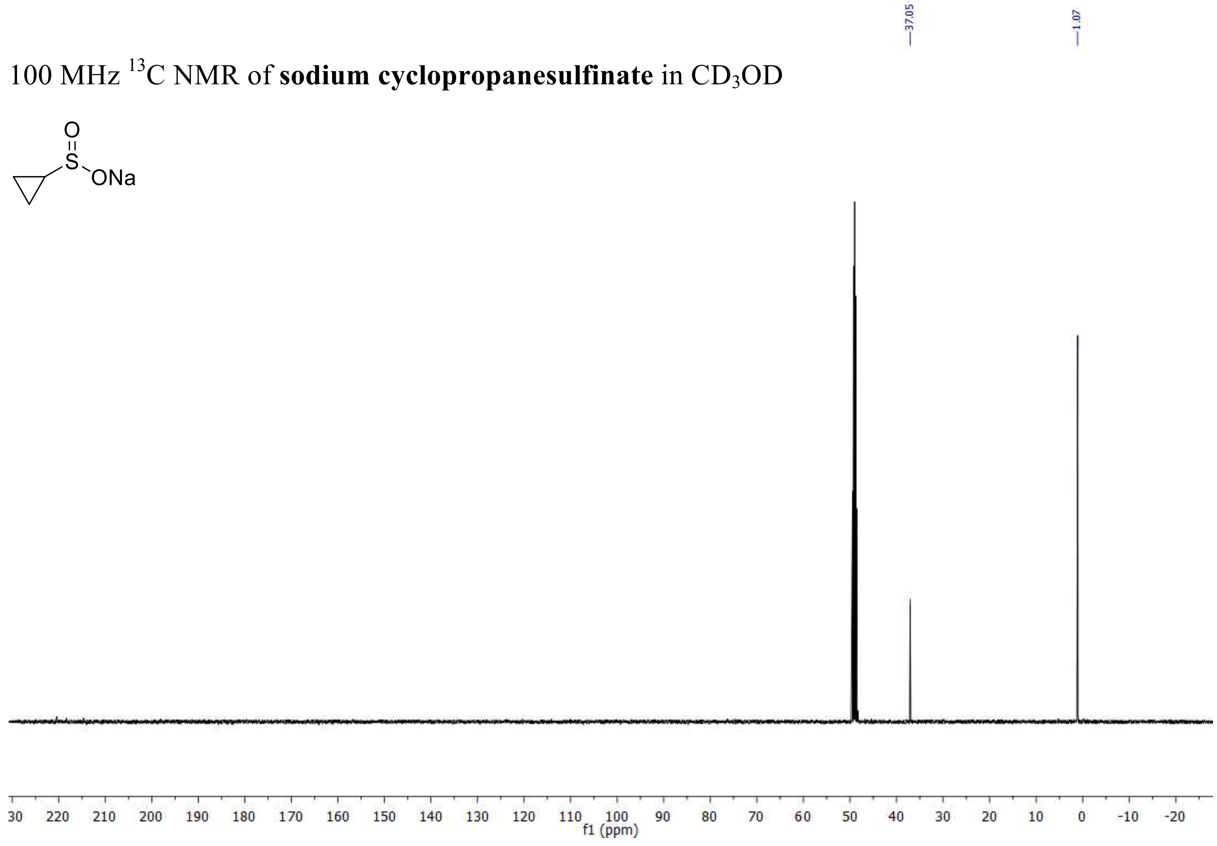

400 MHz ^{1}H NMR of **sodium 4-cyanobenzene-1-sulfinate** in CD_3OD

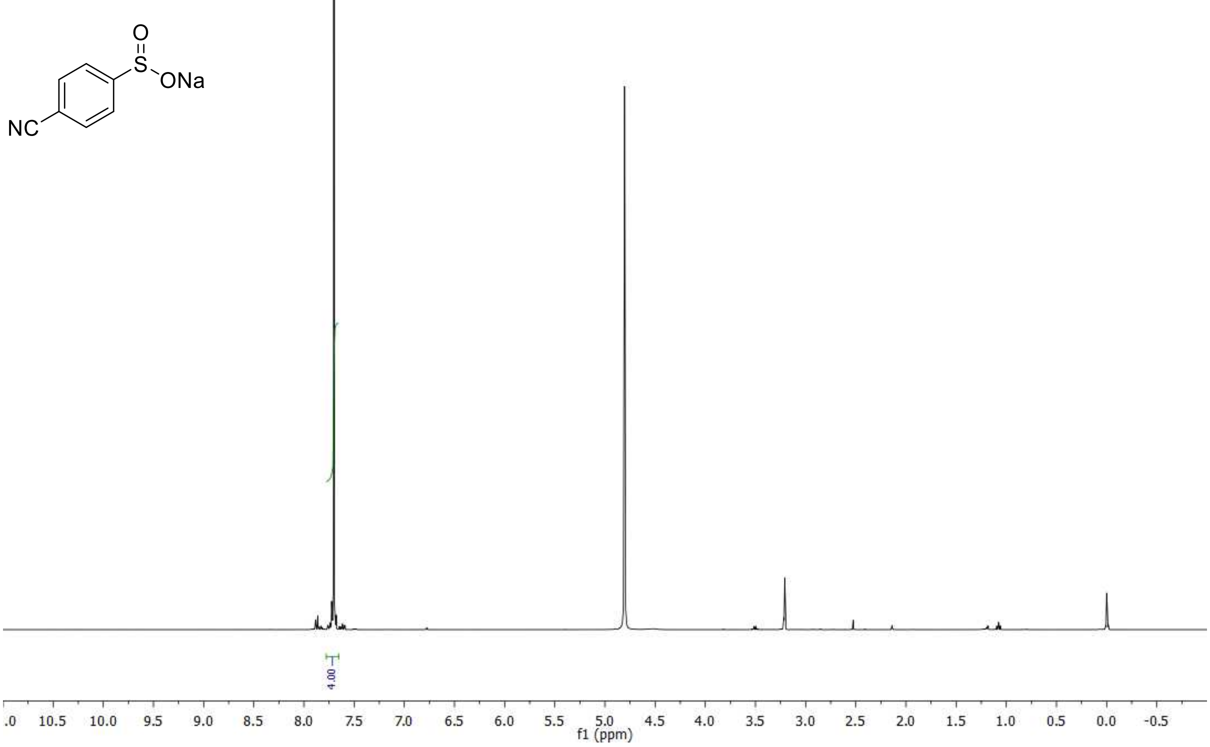

100 MHz ^{13}C NMR of **sodium 4-cyanobenzene-1-sulfinate** in CD_3OD

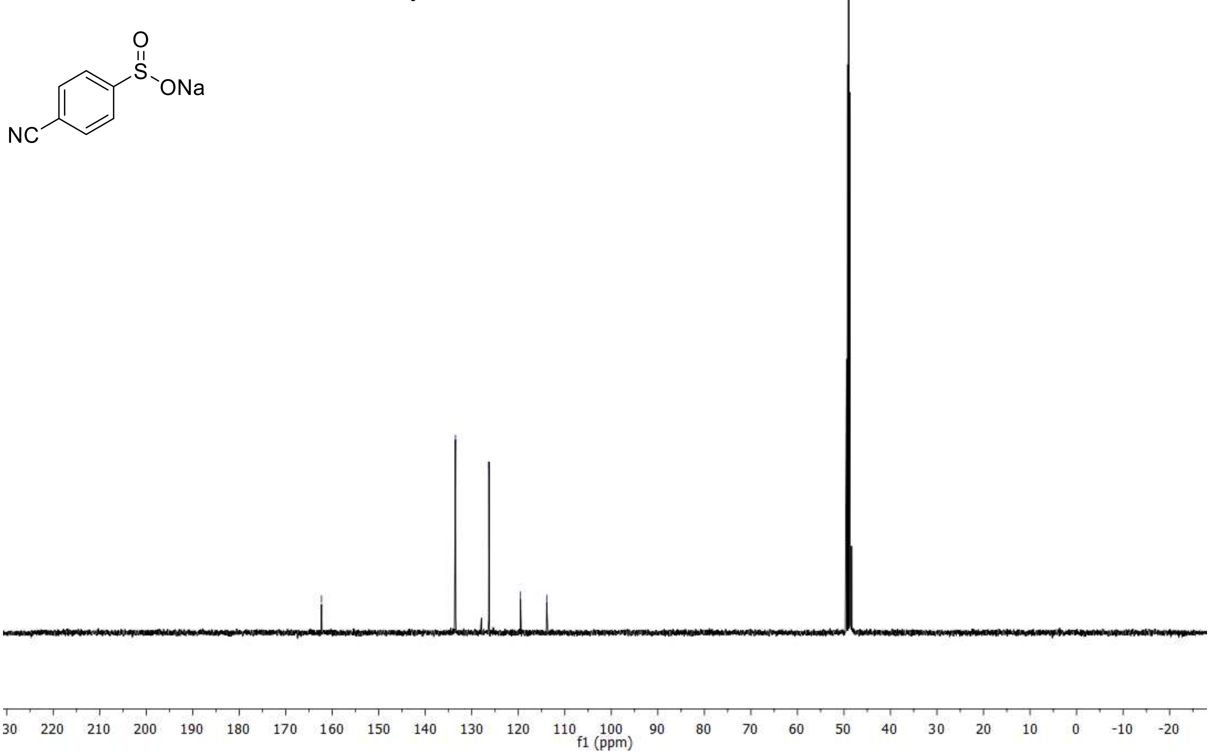

400 MHz ^{1}H NMR of **1** in $CDCl_3$

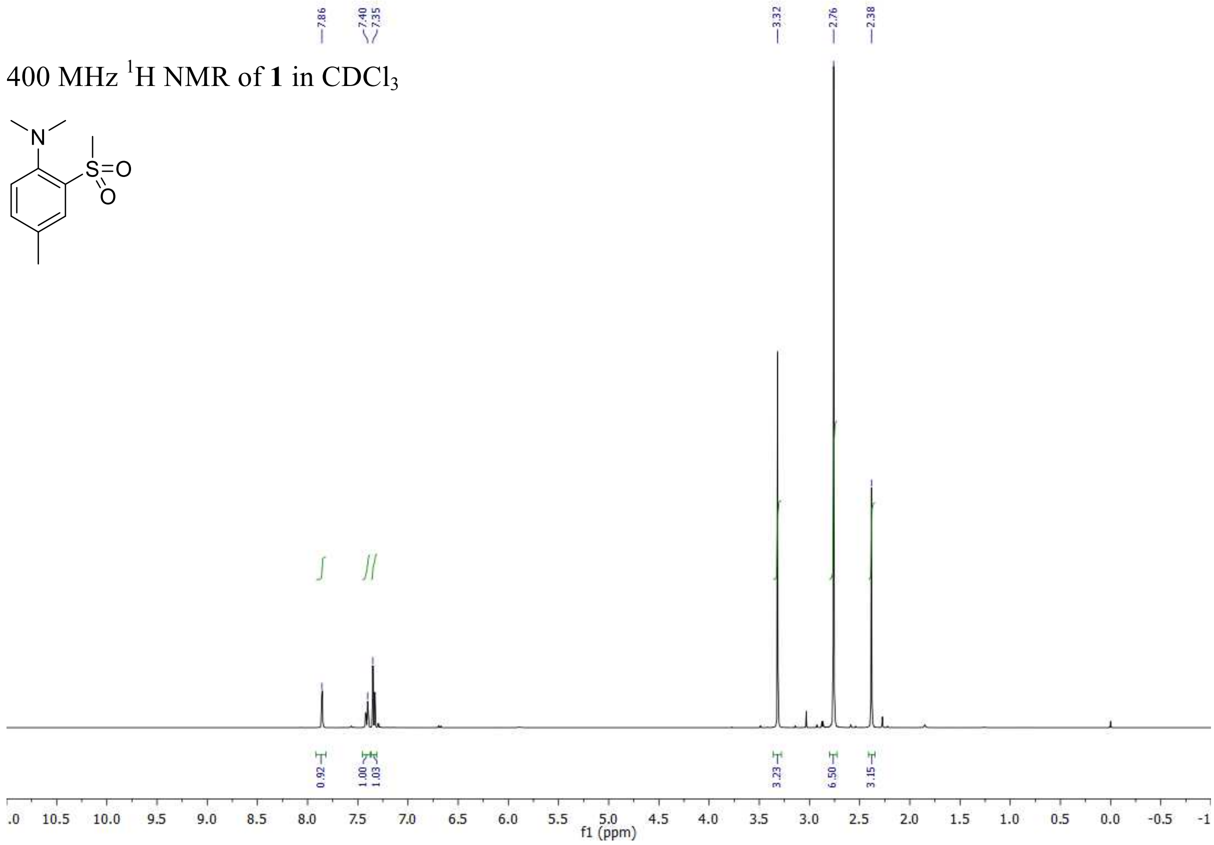

100 MHz ^{13}C NMR of **1** in $CDCl_3$

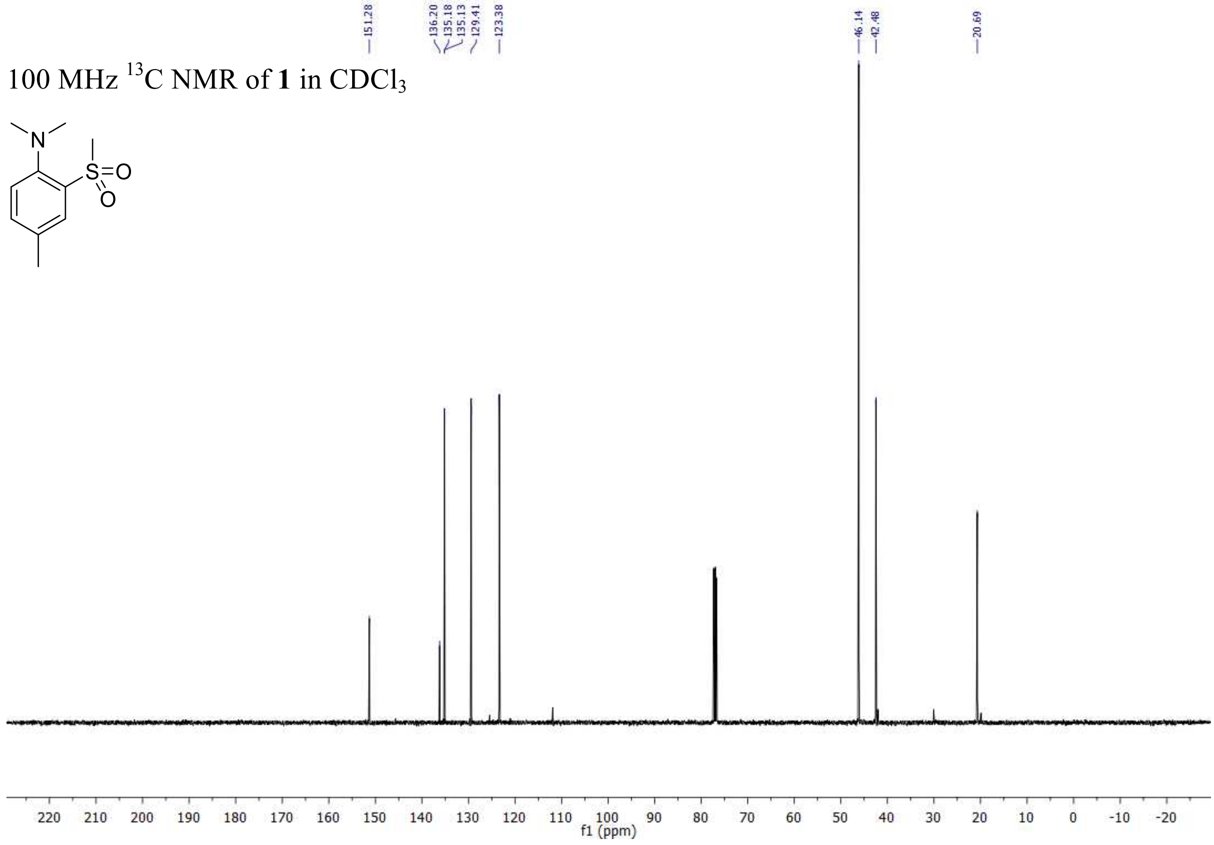

400 MHz ^{1}H NMR of **7** in $CDCl_3$

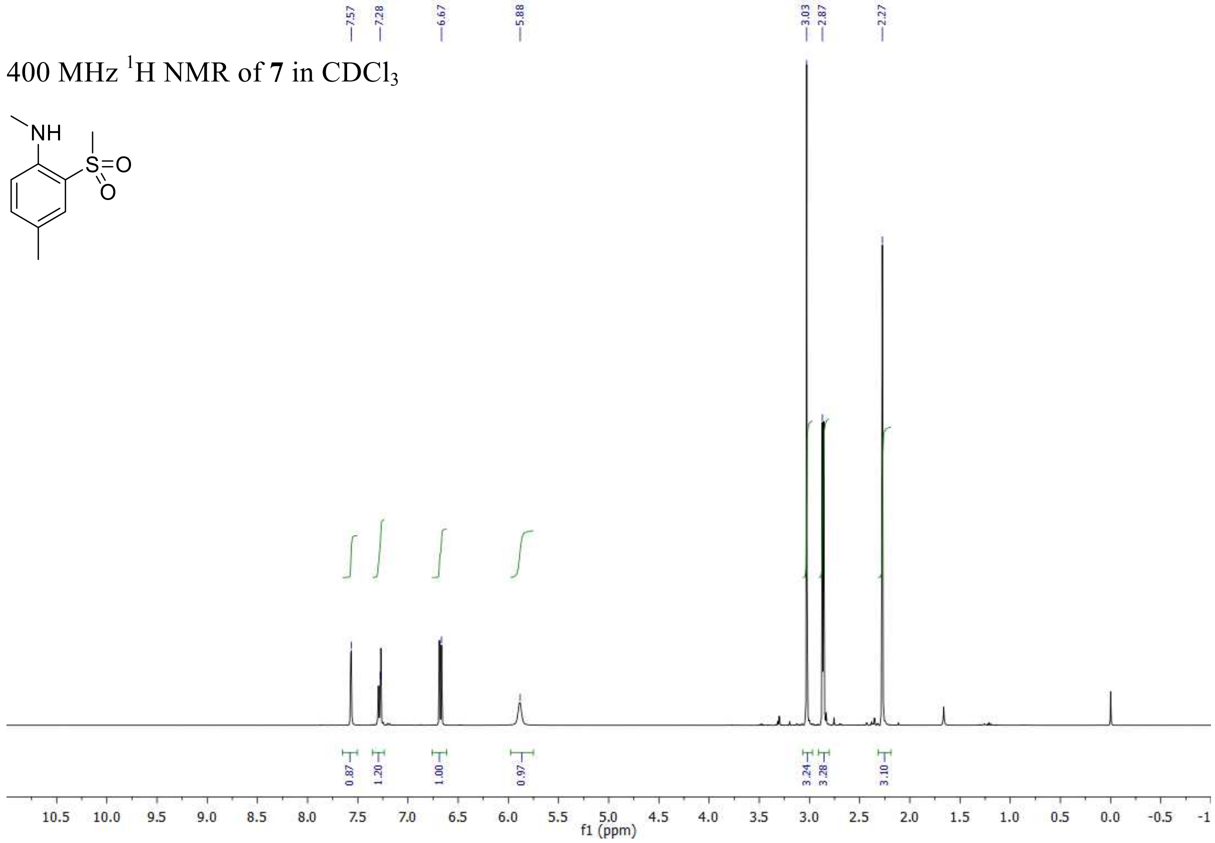

100 MHz ^{13}C NMR of **7** in $CDCl_3$

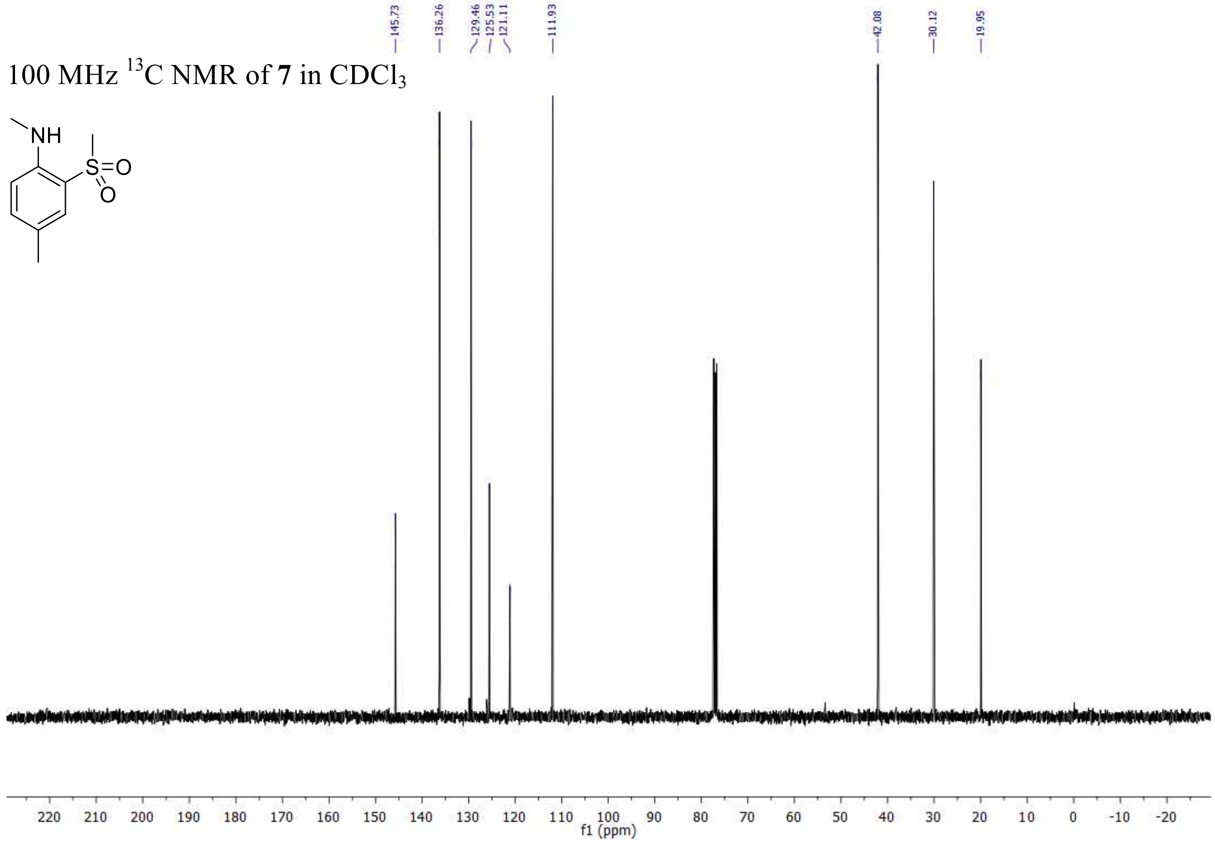

400 MHz ^{1}H NMR of **8** in $CDCl_3$

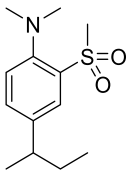

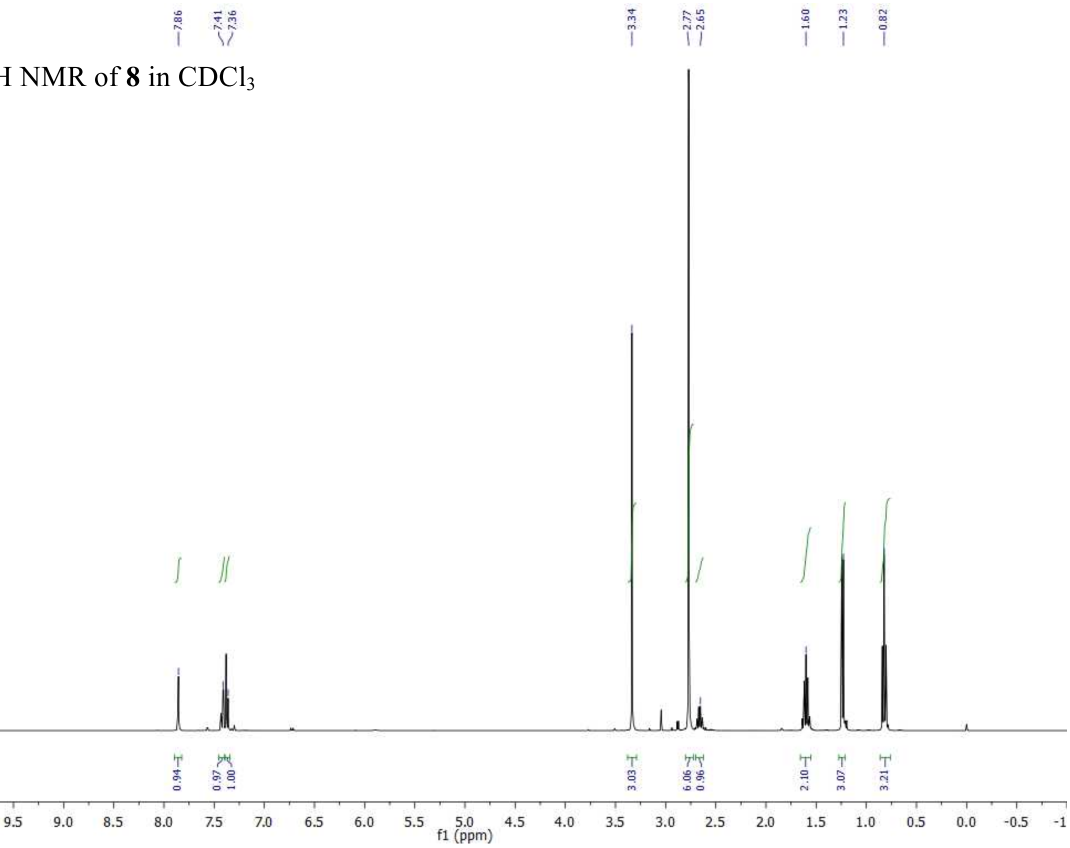

100 MHz ^{13}C NMR of **8** in $CDCl_3$

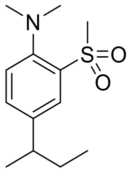

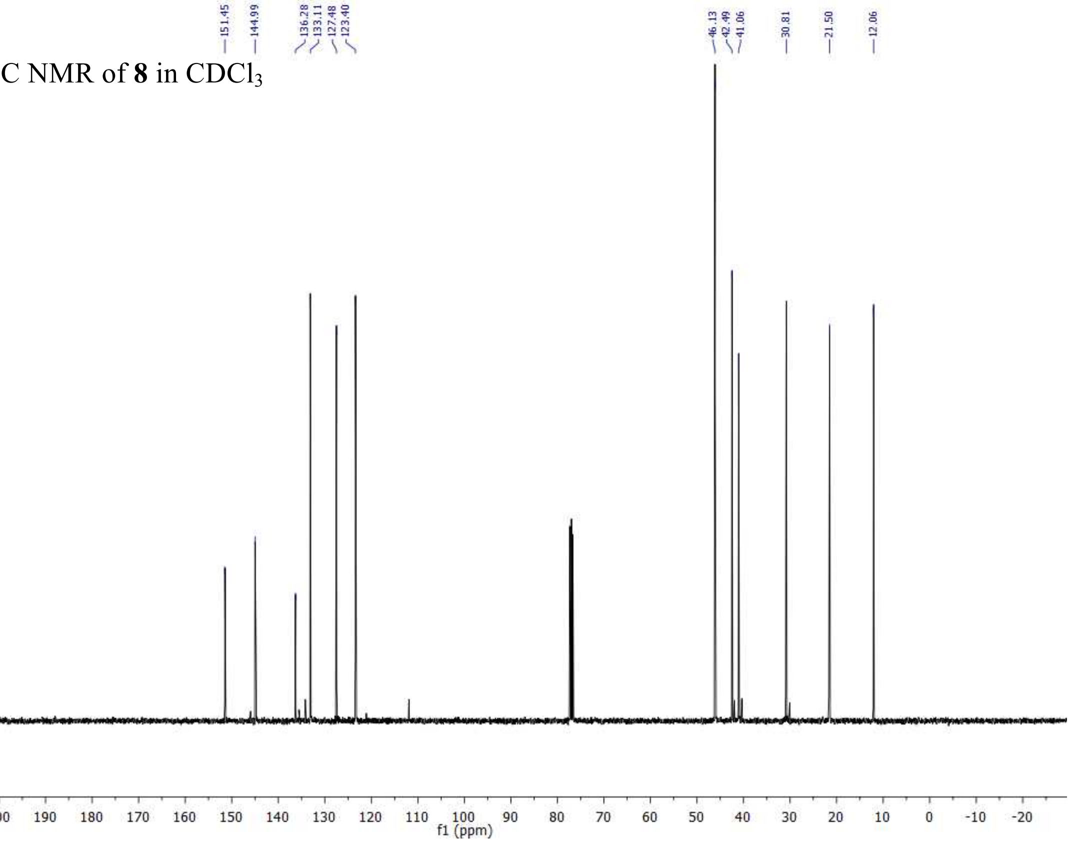

400 MHz ^{1}H NMR of **9** in $CDCl_3$

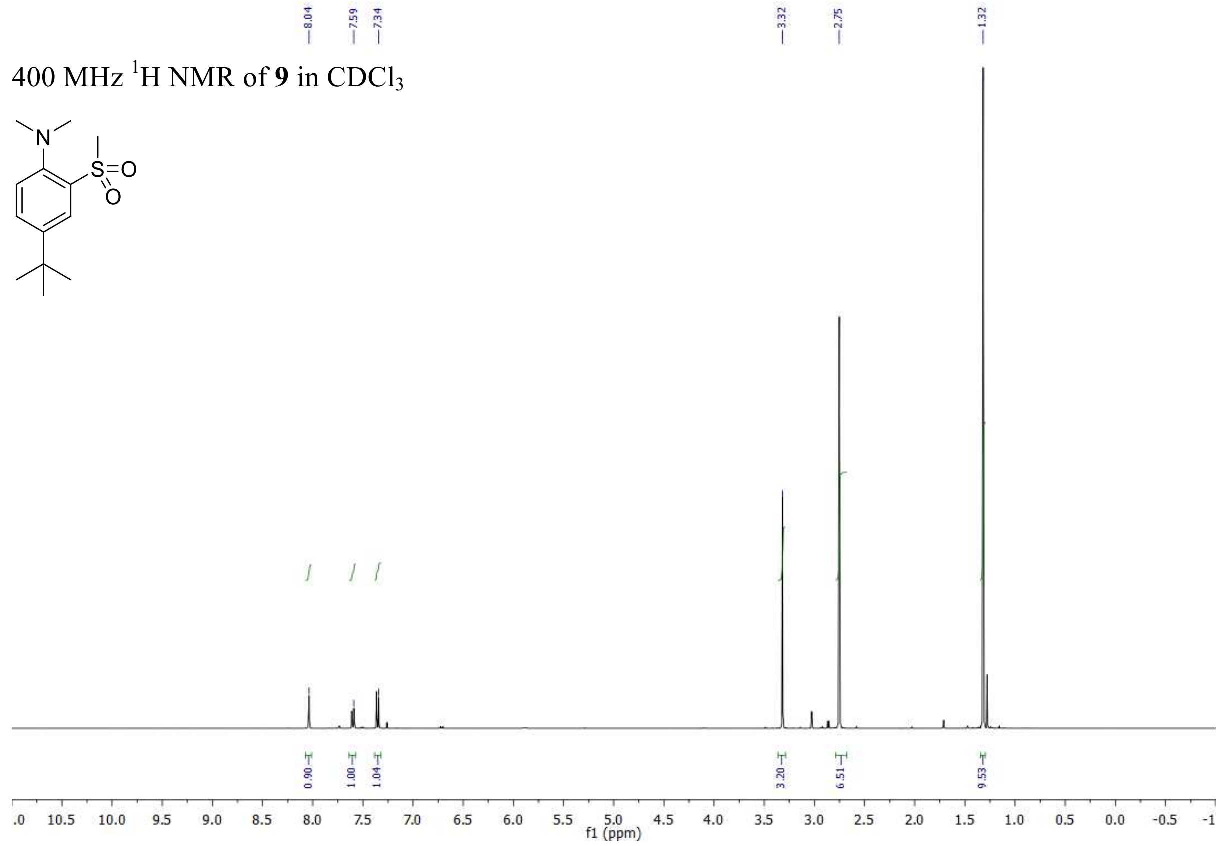

100 MHz ^{13}C NMR of **9** in $CDCl_3$

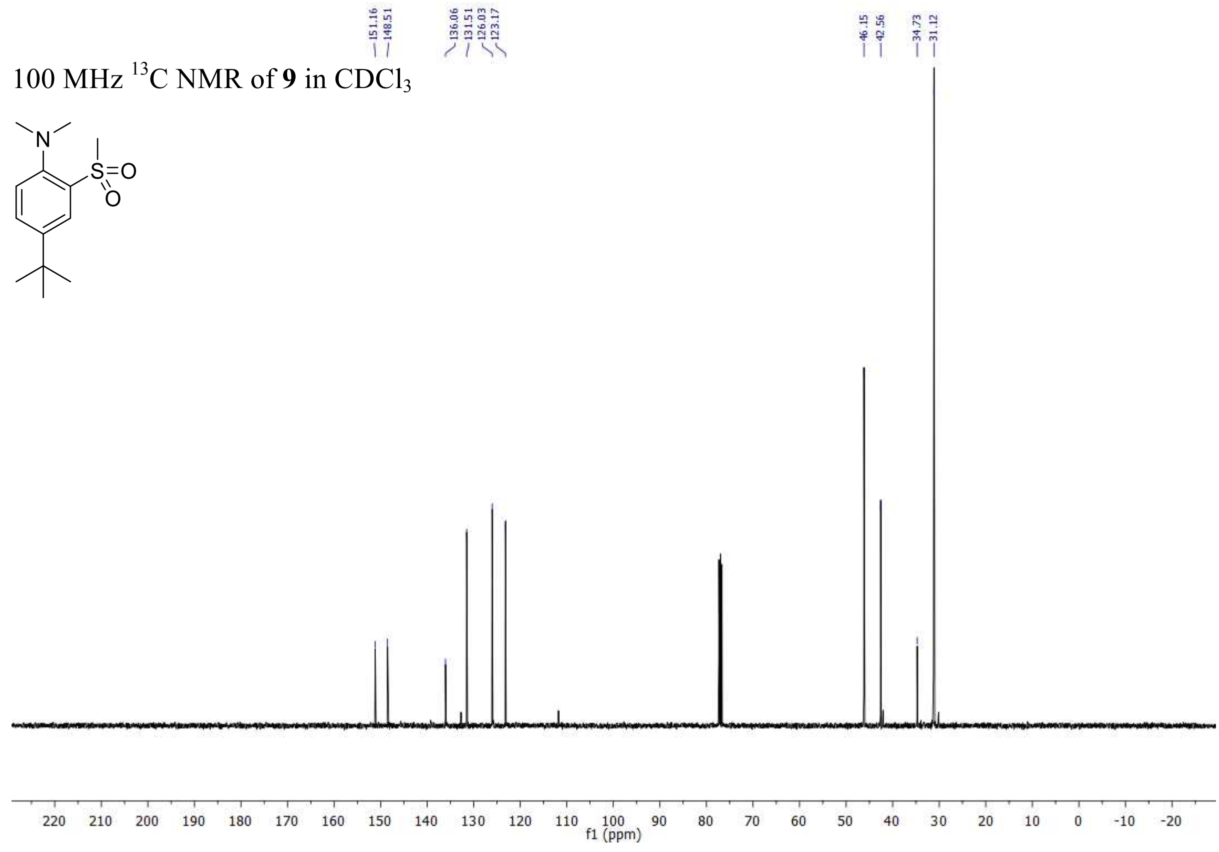

400 MHz ^{1}H NMR of **10** in $CDCl_3$

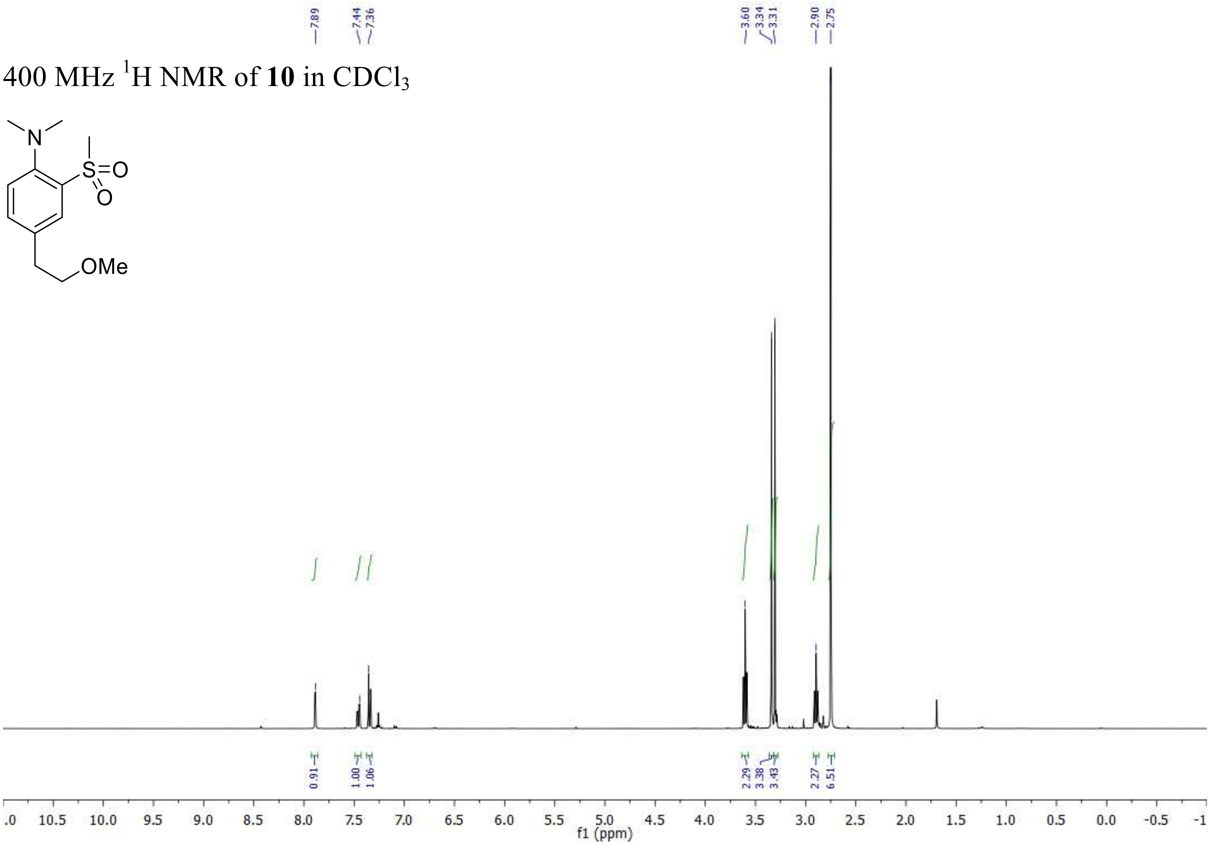

100 MHz ^{13}C NMR of **10** in $CDCl_3$

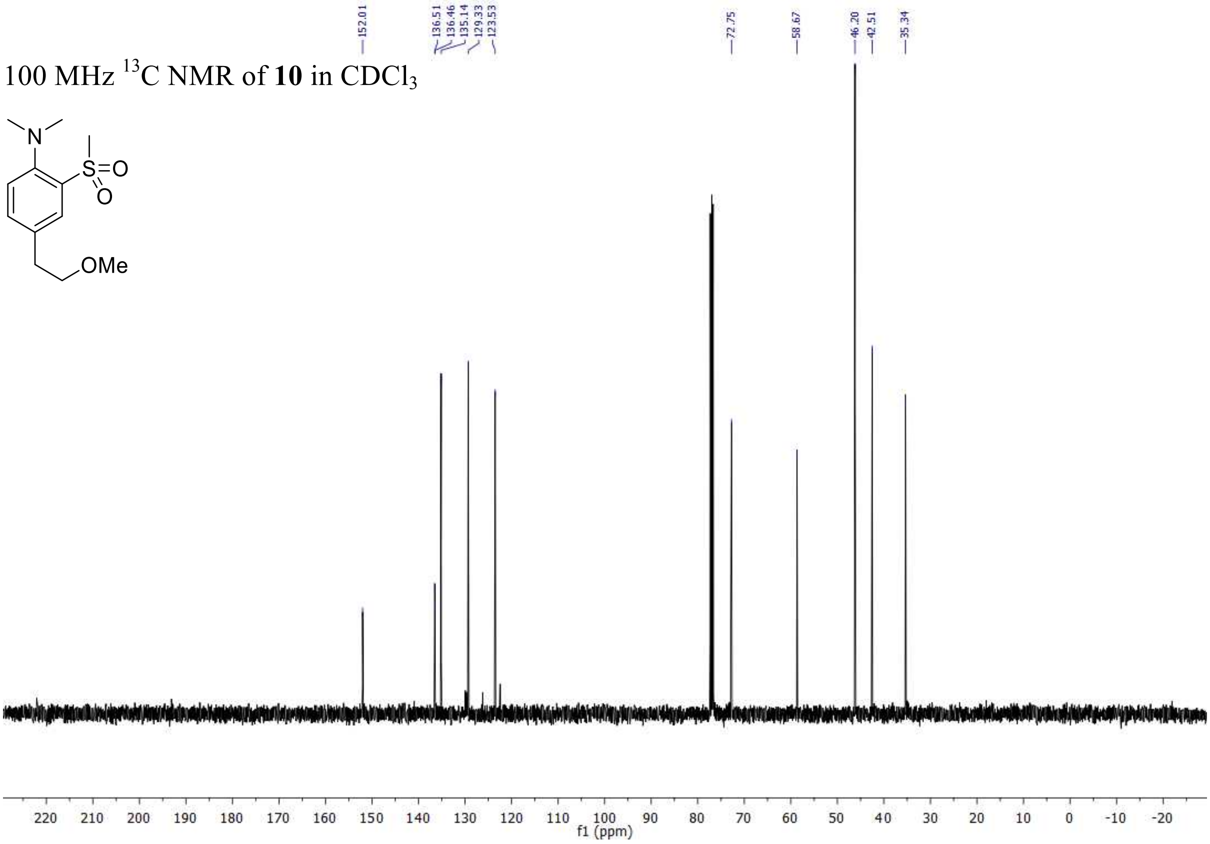

500 MHz ^{1}H NMR of **11** in $CDCl_3$

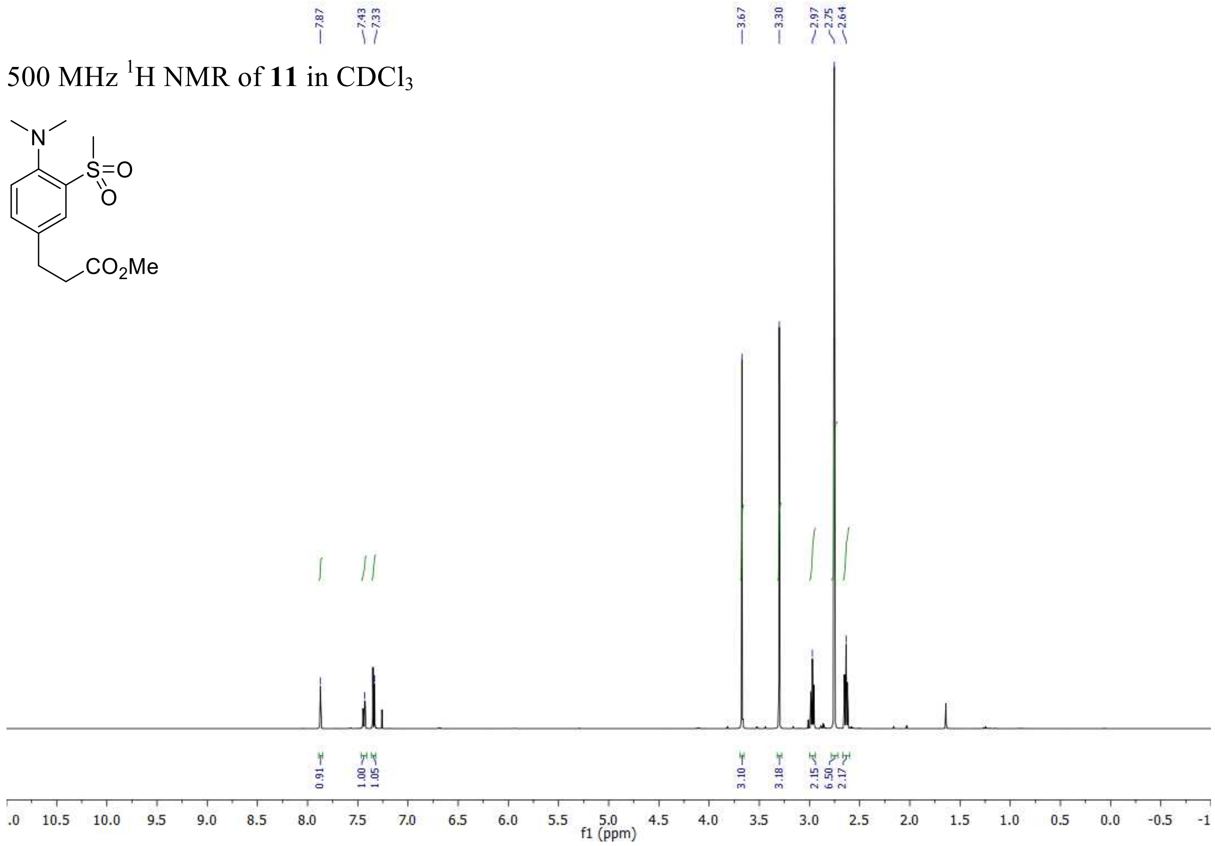

125 MHz ^{13}C NMR of **11** in $CDCl_3$

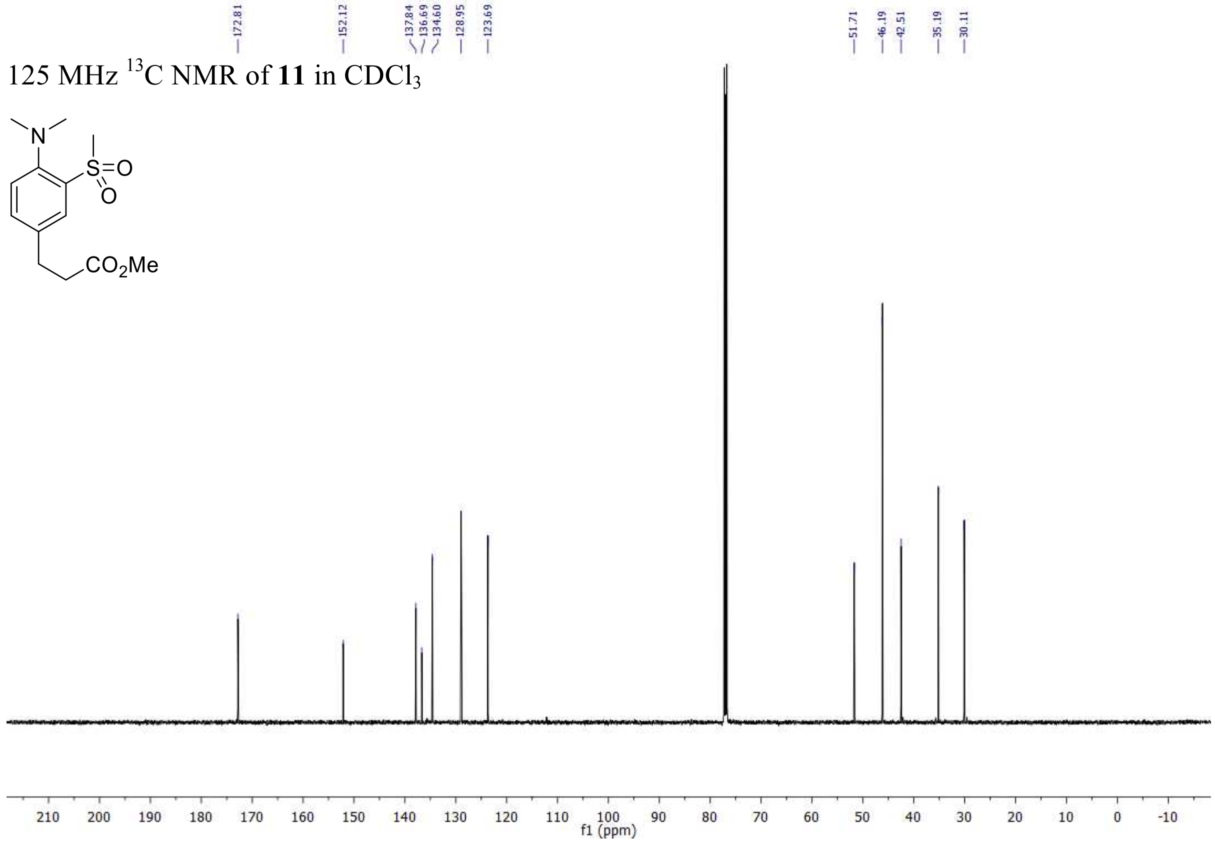

400 MHz ^{1}H NMR of **12** in $CDCl_3$

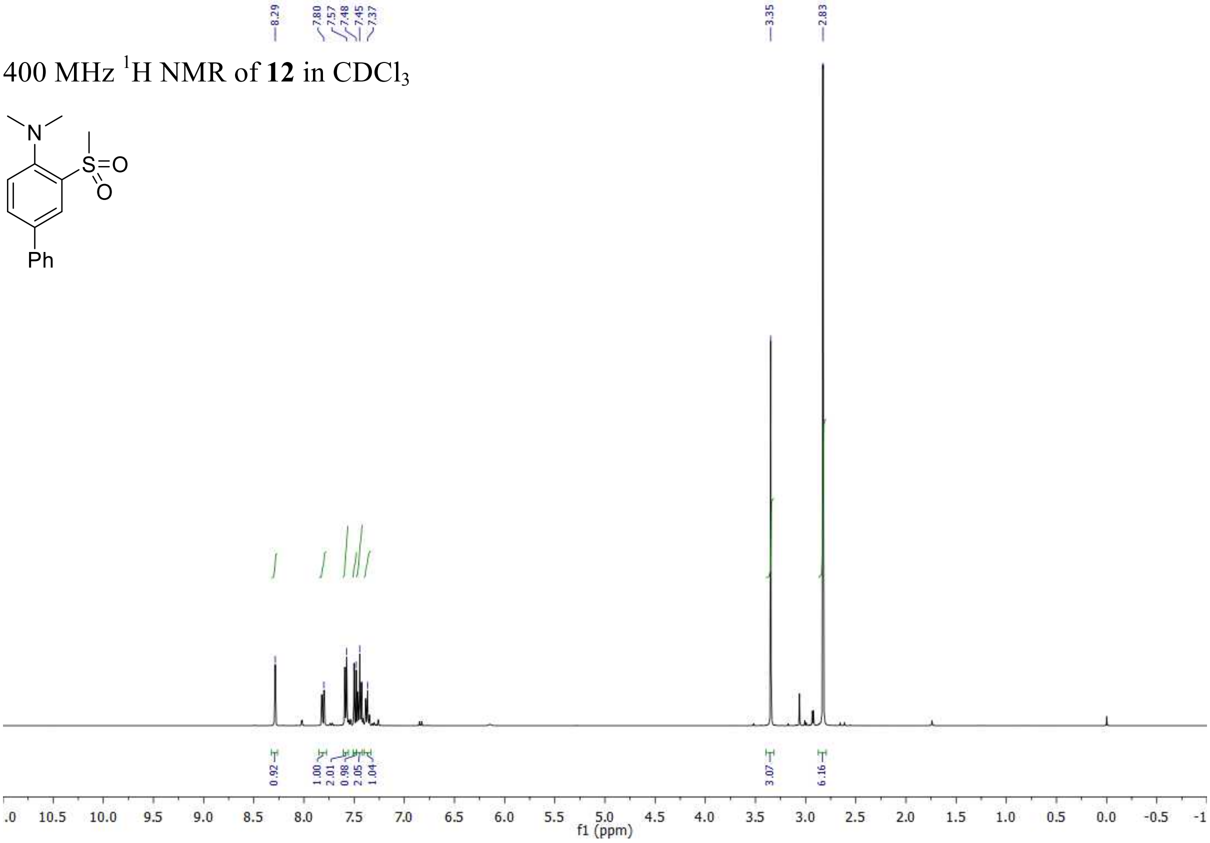

100 MHz ^{13}C NMR of **12** in $CDCl_3$

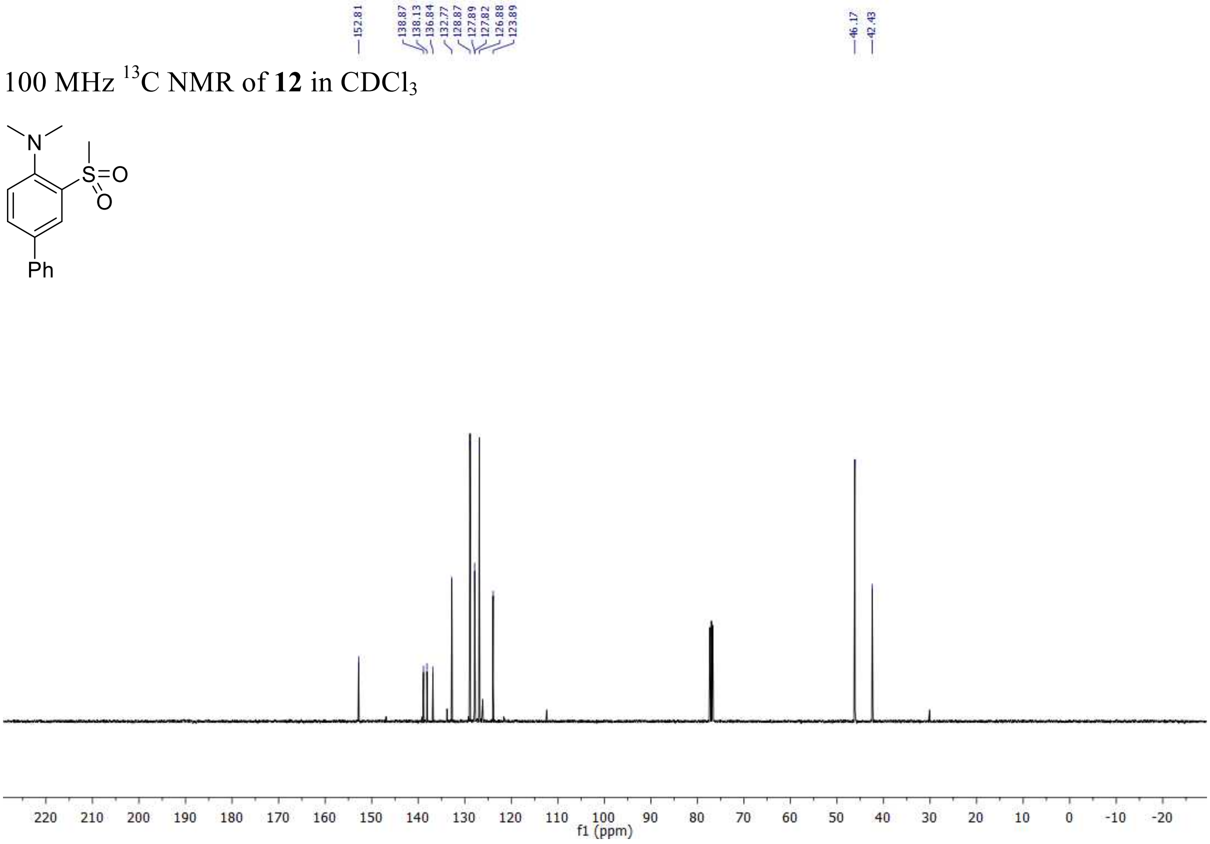

400 MHz ^{1}H NMR of **13a** in $CDCl_3$

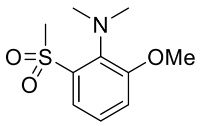

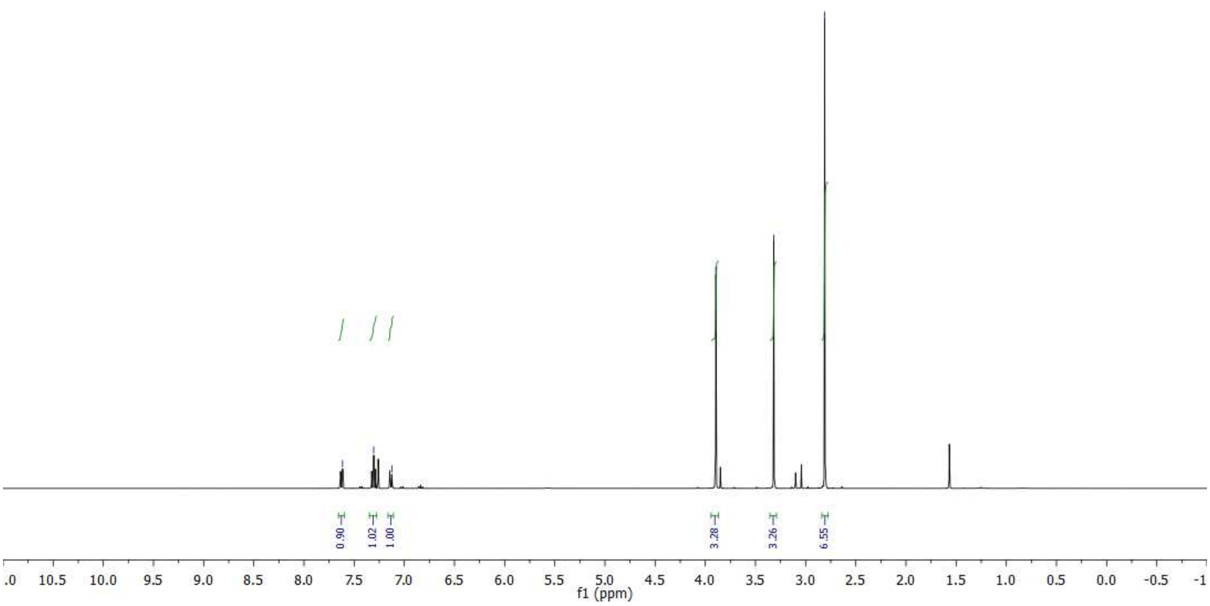

100 MHz ^{13}C NMR of **13a** in $CDCl_3$

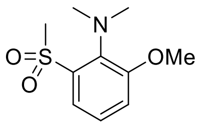

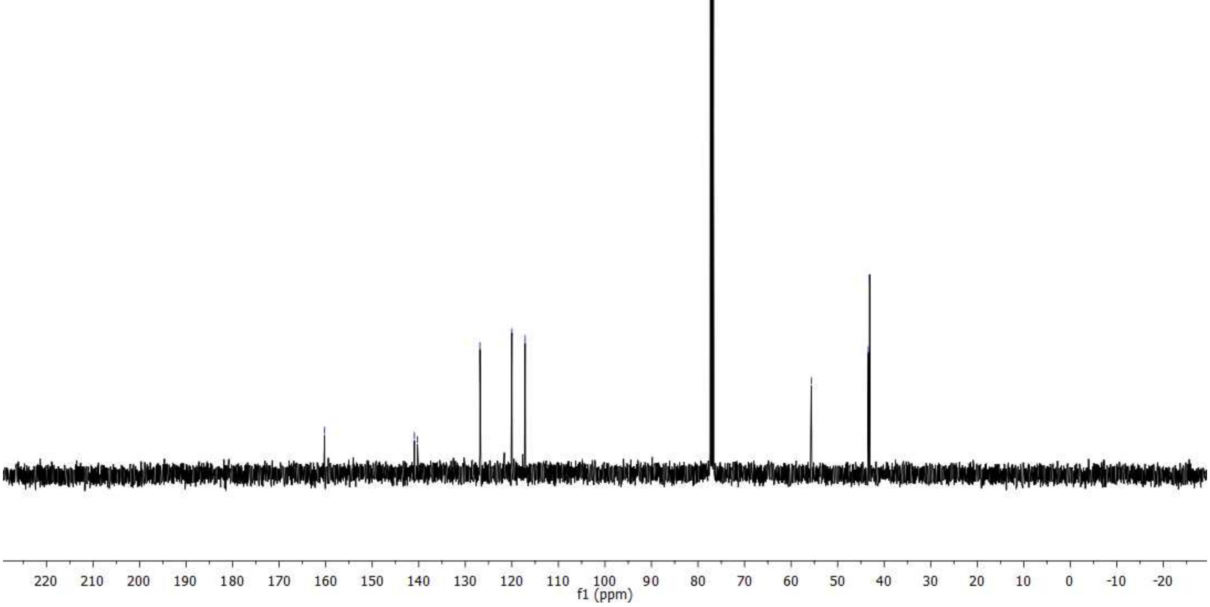

400 MHz ^{1}H NMR of **13b** in $CDCl_3$

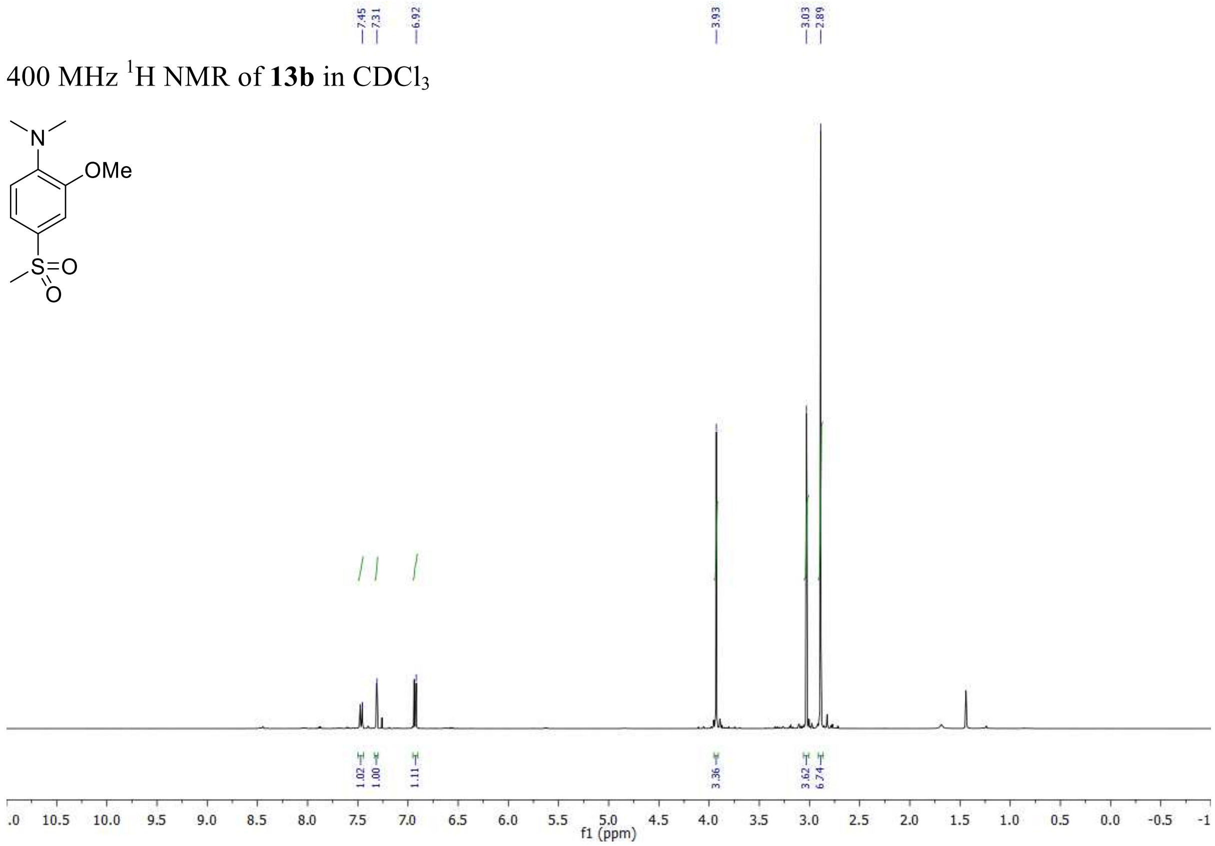

100 MHz ^{13}C NMR of **13b** in $CDCl_3$

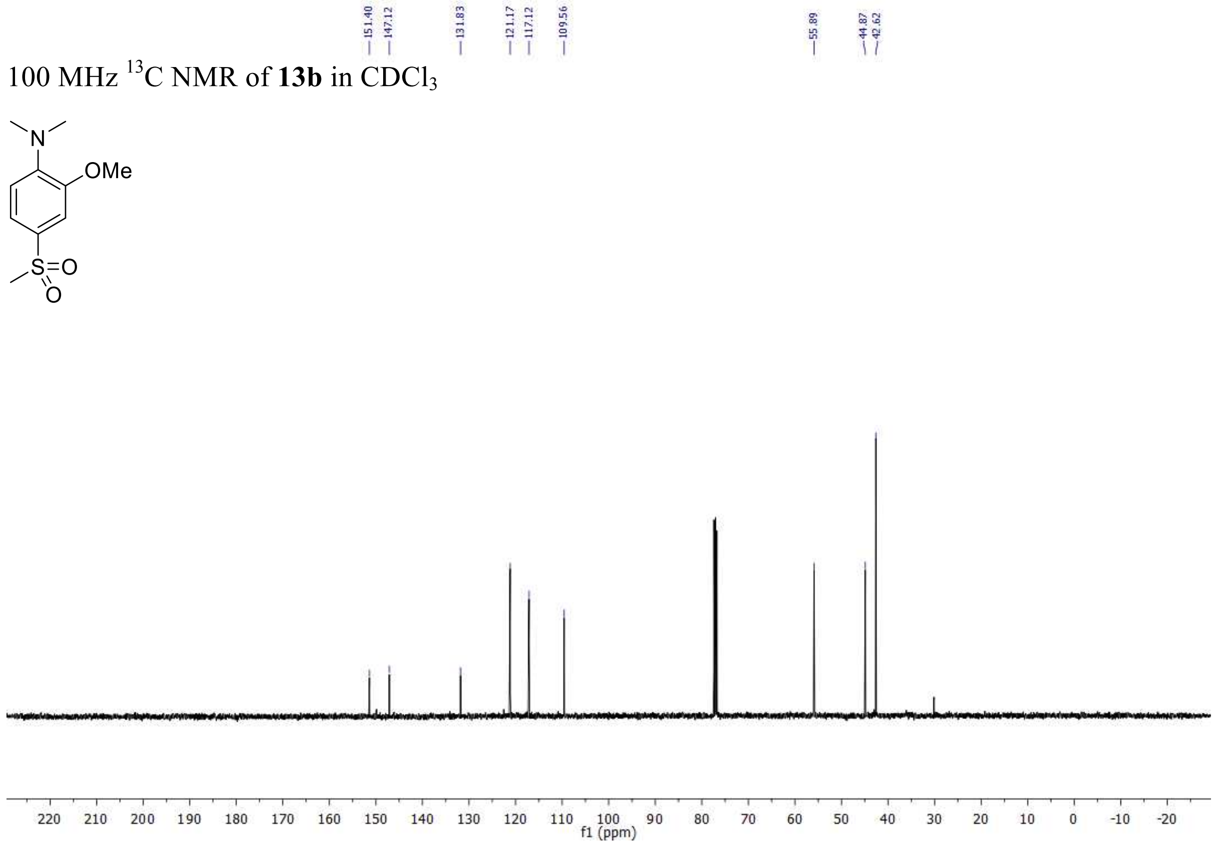

400 MHz ^{1}H NMR of **14a** in $CDCl_3$

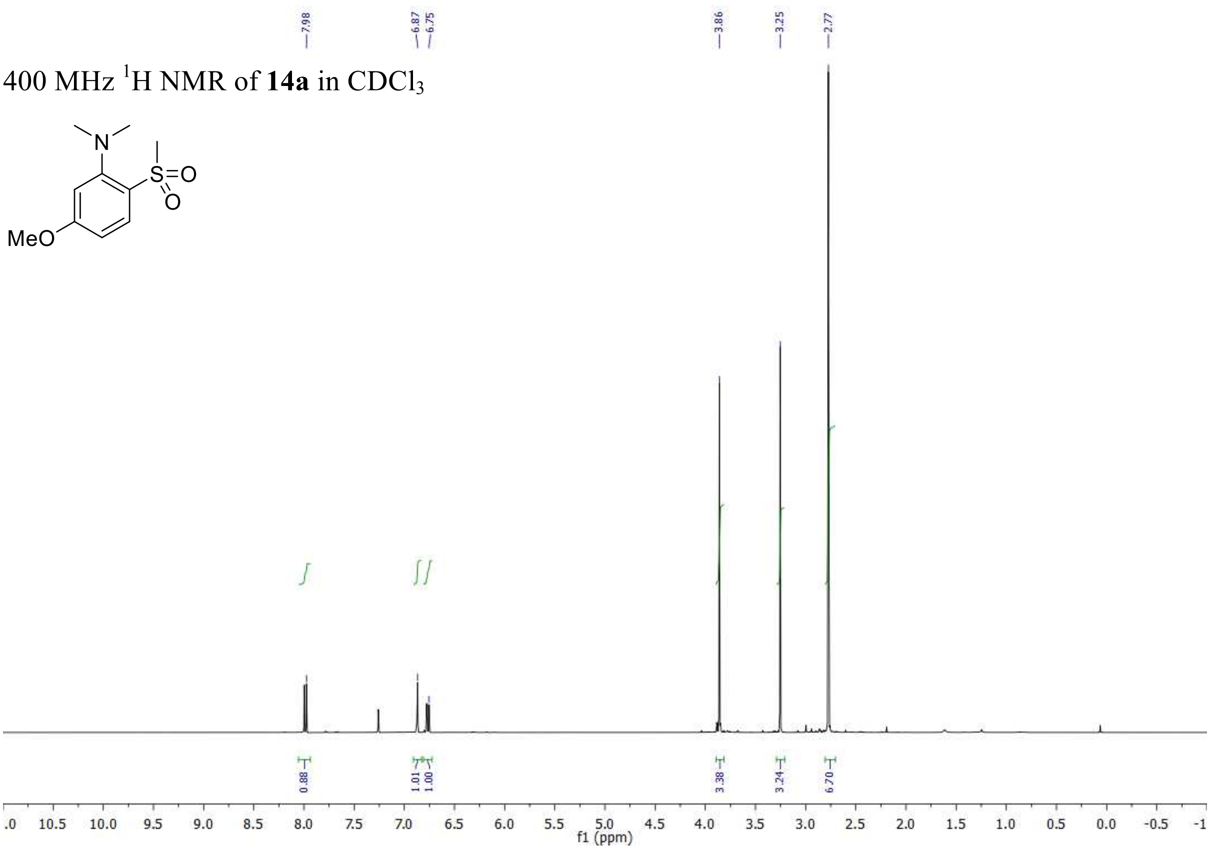

100 MHz ^{13}C NMR of **14a** in $CDCl_3$

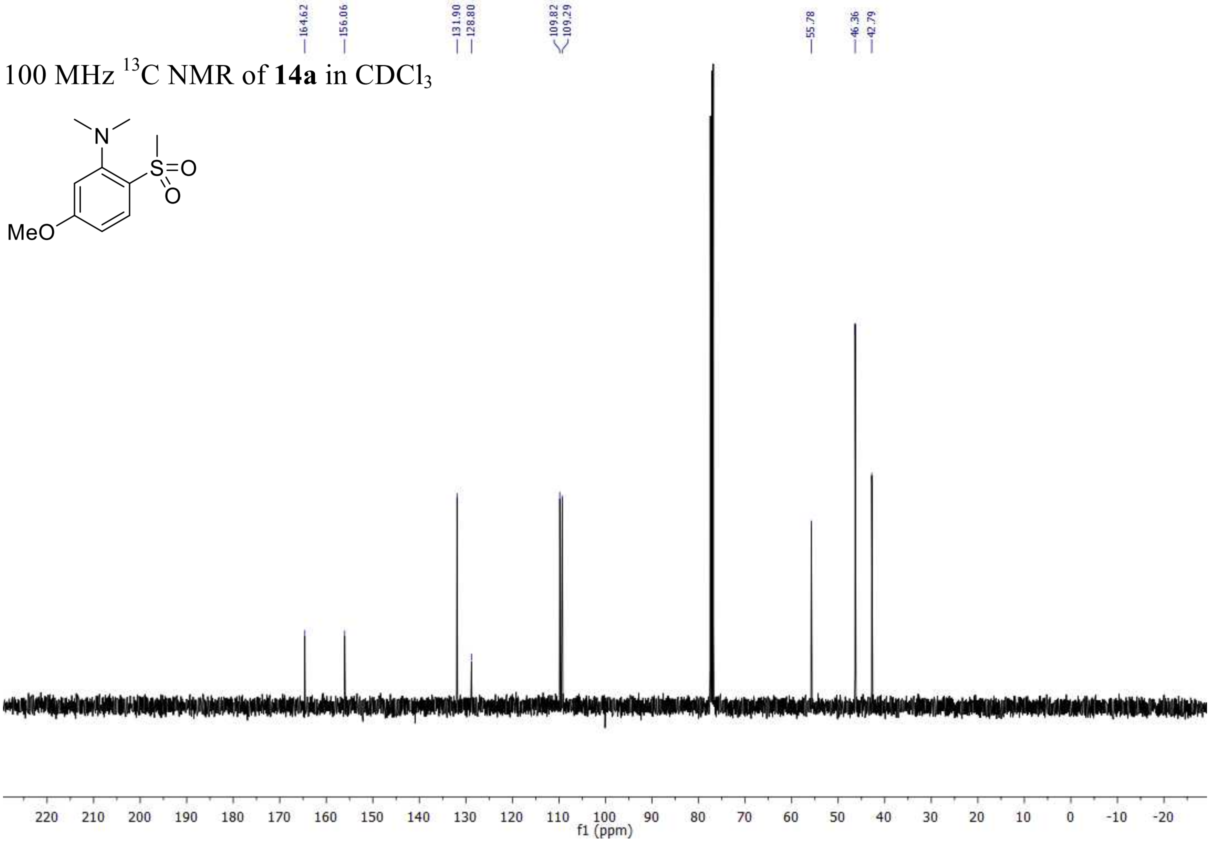

400 MHz ^{1}H NMR of **14b** in $CDCl_3$

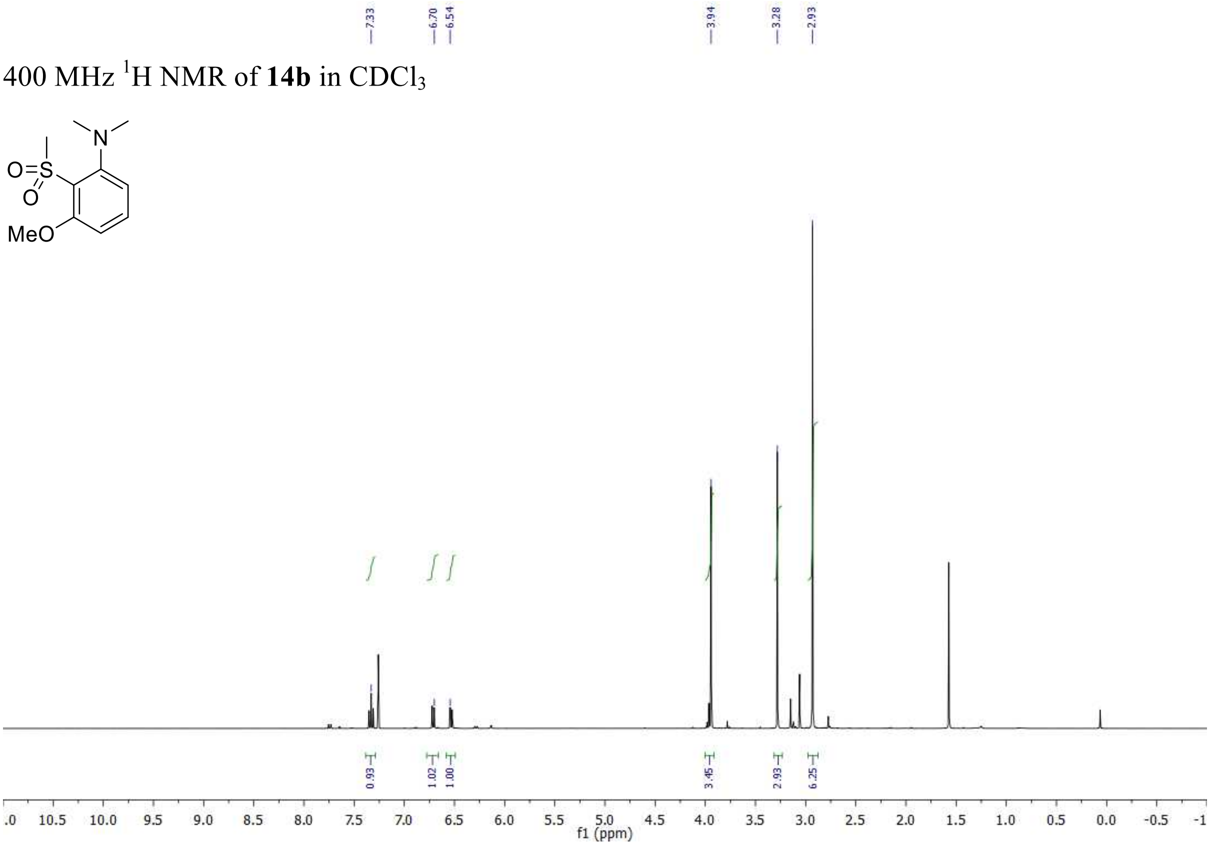

100 MHz ^{13}C NMR of **14b** in $CDCl_3$

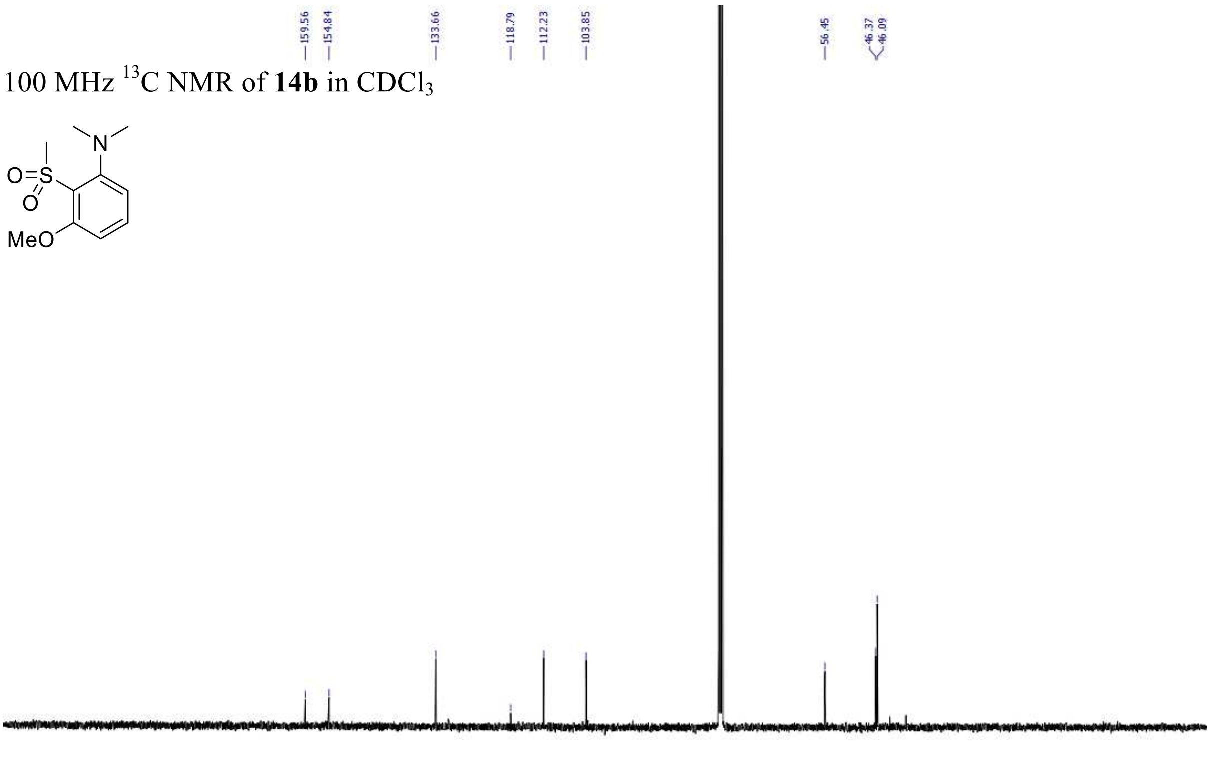

400 MHz ^{1}H NMR of **14c** in $CDCl_3$

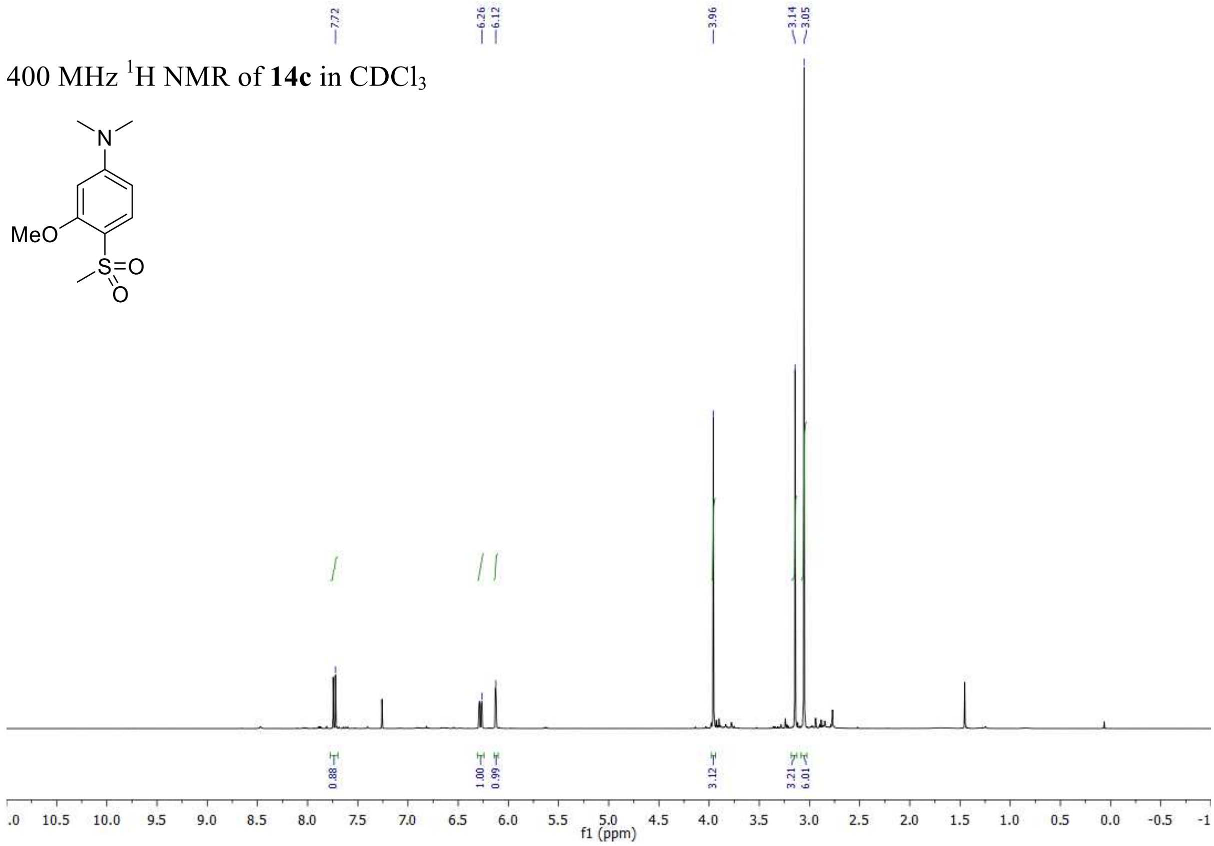

100 MHz ^{13}C NMR of **14c** in $CDCl_3$

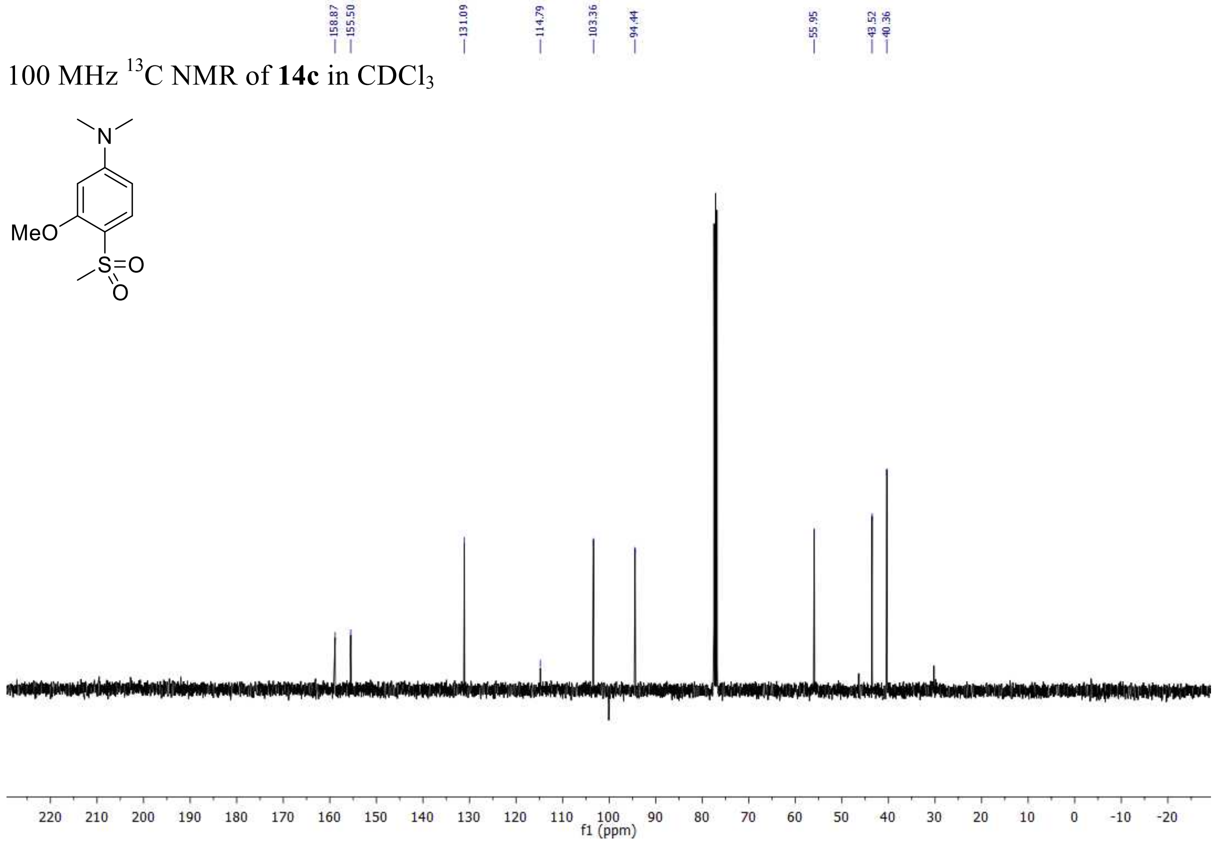

400 MHz ^{1}H NMR of **15** in $CDCl_3$

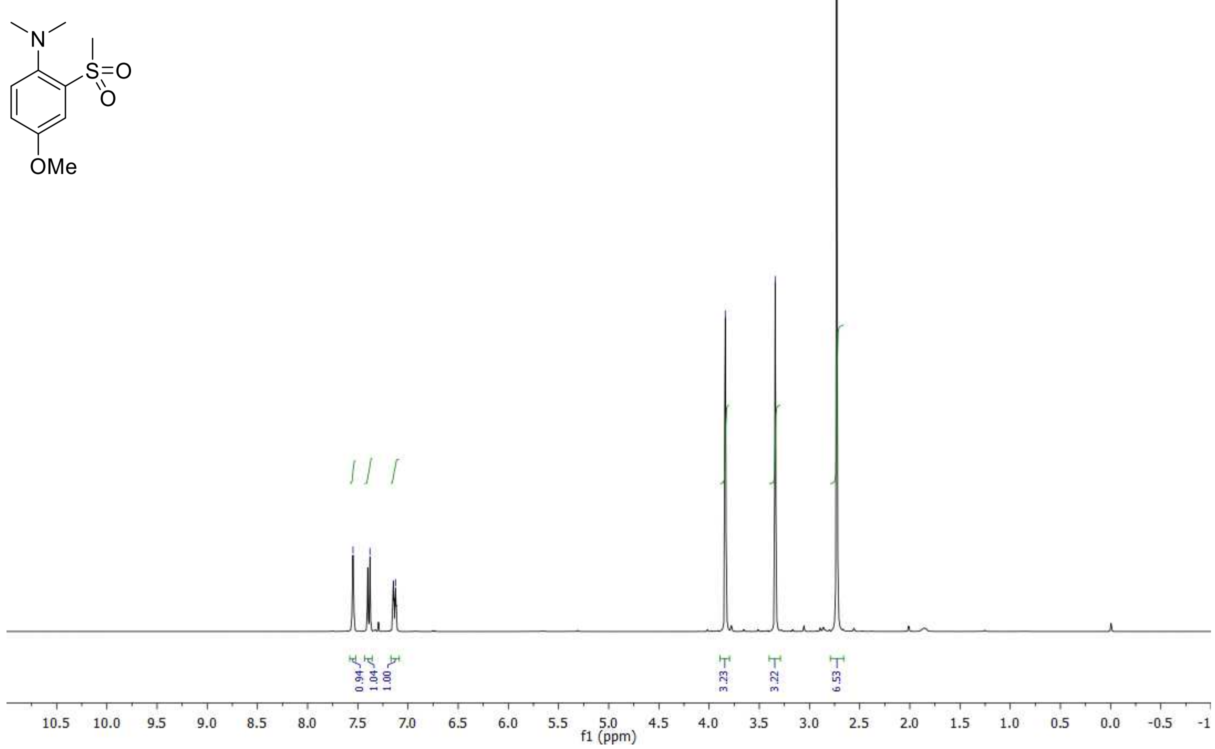

100 MHz ^{13}C NMR of **15** in $CDCl_3$

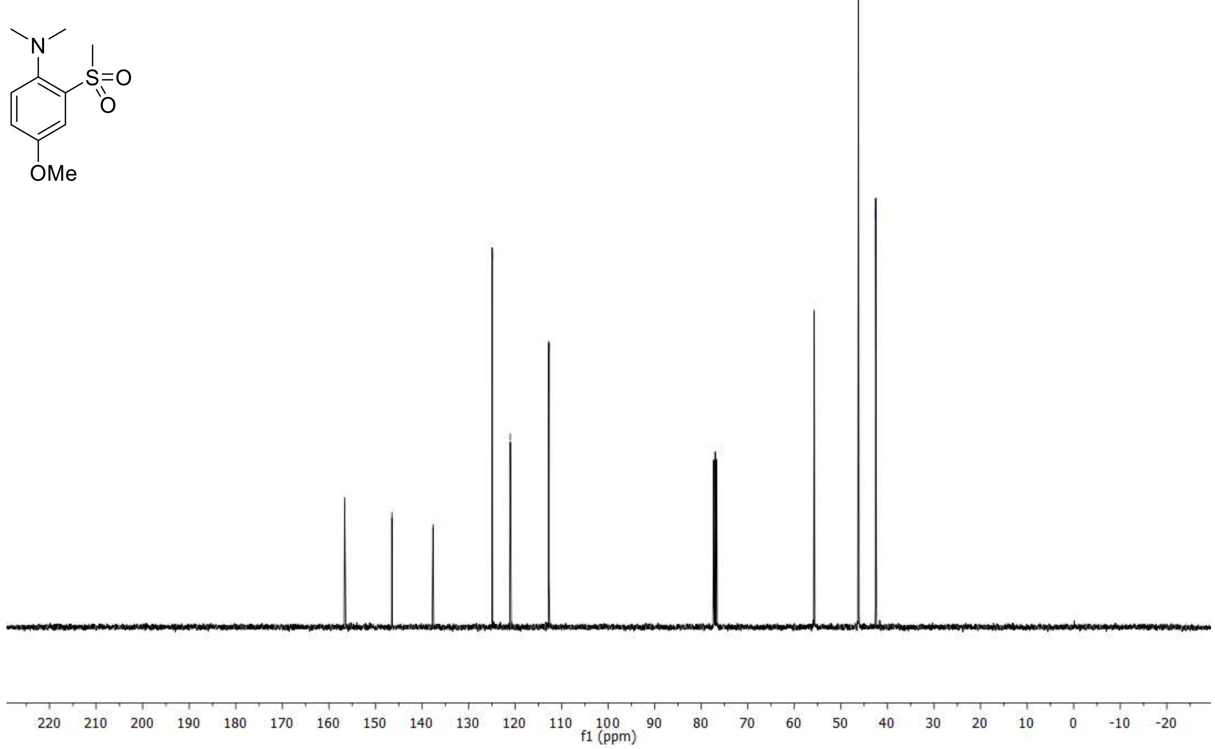

400 MHz ^{1}H NMR of **16** in $CDCl_3$

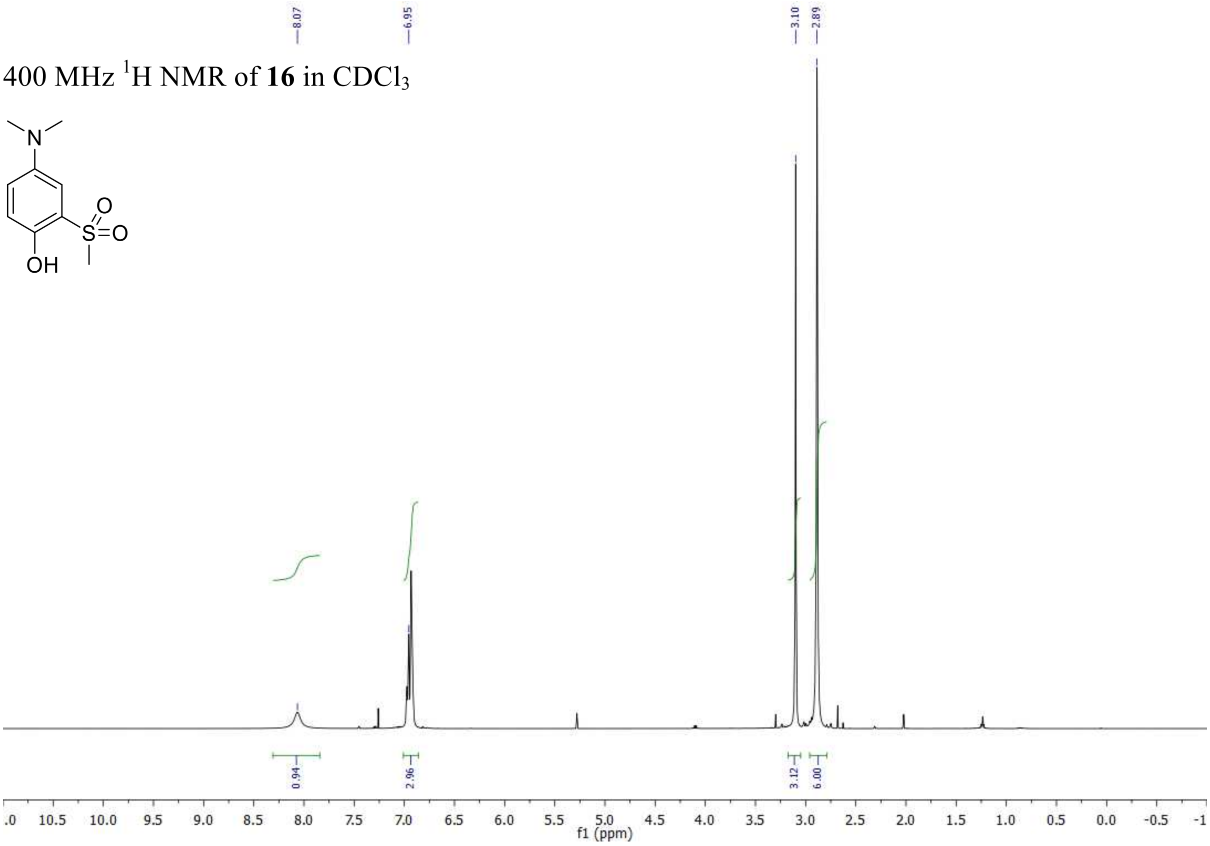

100 MHz ^{13}C NMR of **16** in $CDCl_3$

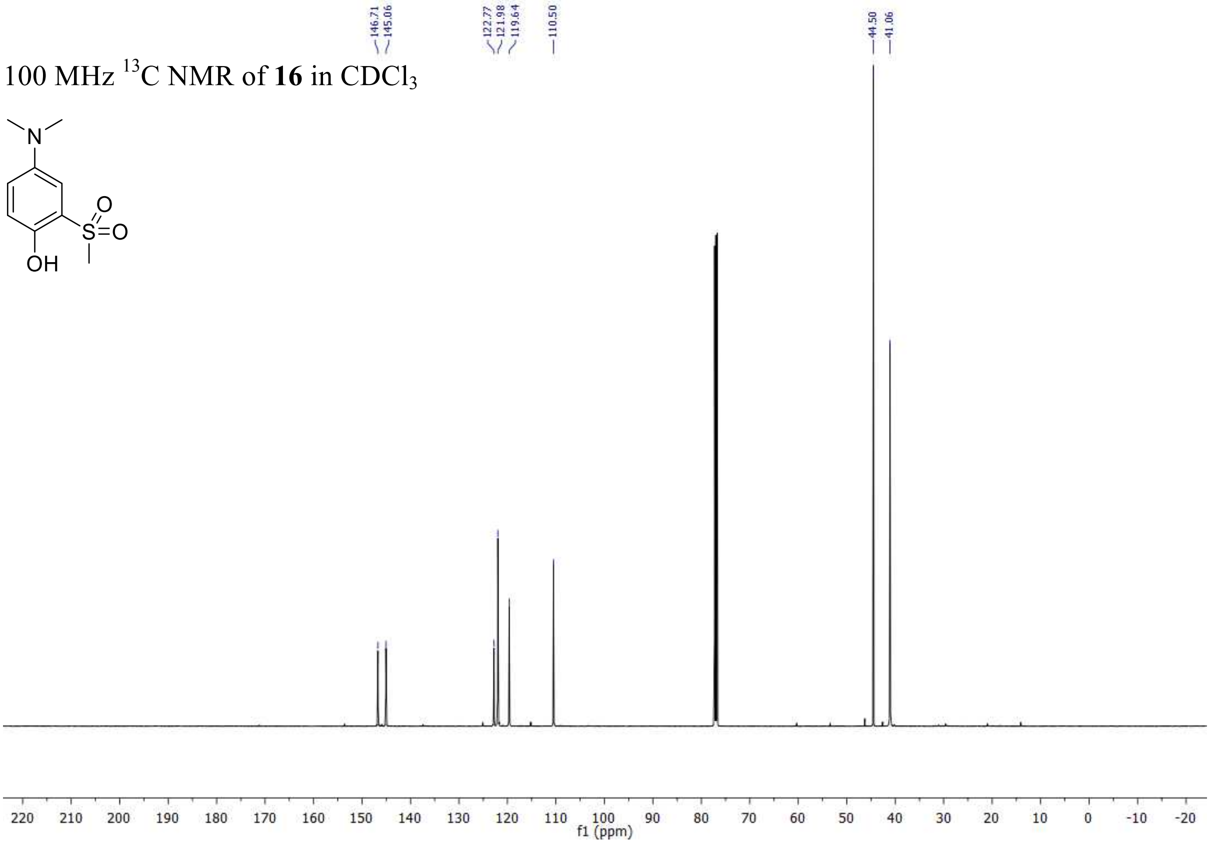

400 MHz ^{1}H NMR of **17** in $CDCl_3$

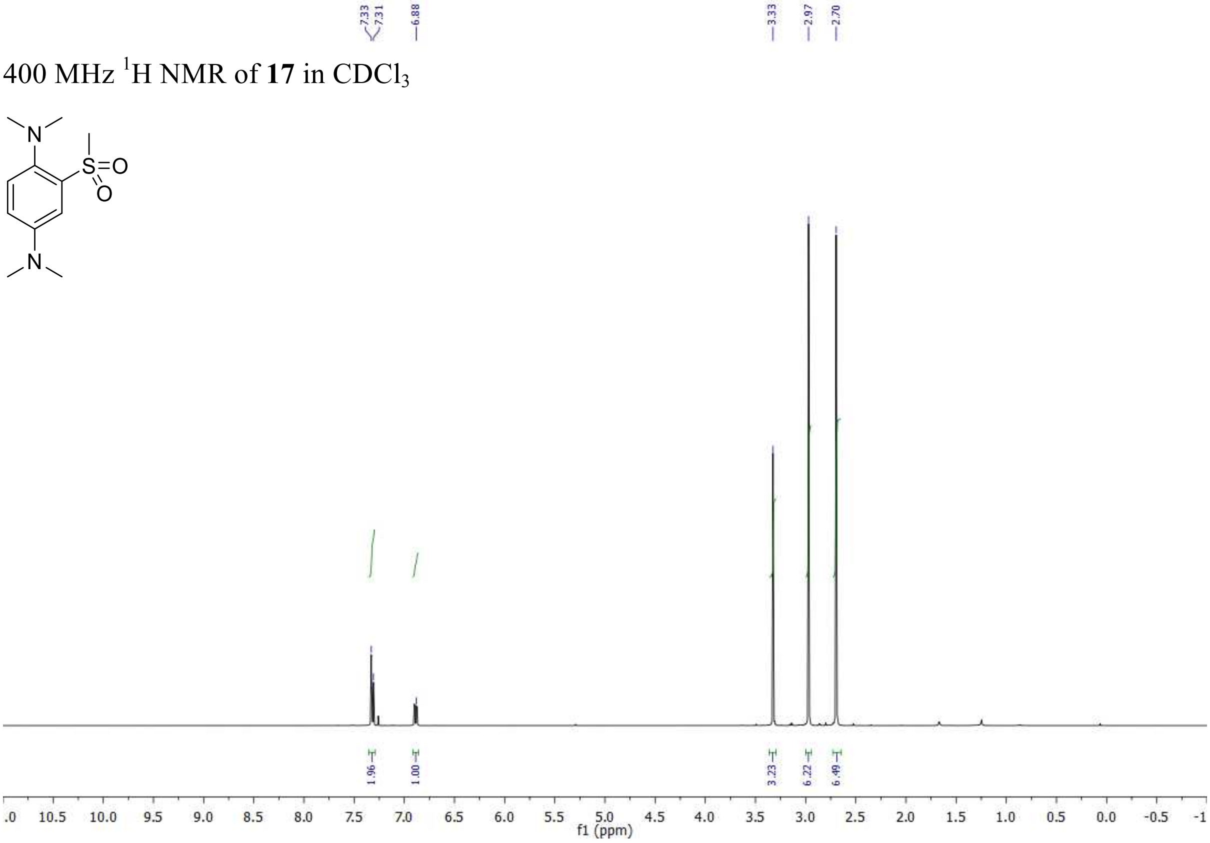

100 MHz ^{13}C NMR of **17** in $CDCl_3$

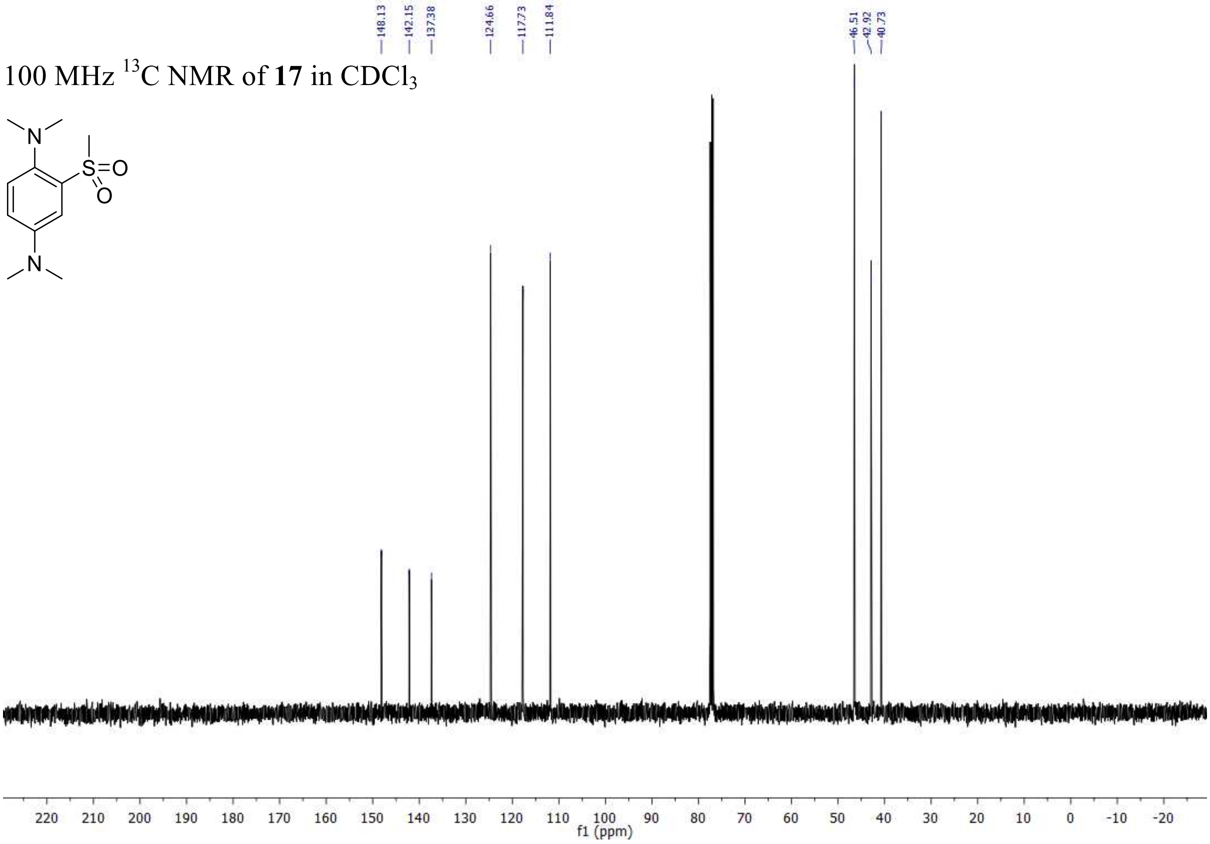

400 MHz ^{1}H NMR of **18** in $CDCl_3$

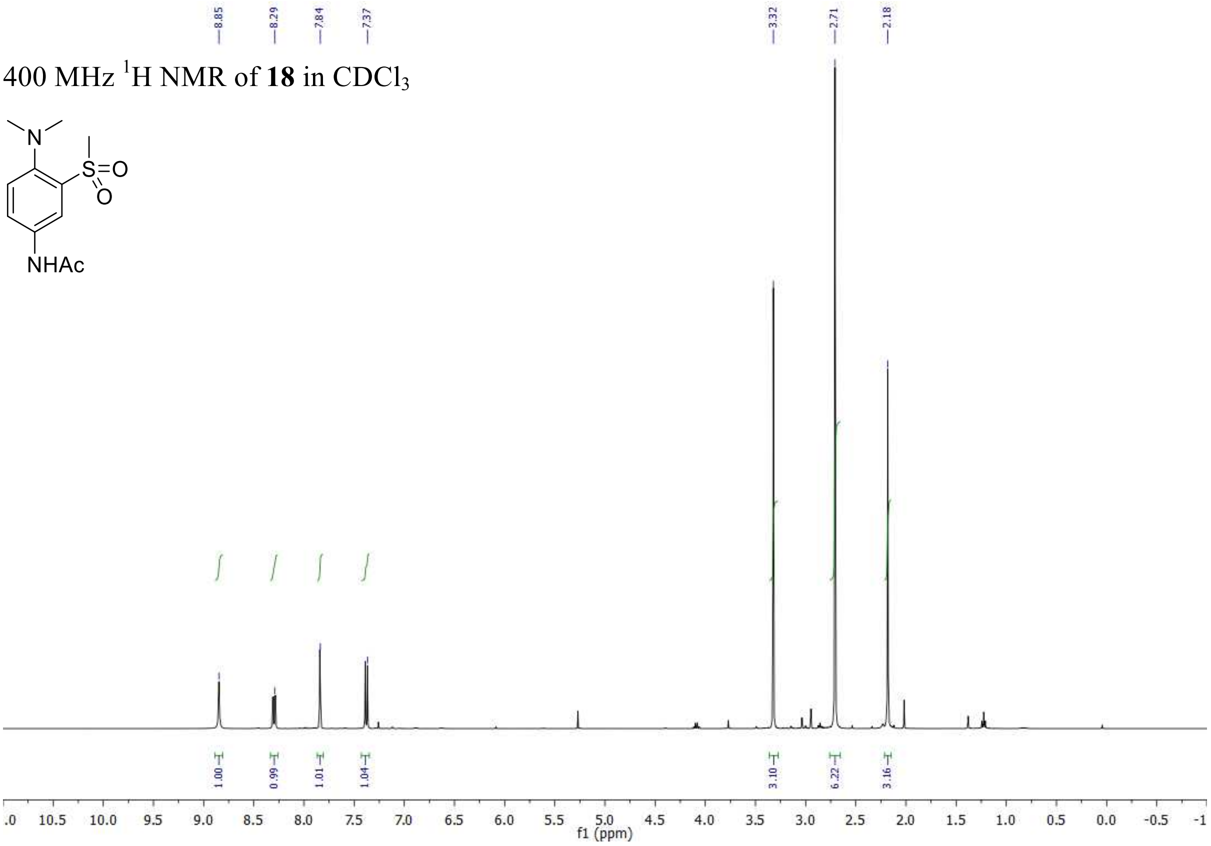

100 MHz ^{13}C NMR of **18** in $CDCl_3$

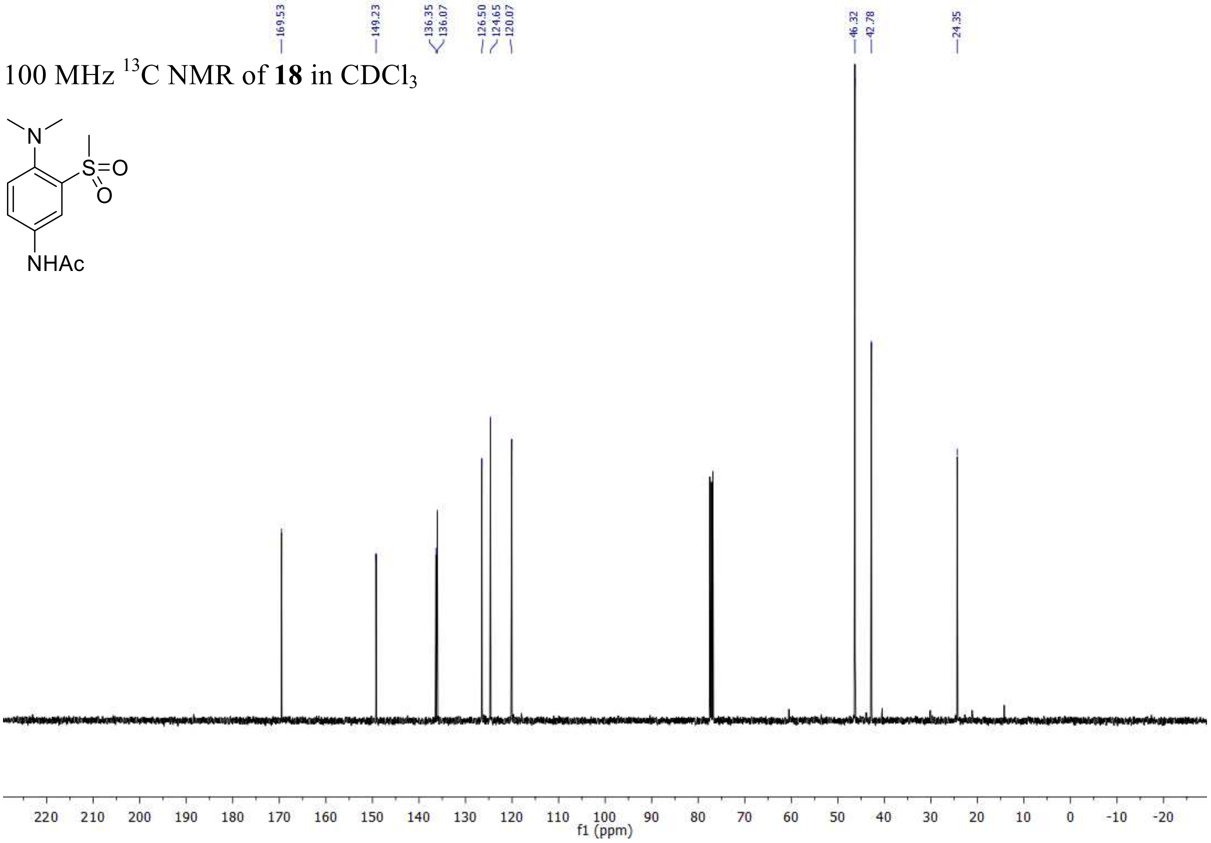

500 MHz ^{1}H NMR of **19** in $CDCl_3$

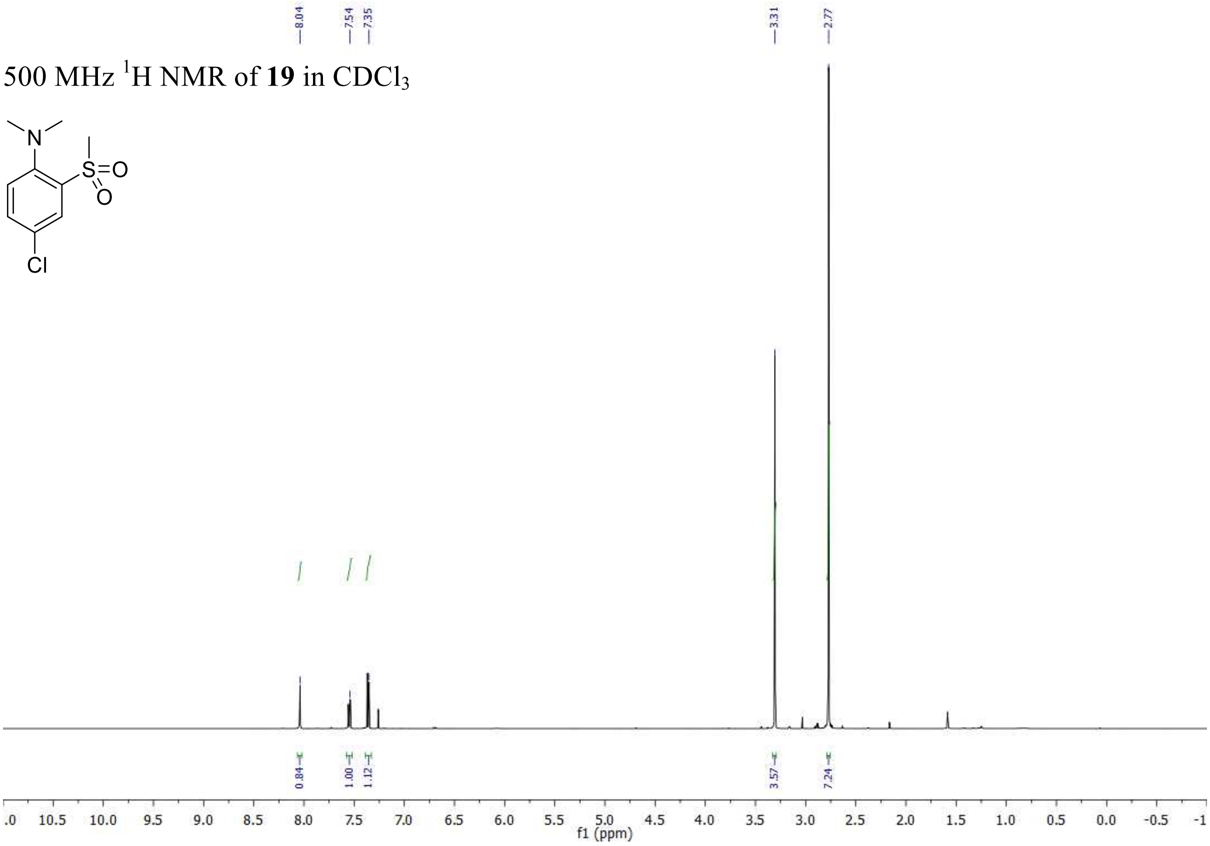

125 MHz ^{13}C NMR of **19** in $CDCl_3$

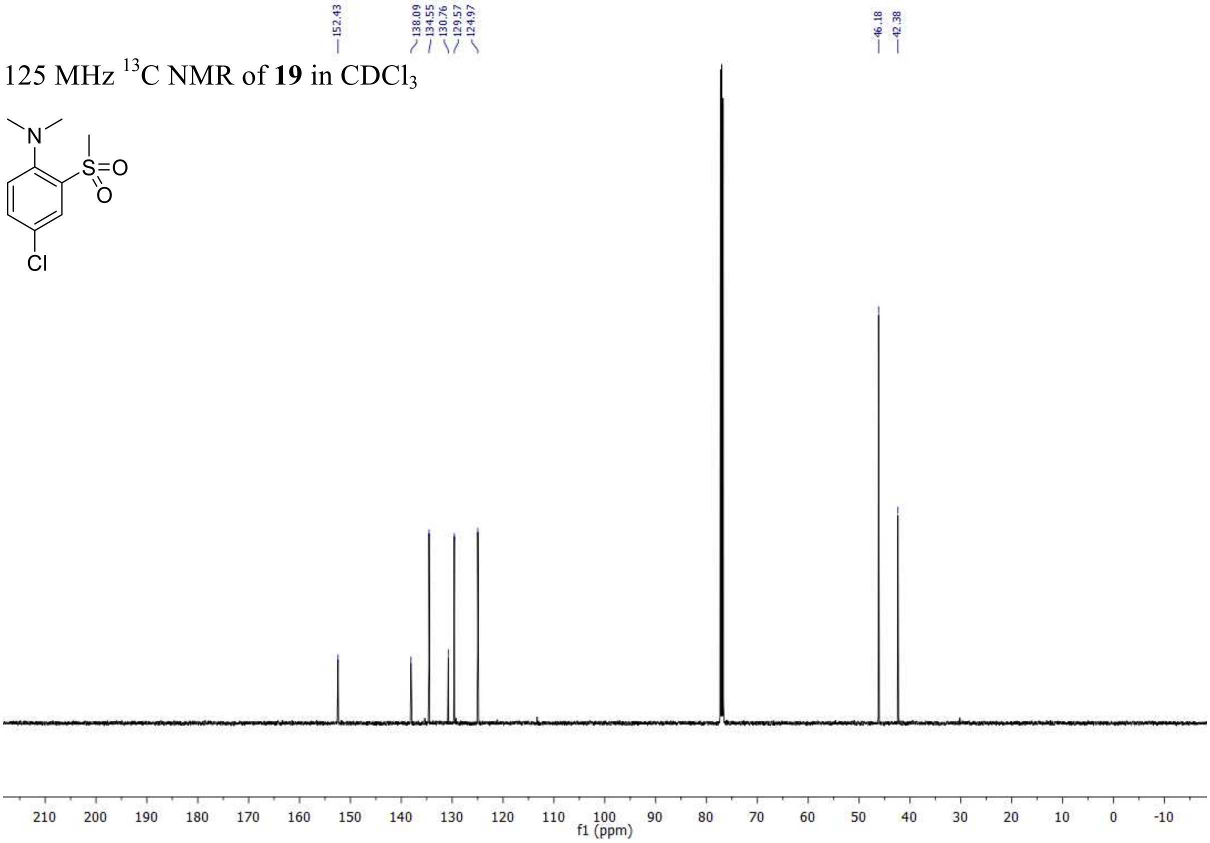

400 MHz ^{1}H NMR of **20** in $CDCl_3$

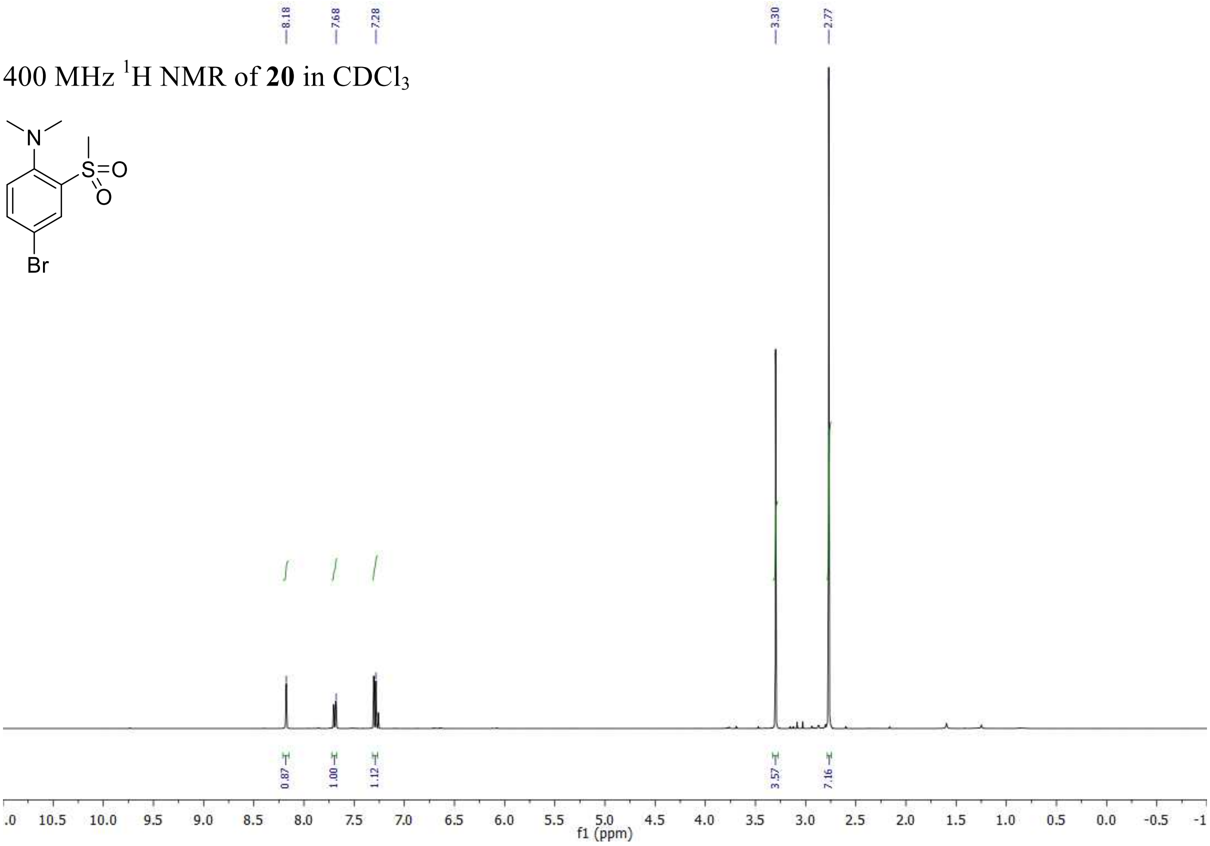

100 MHz ^{13}C NMR of **20** in $CDCl_3$

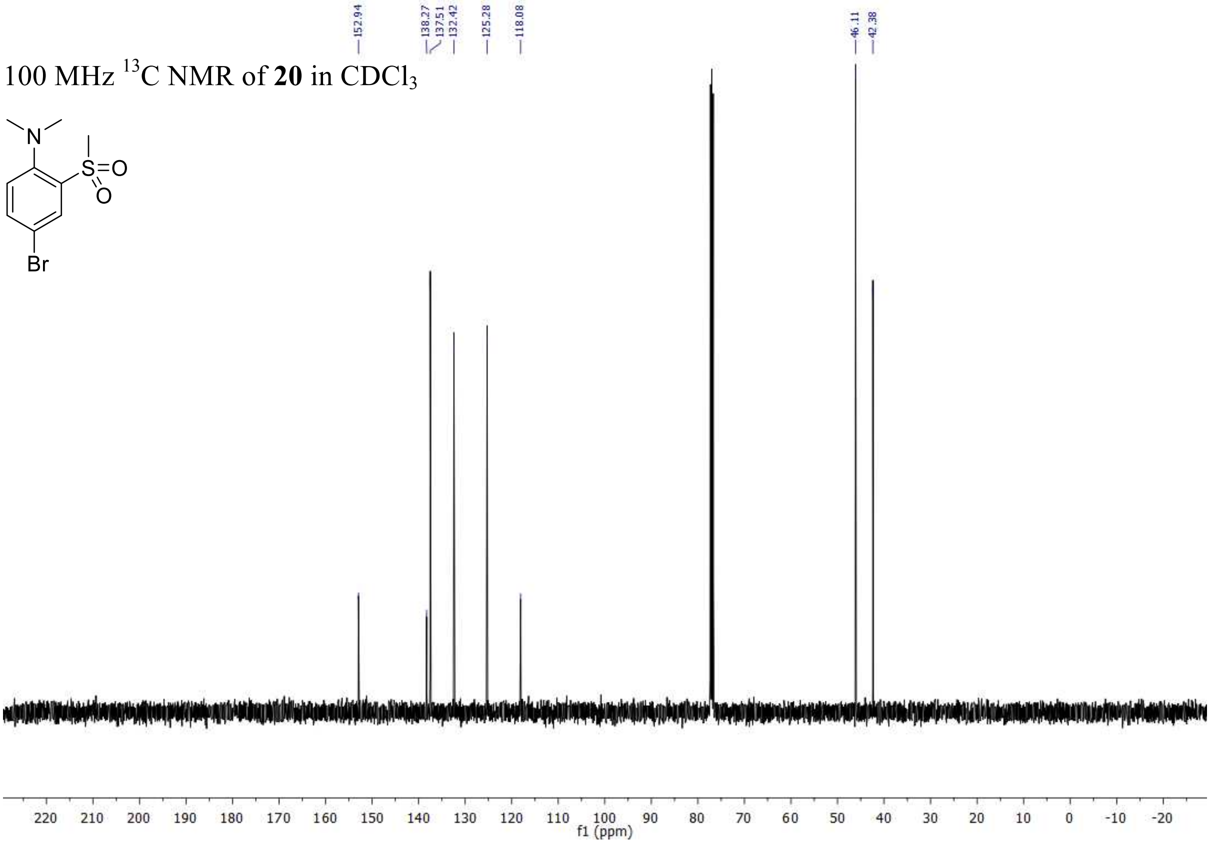

400 MHz ^{1}H NMR of **21** in $CDCl_3$

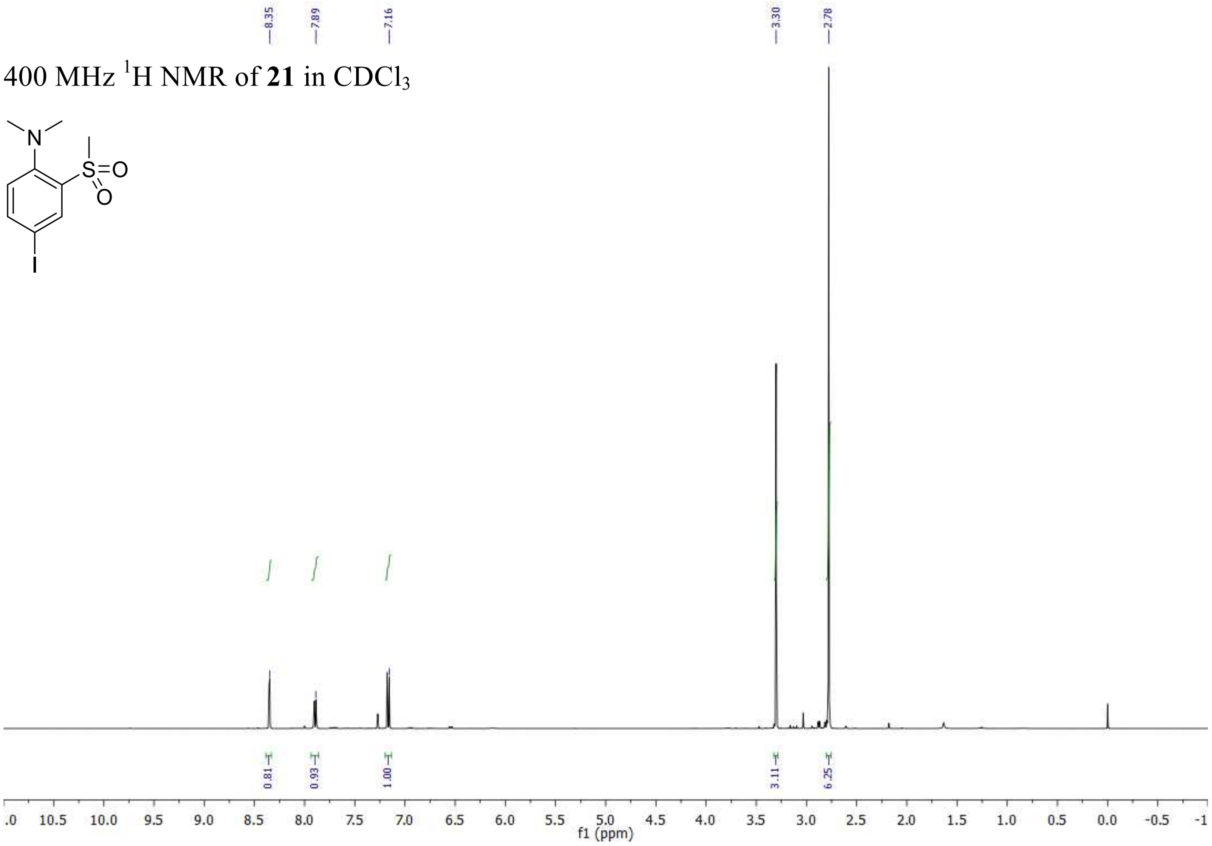

100 MHz ^{13}C NMR of **21** in $CDCl_3$

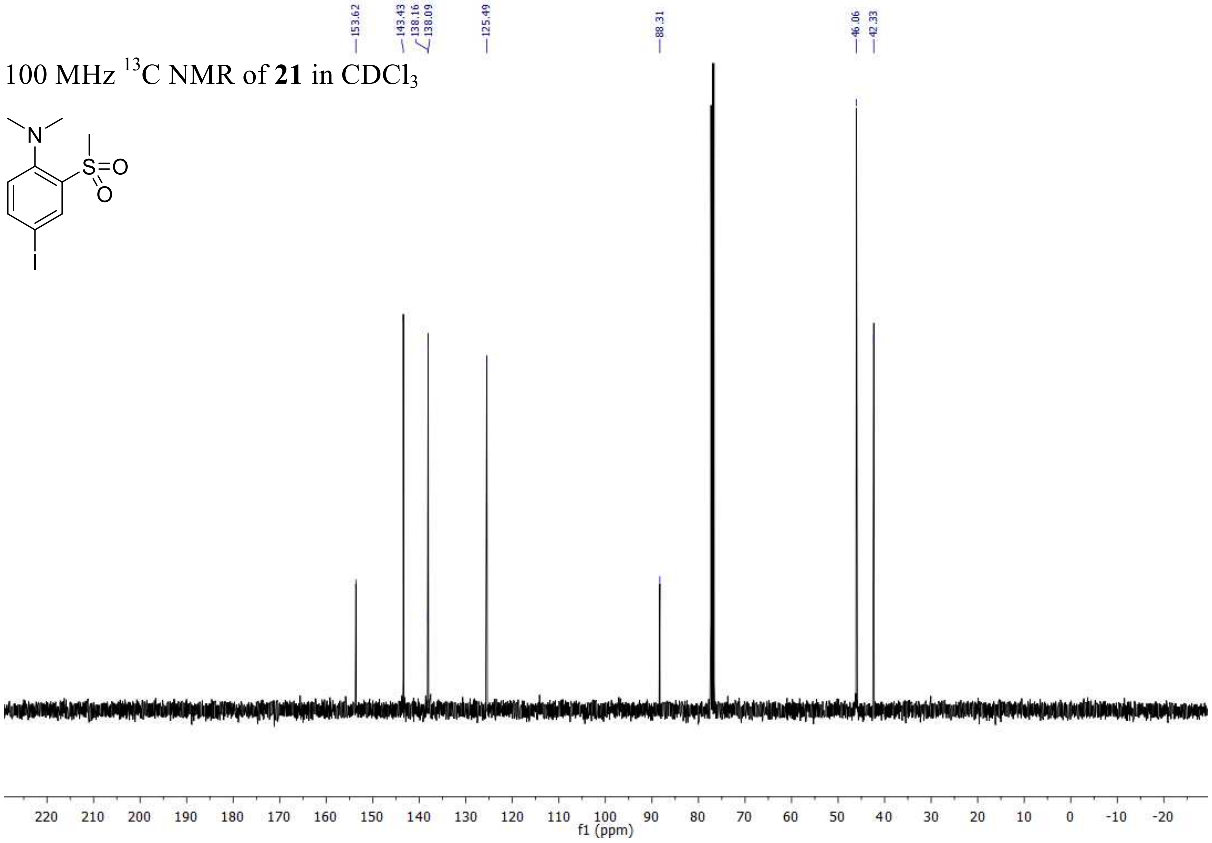

400 MHz ^{1}H NMR of **22** in $CDCl_3$

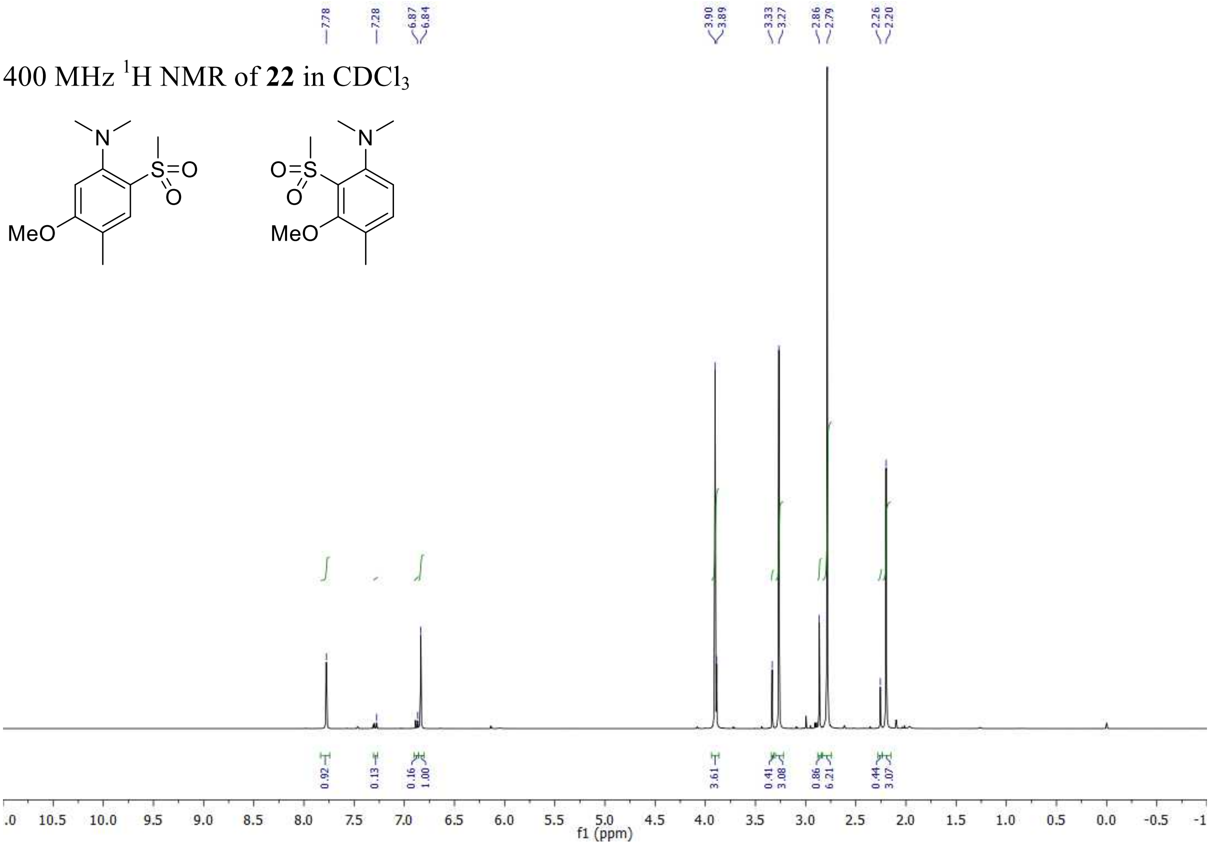

100 MHz ^{13}C NMR of **22** in $CDCl_3$

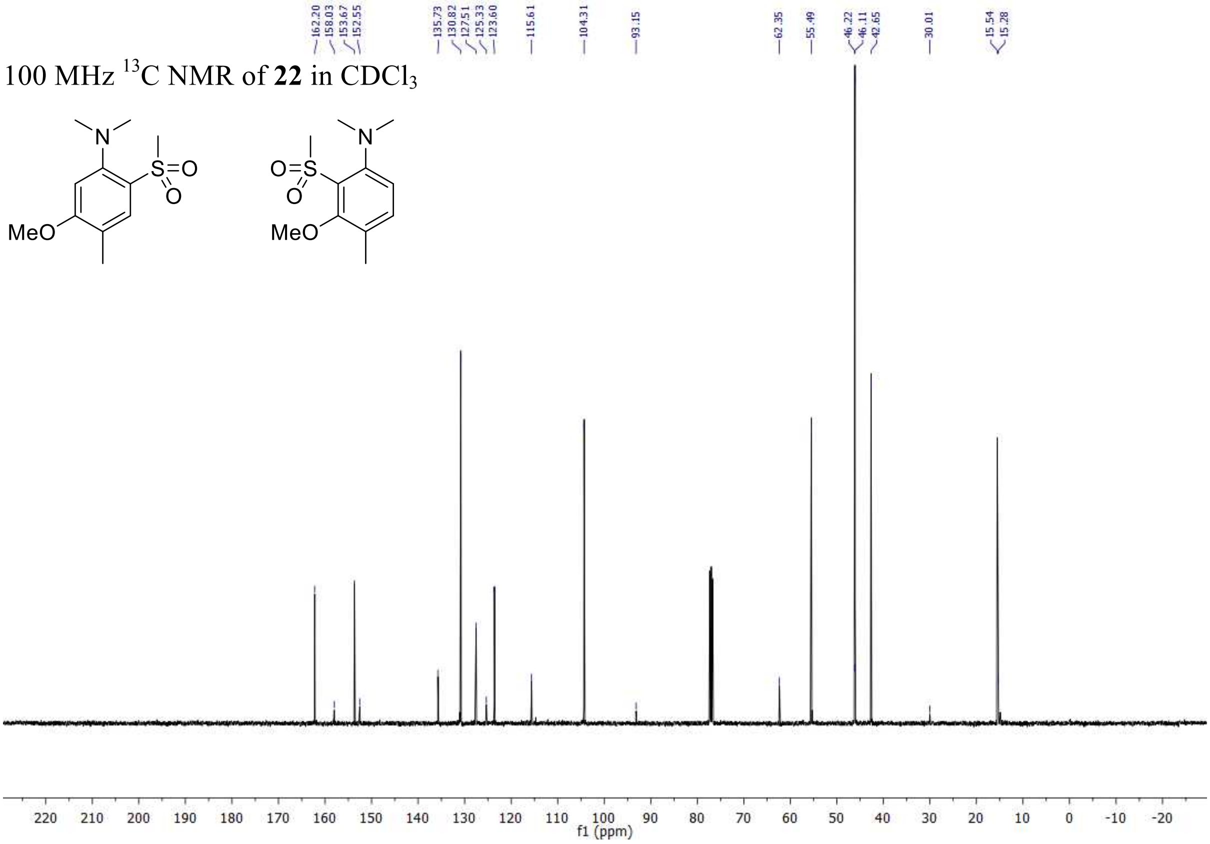

400 MHz ^{1}H NMR of **23** in $CDCl_3$

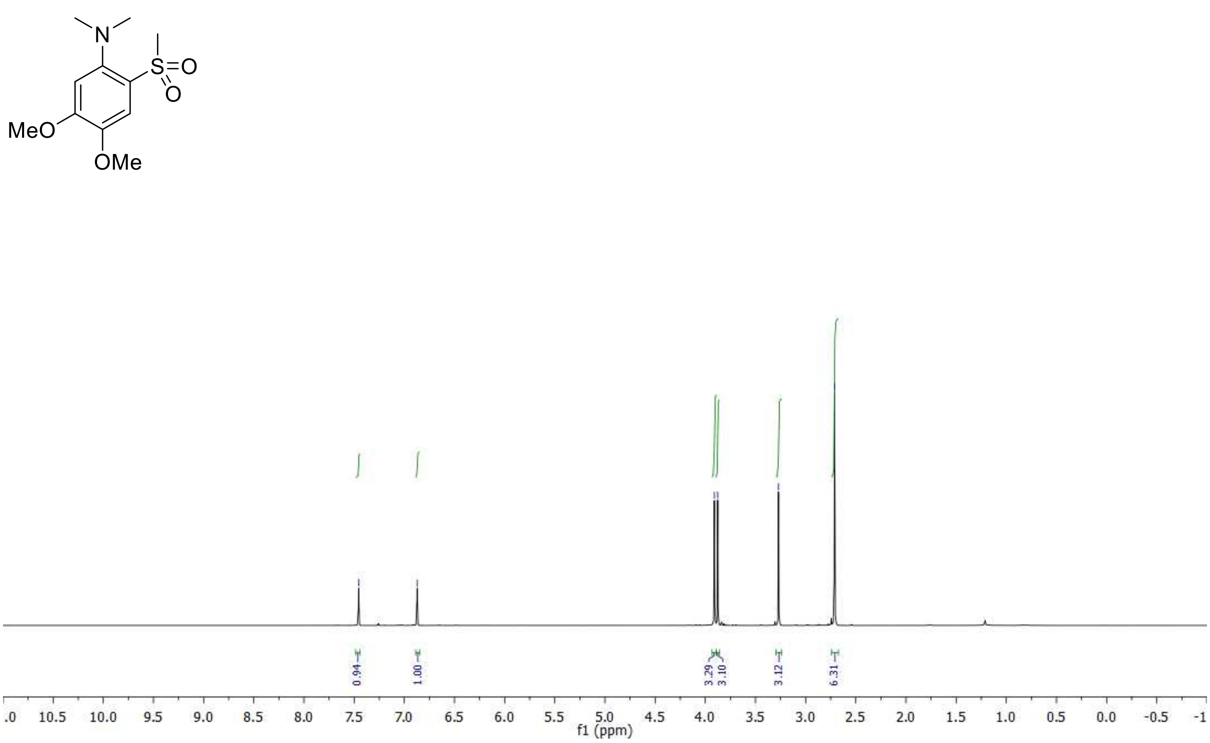

100 MHz ^{13}C NMR of **23** in $CDCl_3$

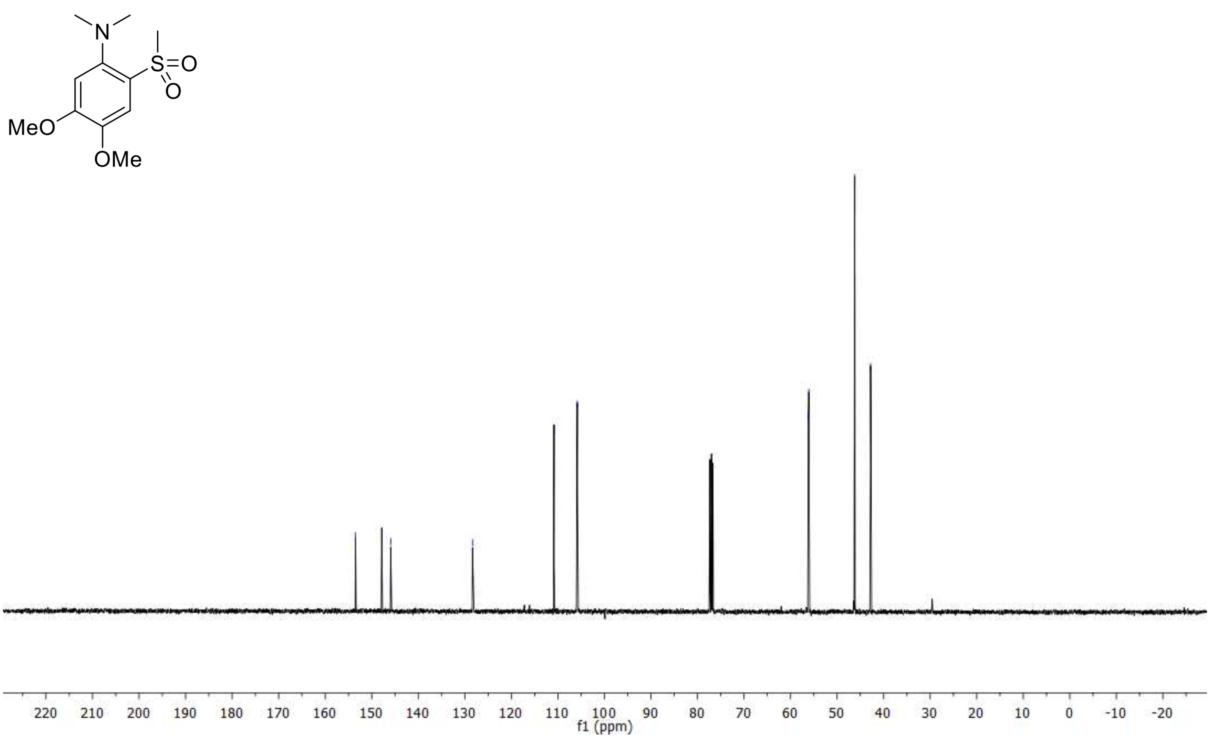

400 MHz ^{1}H NMR of **24a** in $CDCl_3$

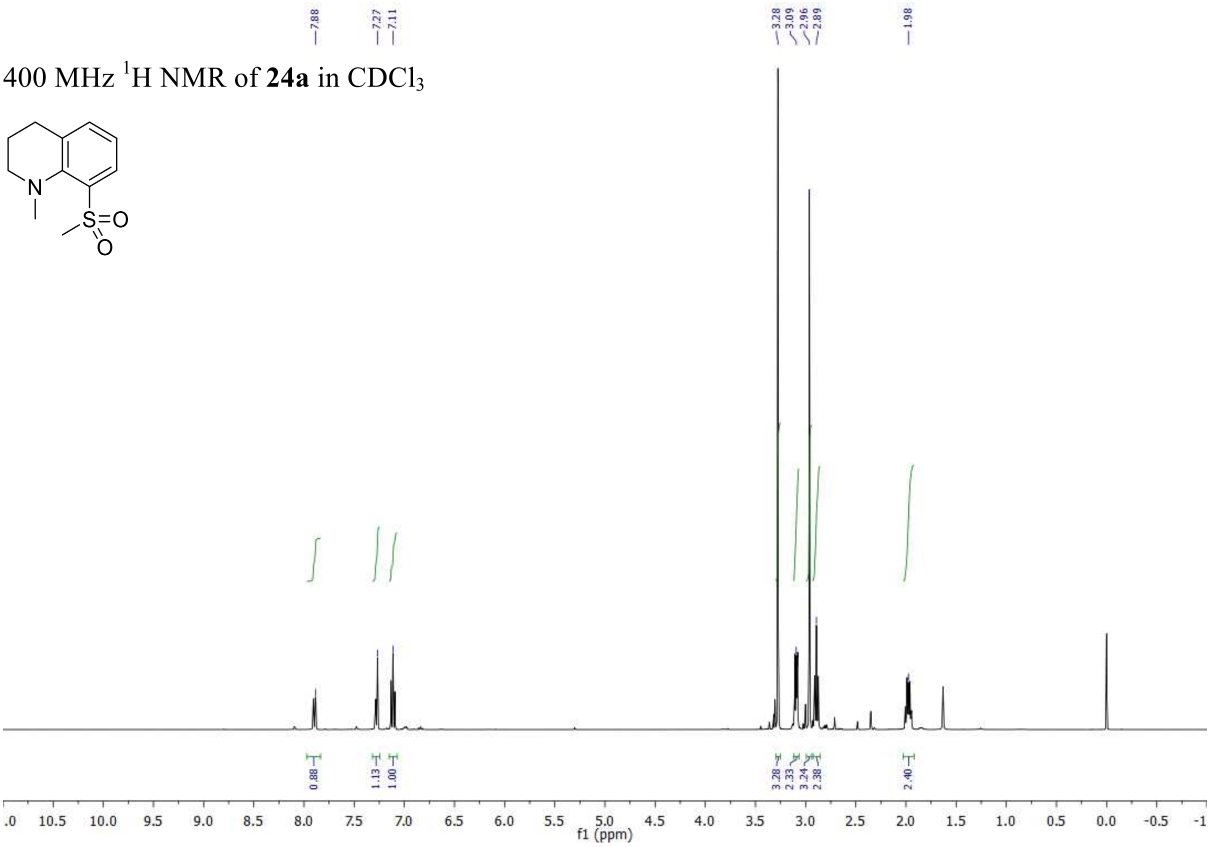

125 MHz ^{13}C NMR of **24a** in $CDCl_3$

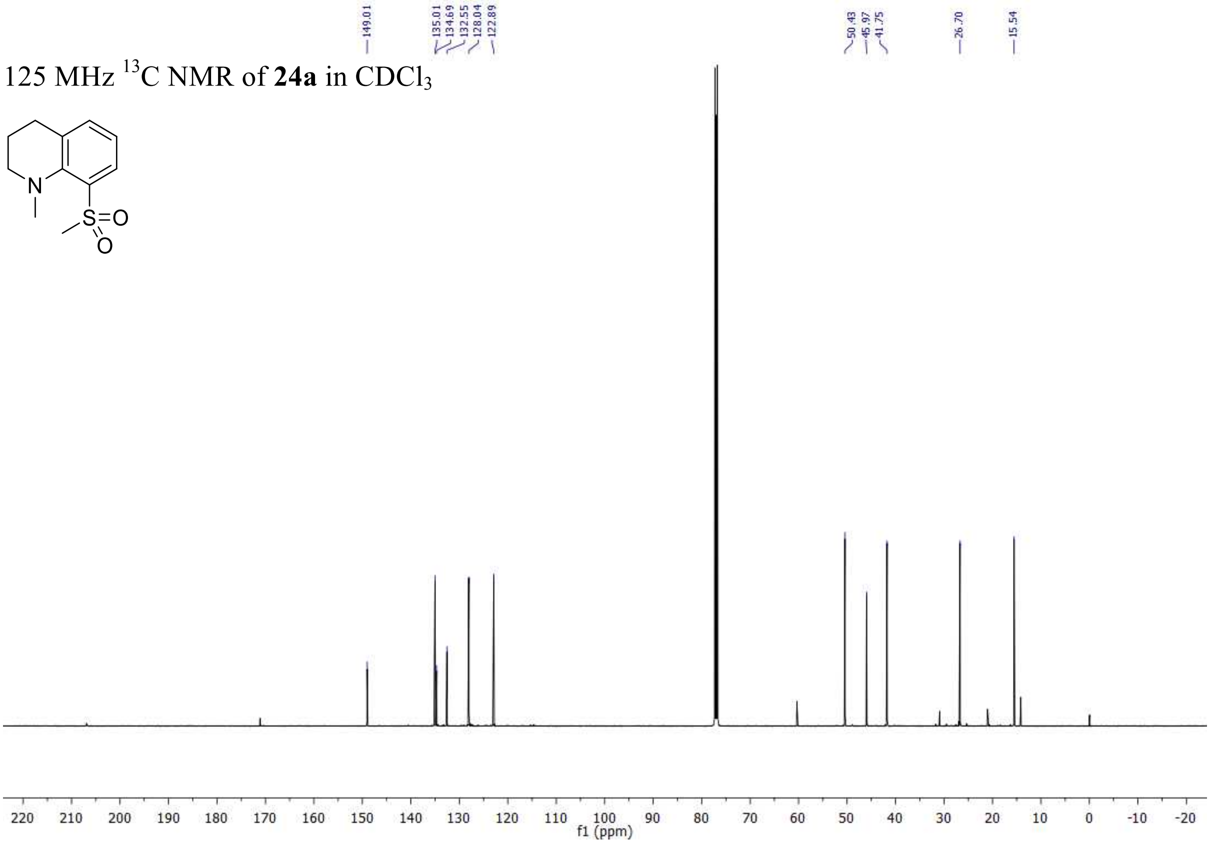

400 MHz ^{1}H NMR of **24b** in $CDCl_3$

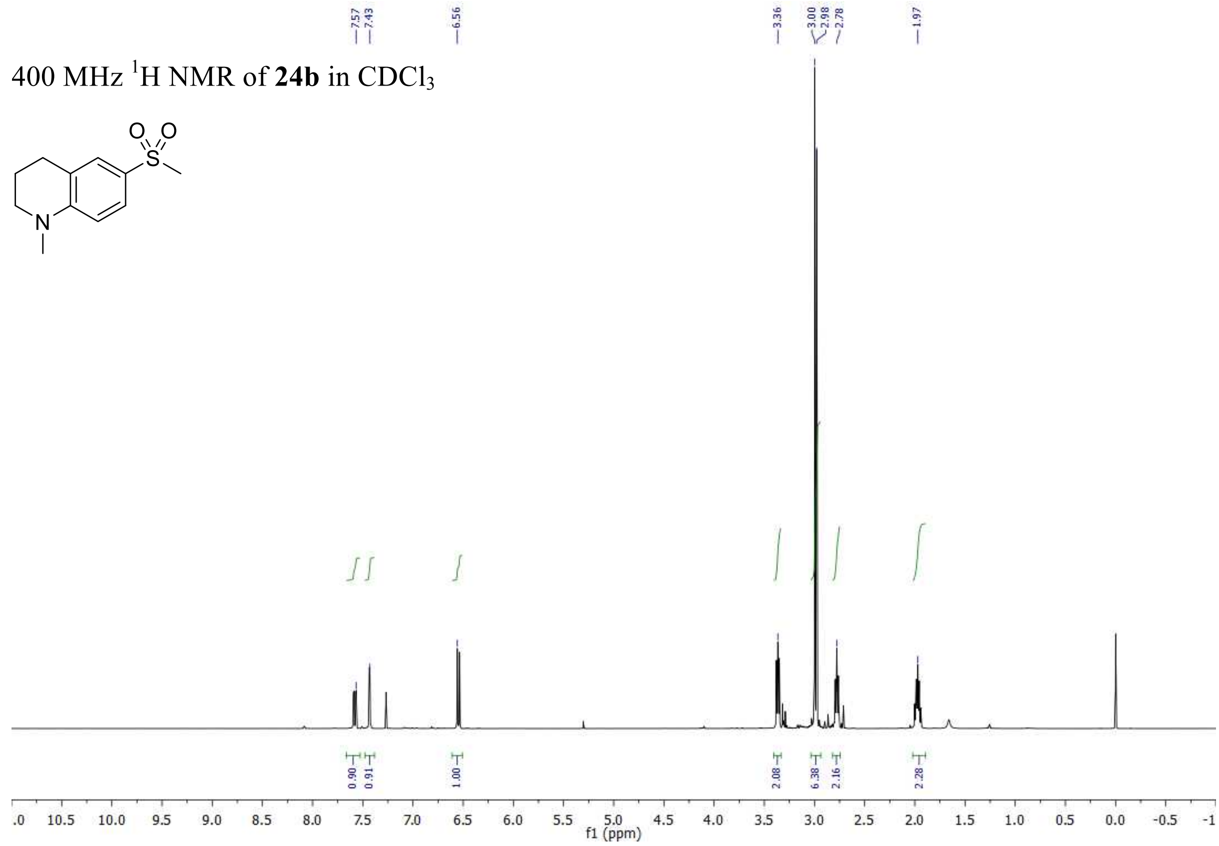

125 MHz ^{13}C NMR of **24b** in $CDCl_3$

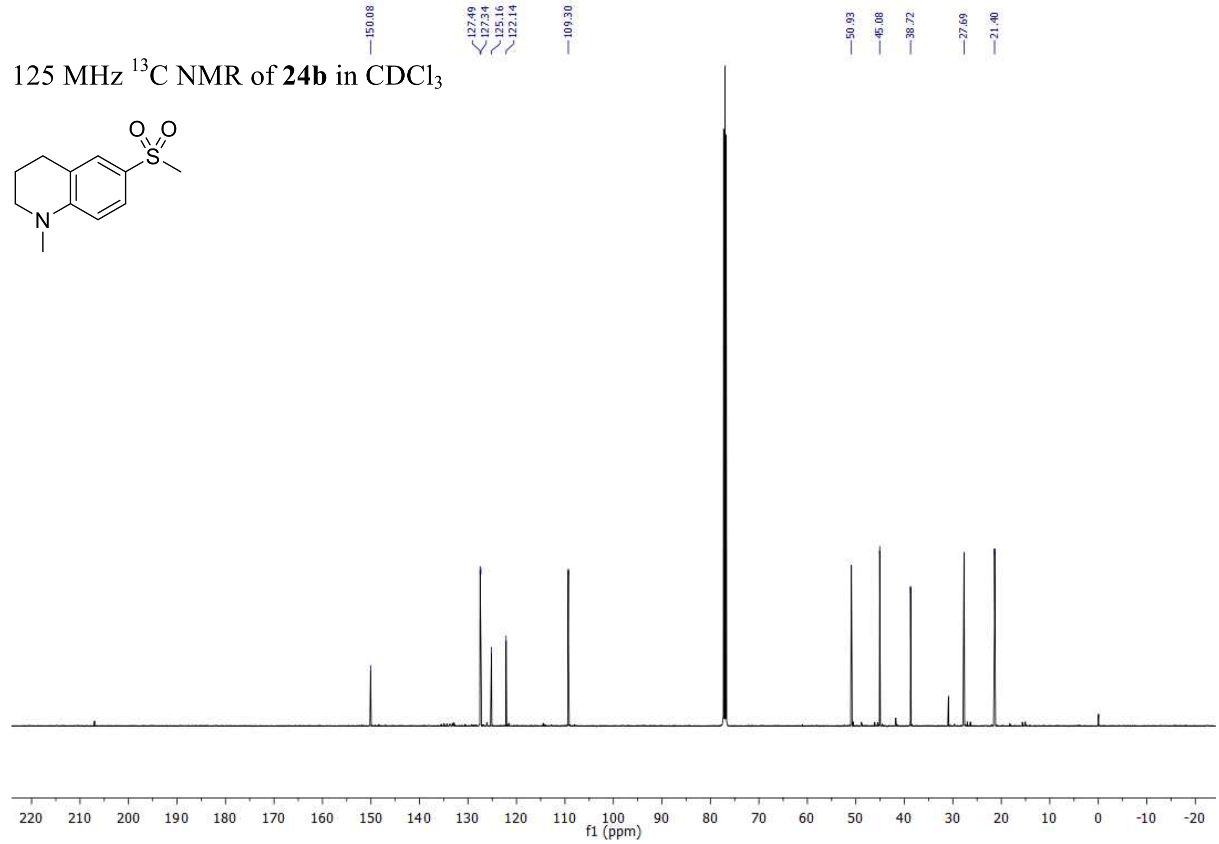

400 MHz ^{1}H NMR of **25a** in $CDCl_3$

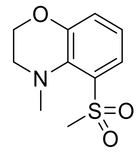

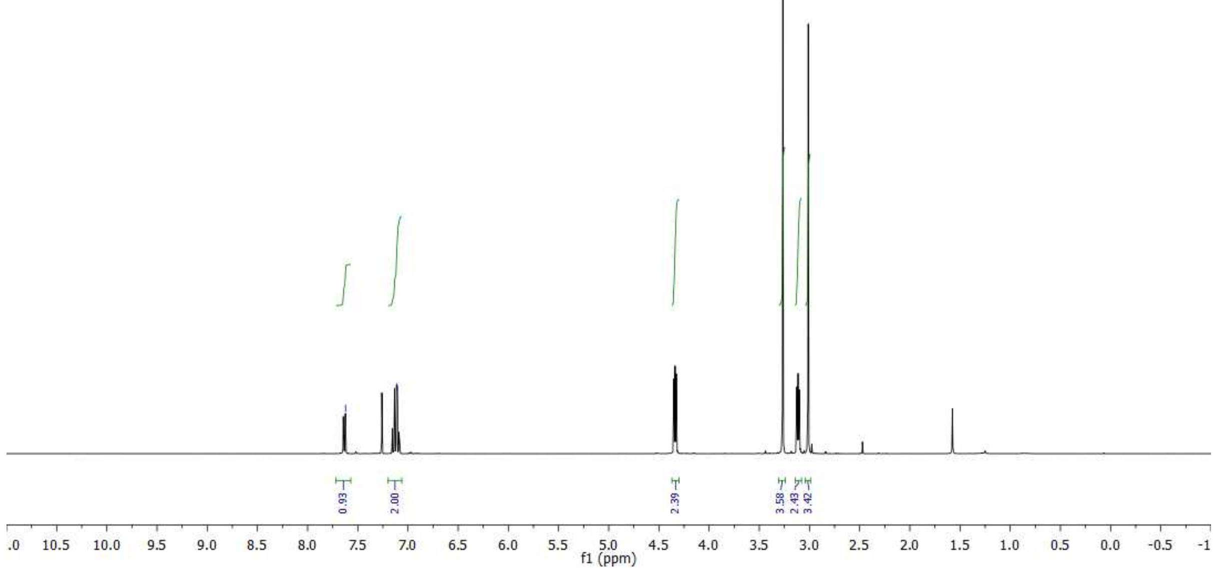

100 MHz ^{13}C NMR of **25a** in $CDCl_3$

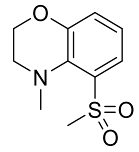

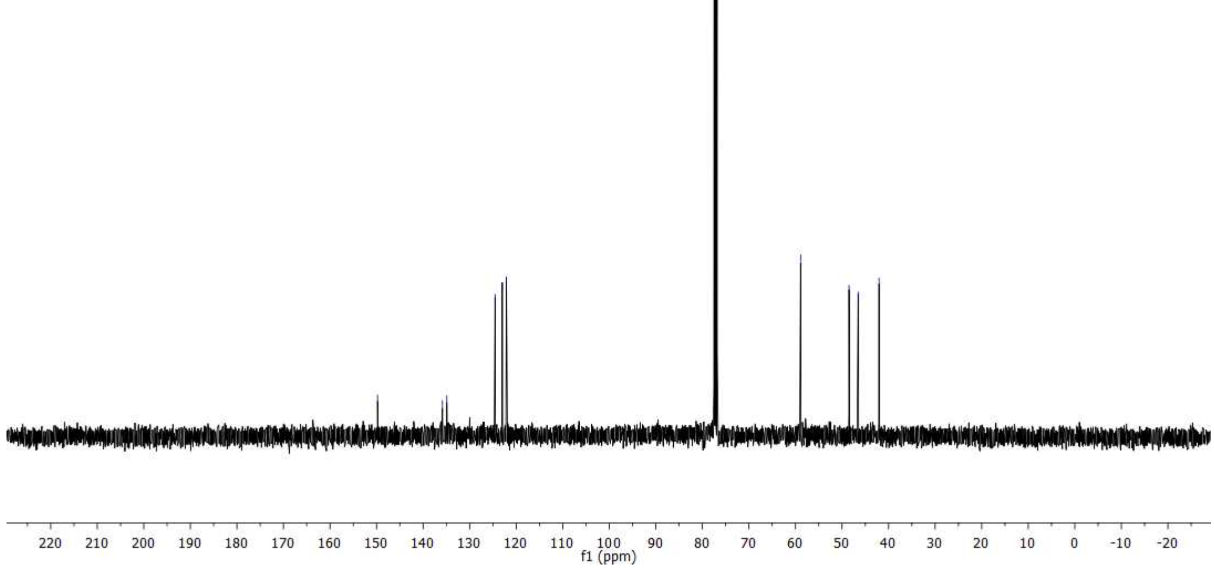

400 MHz ^{1}H NMR of **25b** and **25c** in $CDCl_3$

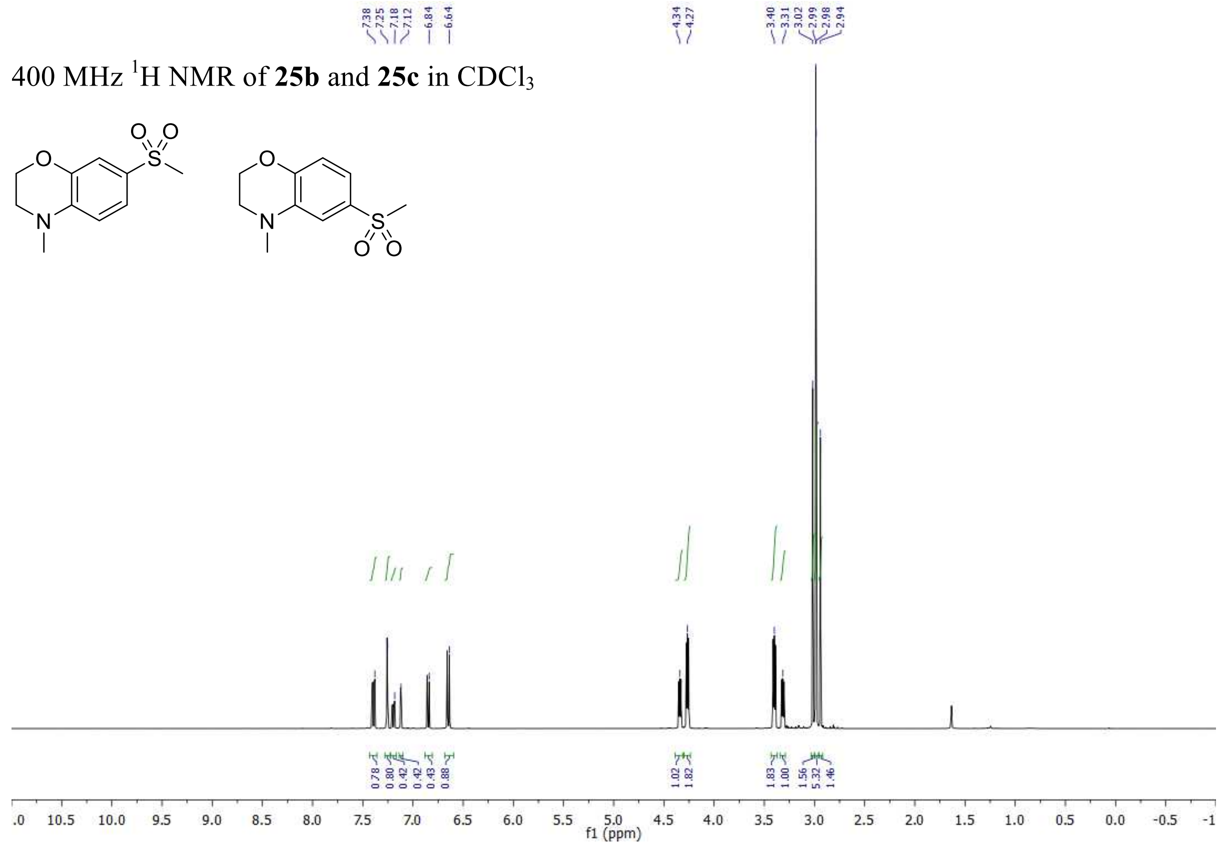

100 MHz ^{13}C NMR of **25b** and **25c** in $CDCl_3$

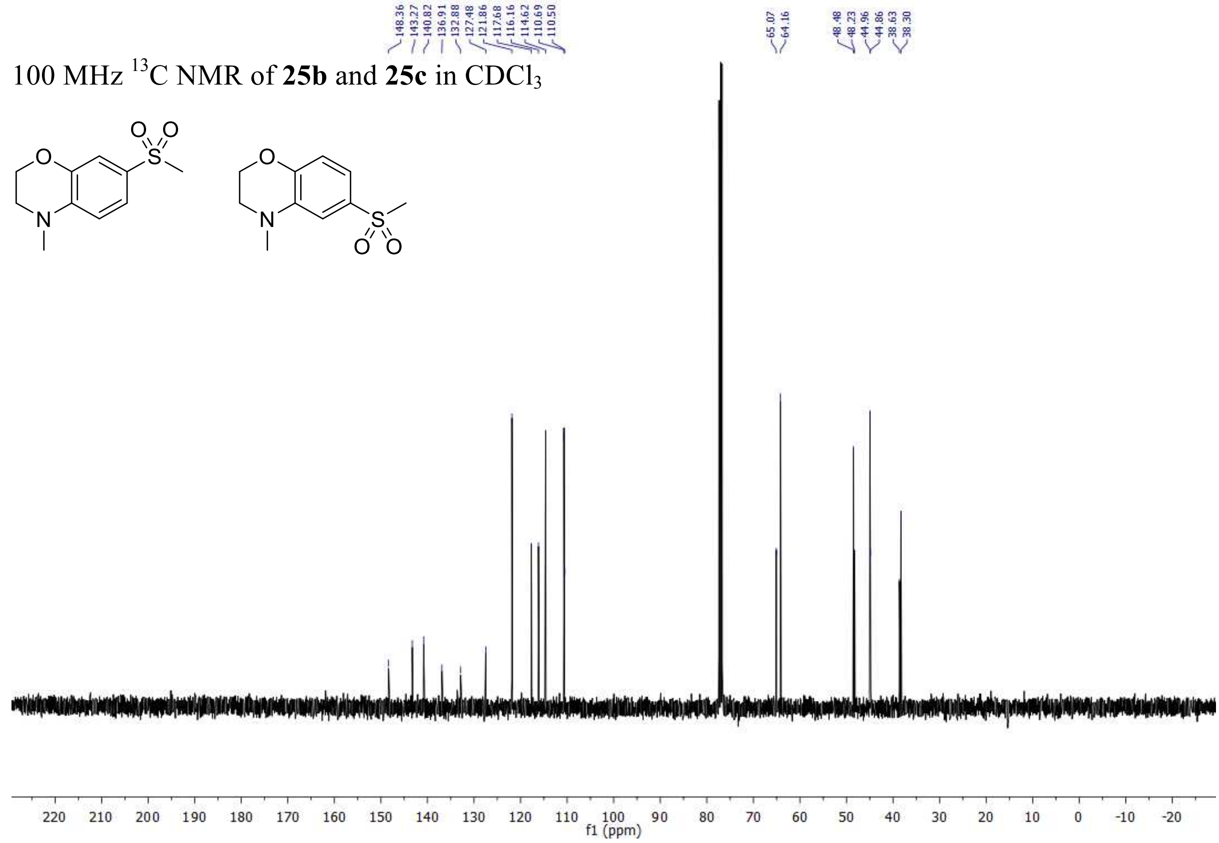

400 MHz ^{1}H NMR of **26** in $CDCl_3$

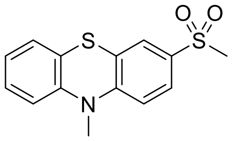

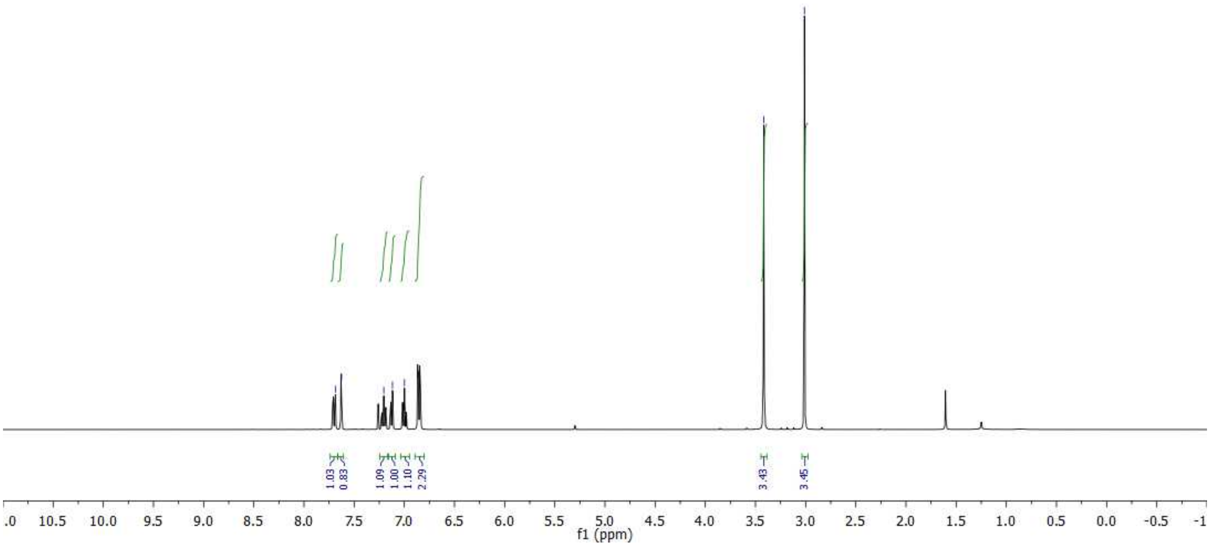

100 MHz ^{13}C NMR of **26** in $CDCl_3$

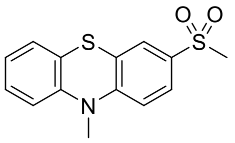

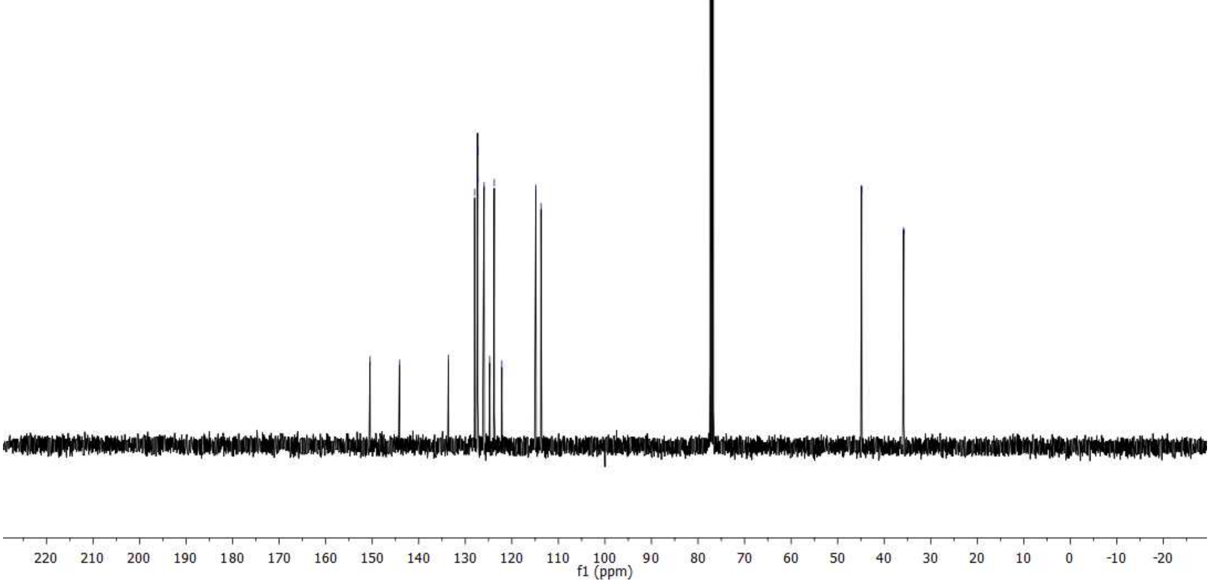

400 MHz ^{1}H NMR of **27a** in $CDCl_3$

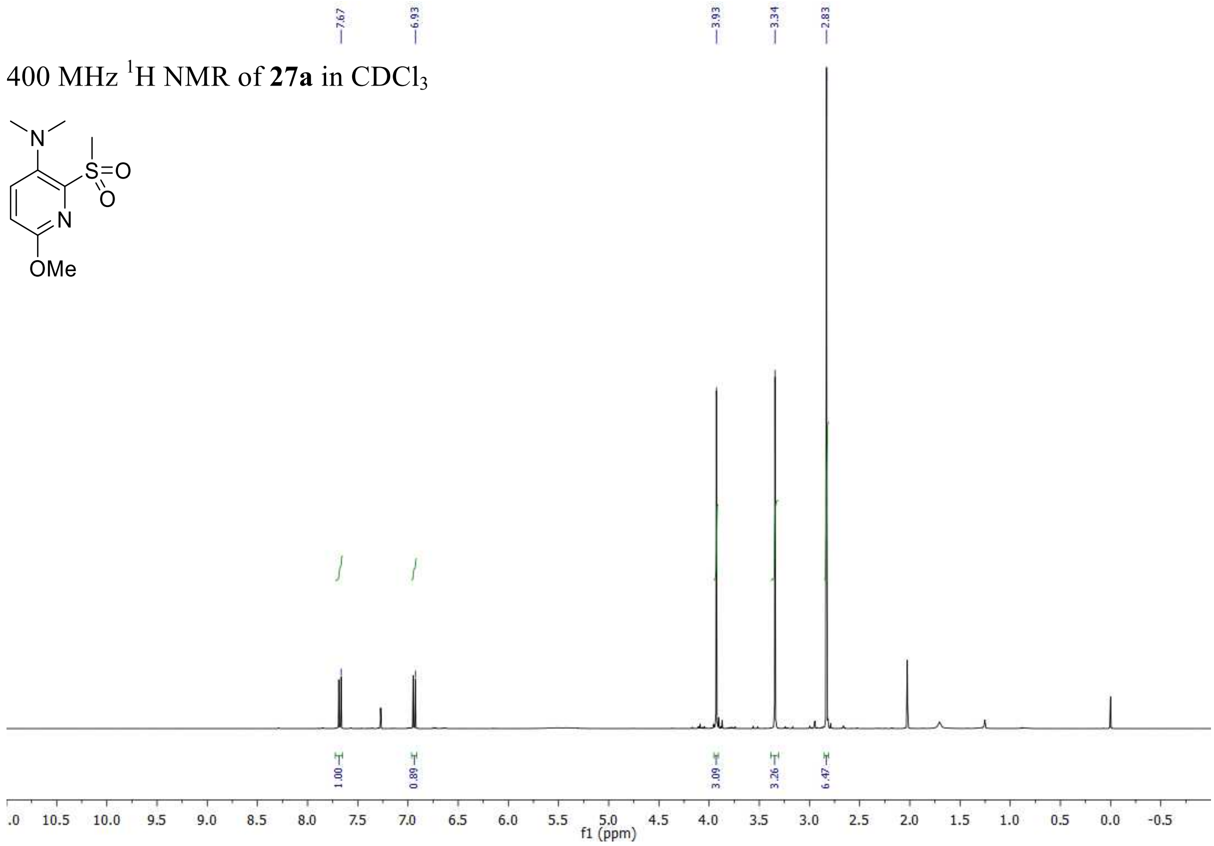

125 MHz ^{13}C NMR of **27a** in $CDCl_3$

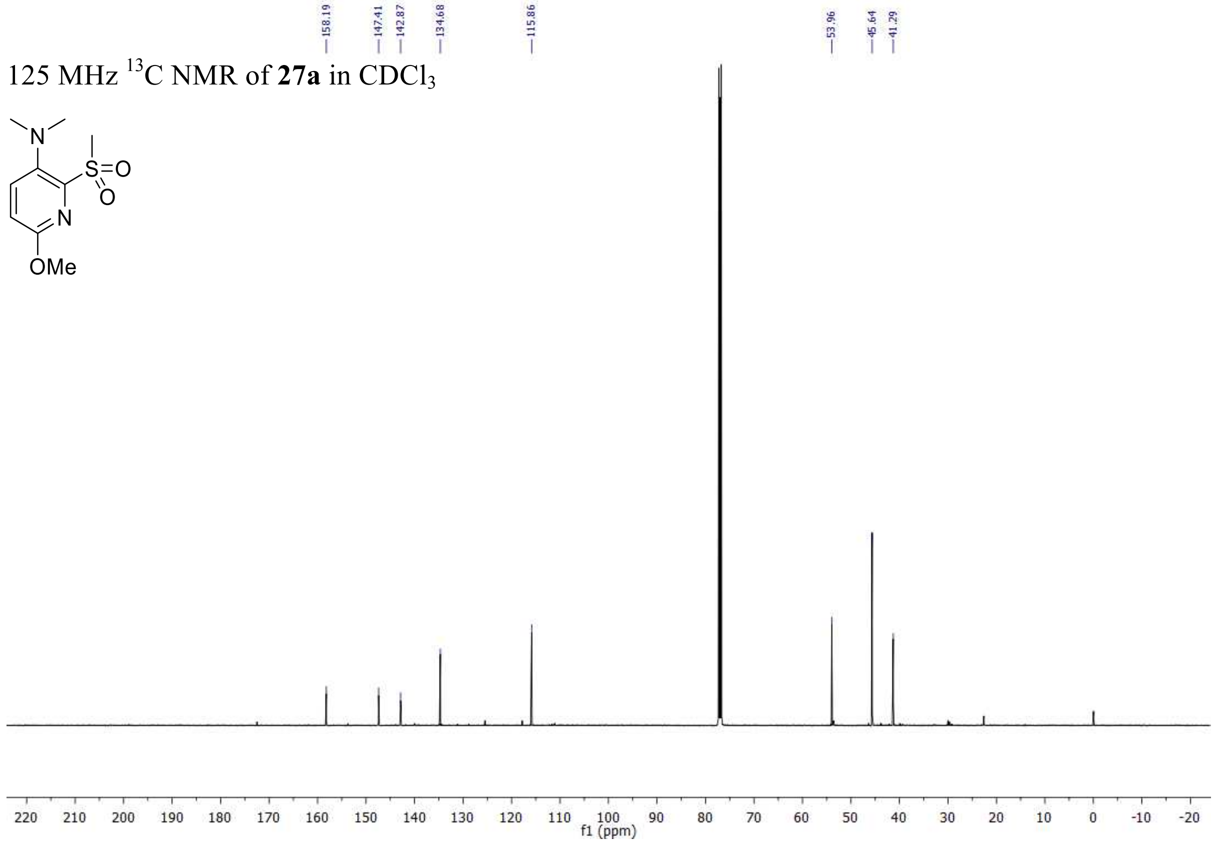

400 MHz ^{1}H NMR of **27b** in $CDCl_3$

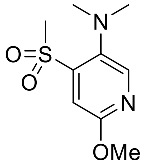

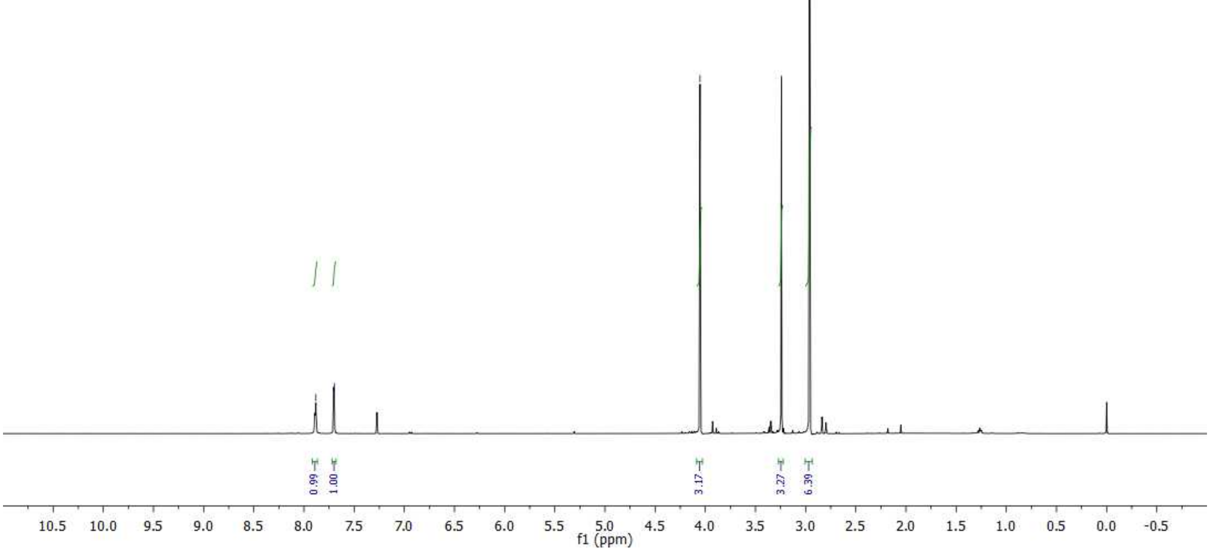

125 MHz ^{13}C NMR of **27b** in $CDCl_3$

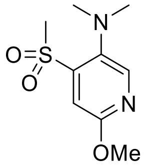

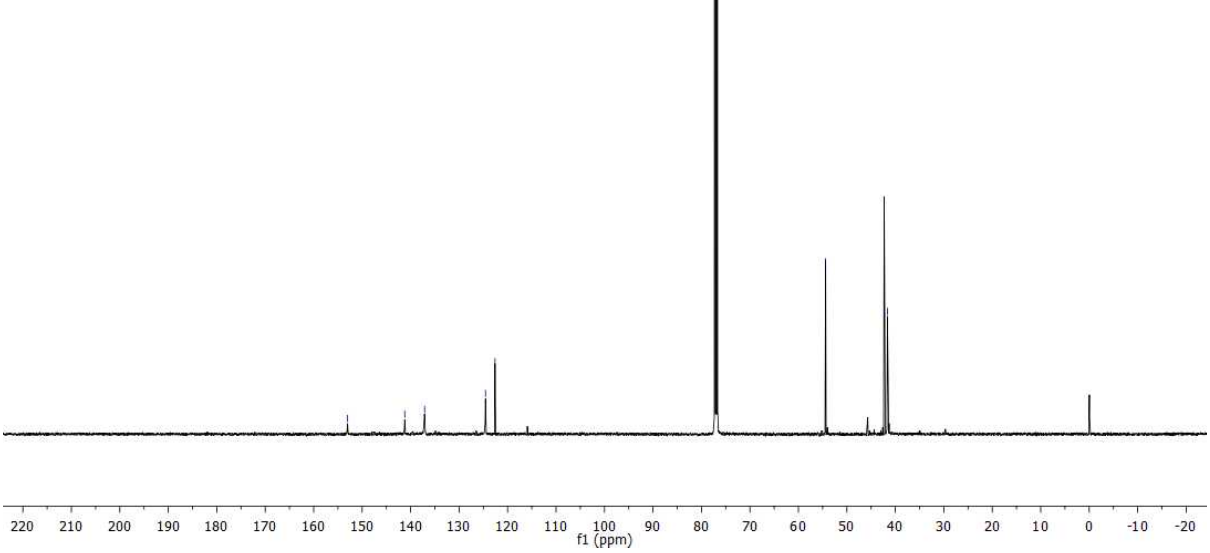

500 MHz ^{1}H NMR of **29a** in $CDCl_3$

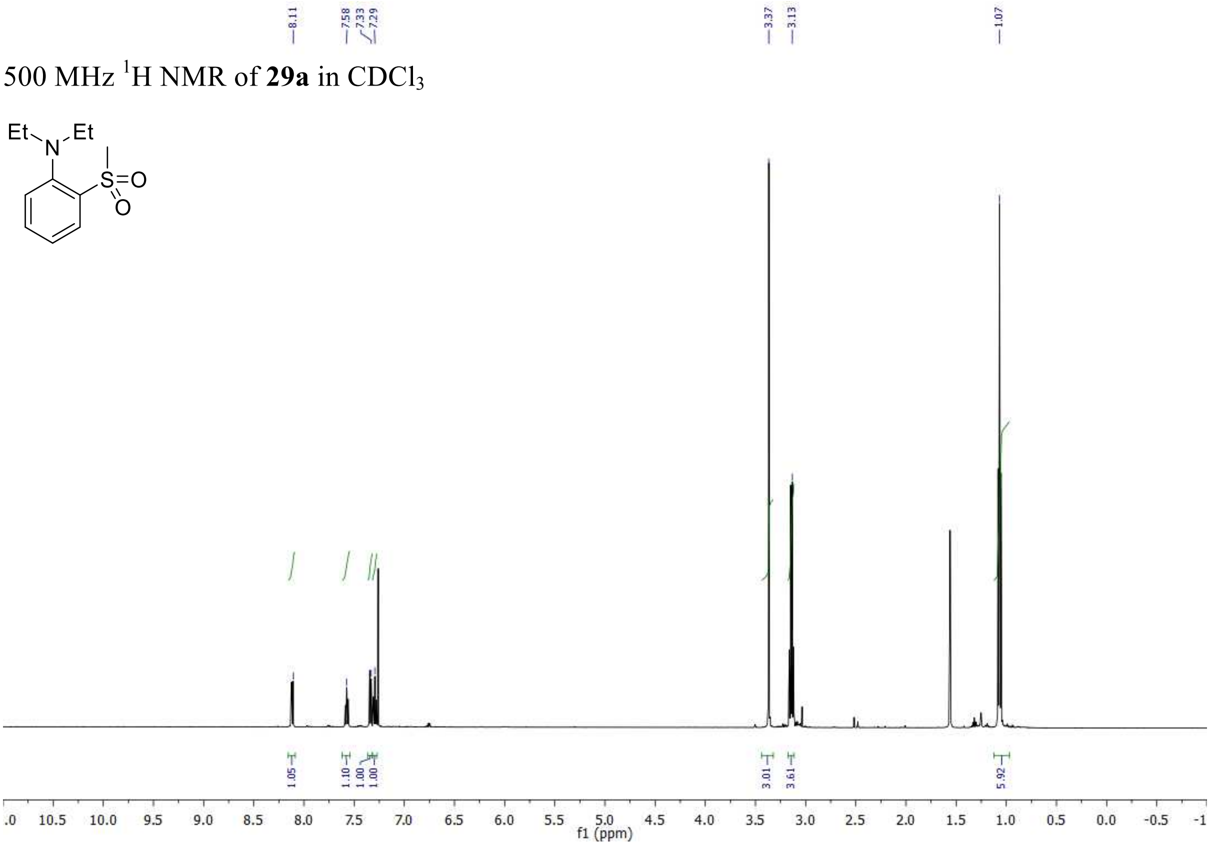

125 MHz ^{13}C NMR of **29a** in $CDCl_3$

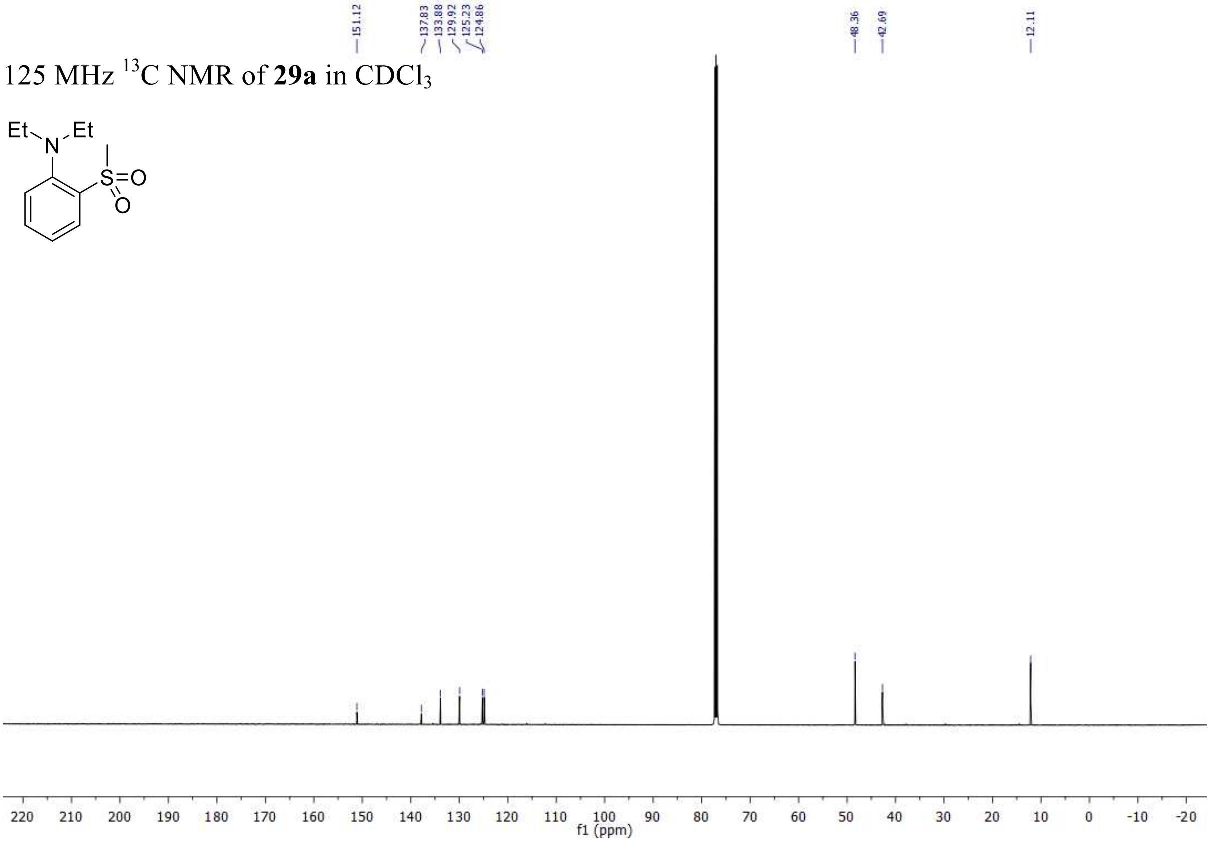

500 MHz ^{1}H NMR of **29b** in $CDCl_3$

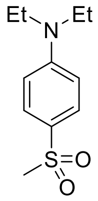

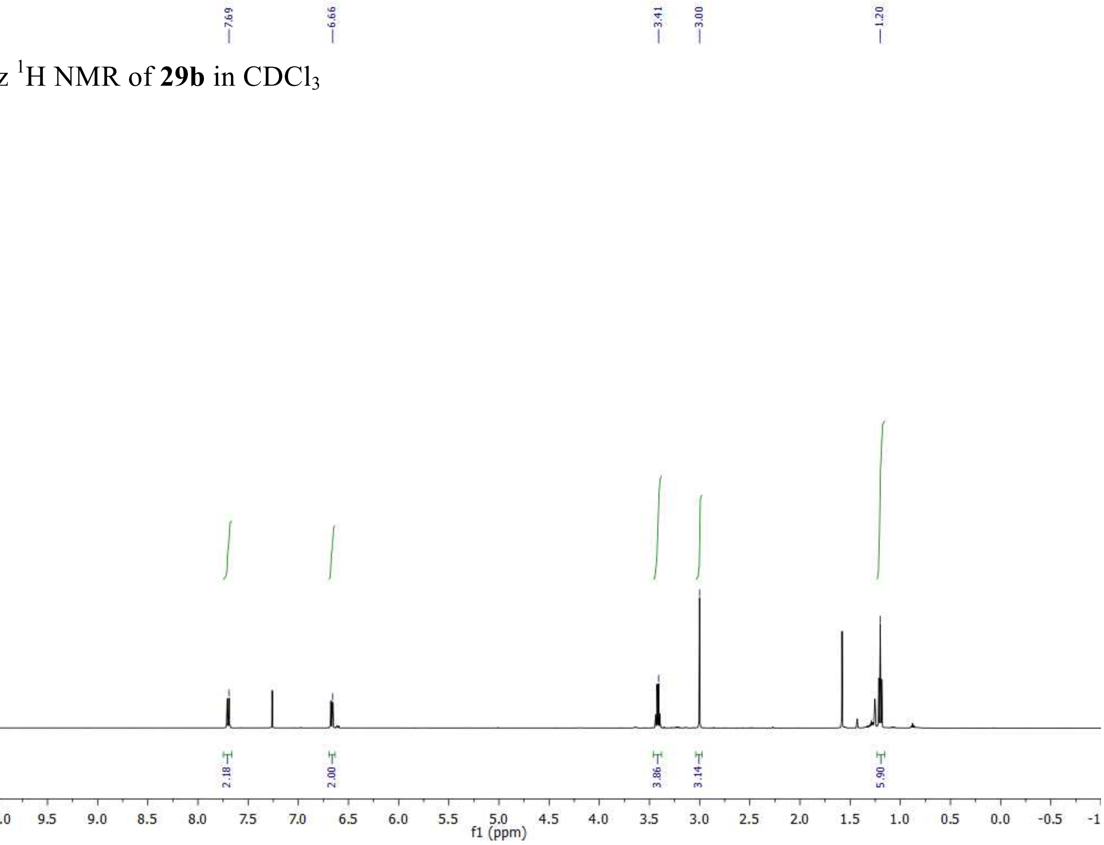

125 MHz ^{13}C NMR of **29b** in $CDCl_3$

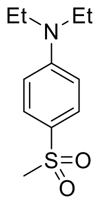

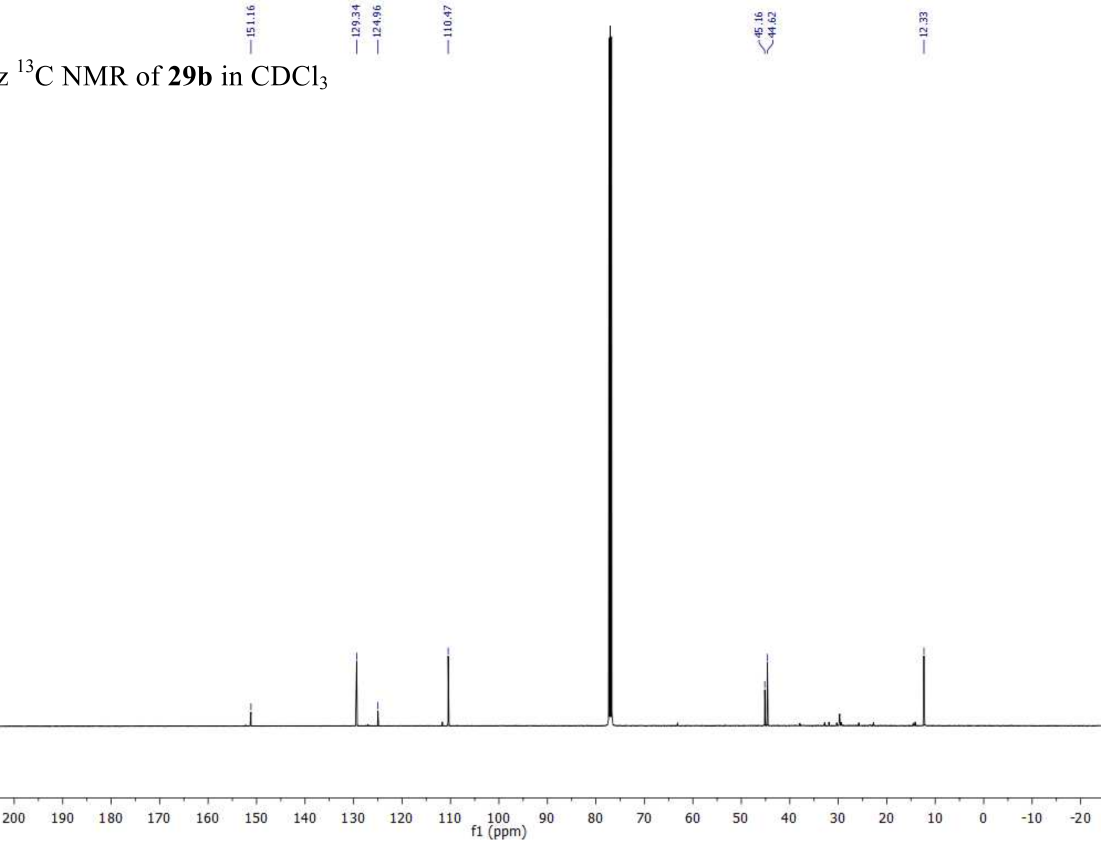

500 MHz ^1H NMR of **30a** in $CDCl_3$

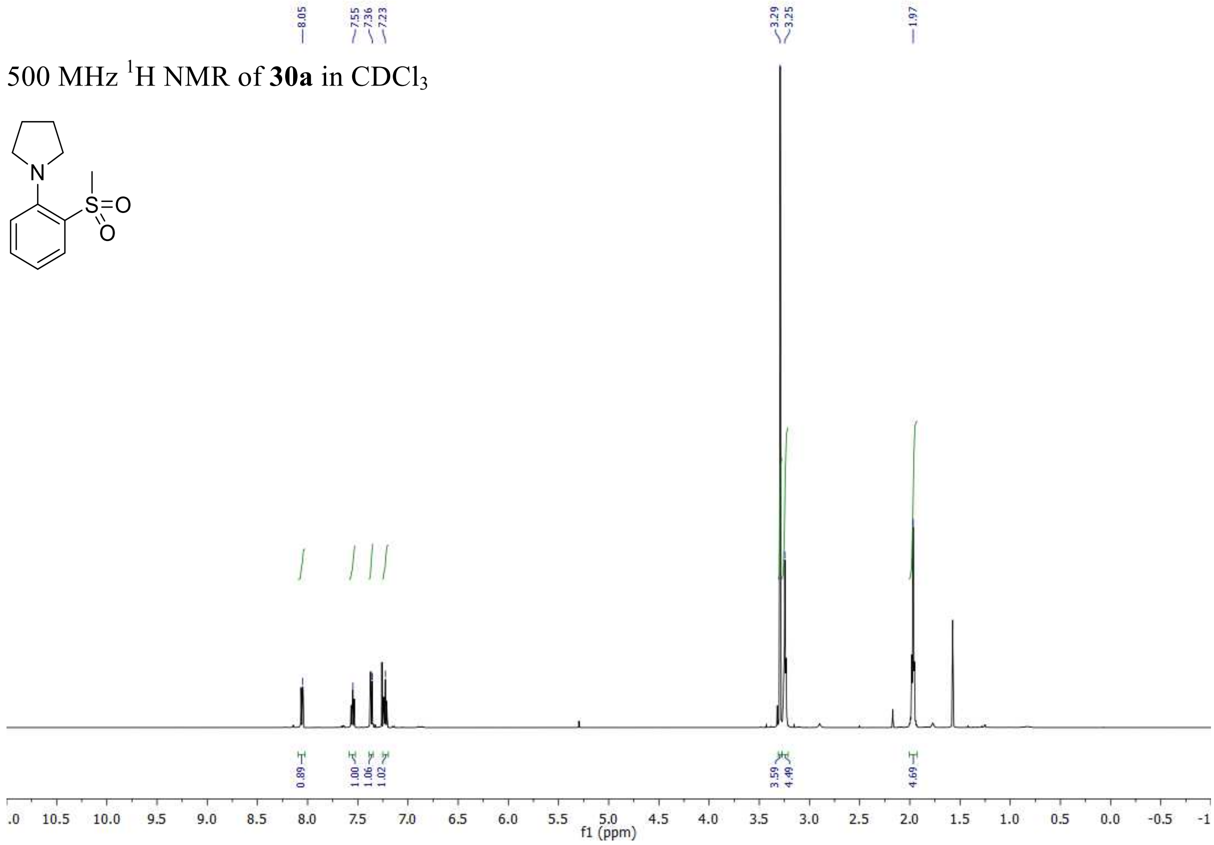

125 MHz ^{13}C NMR of **30a** in $CDCl_3$

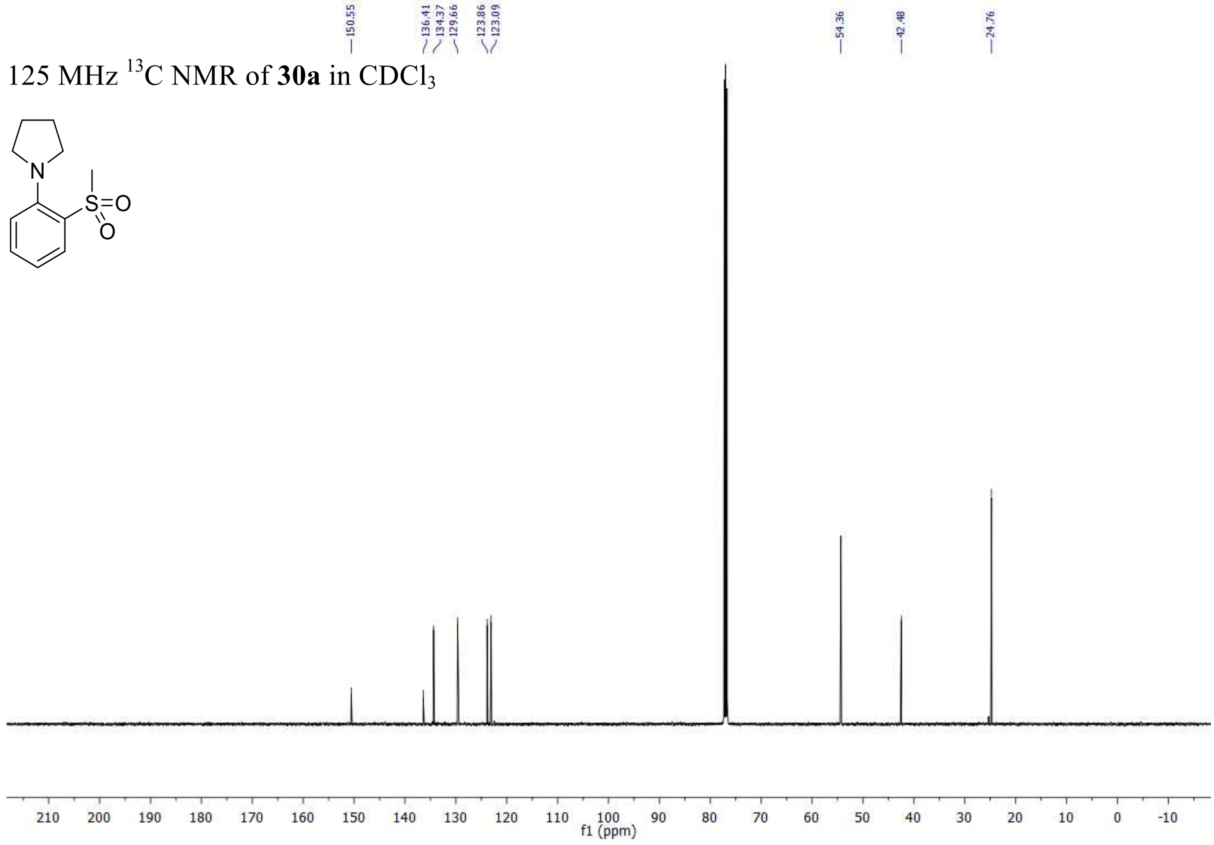

500 MHz ^{1}H NMR of **30b** in $CDCl_3$

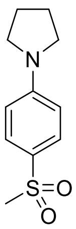

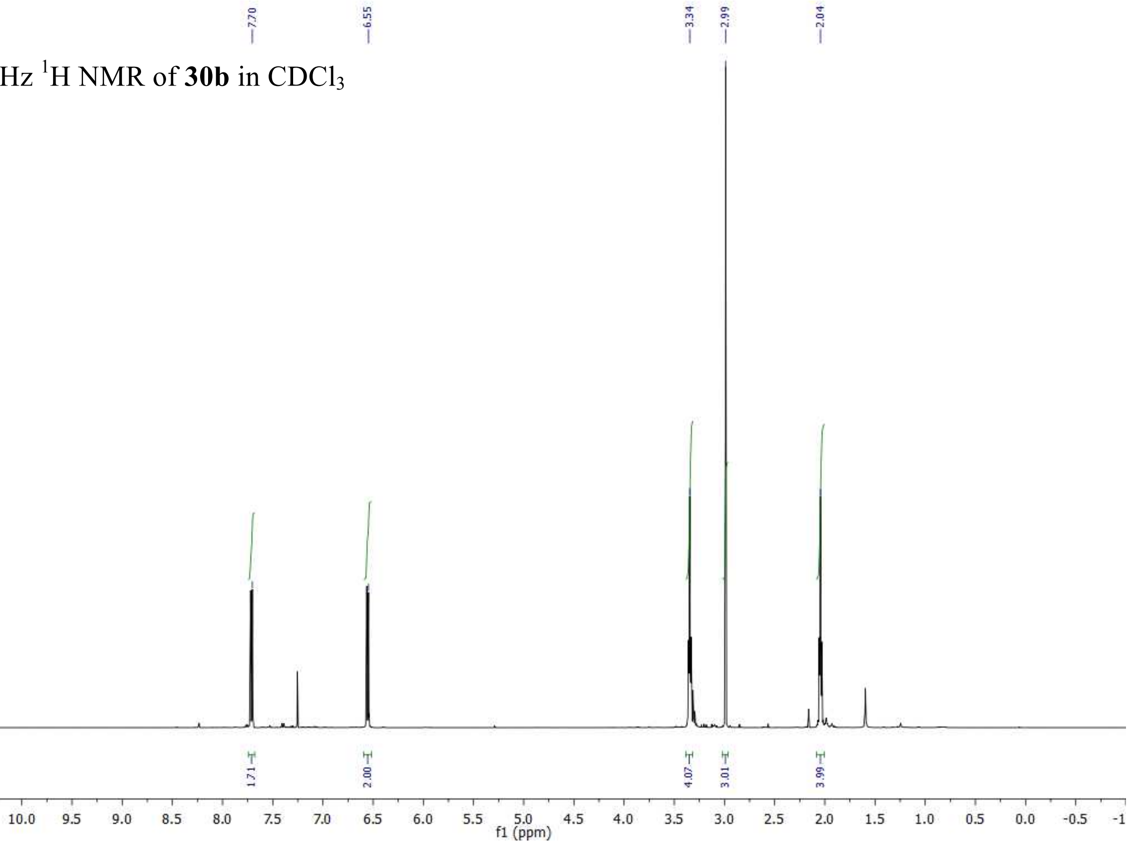

125 MHz ^{13}C NMR of **30b** in $CDCl_3$

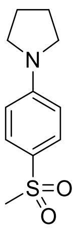

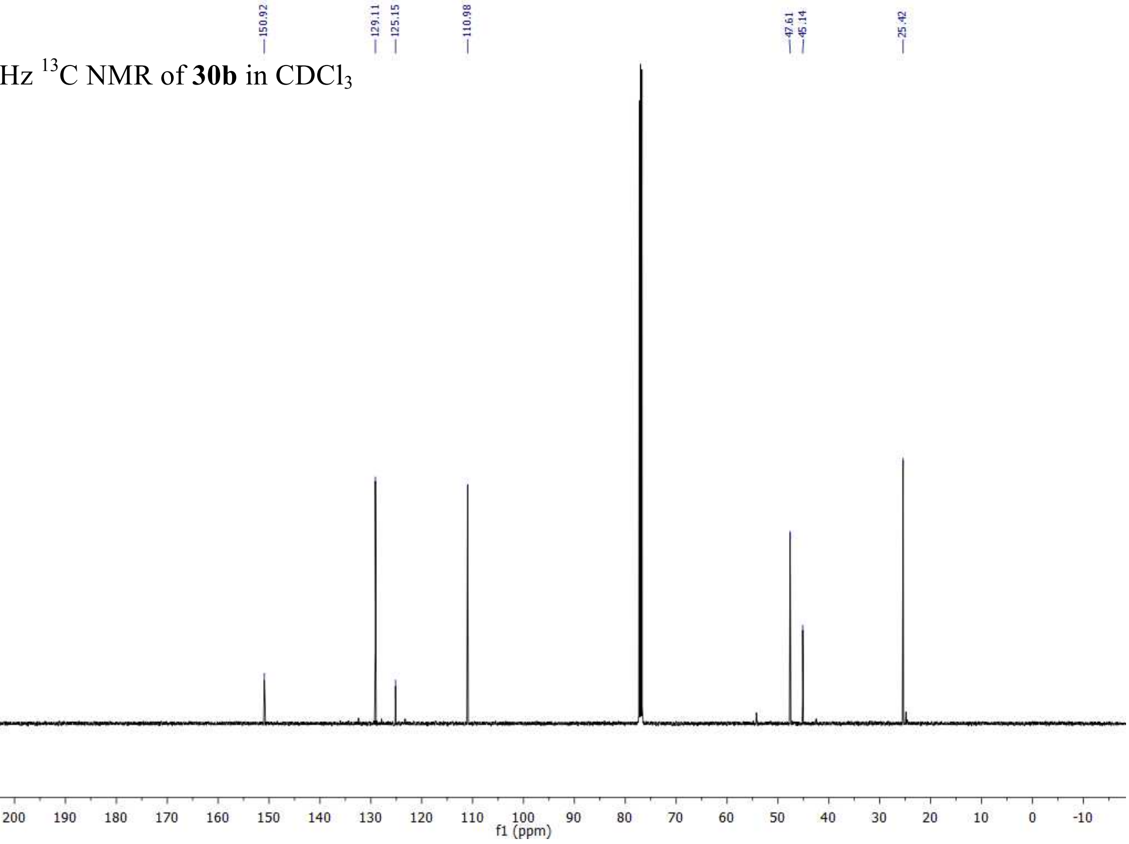

500 MHz ¹H NMR of **31a** in $CDCl_3$

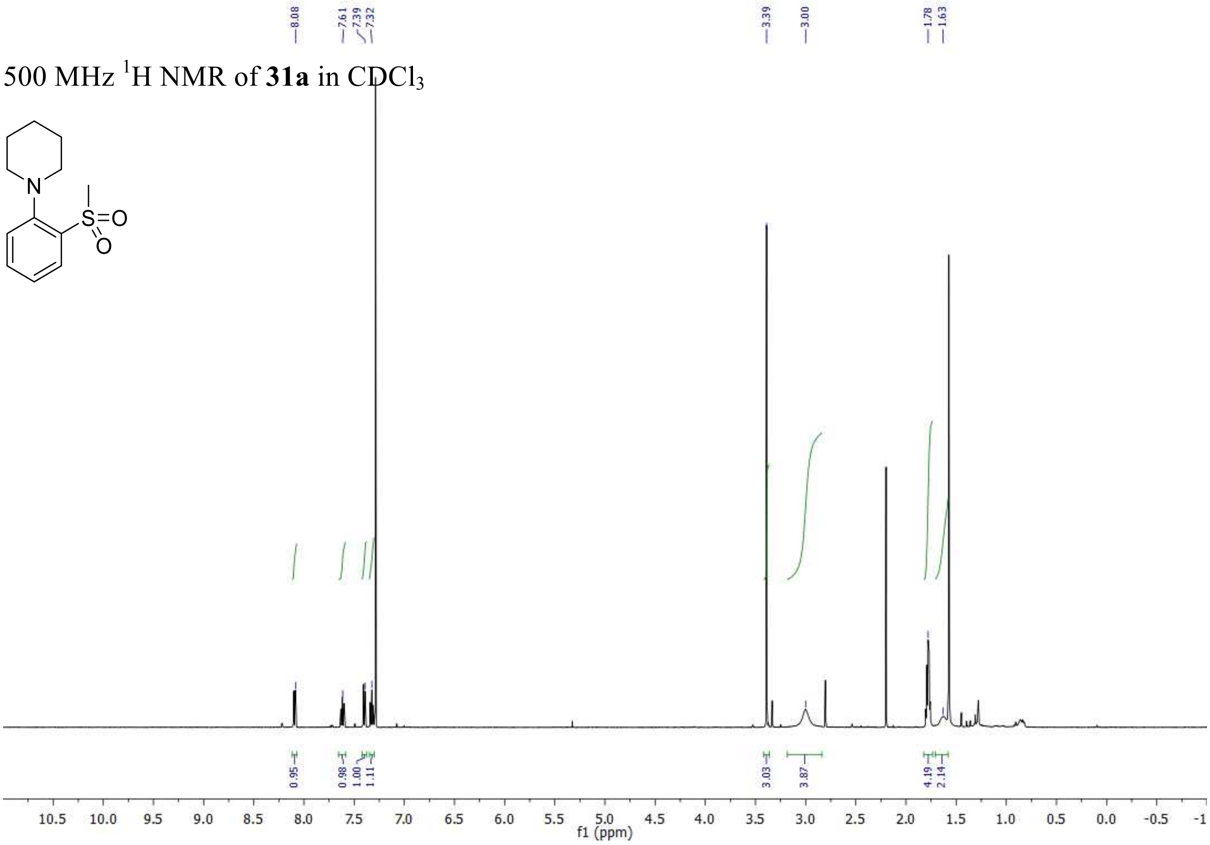

125 MHz ¹³C NMR of **31a** in $CDCl_3$

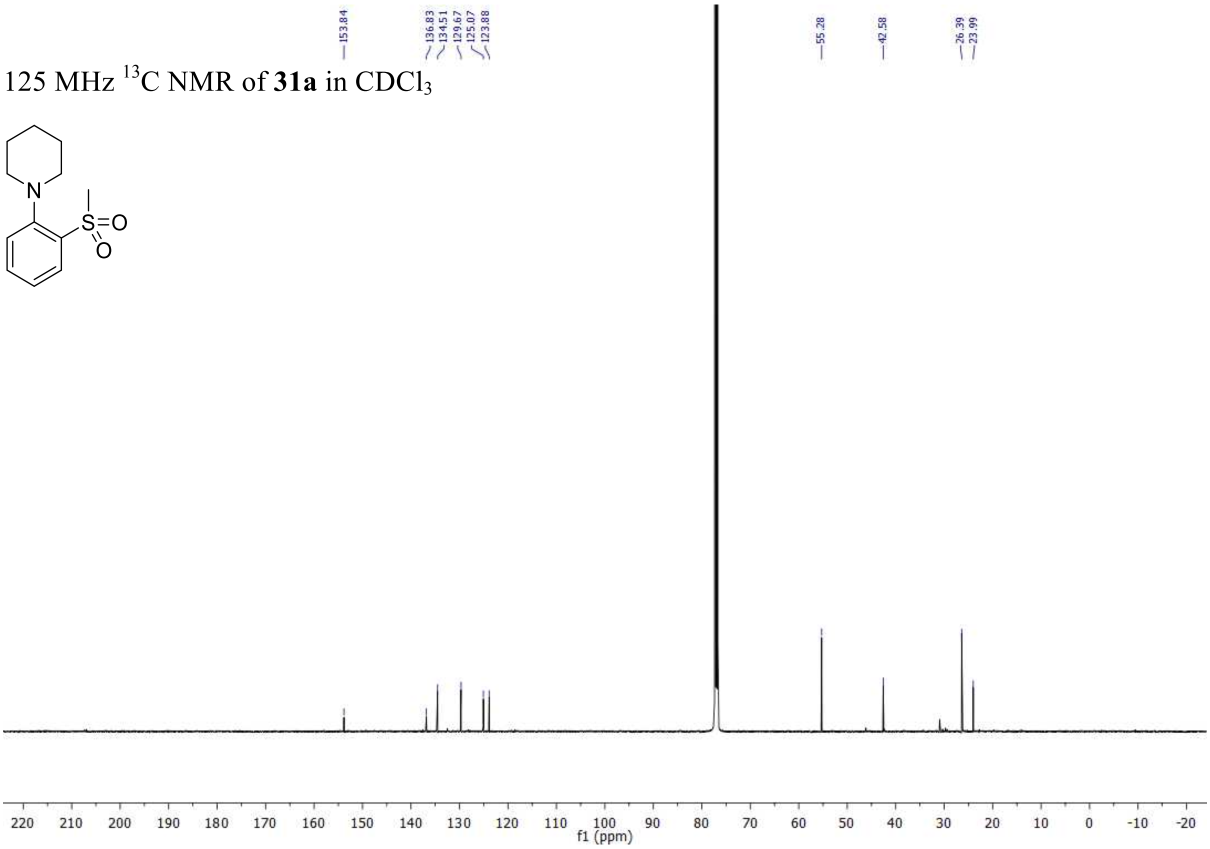

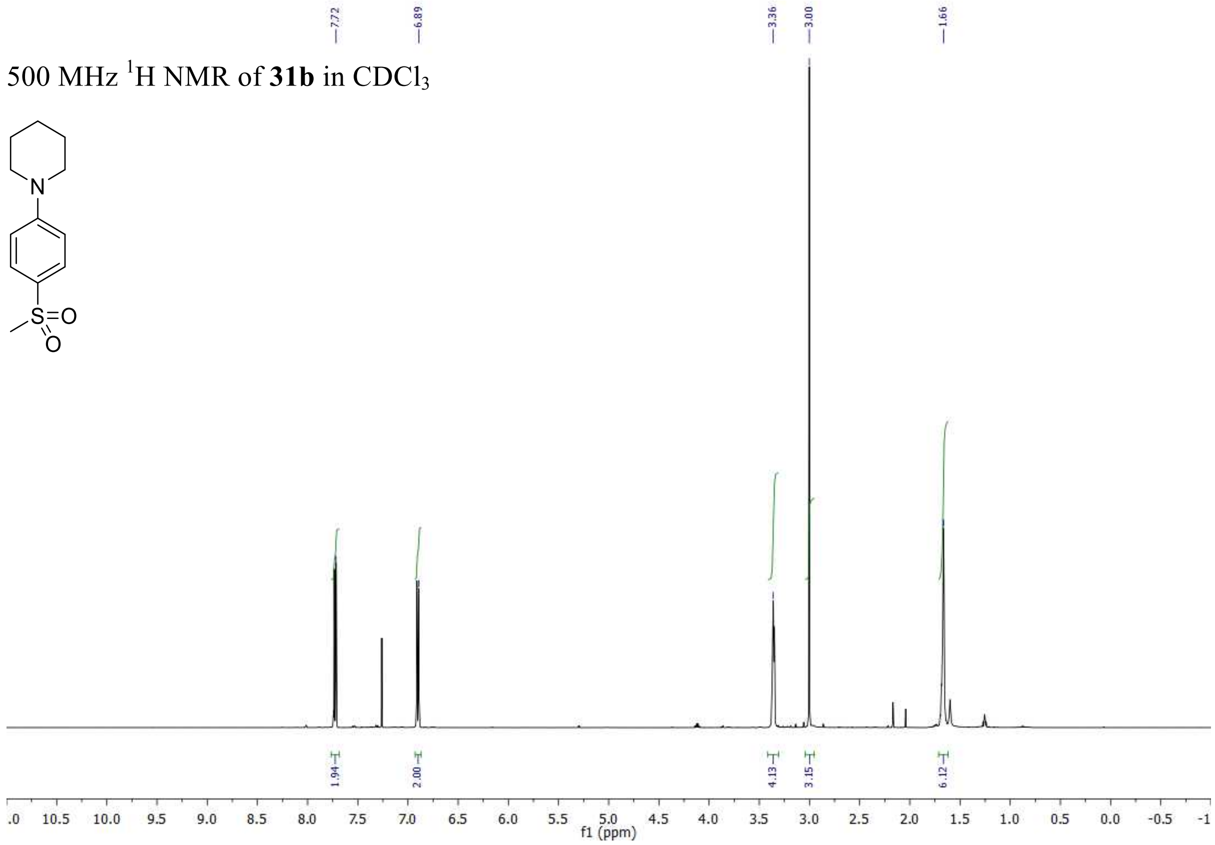

500 MHz ^{1}H NMR of **31b** in $CDCl_3$

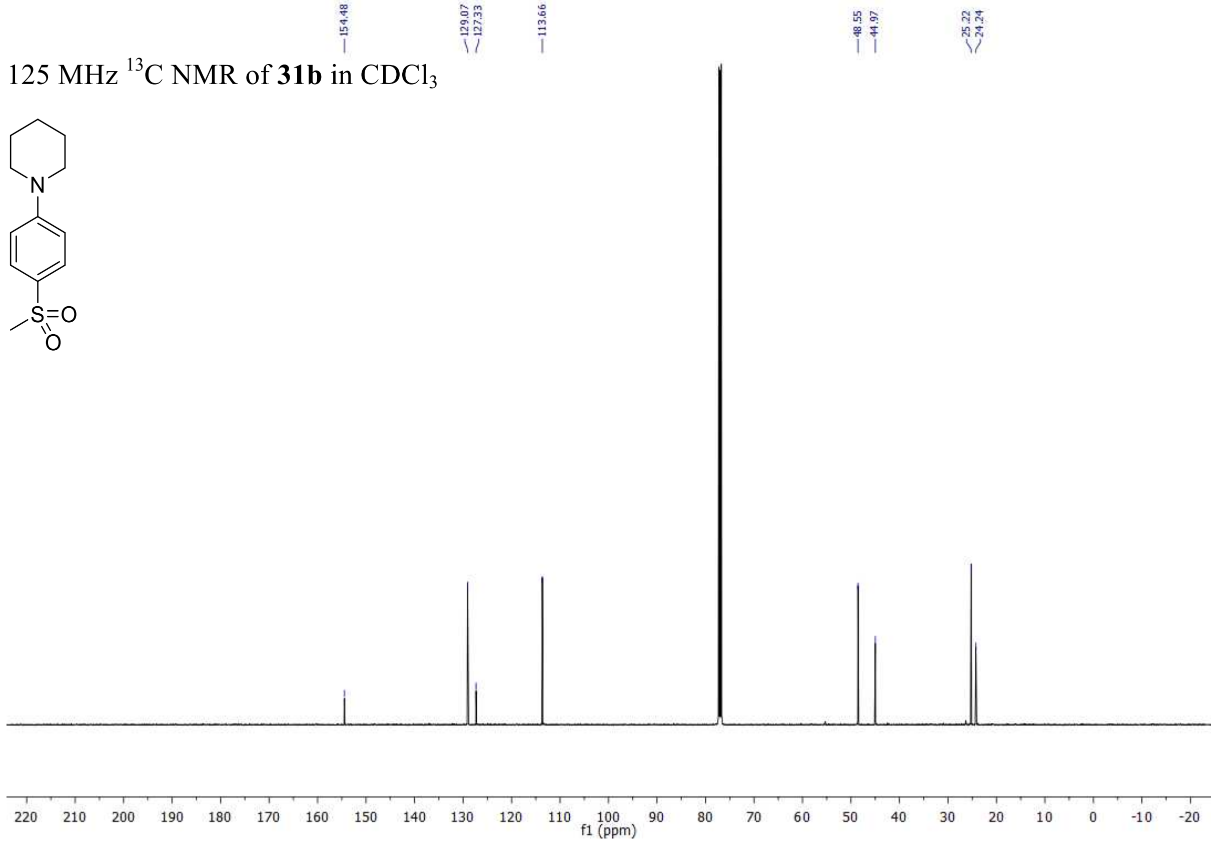

125 MHz ^{13}C NMR of **31b** in $CDCl_3$

500 MHz ^{1}H NMR of **32a** in $CDCl_3$

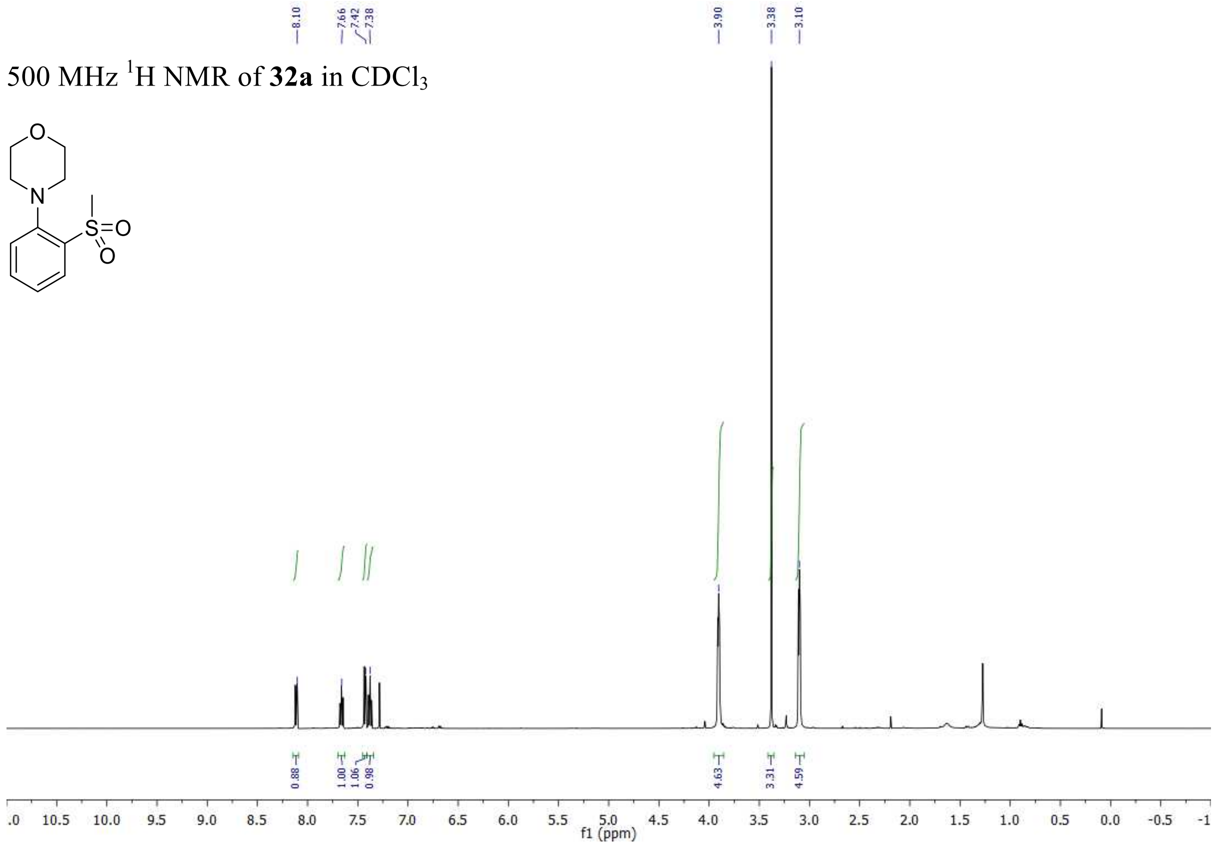

125 MHz ^{13}C NMR of **32a** in $CDCl_3$

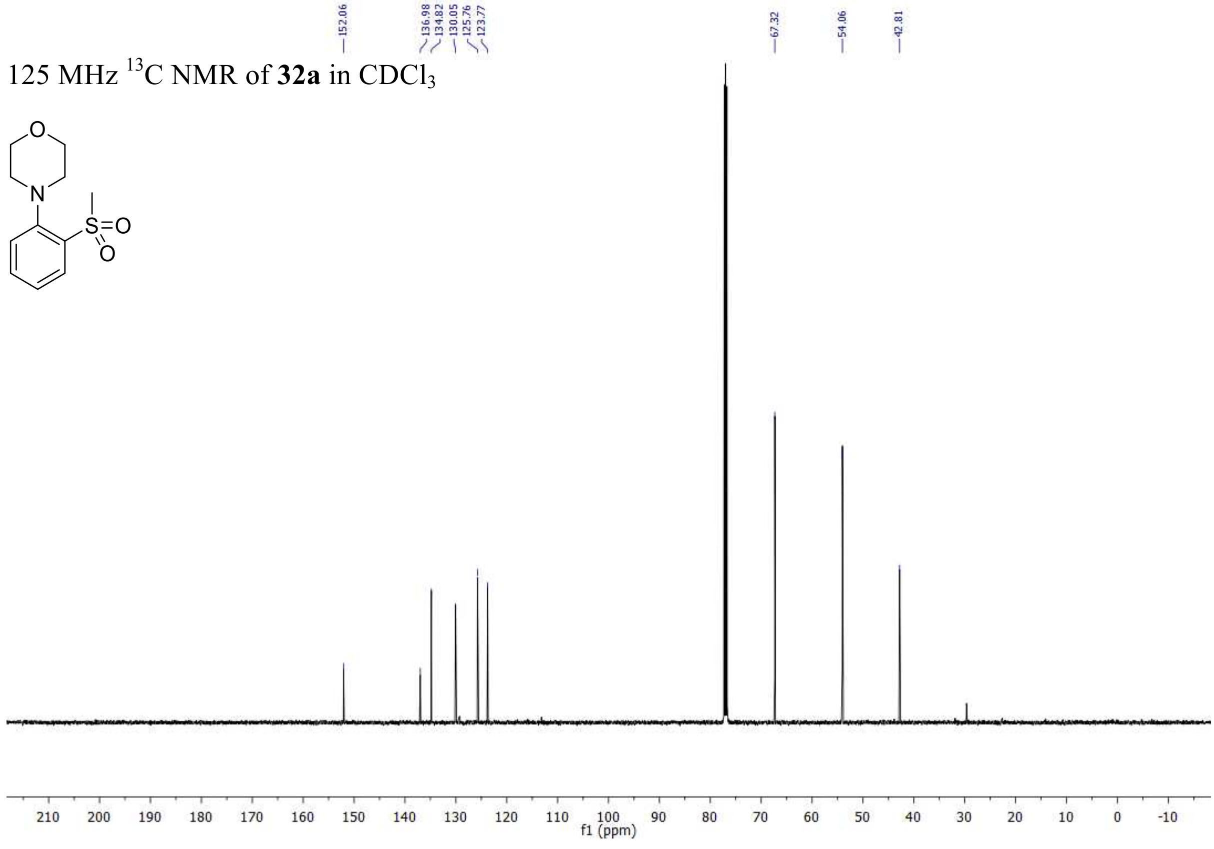

500 MHz ^{1}H NMR of **32b** in $CDCl_3$

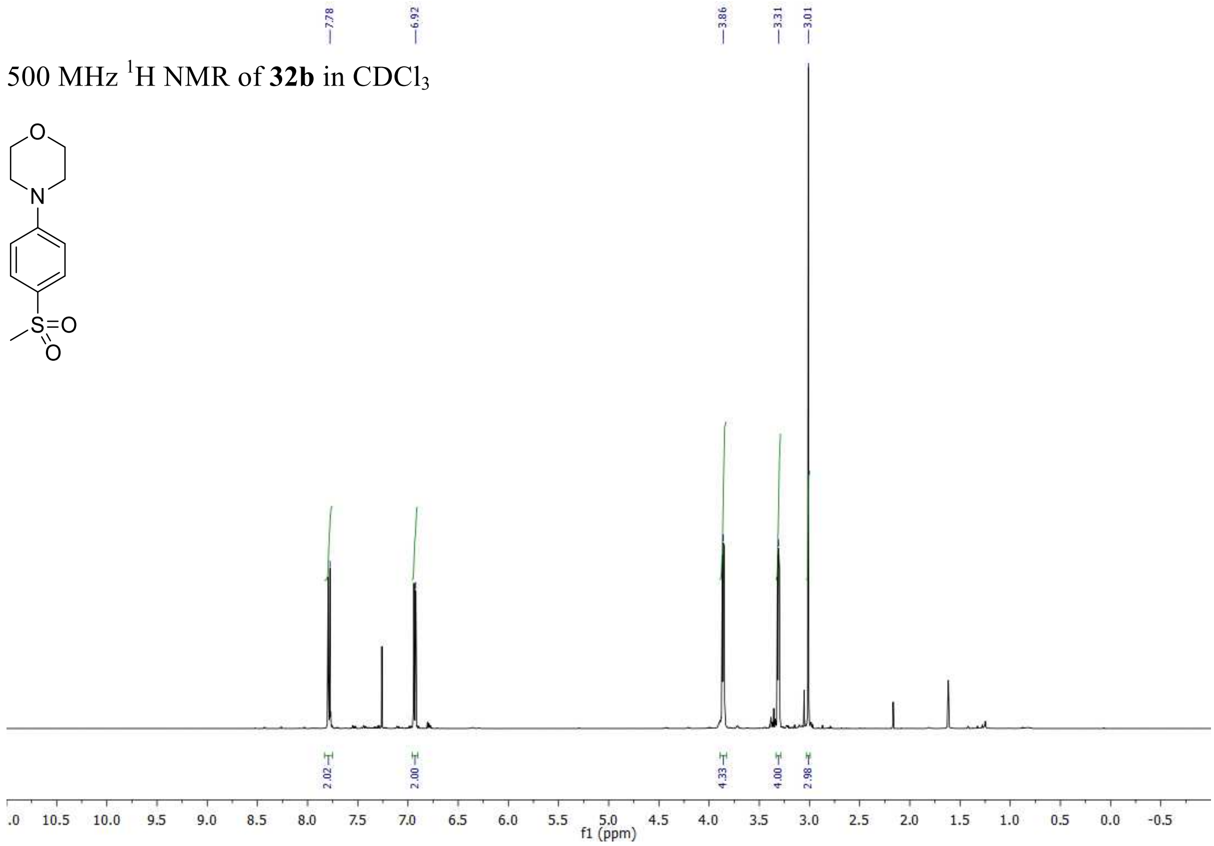

125 MHz ^{13}C NMR of **32b** in $CDCl_3$

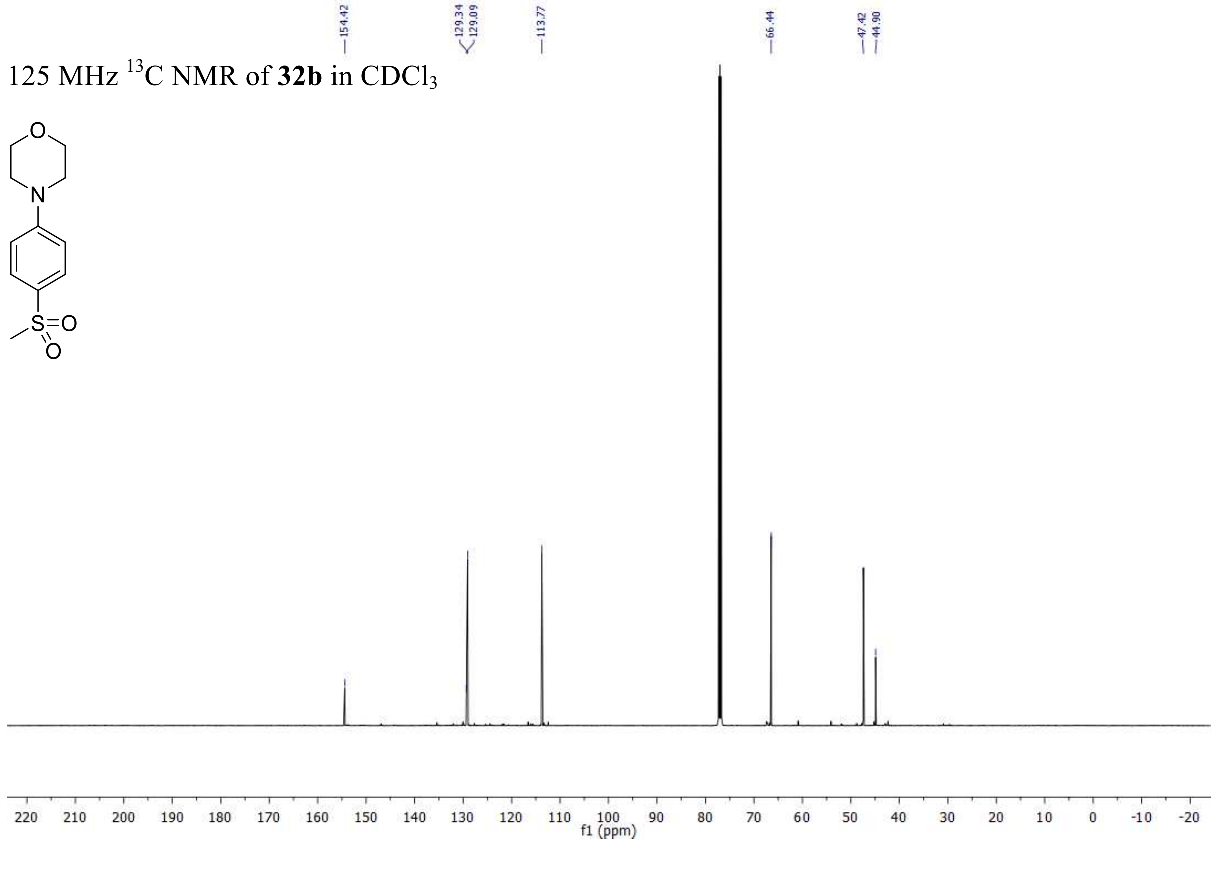

400 MHz ^{1}H NMR of **33a** in $CDCl_3$

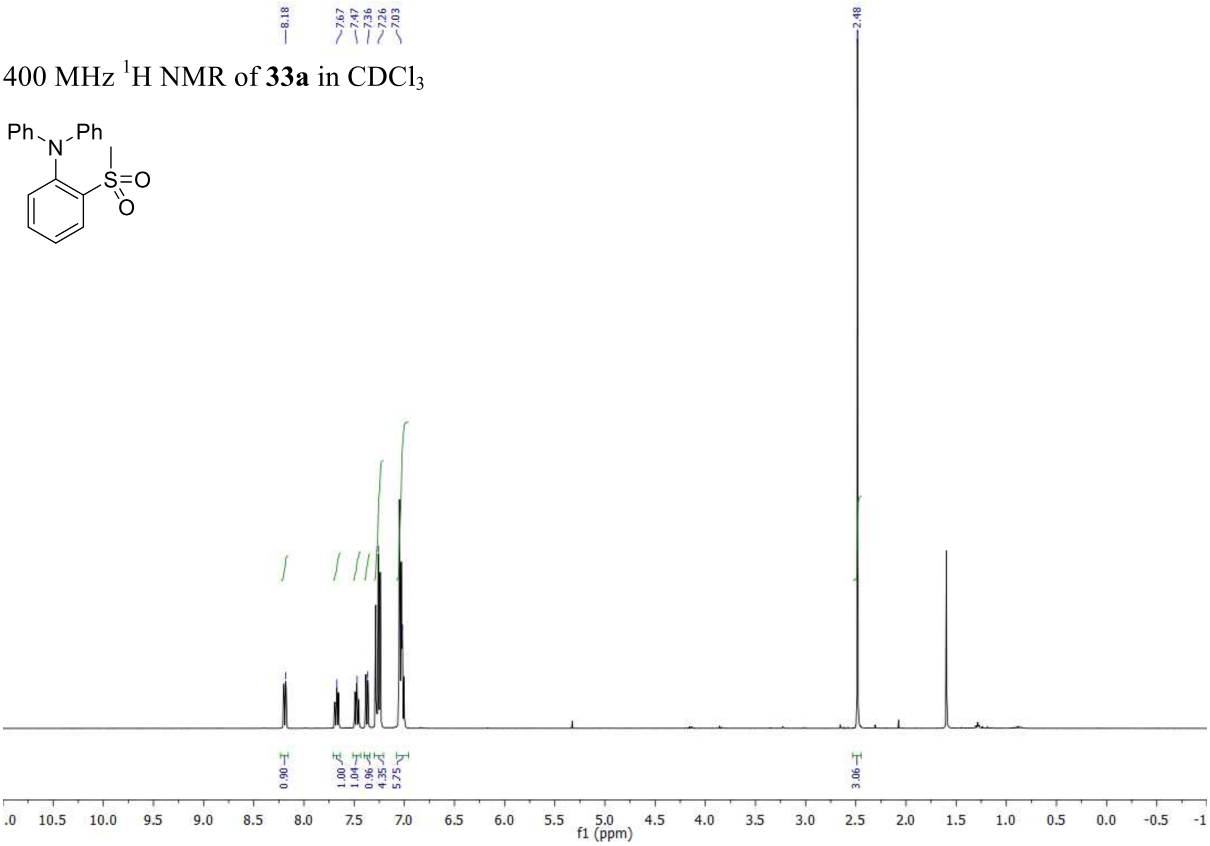

100 MHz ^{13}C NMR of **33a** in $CDCl_3$

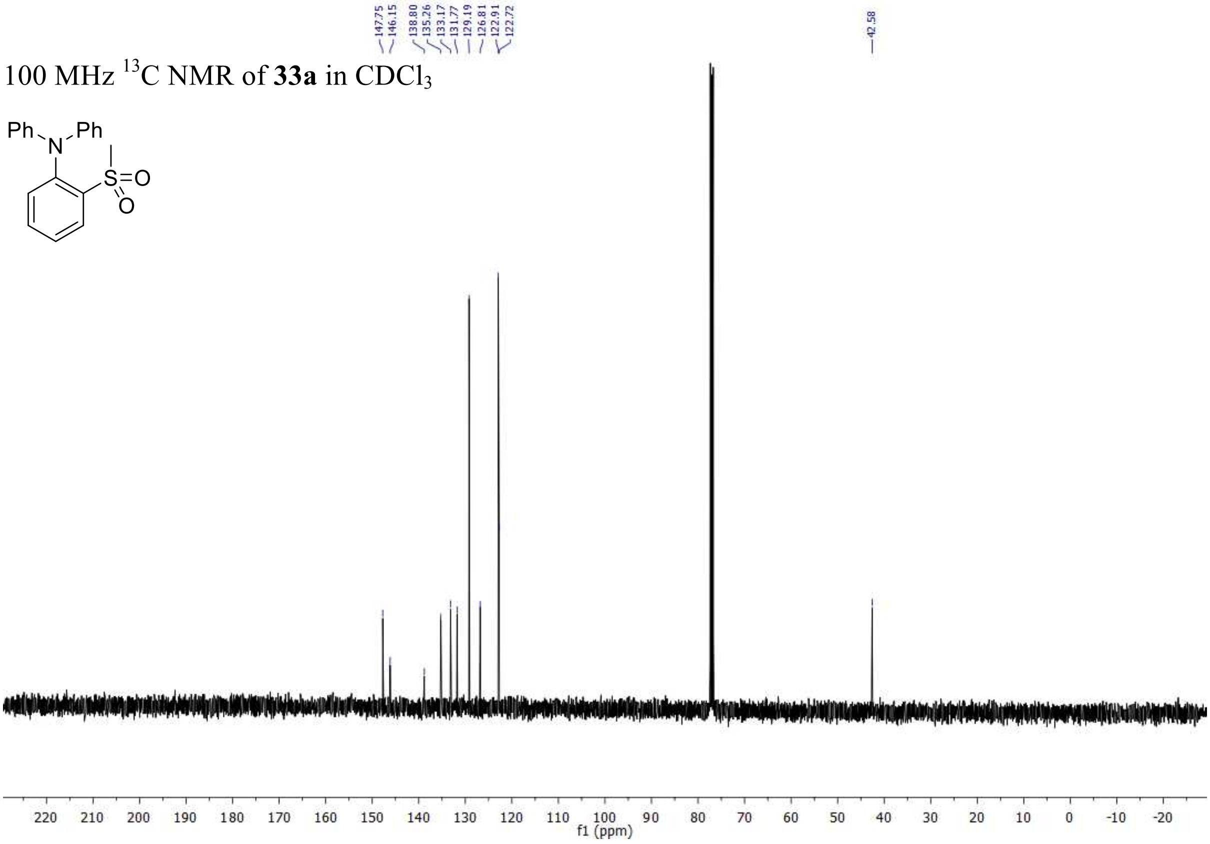

400 MHz ^{1}H NMR of **33b** in $CDCl_3$

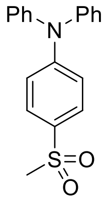

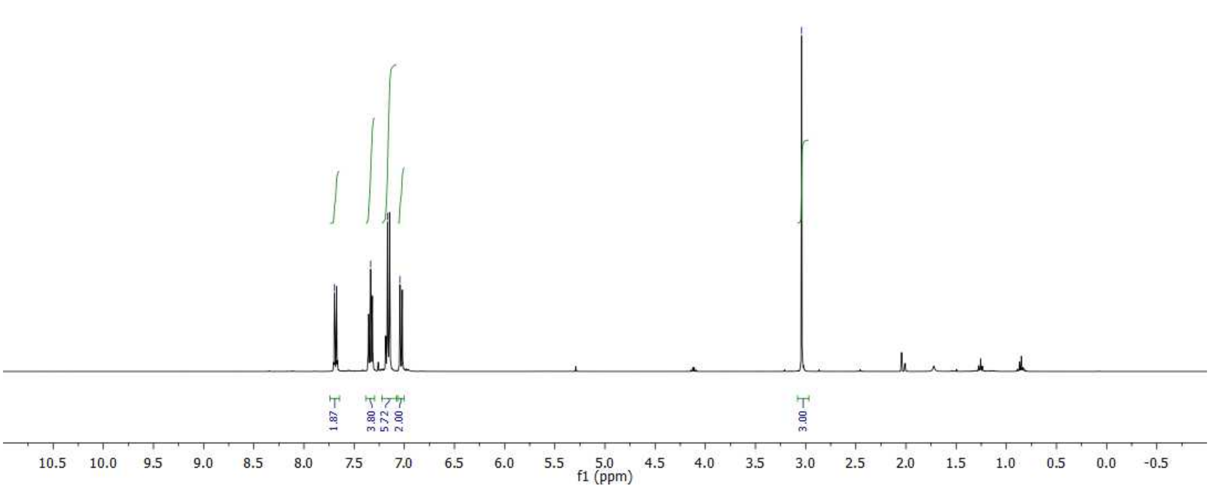

100 MHz ^{13}C NMR of **33b** in $CDCl_3$

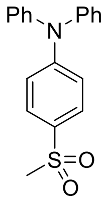

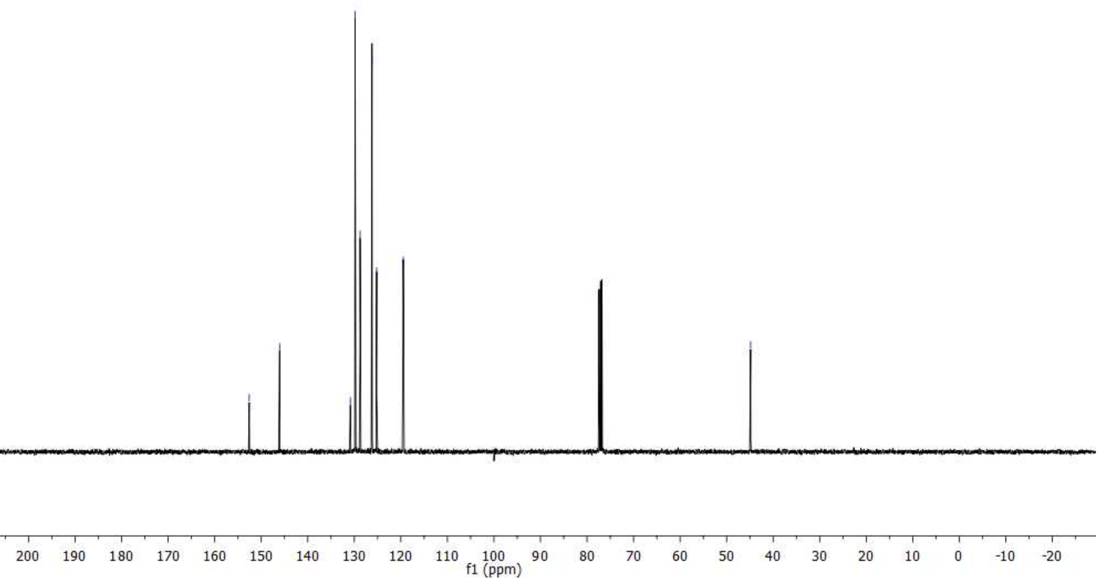

400 MHz ^{1}H NMR of **34a** in $CDCl_3$

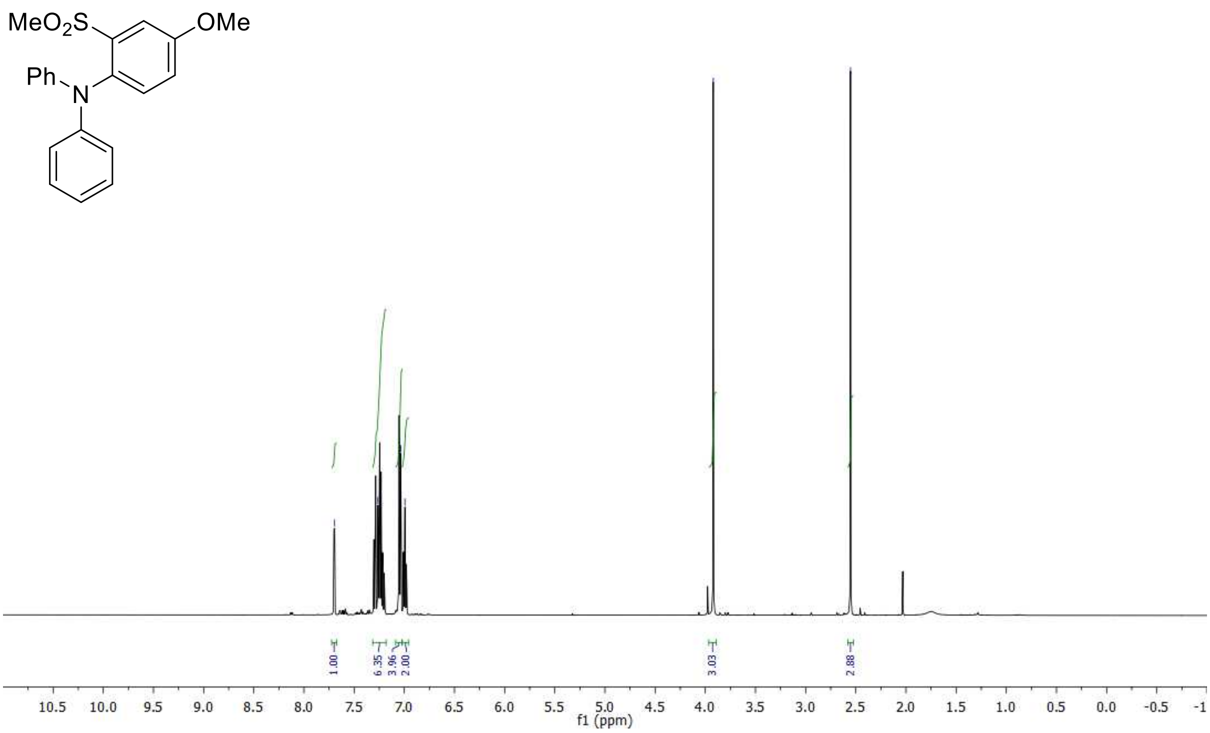

100 MHz ^{13}C NMR of **34a** in $CDCl_3$

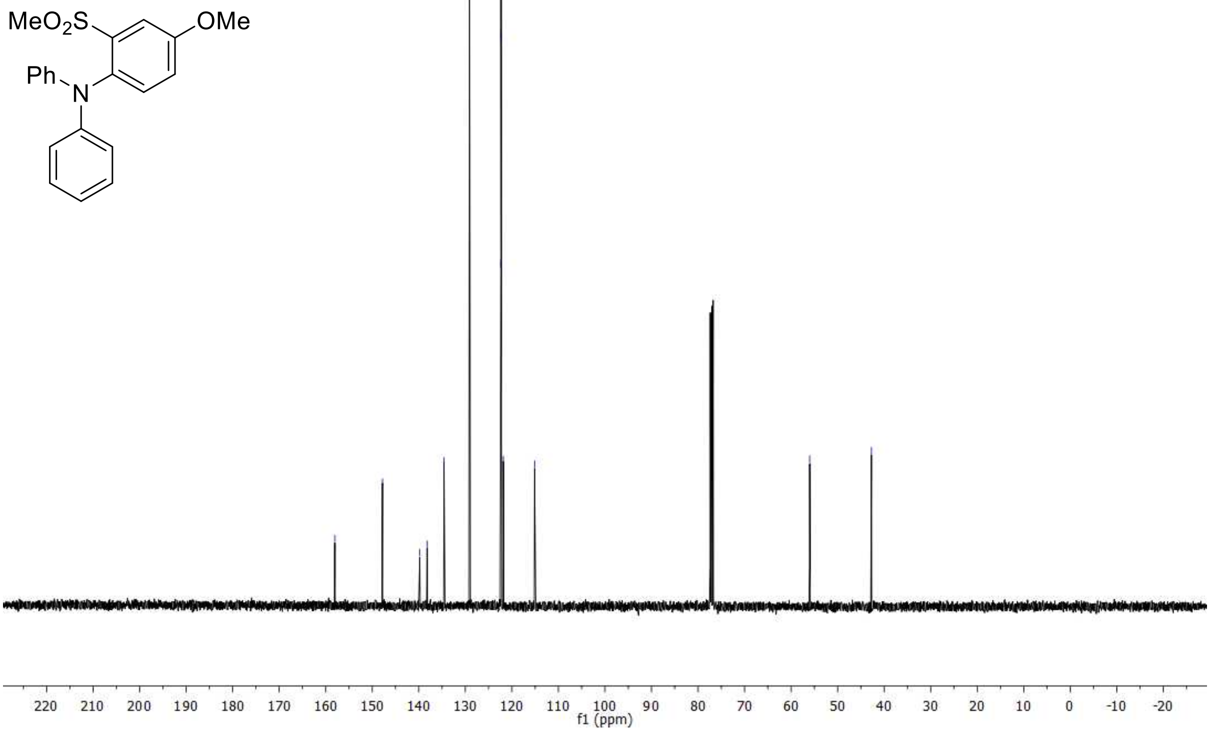

400 MHz ^{1}H NMR of **34b** in $CDCl_3$

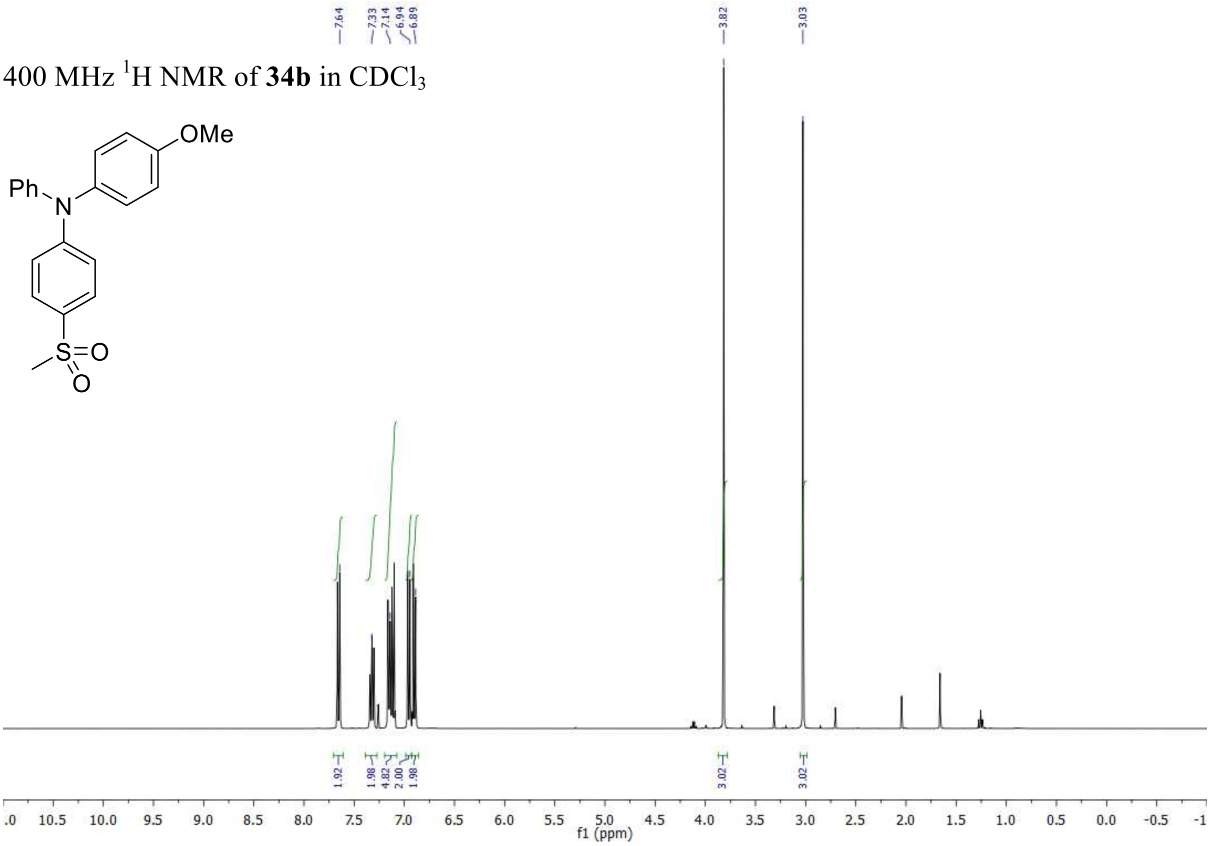

100 MHz ^{13}C NMR of **34b** in $CDCl_3$

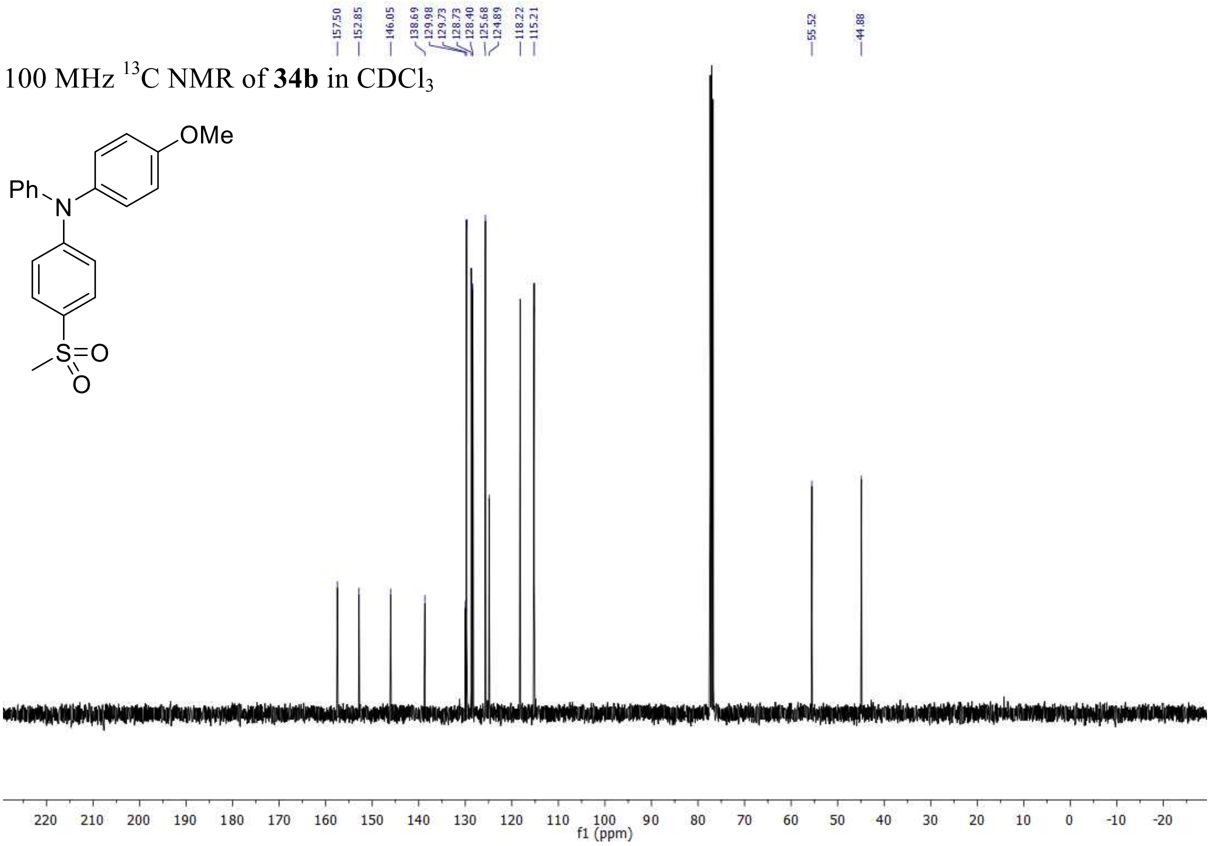

400 MHz ^{1}H NMR of **34c** in $CDCl_3$

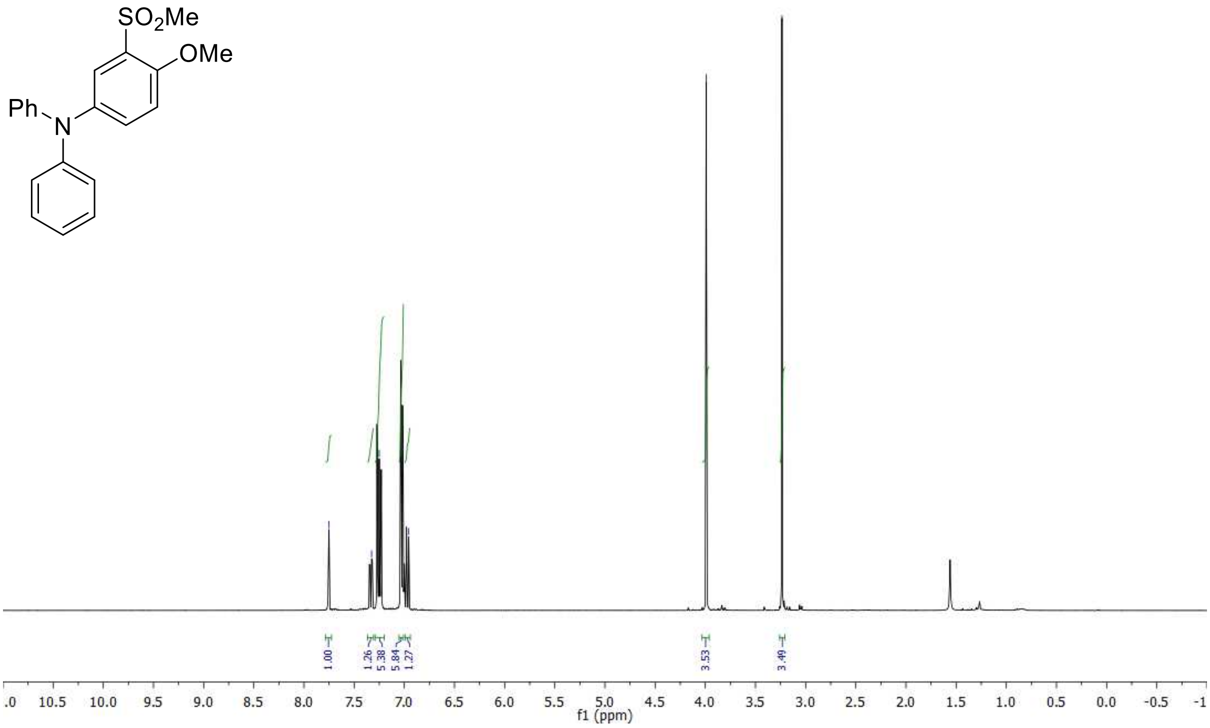

100 MHz ^{13}C NMR of **34c** in $CDCl_3$

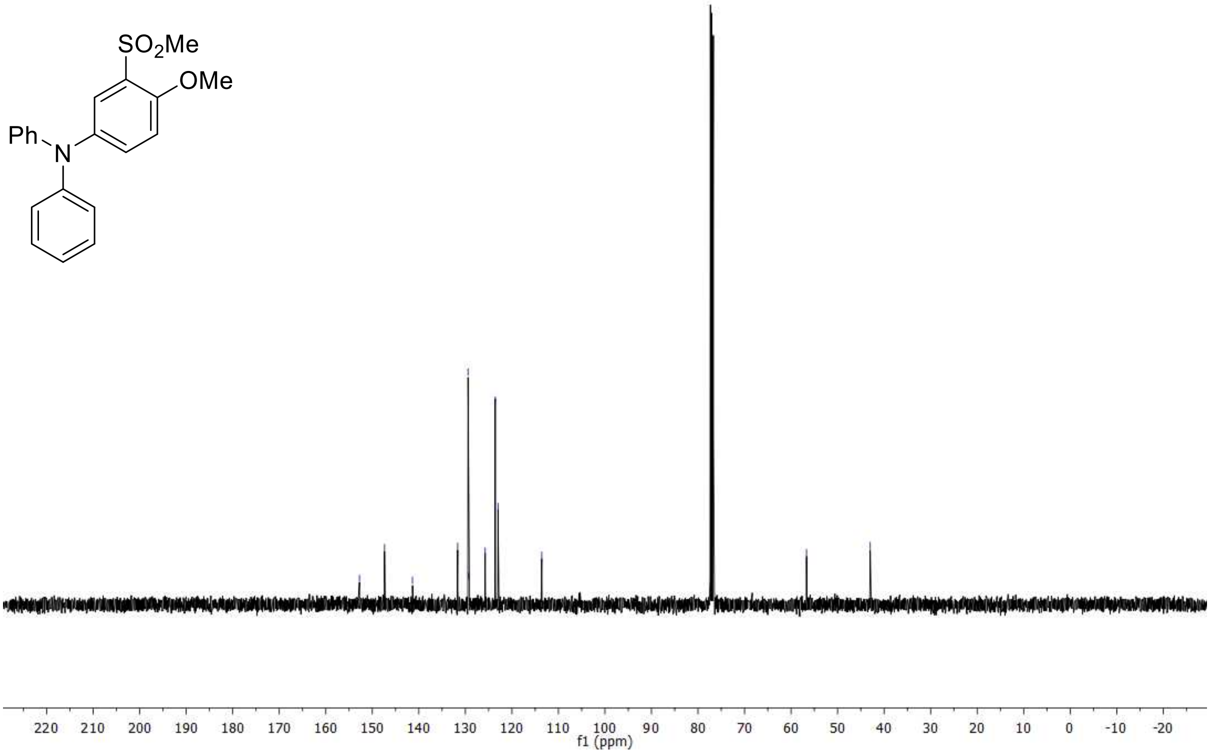

400 MHz ^{1}H NMR of **35** in $CDCl_3$

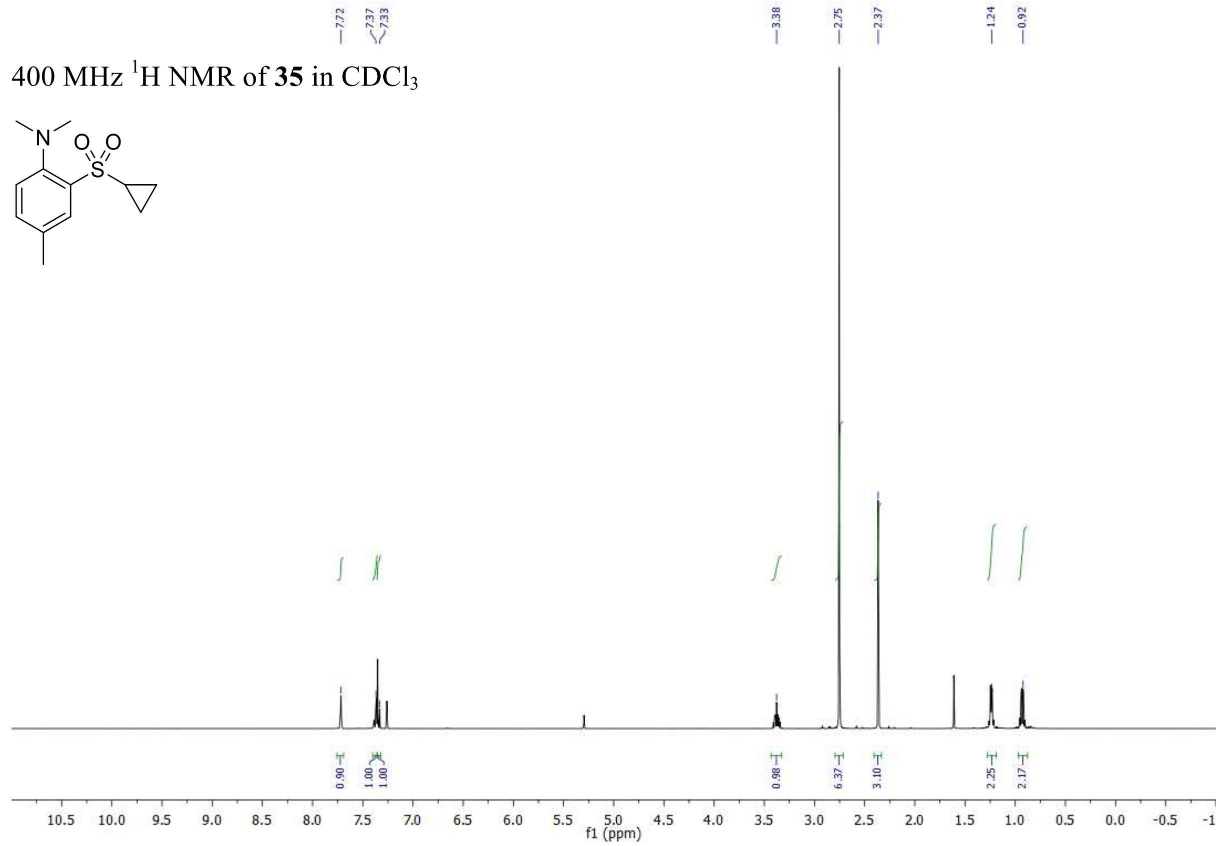

100 MHz ^{13}C NMR of **35** in $CDCl_3$

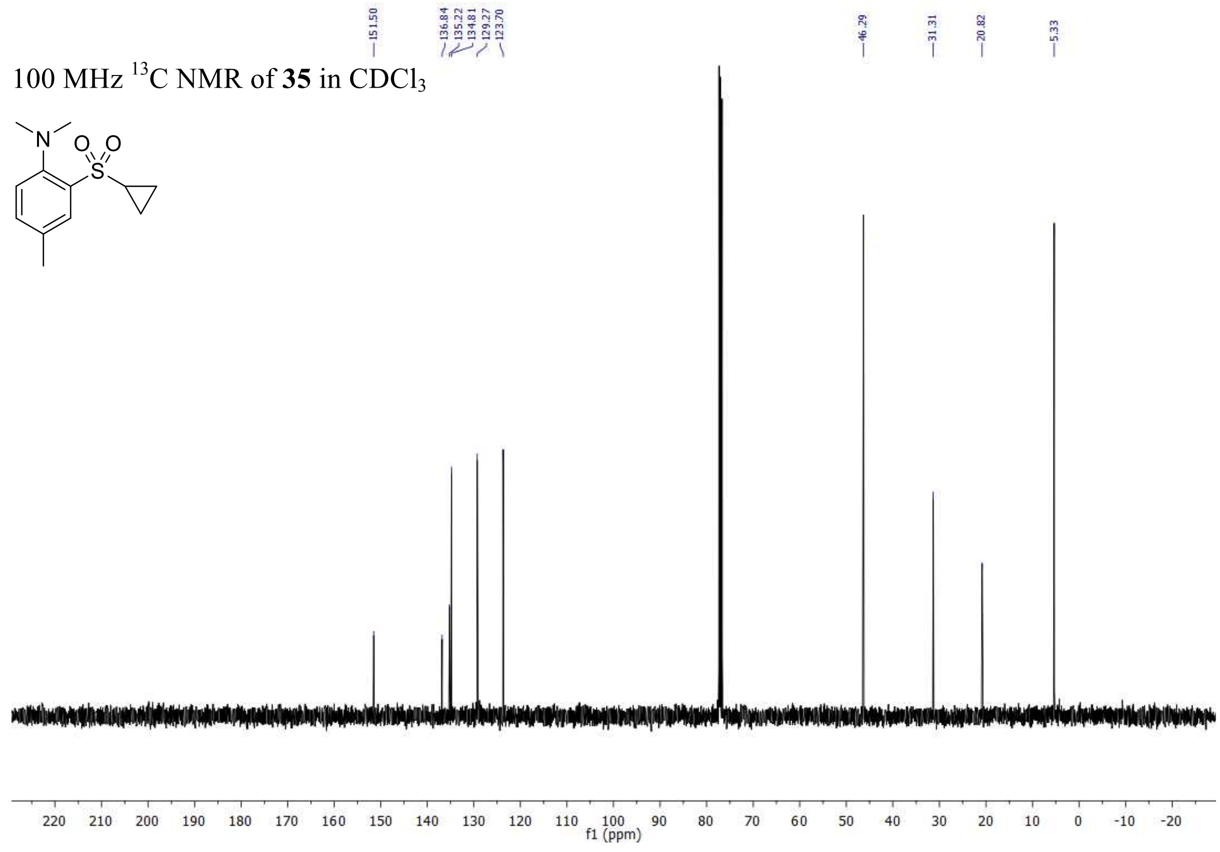

400 MHz ^{1}H NMR of **36** in $CDCl_3$

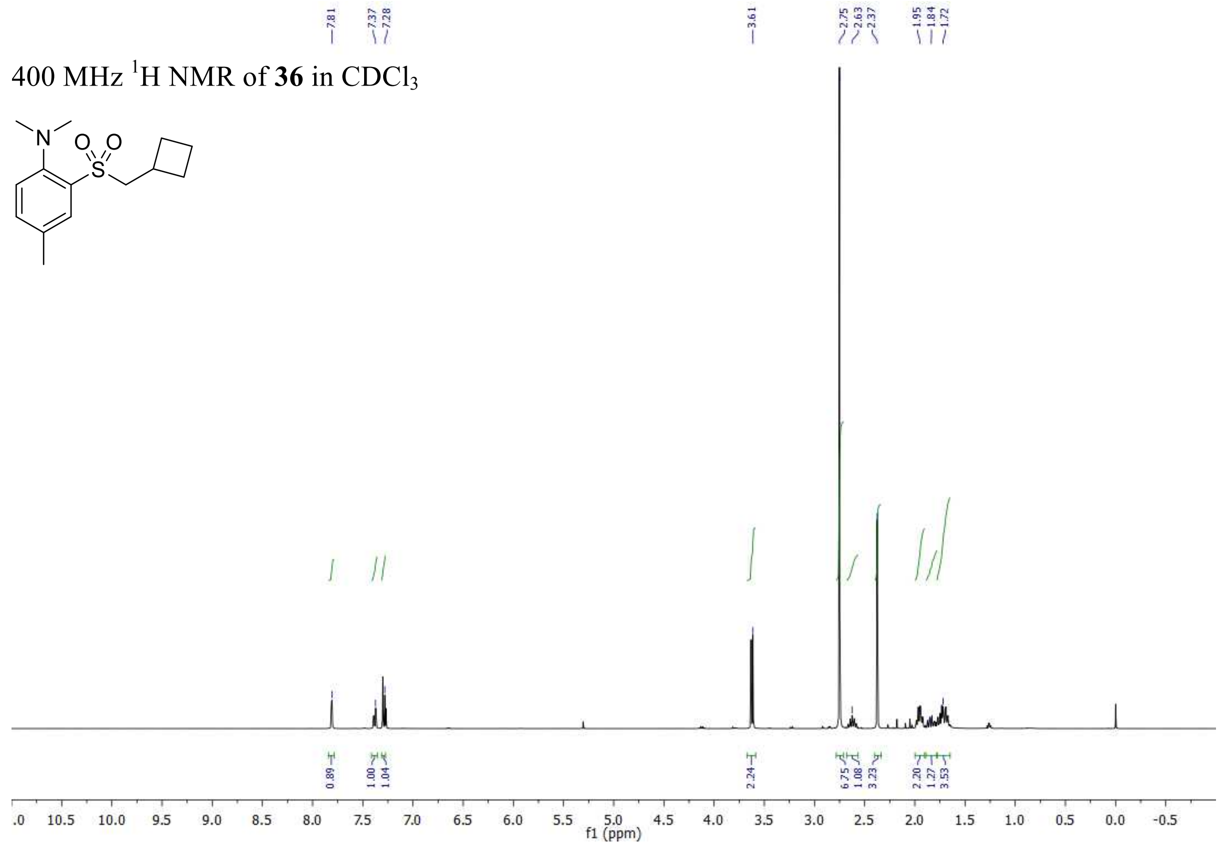

100 MHz ^{13}C NMR of **36** in $CDCl_3$

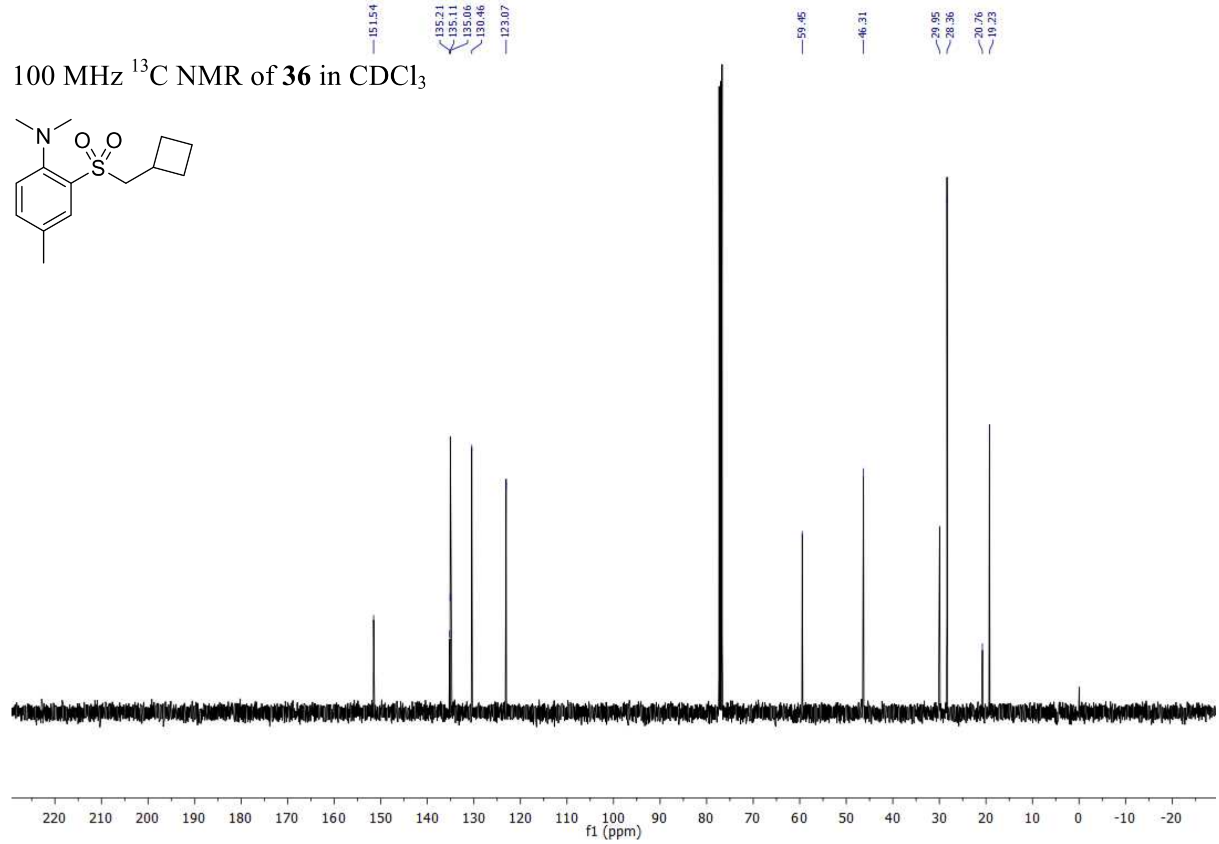

400 MHz ^{1}H NMR of **37** in $CDCl_3$

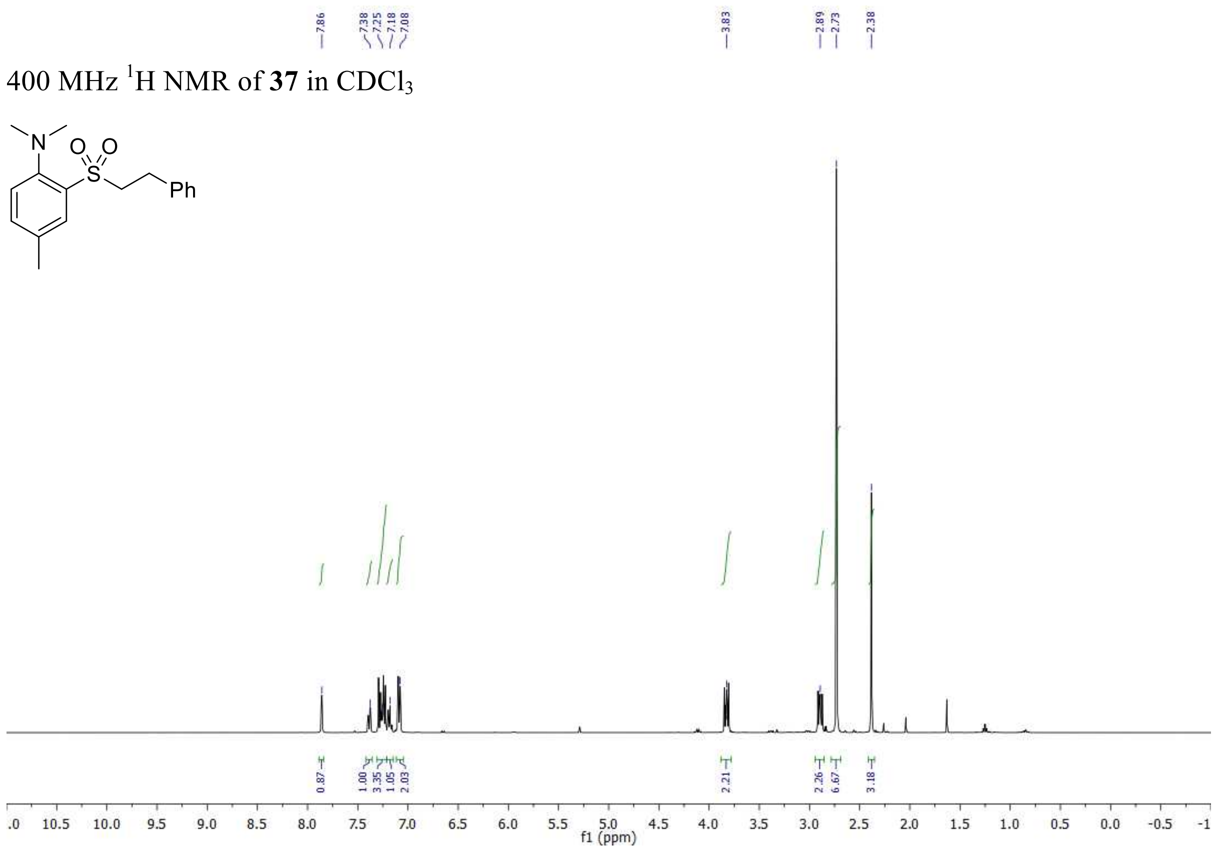

100 MHz ^{13}C NMR of **37** in $CDCl_3$

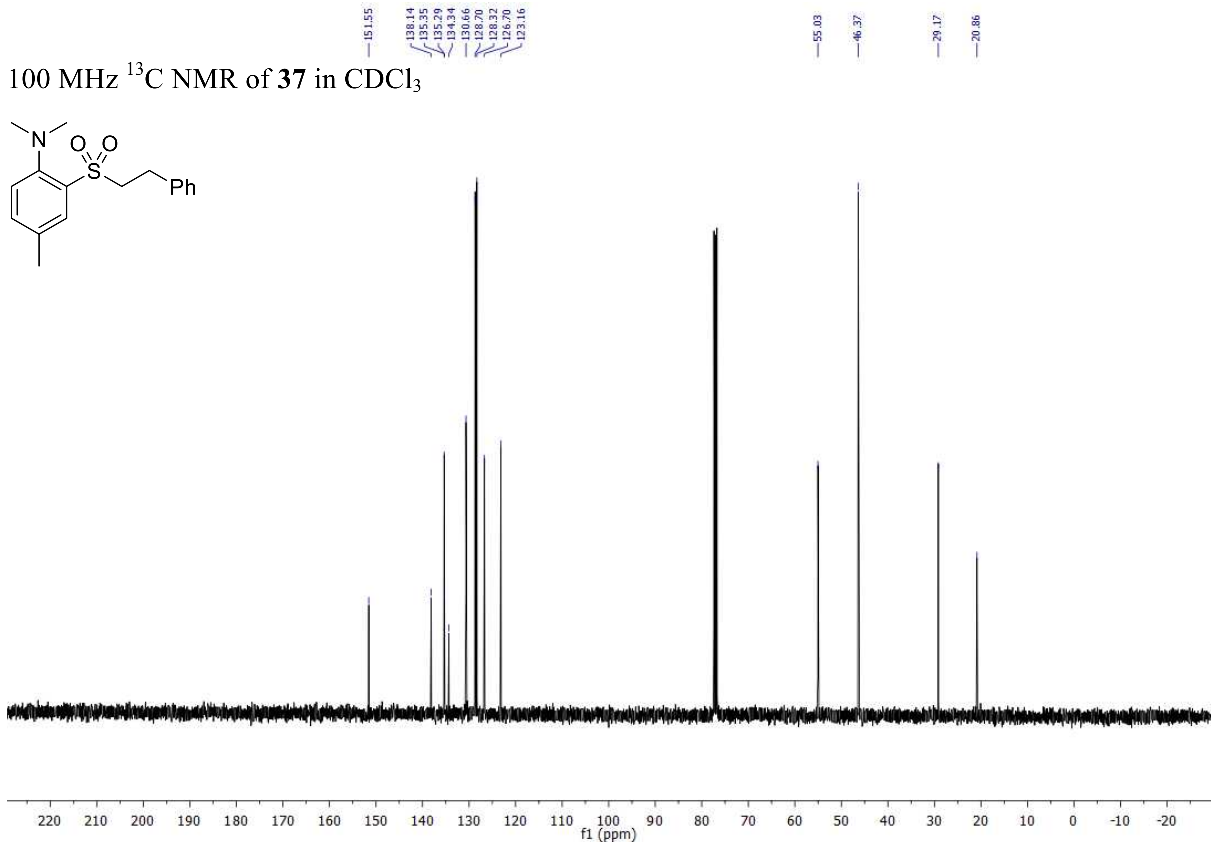

400 MHz ^{1}H NMR of **38** in $CDCl_3$

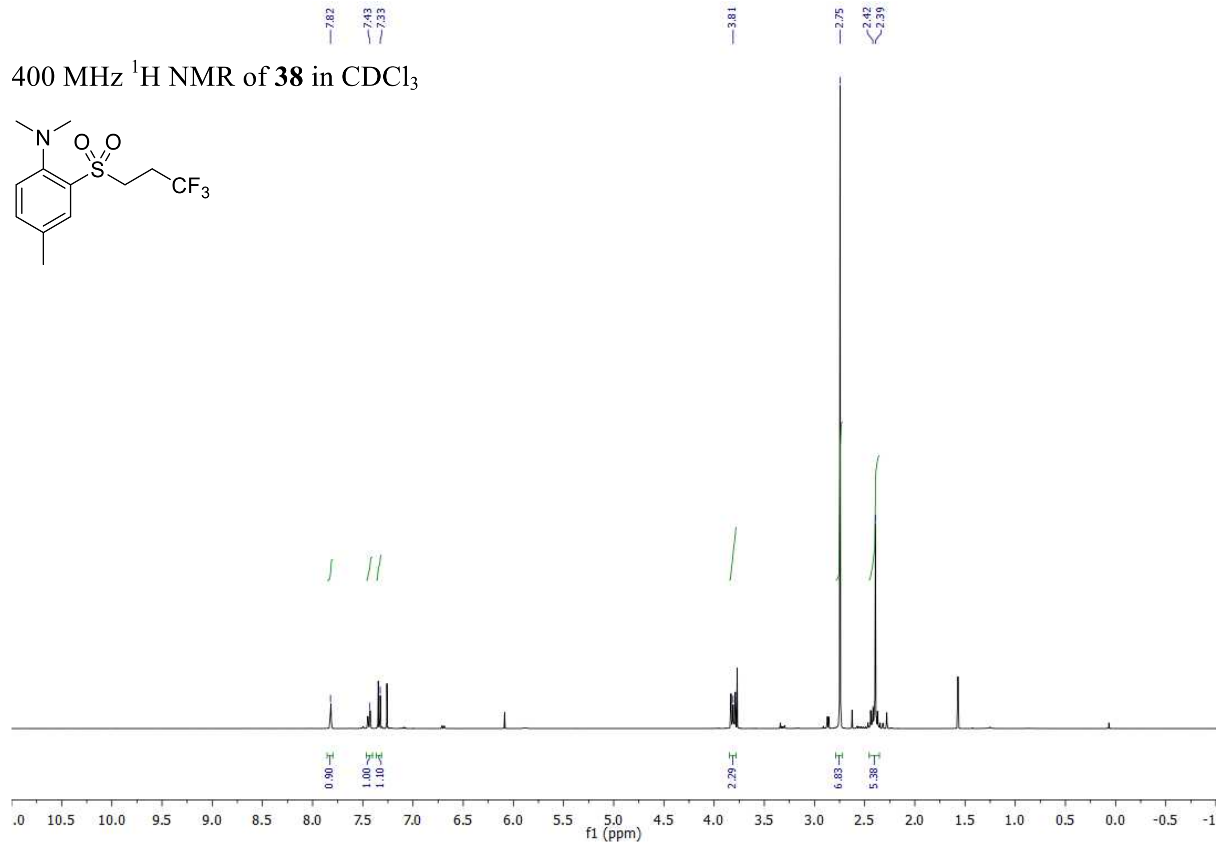

100 MHz ^{13}C NMR of **38** in $CDCl_3$

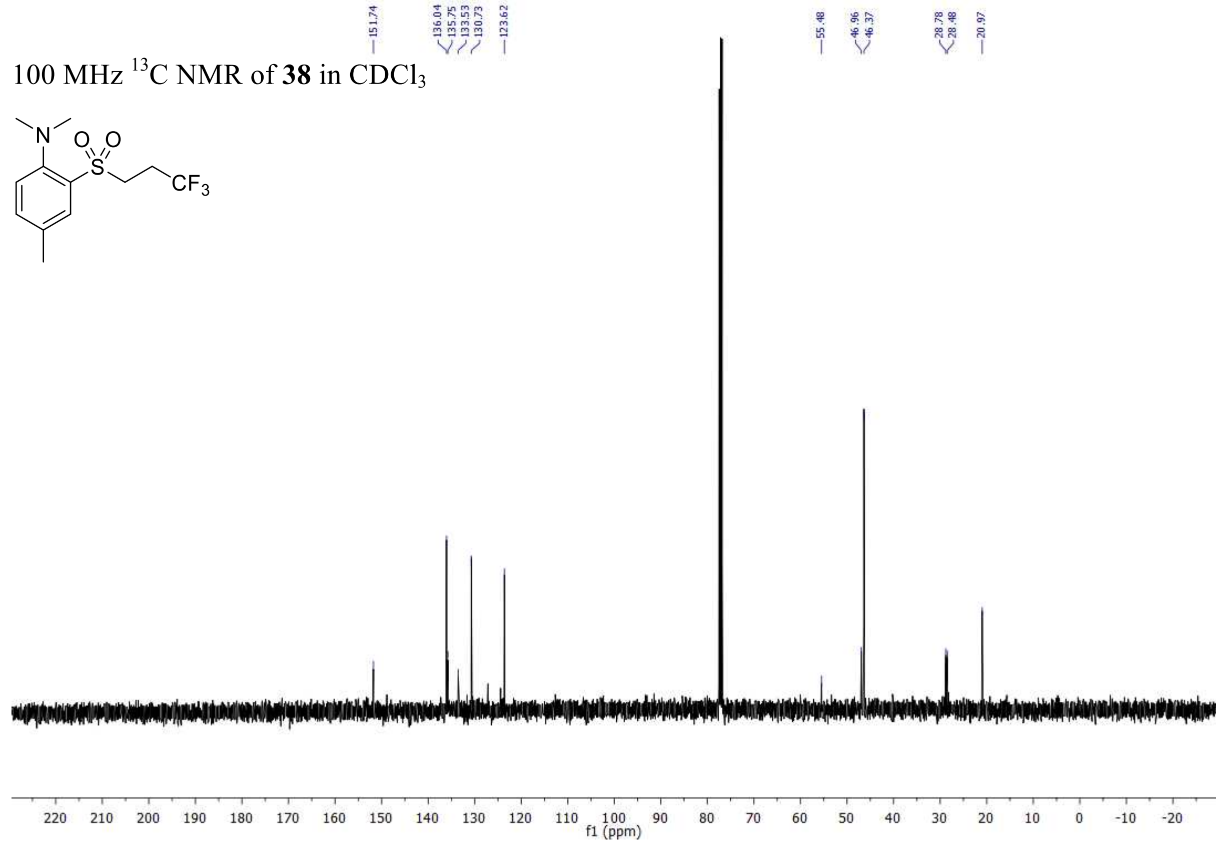

400 MHz ^{1}H NMR of **39** in $CDCl_3$

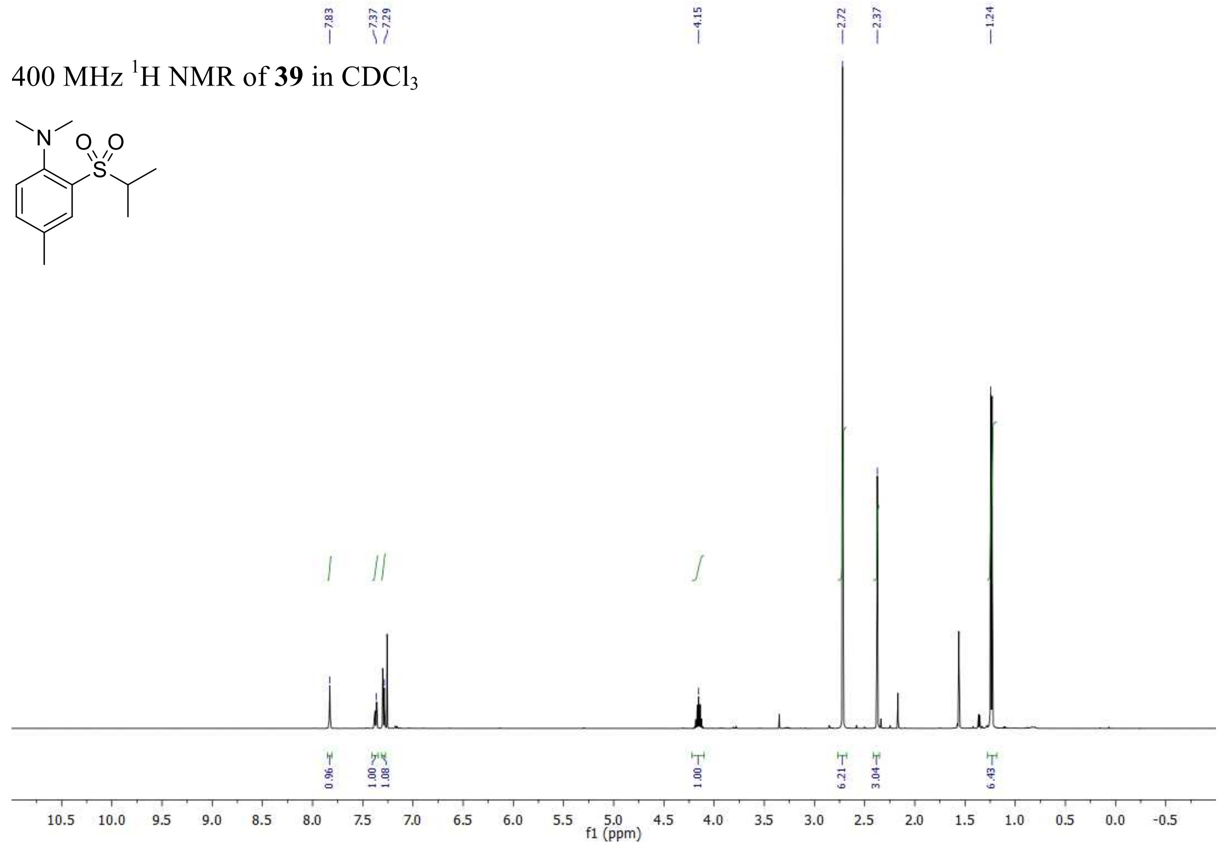

100 MHz ^{13}C NMR of **39** in $CDCl_3$

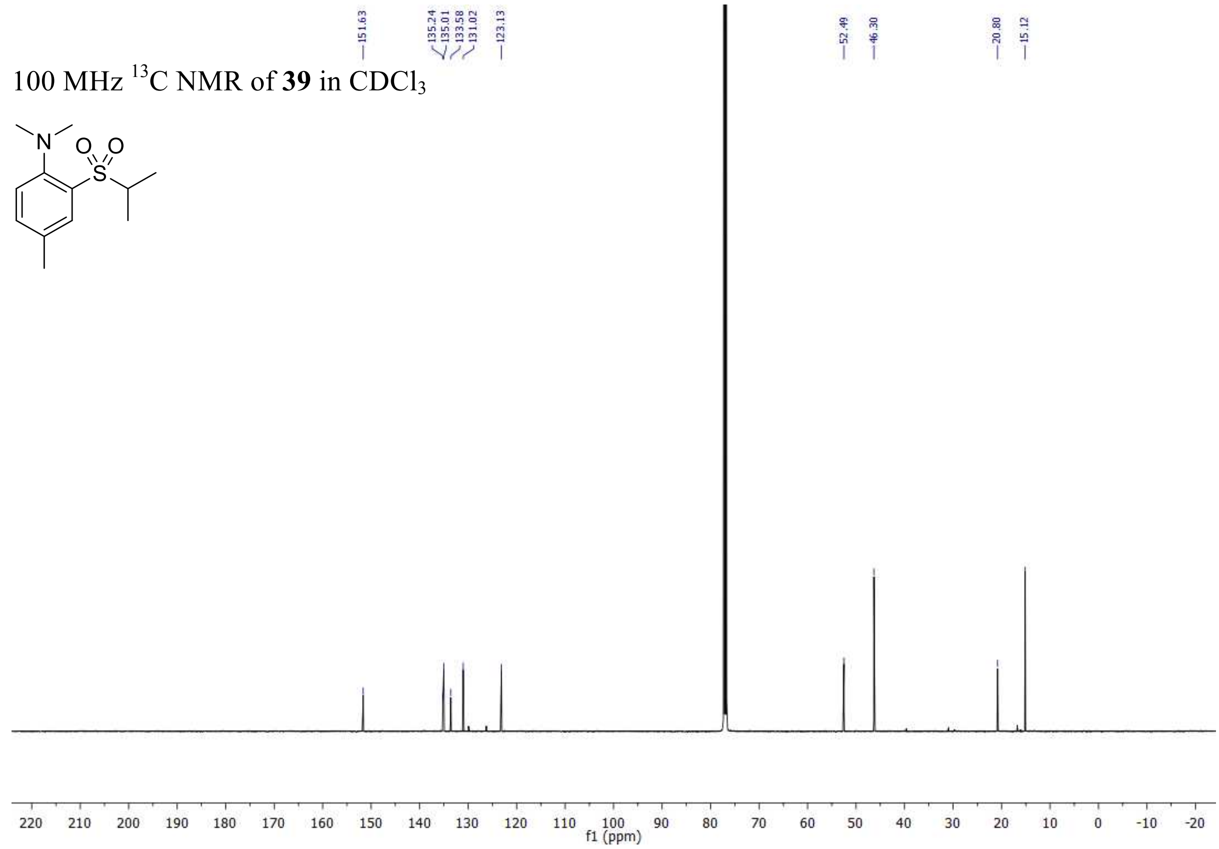

400 MHz ^{1}H NMR of **40** in $CDCl_3$

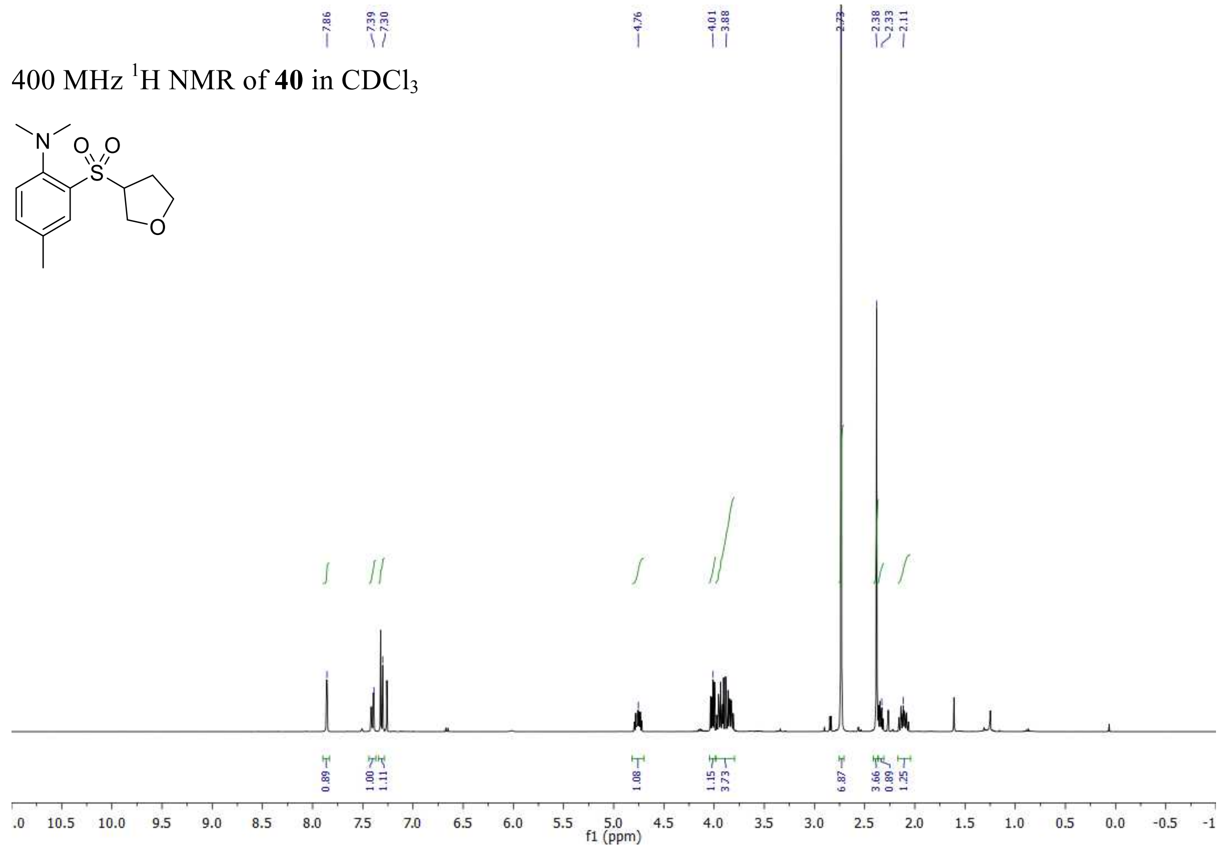

100 MHz ^{13}C NMR of **40** in $CDCl_3$

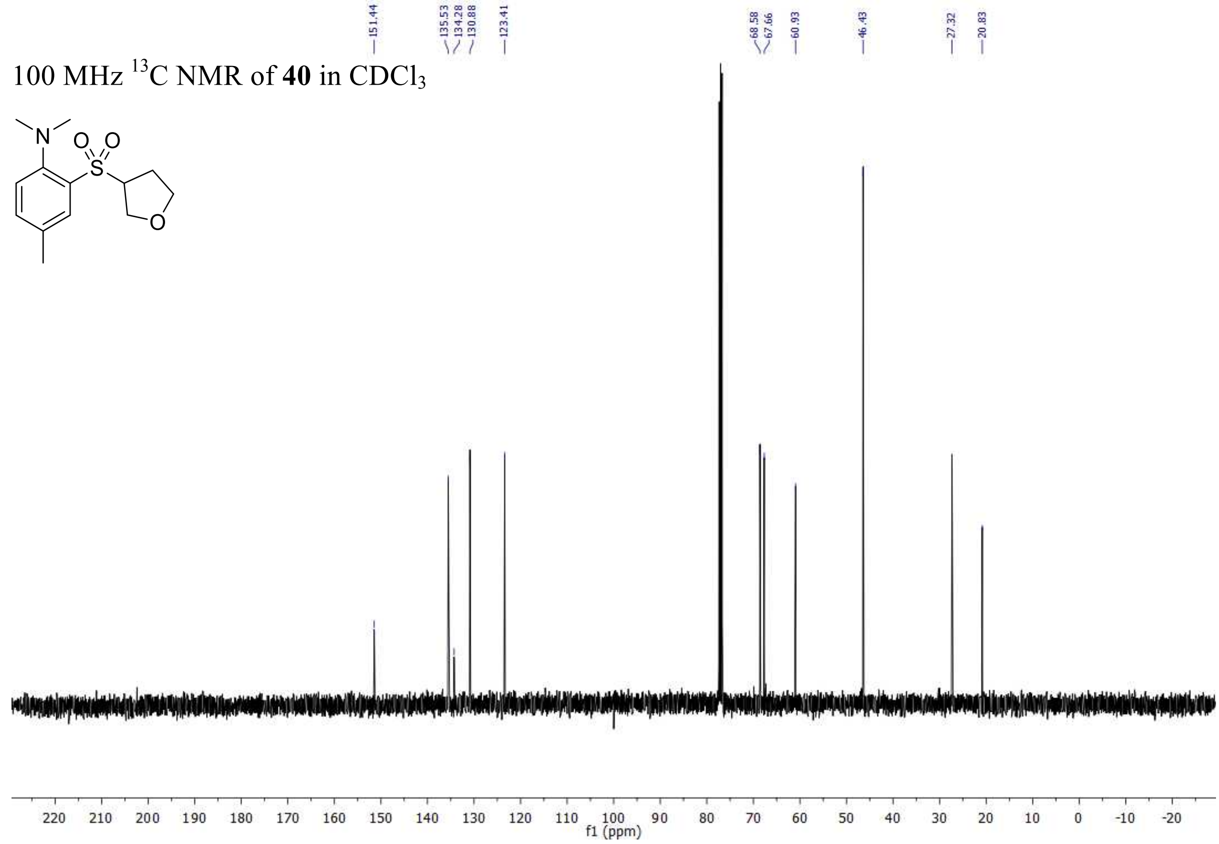

400 MHz ^{1}H NMR of **41** in $CDCl_3$

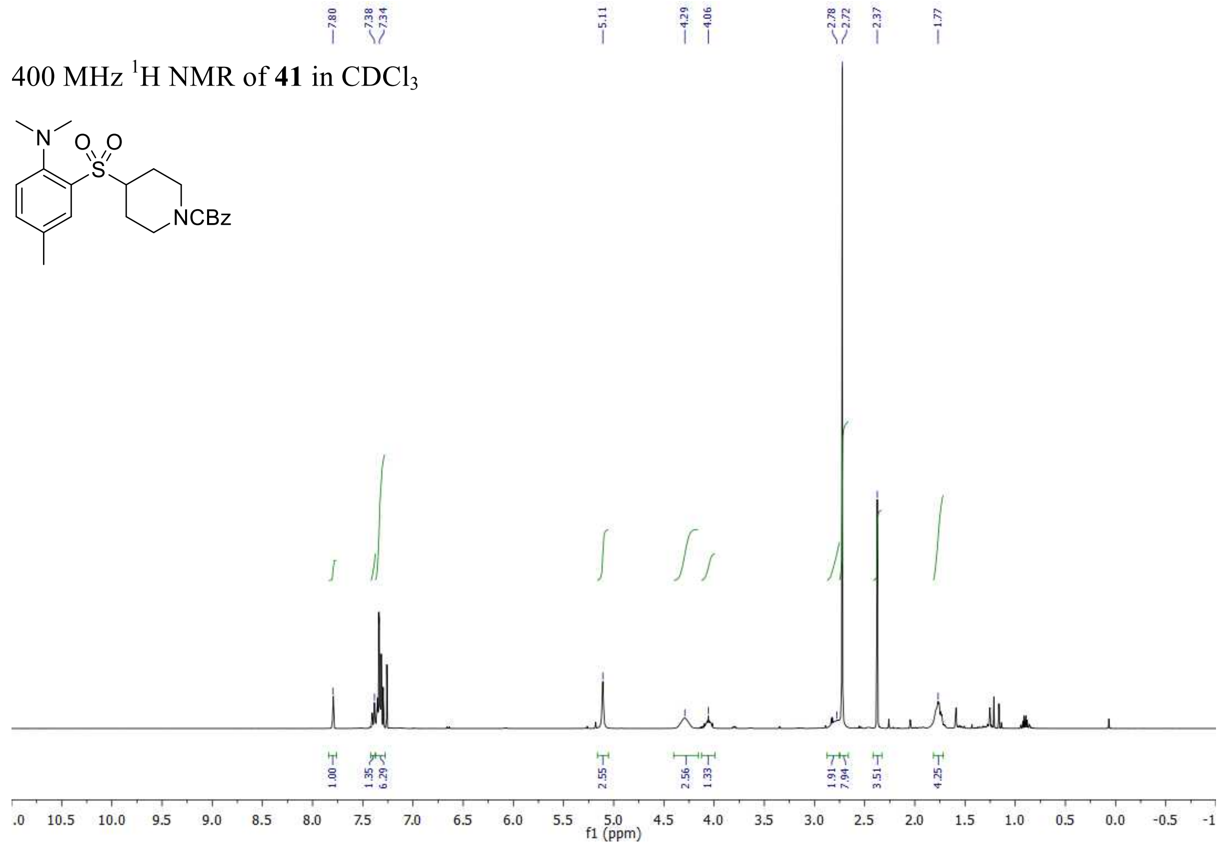

100 MHz ^{13}C NMR of **41** in $CDCl_3$

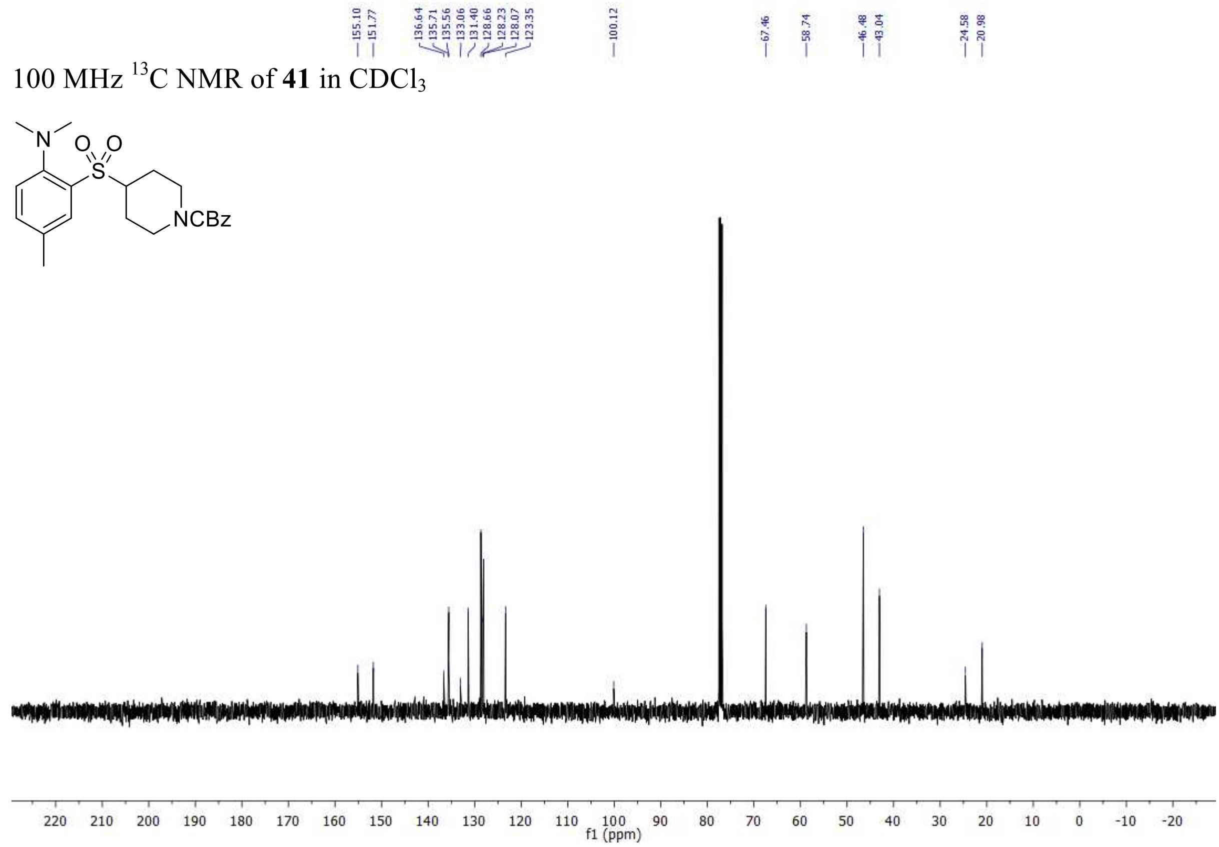

500 MHz ^{1}H NMR of **42** in $CDCl_3$

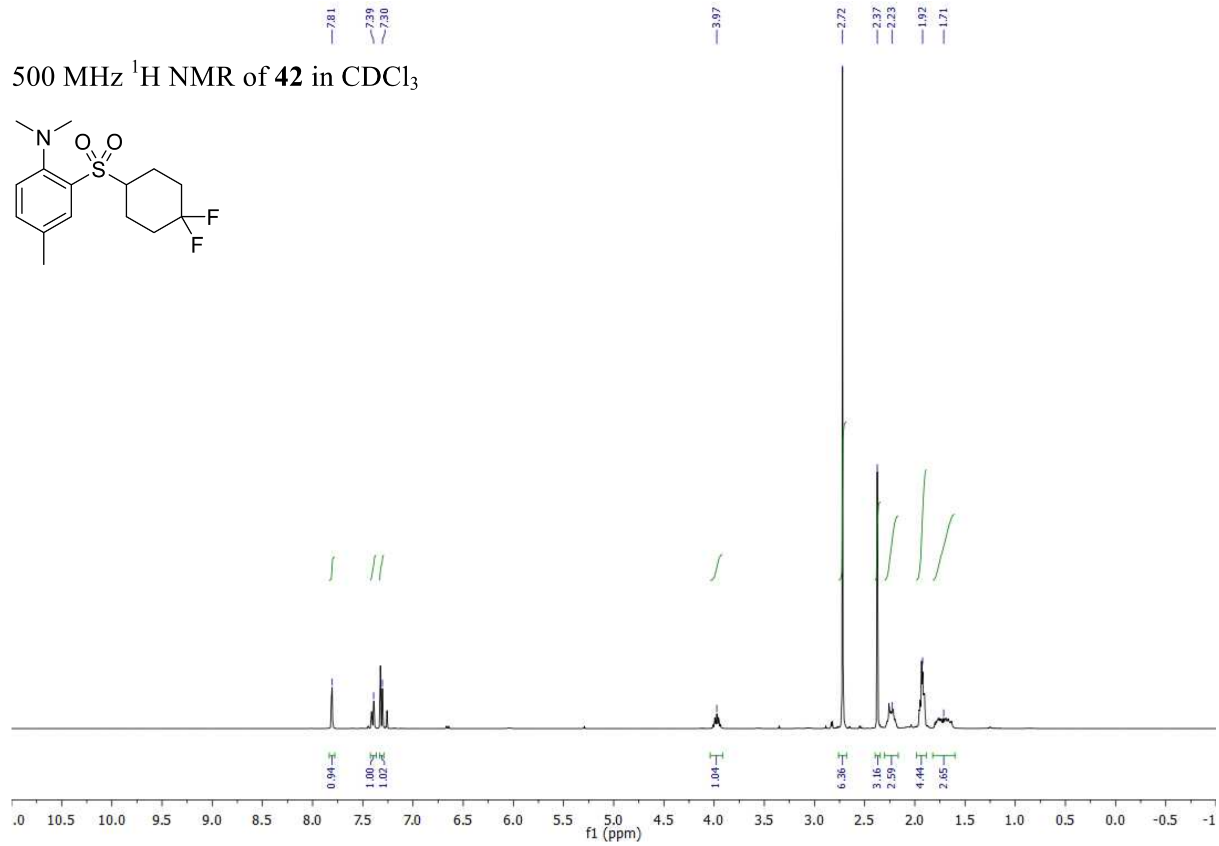

125 MHz ^{13}C NMR of **42** in $CDCl_3$

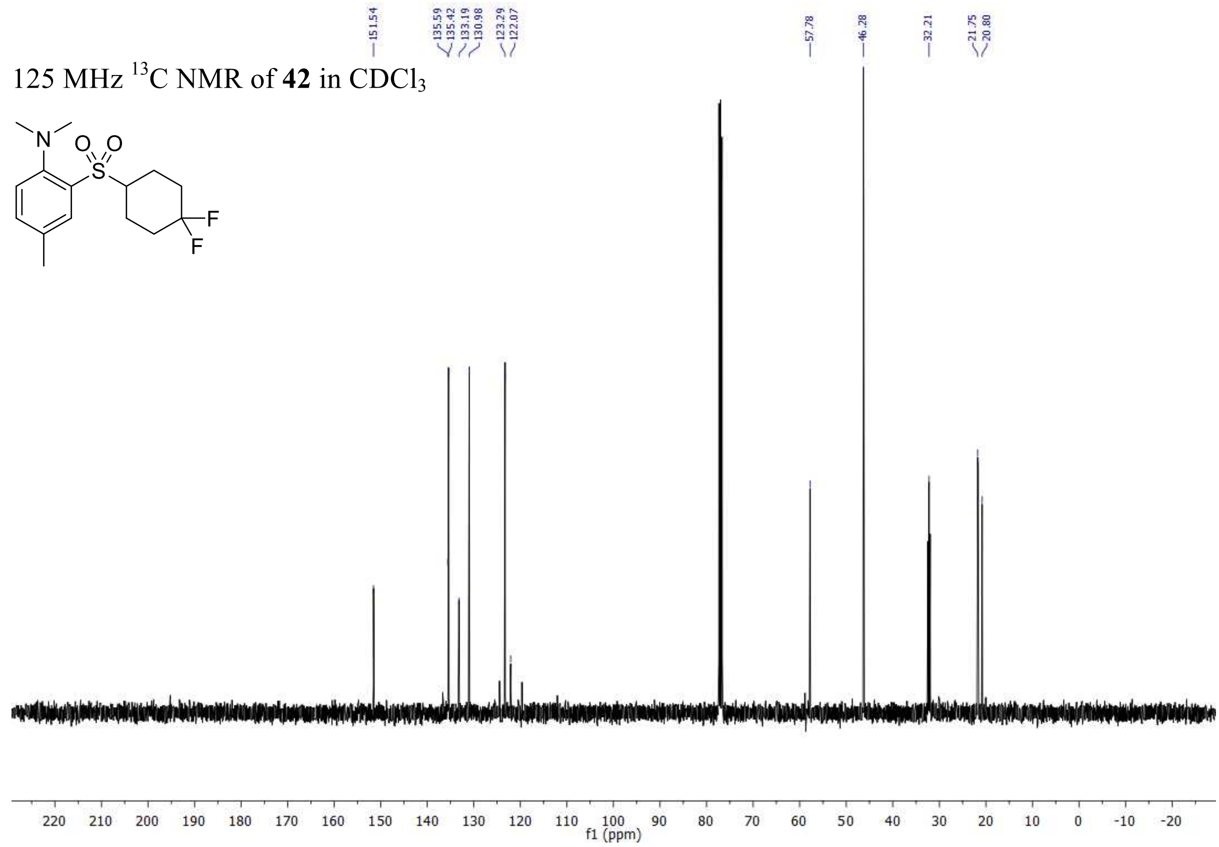

470 MHz ^{19}F NMR of **42** in $CDCl_3$

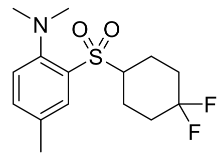

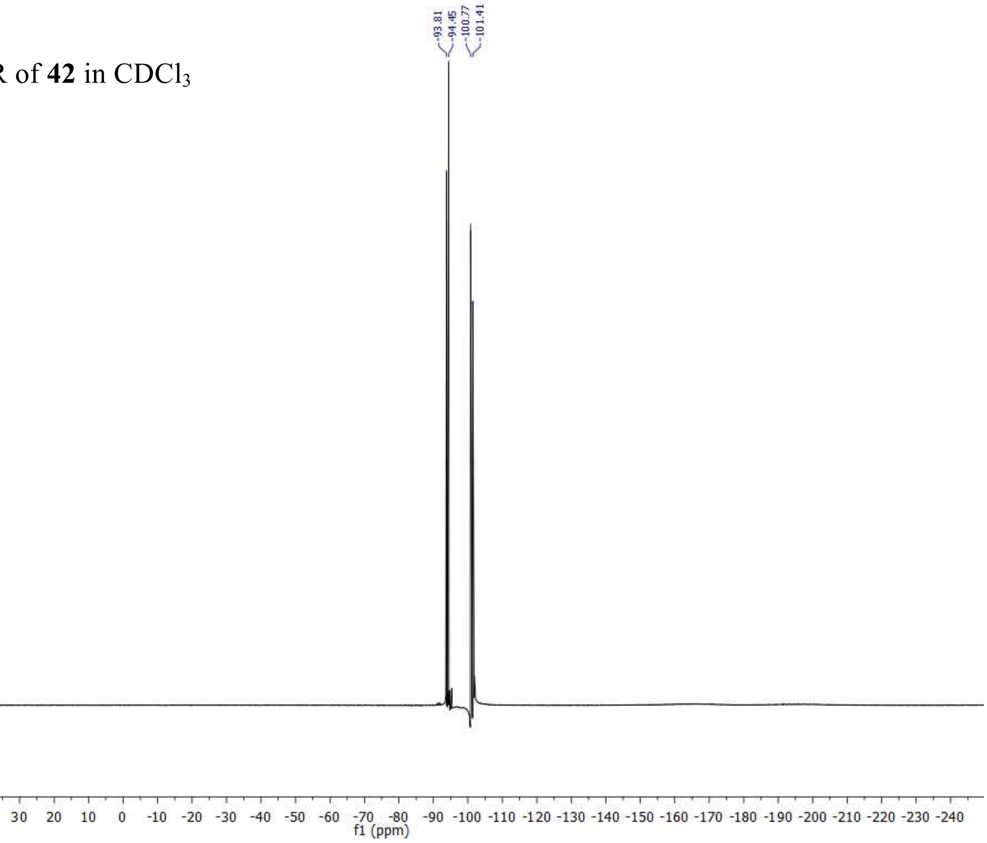

400 MHz ^{1}H NMR of **43** in $CDCl_3$

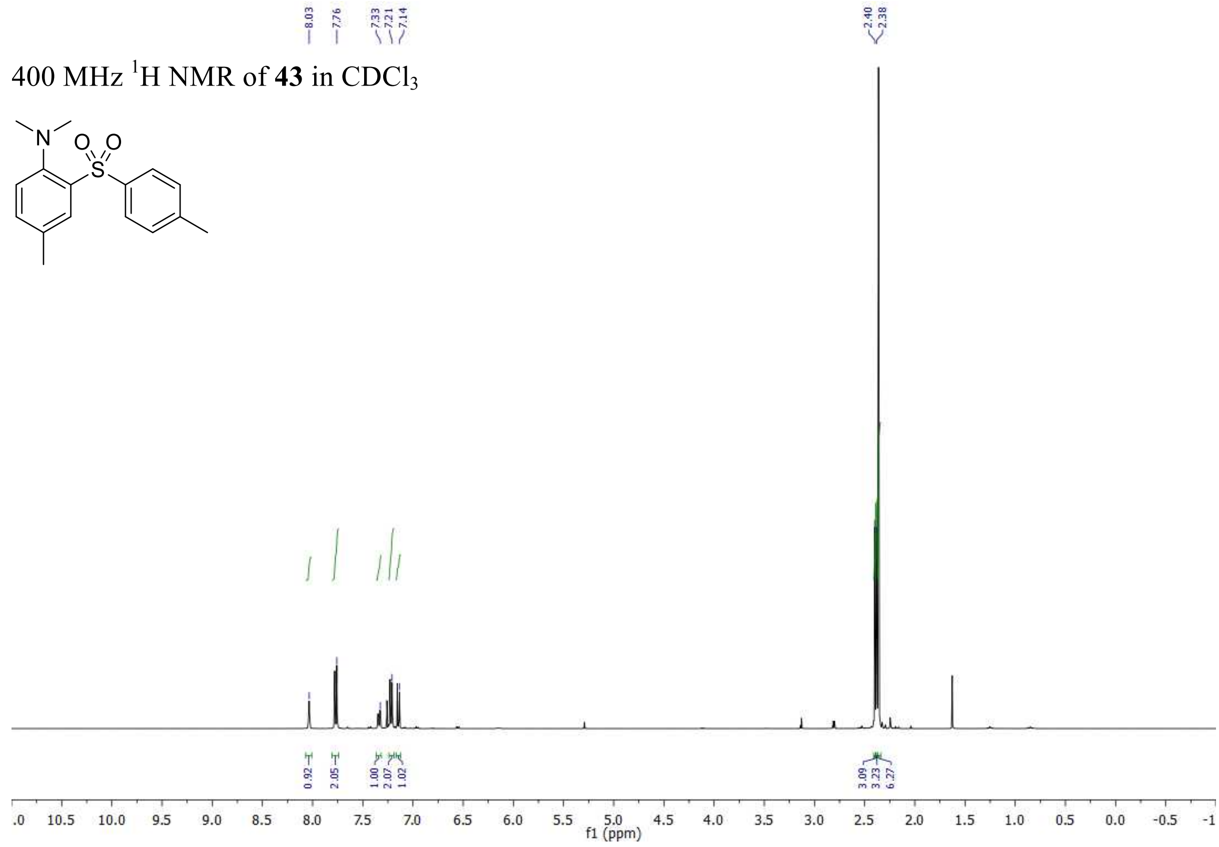

100 MHz ^{13}C NMR of **43** in $CDCl_3$

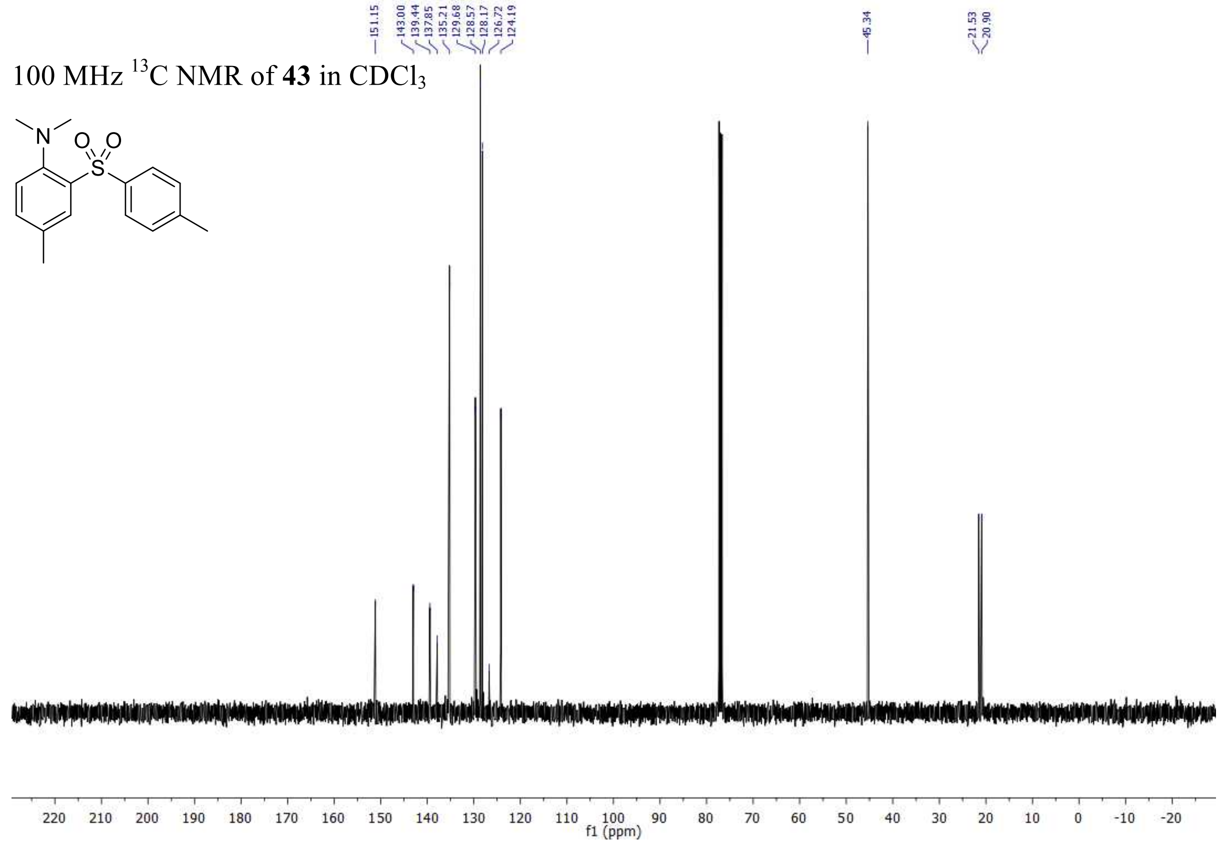

400 MHz ^{1}H NMR of **44** in $CDCl_3$

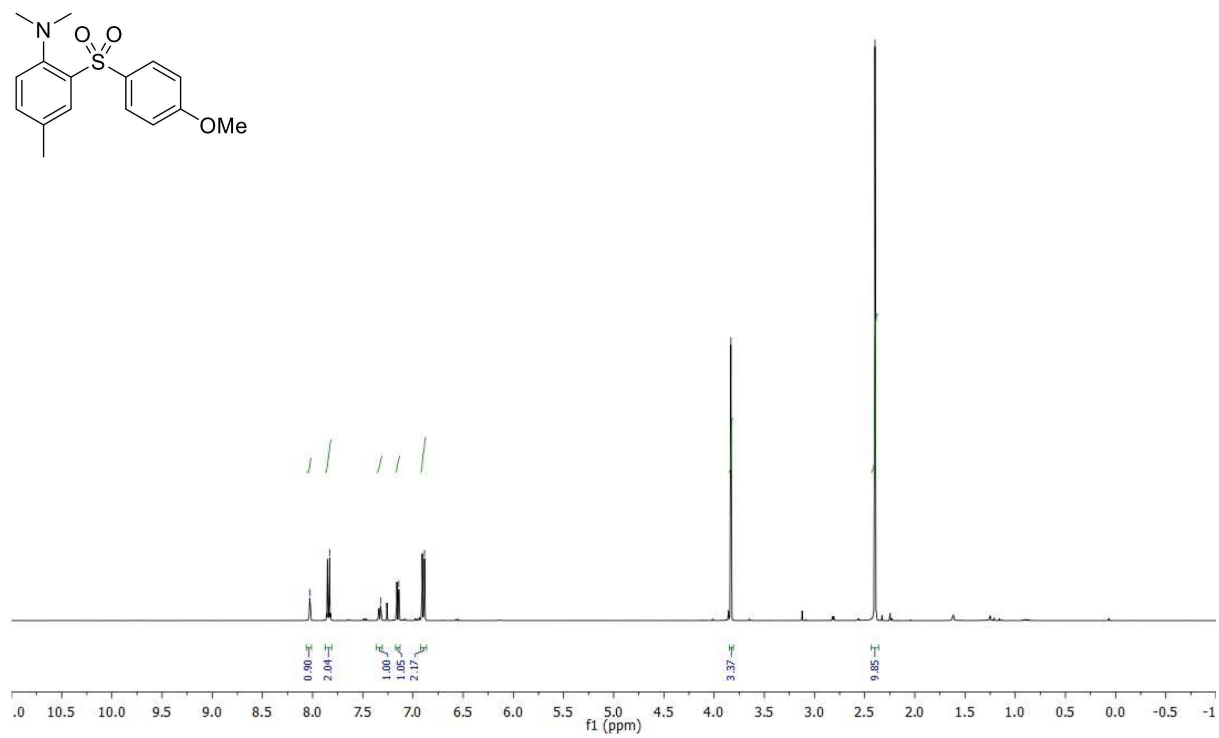

100 MHz ^{13}C NMR of **44** in $CDCl_3$

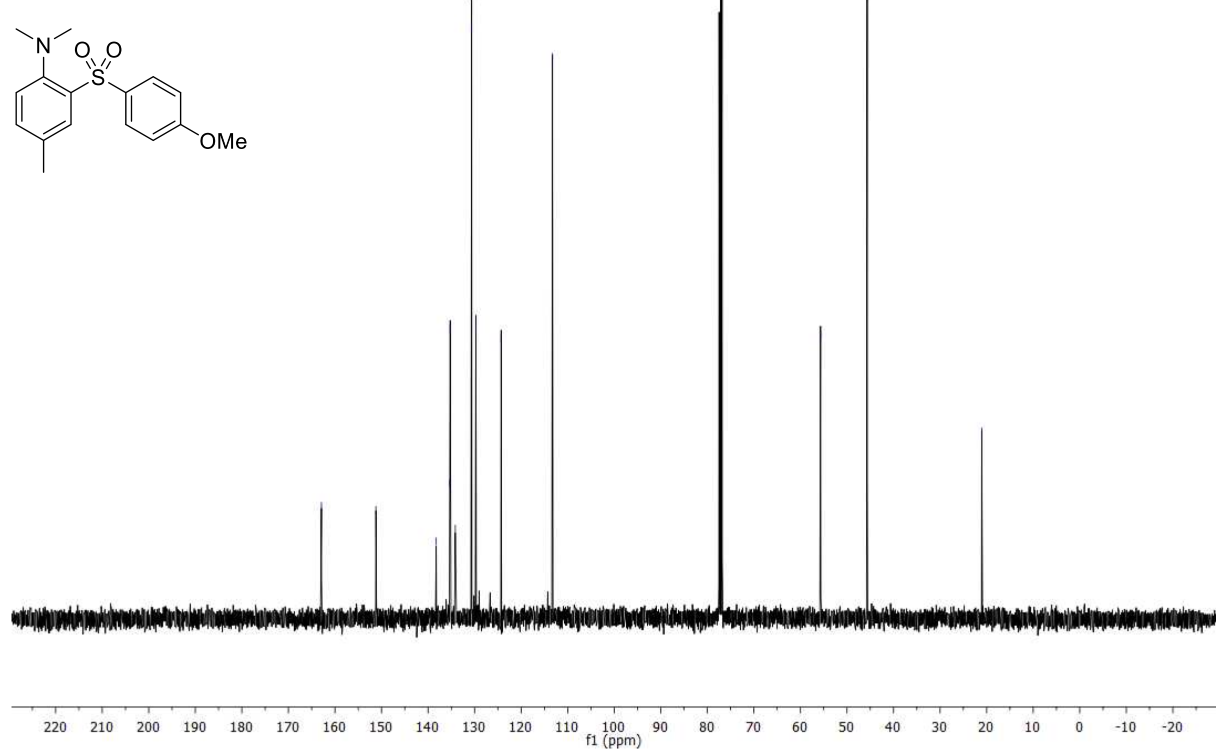

400 MHz ^{1}H NMR of **45** in $CDCl_3$

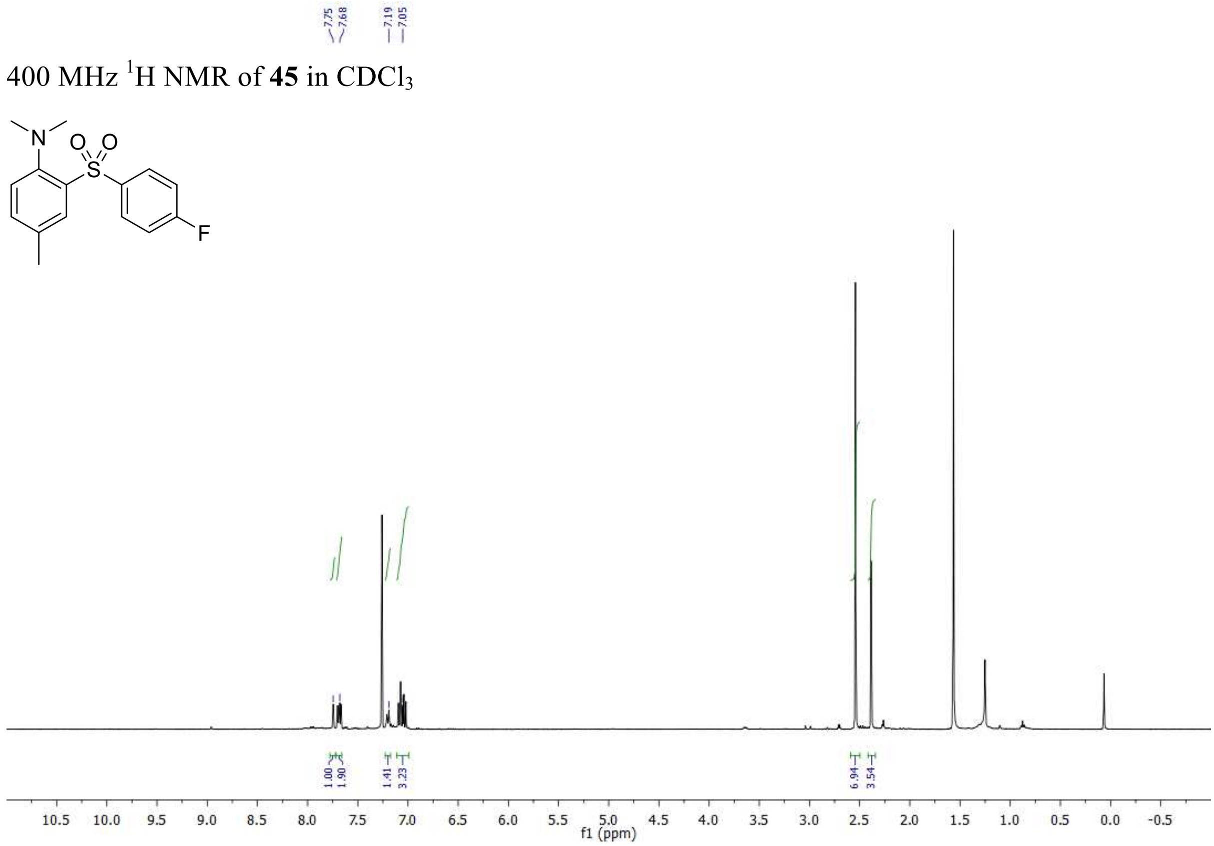

100 MHz ^{13}C NMR of **45** in $CDCl_3$

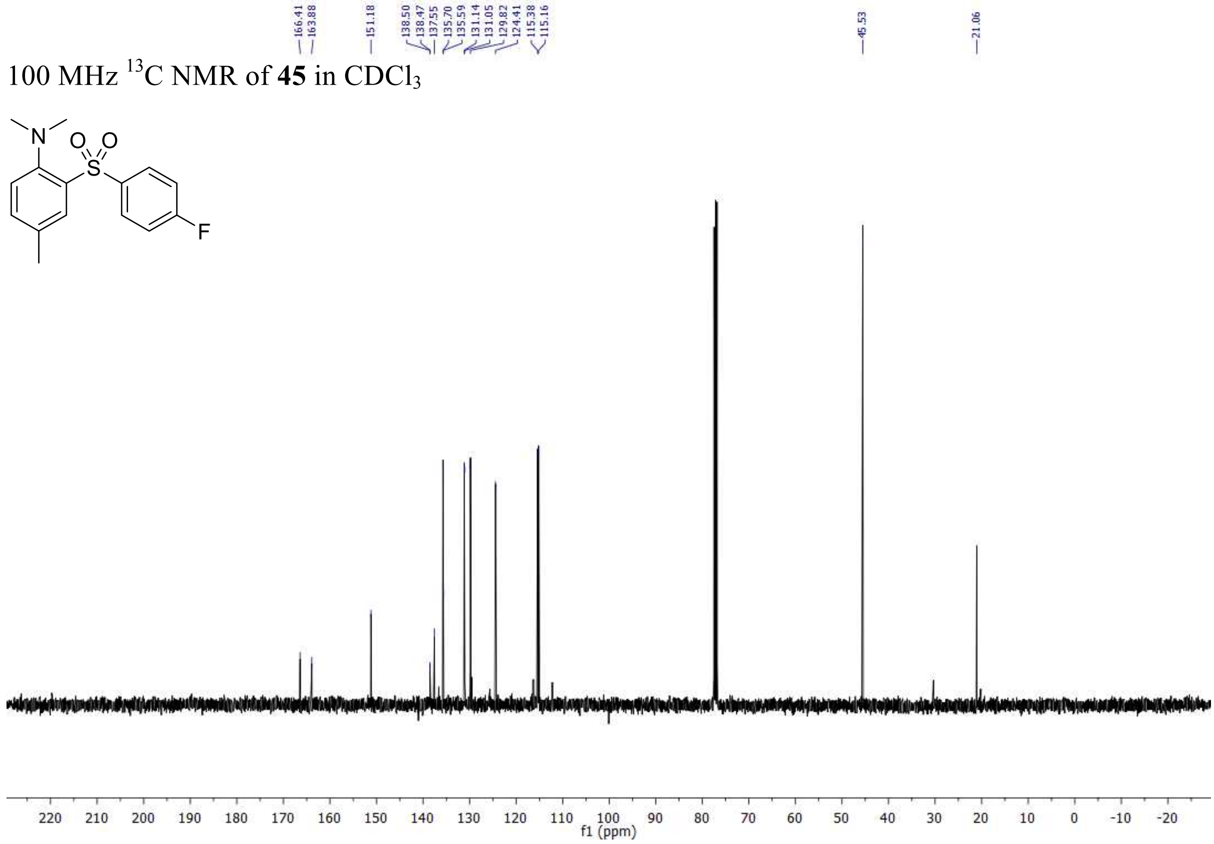

376.5 MHz ^{19}F NMR of **45** in $CDCl_3$

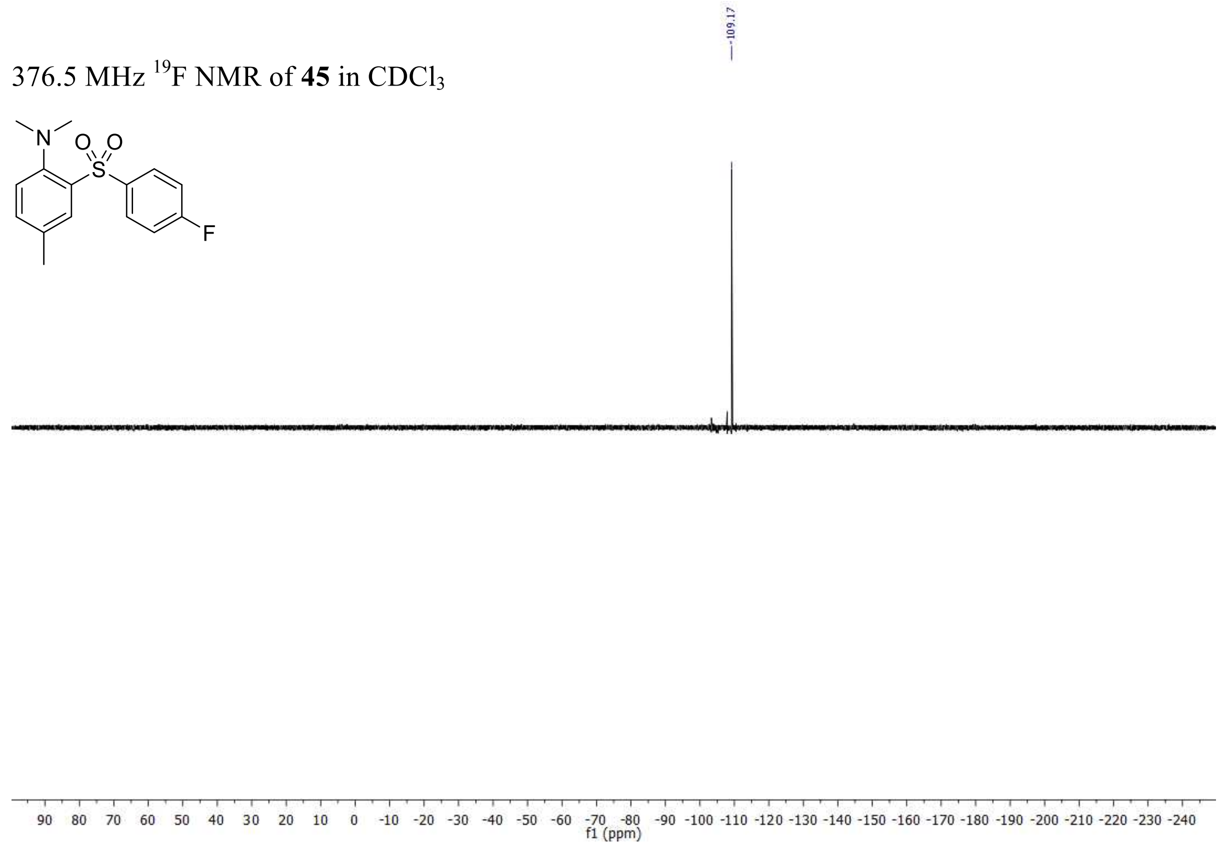

400 MHz ^{1}H NMR of **46** in $CDCl_3$

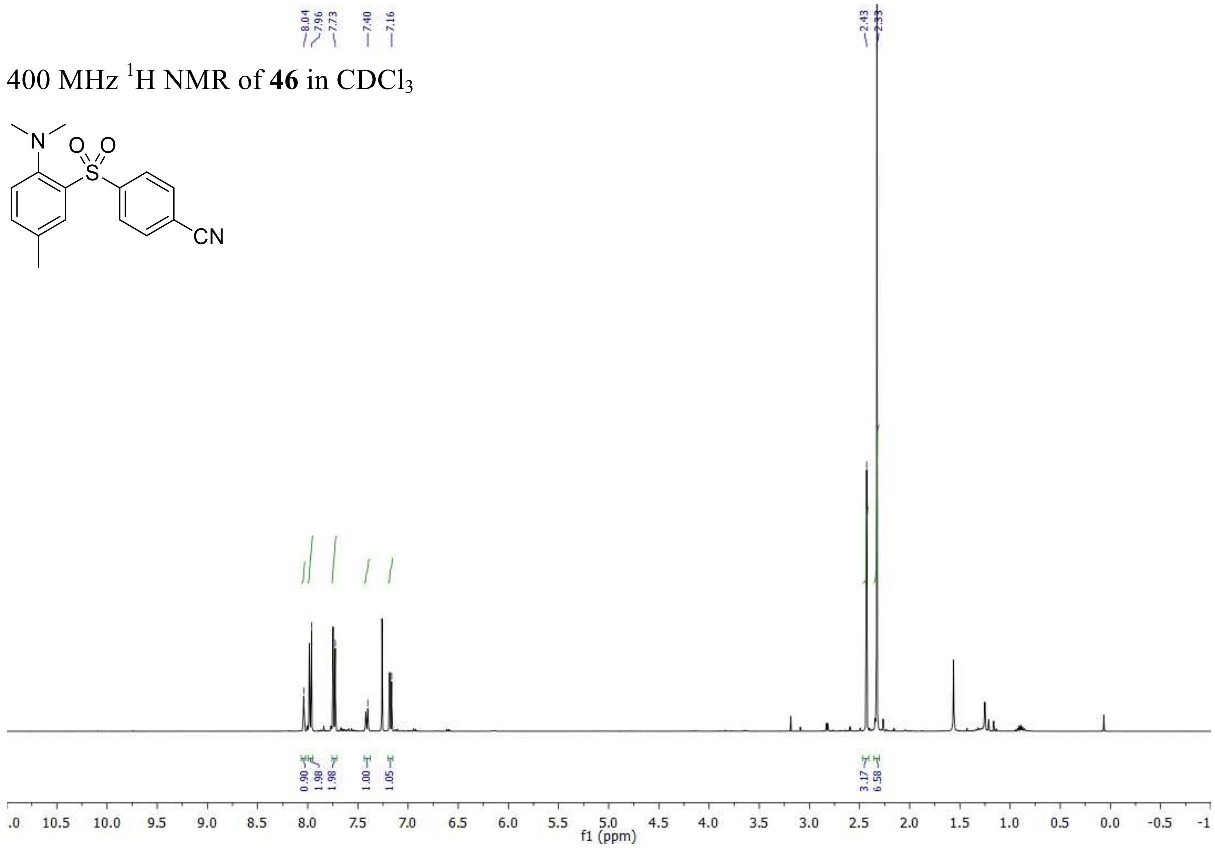

100 MHz ^{13}C NMR of **46** in $CDCl_3$

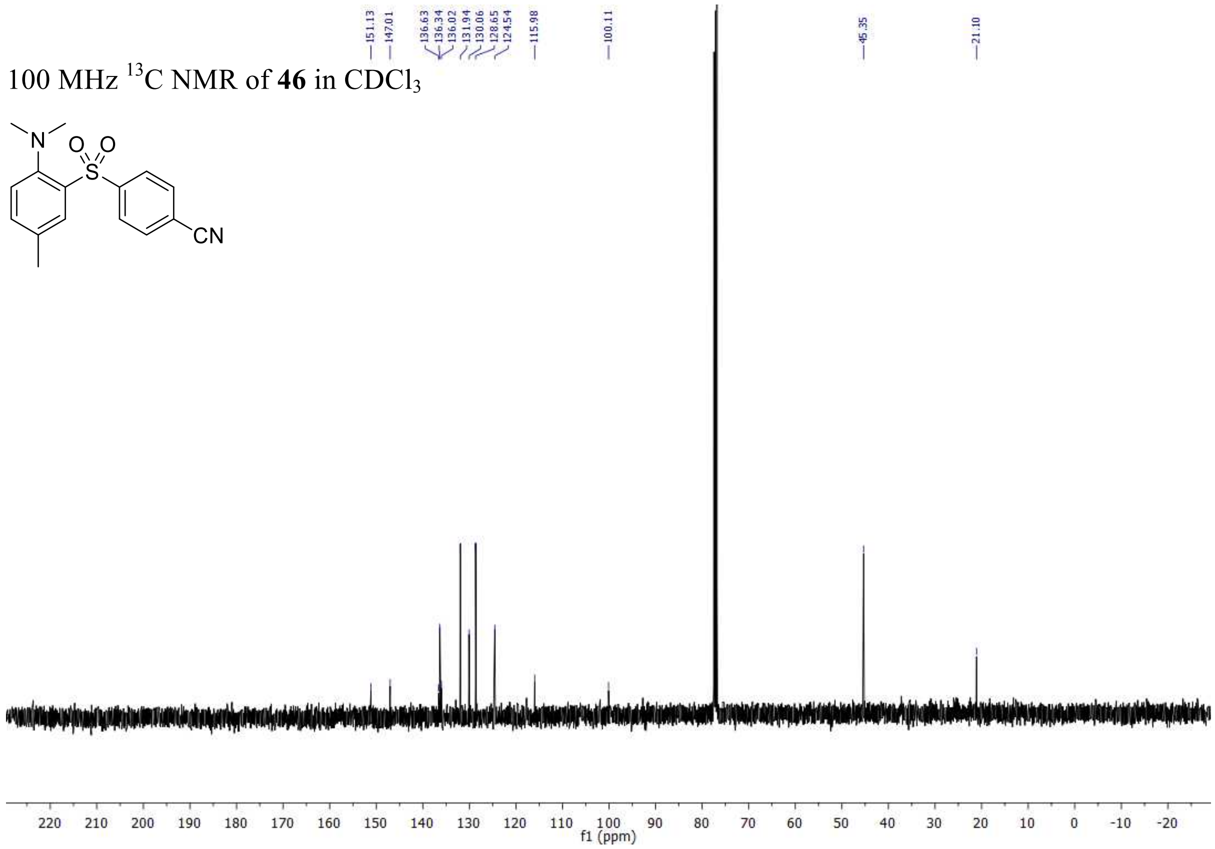

500 MHz ^{1}H NMR of **47a** in $CDCl_3$

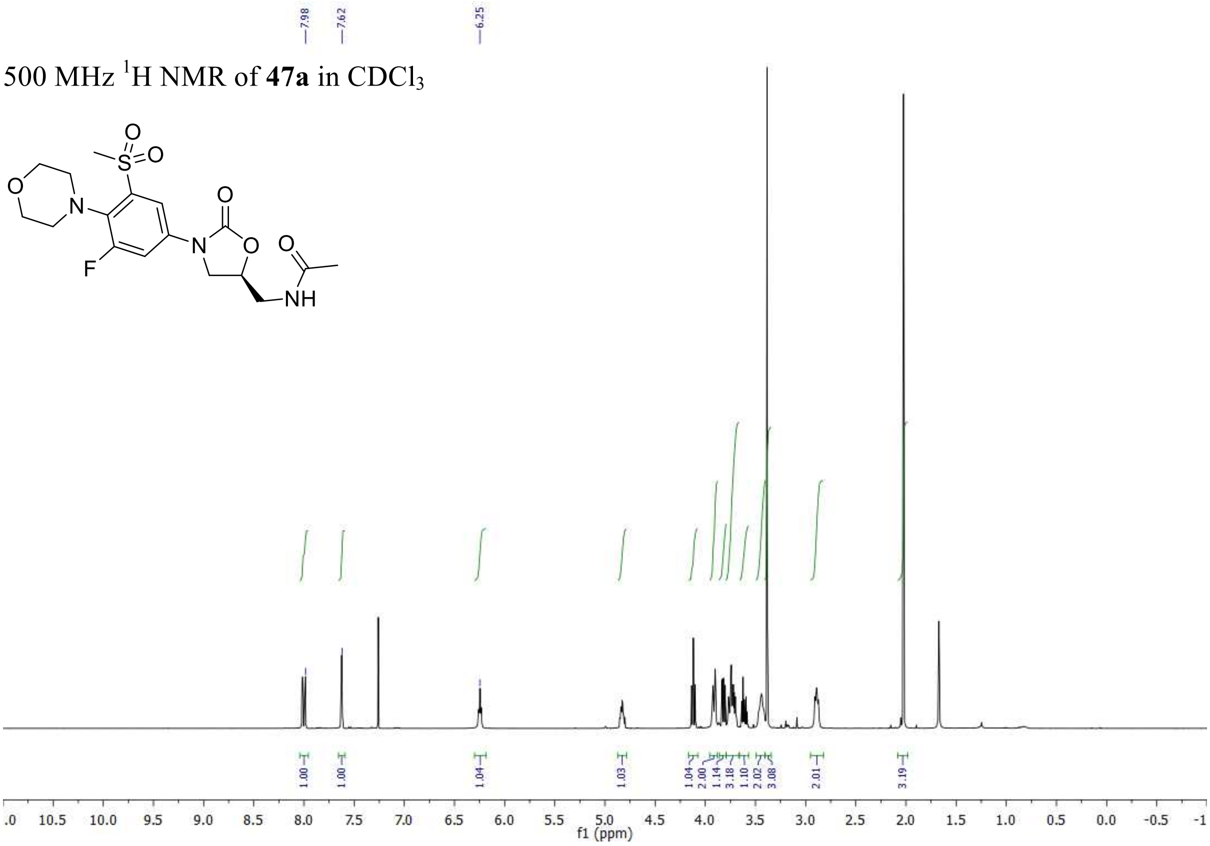

125 MHz ^{13}C NMR of **47a** in $CDCl_3$

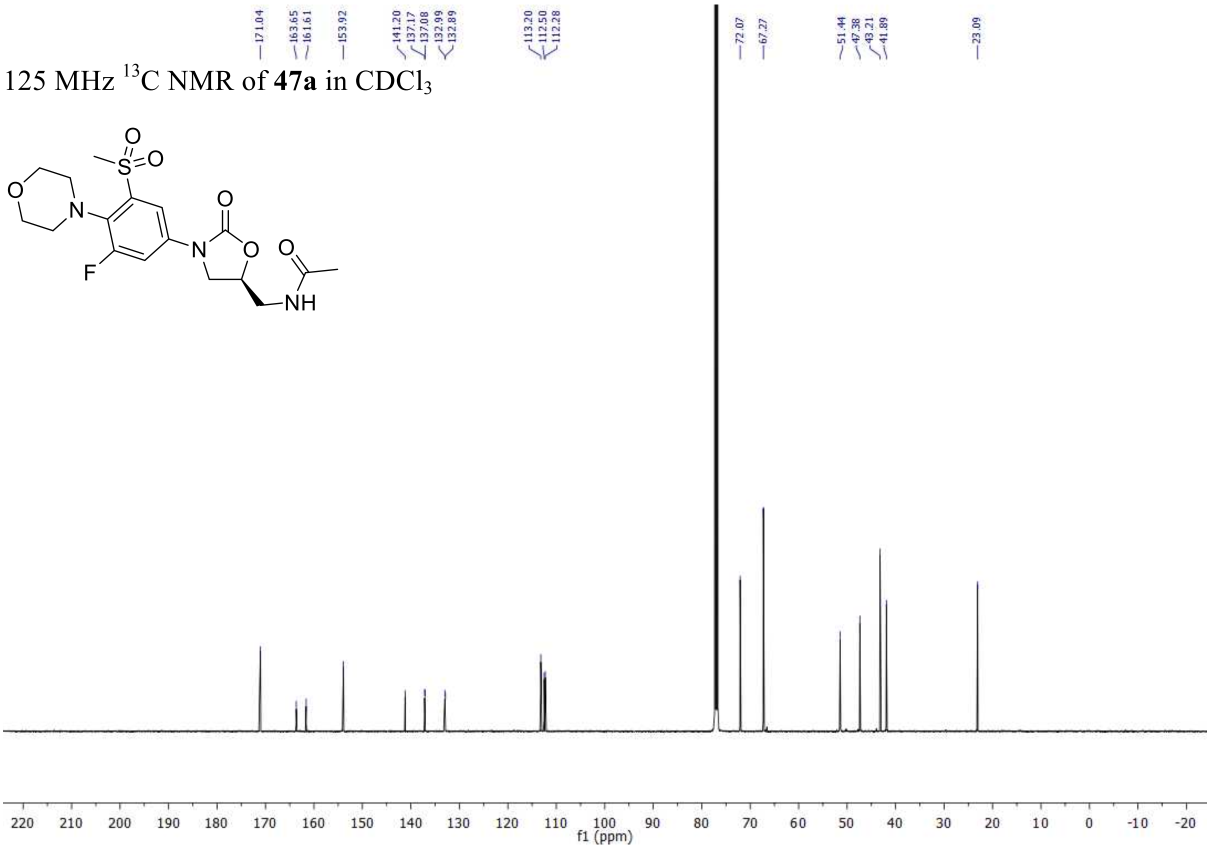

500 MHz ^{1}H NMR of **47b** in $CDCl_3$

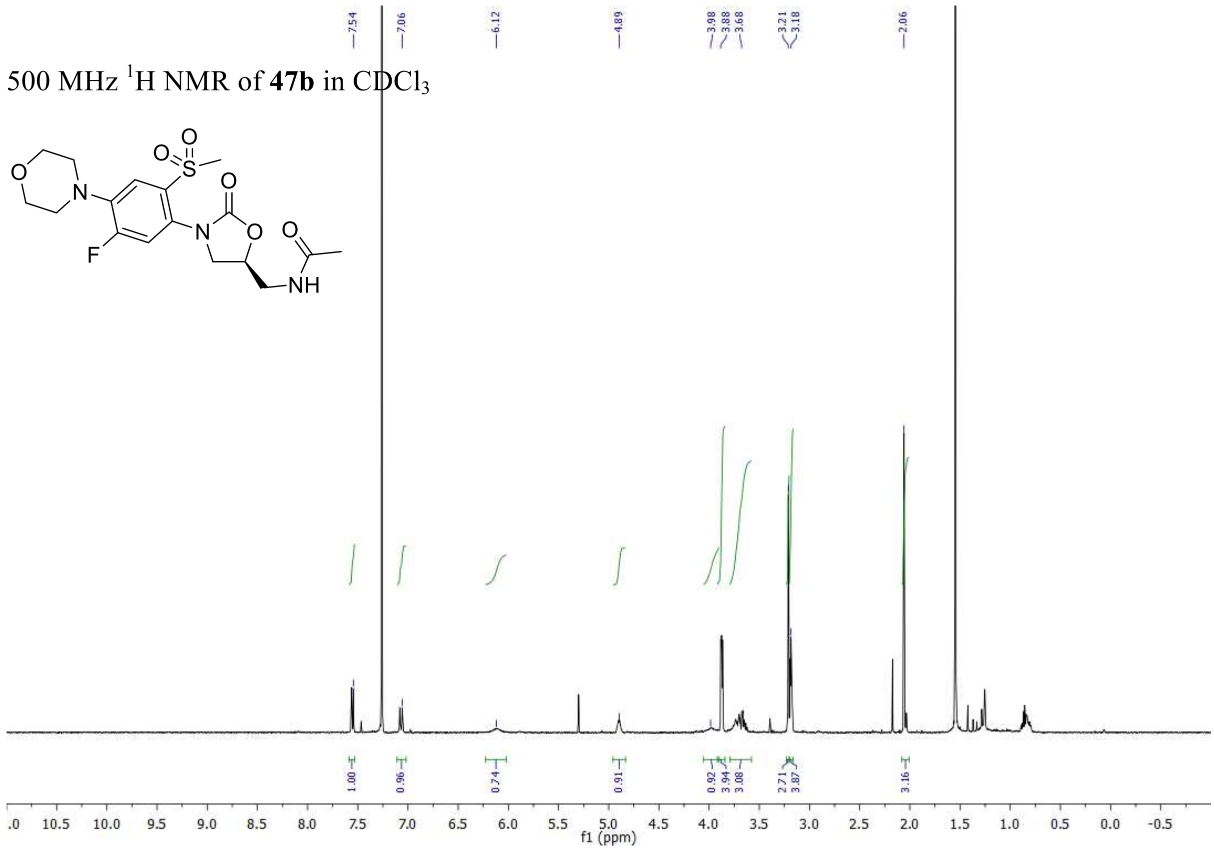

125 MHz ^{13}C NMR of **47b** in $CDCl_3$

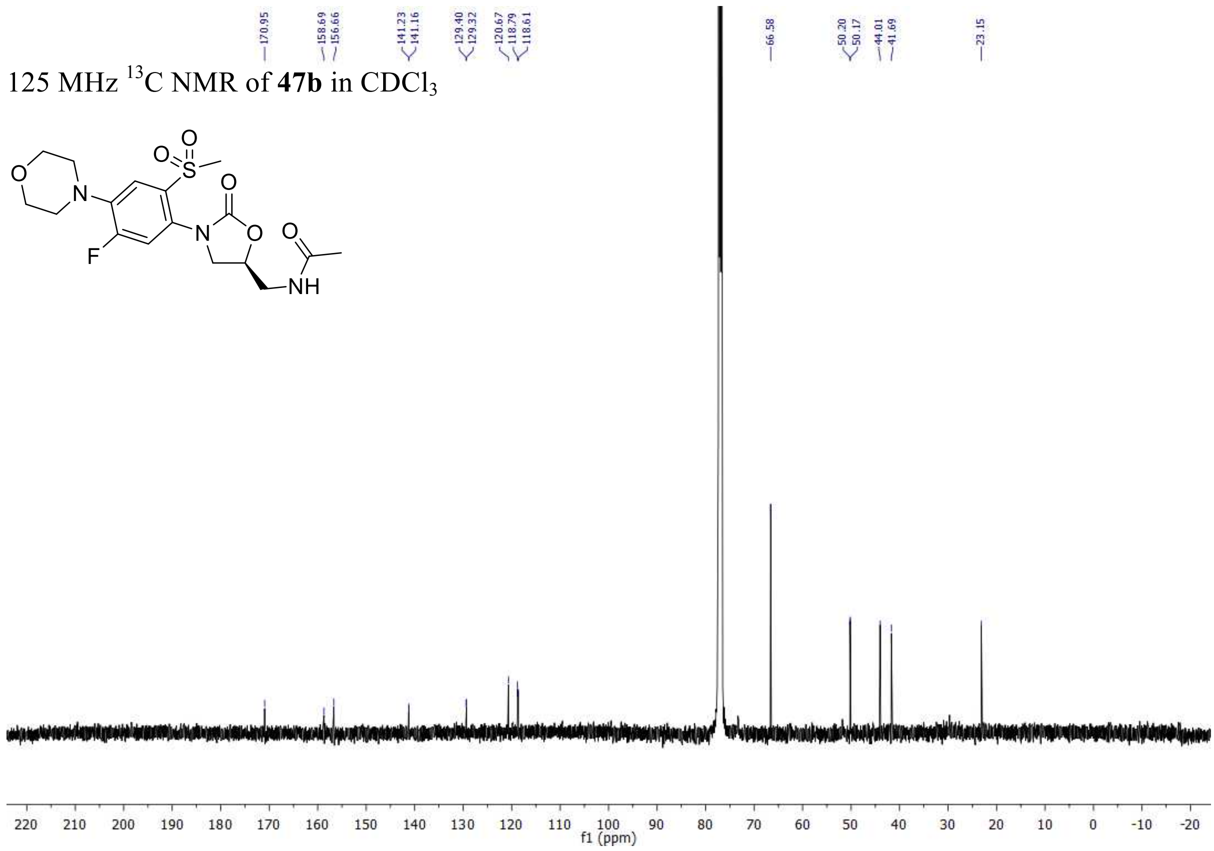

400 MHz ^{1}H NMR of **48** in $CDCl_3$

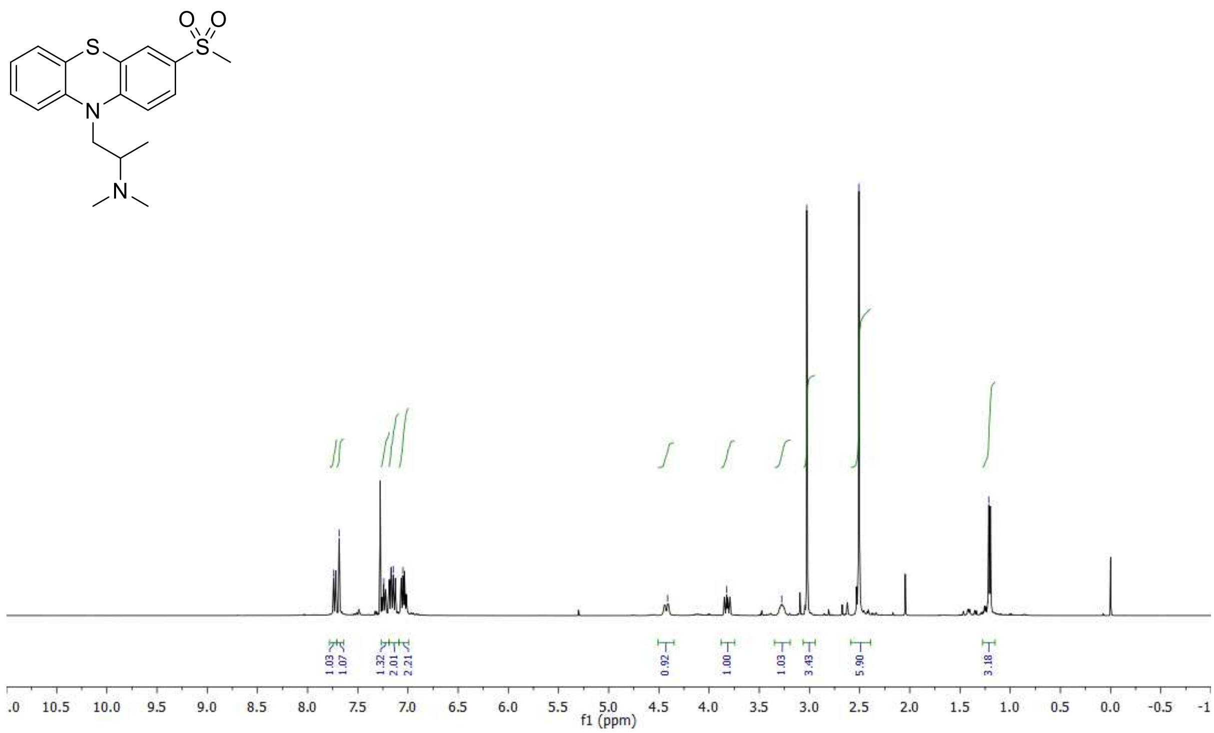

100 MHz ^{13}C NMR of **48** in $CDCl_3$

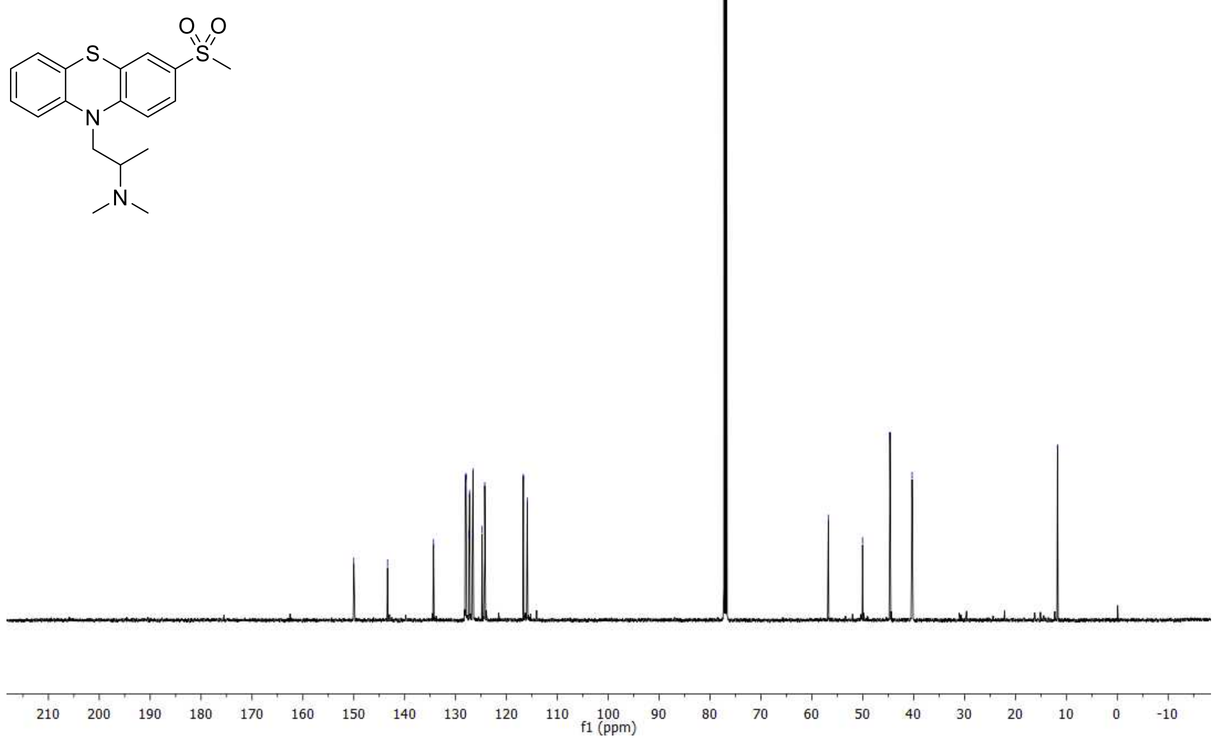

400 MHz ^{1}H NMR of **49a** in $CDCl_3$

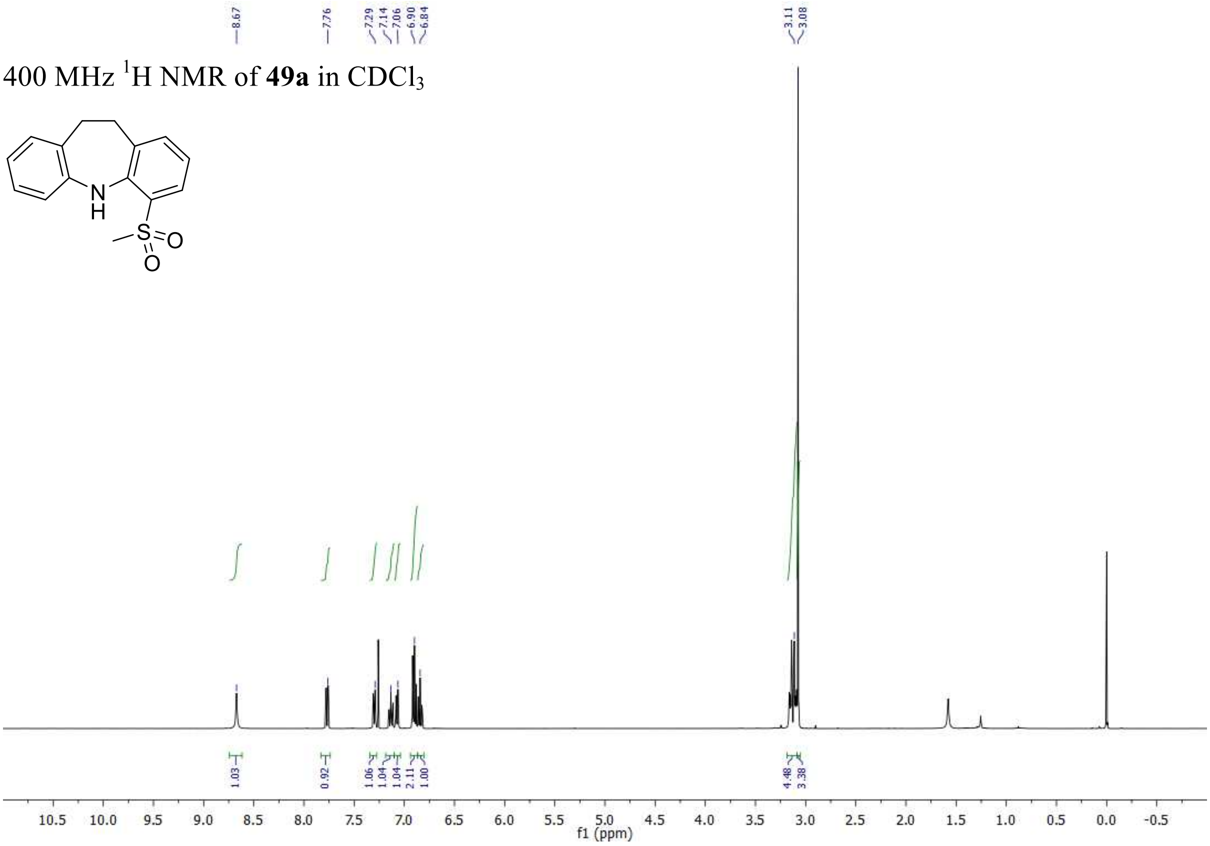

125 MHz ^{13}C NMR of **49a** in $CDCl_3$

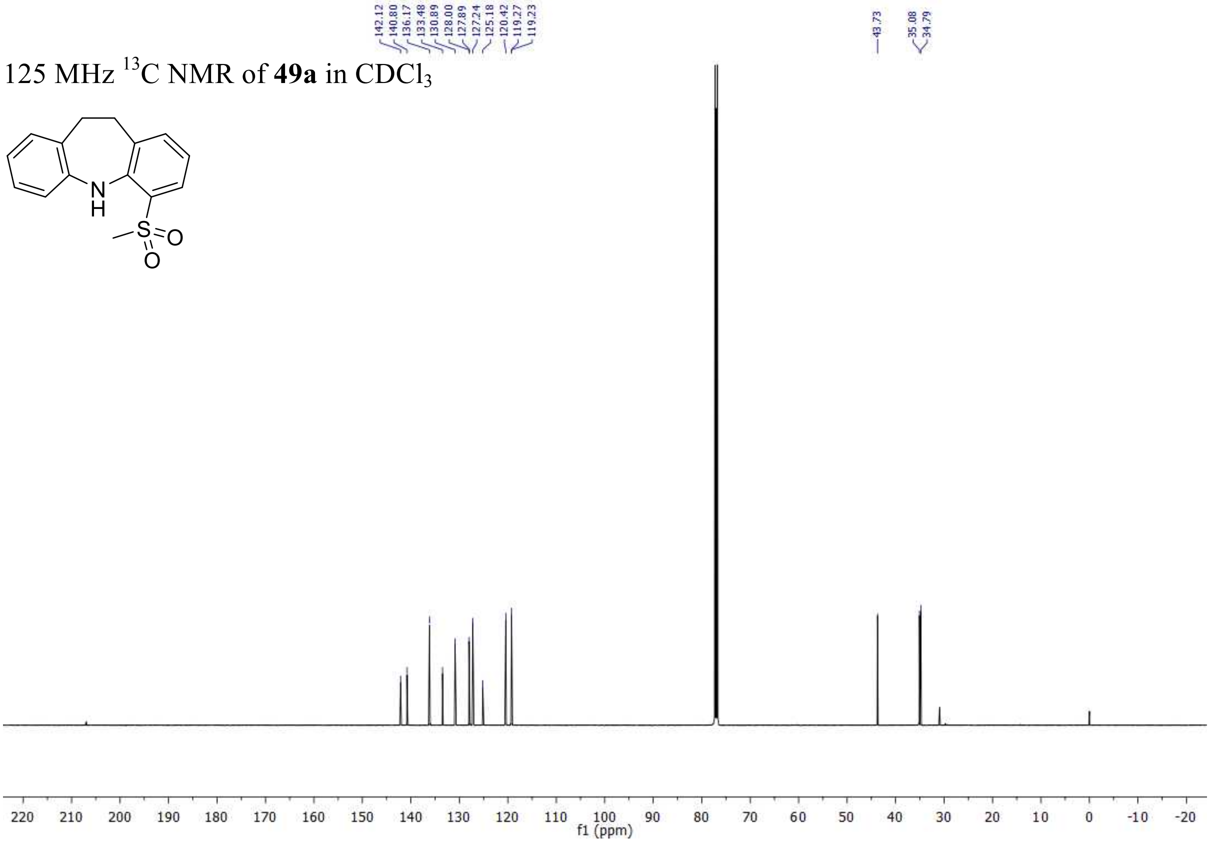

400 MHz ^{1}H NMR of **49b** in $CDCl_3$

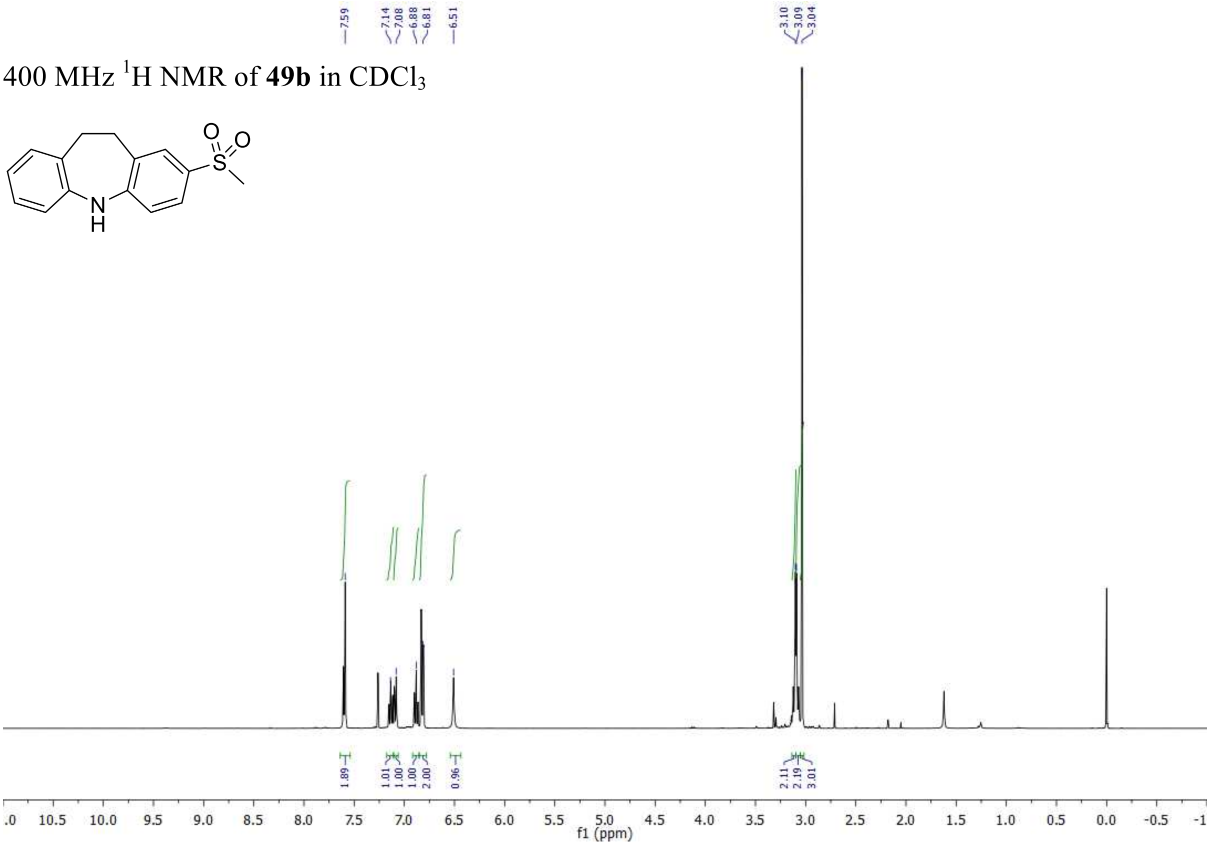

100 MHz ^{13}C NMR of **49b** in $CDCl_3$

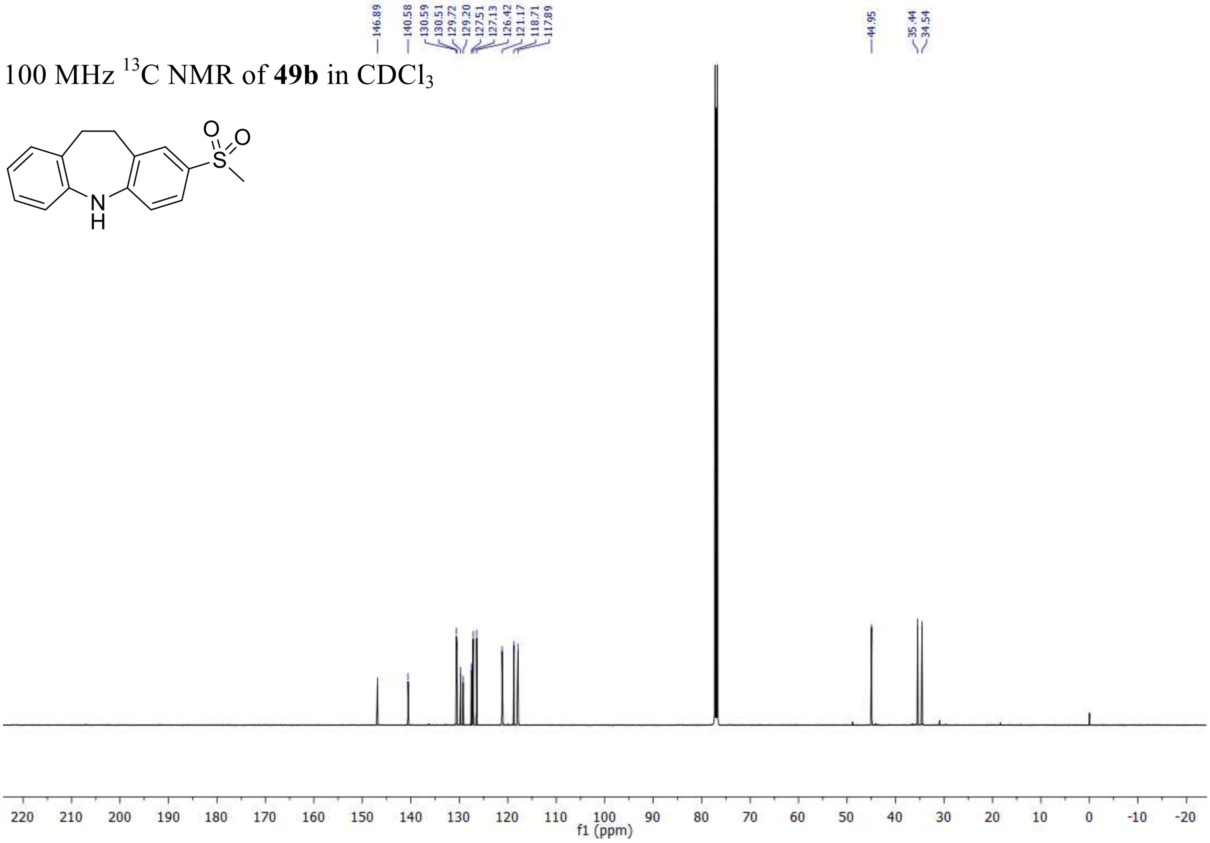

400 MHz ^{1}H NMR of **50** in CD_3OD

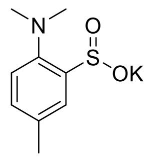

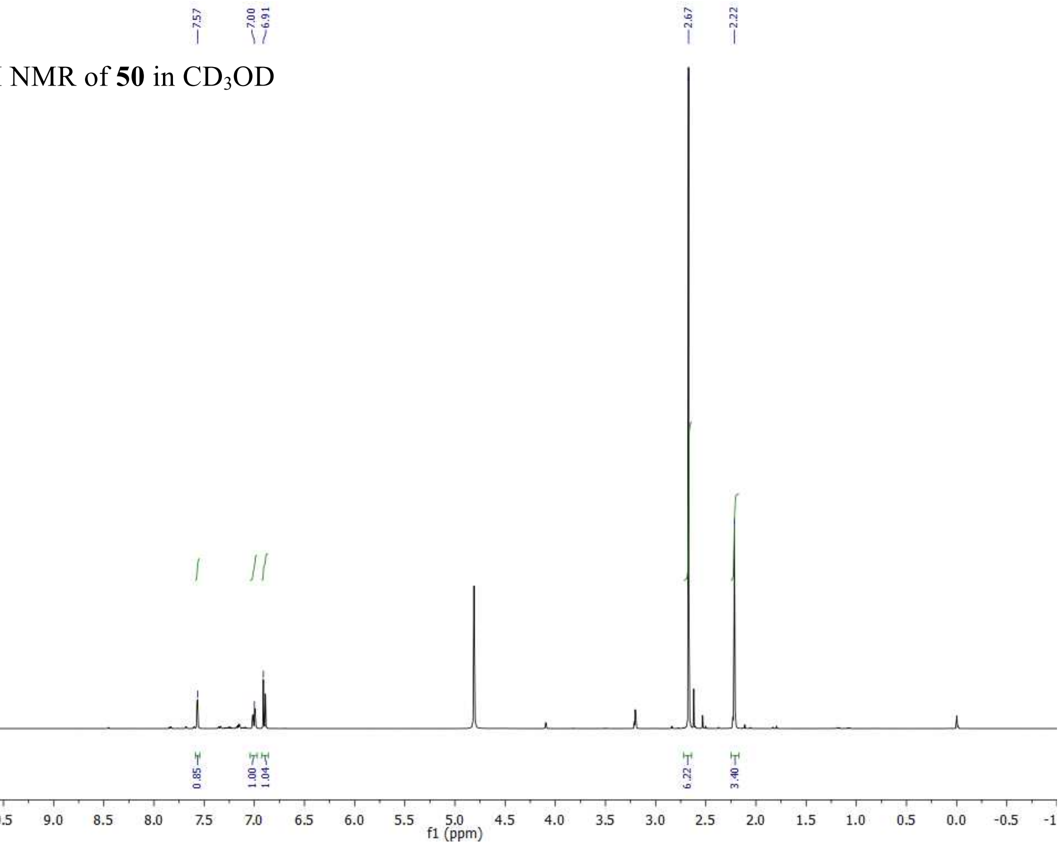

100 MHz ^{13}C NMR of **50** in CD_3OD

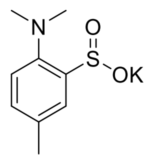

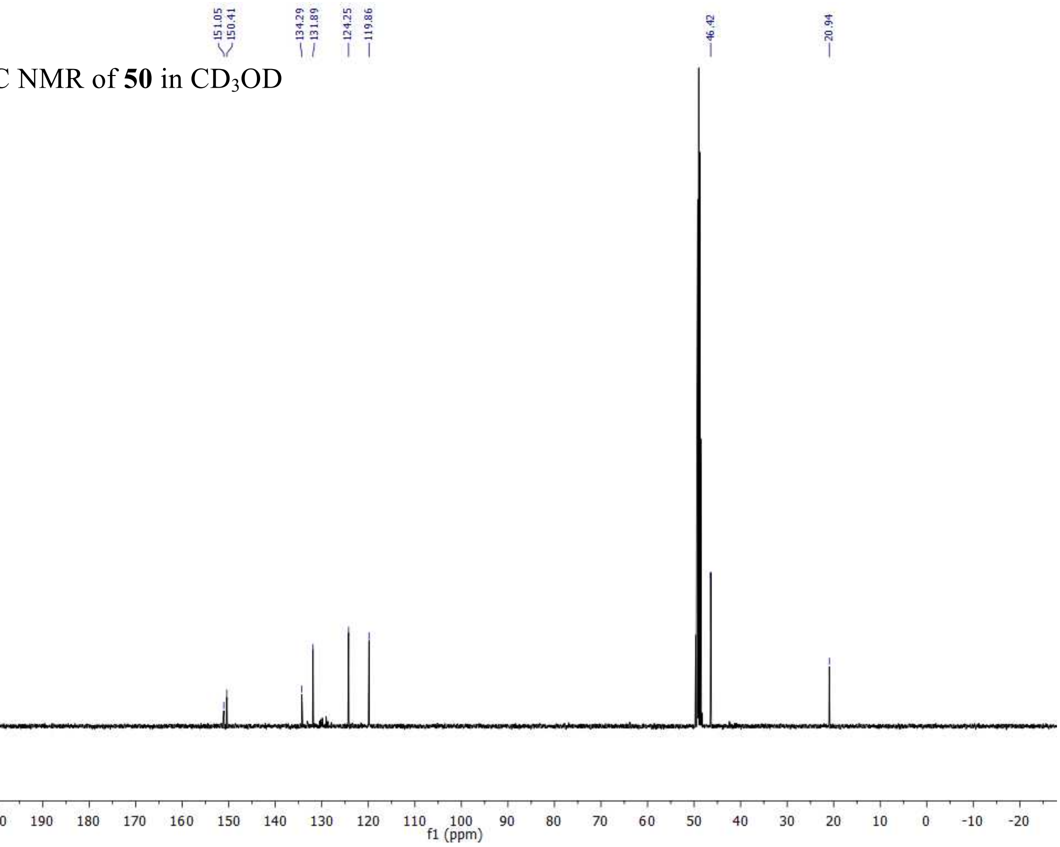

400 MHz ^{1}H NMR of **51** in $CDCl_3$

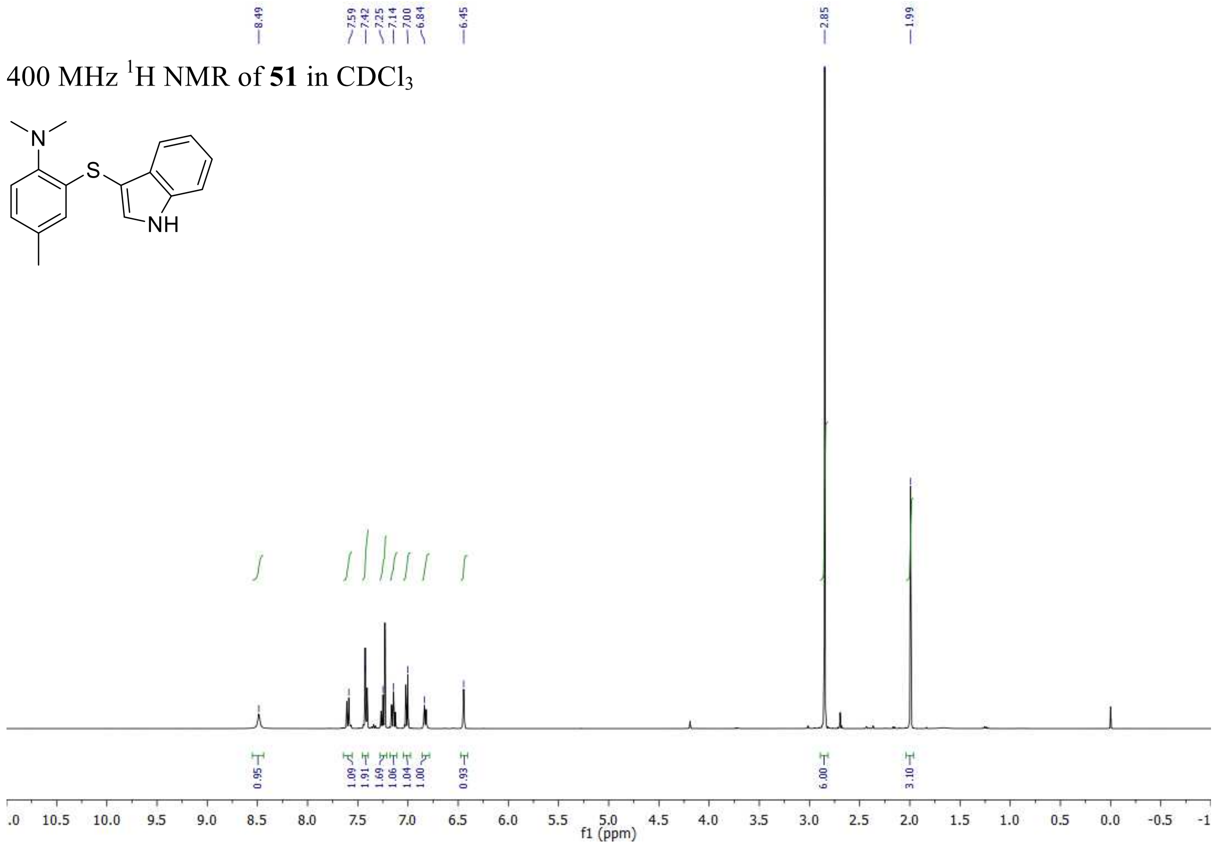

100 MHz ^{13}C NMR of **51** in $CDCl_3$

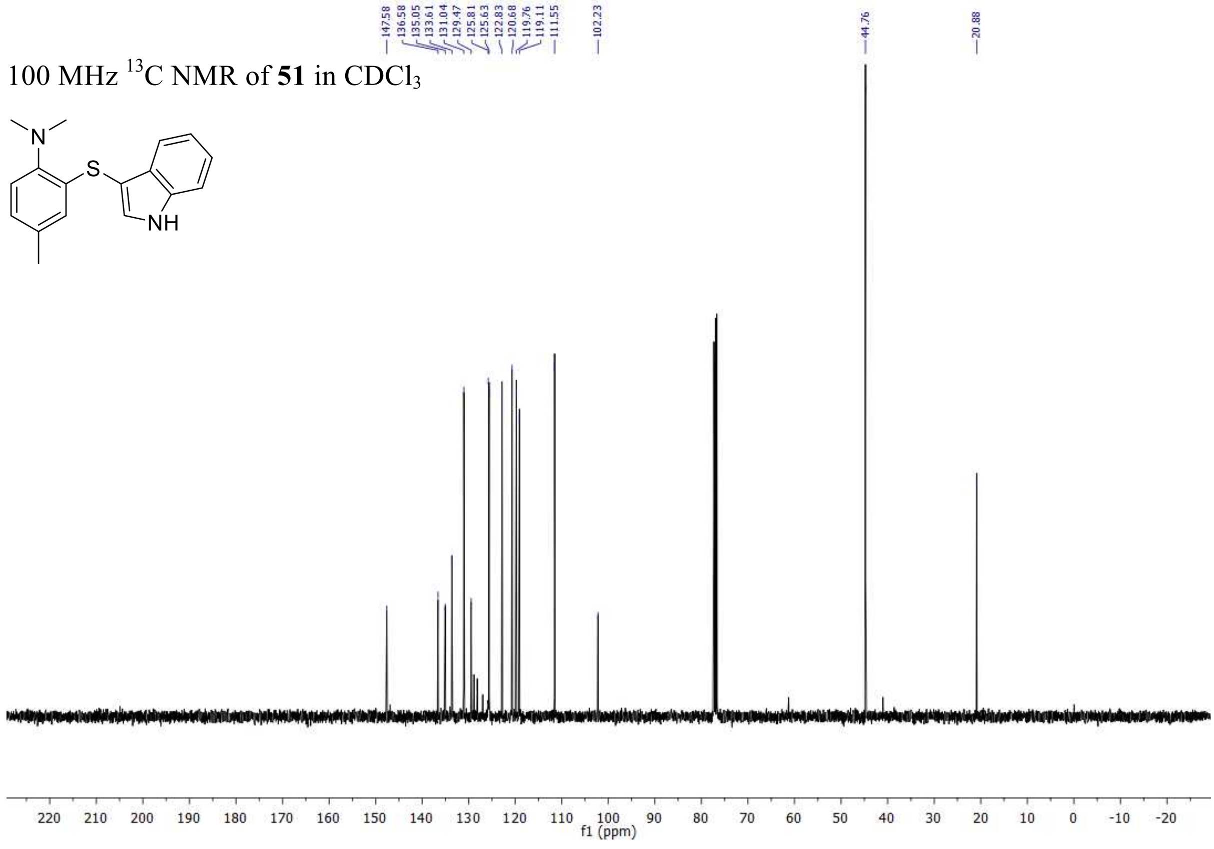

400 MHz ^{1}H NMR of **52** in $CDCl_3$

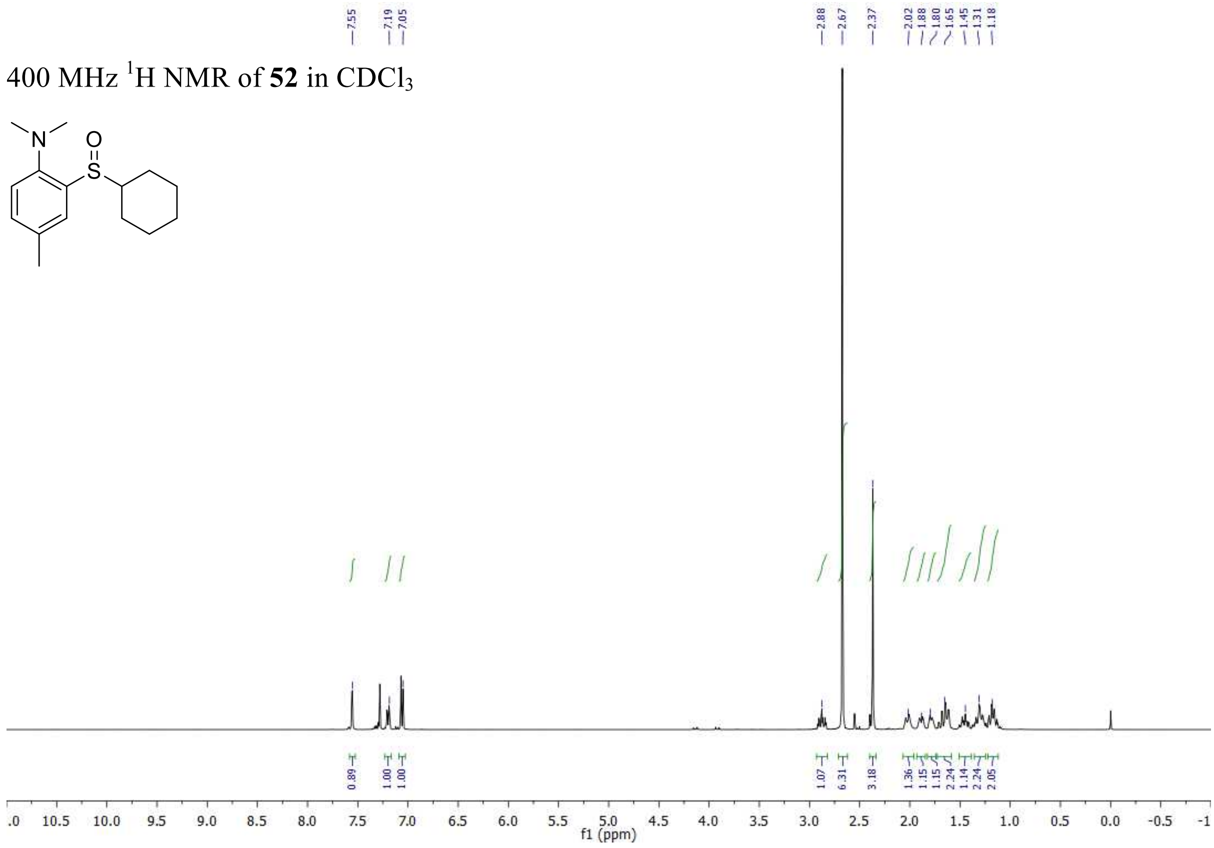

100 MHz ^{13}C NMR of **52** in $CDCl_3$

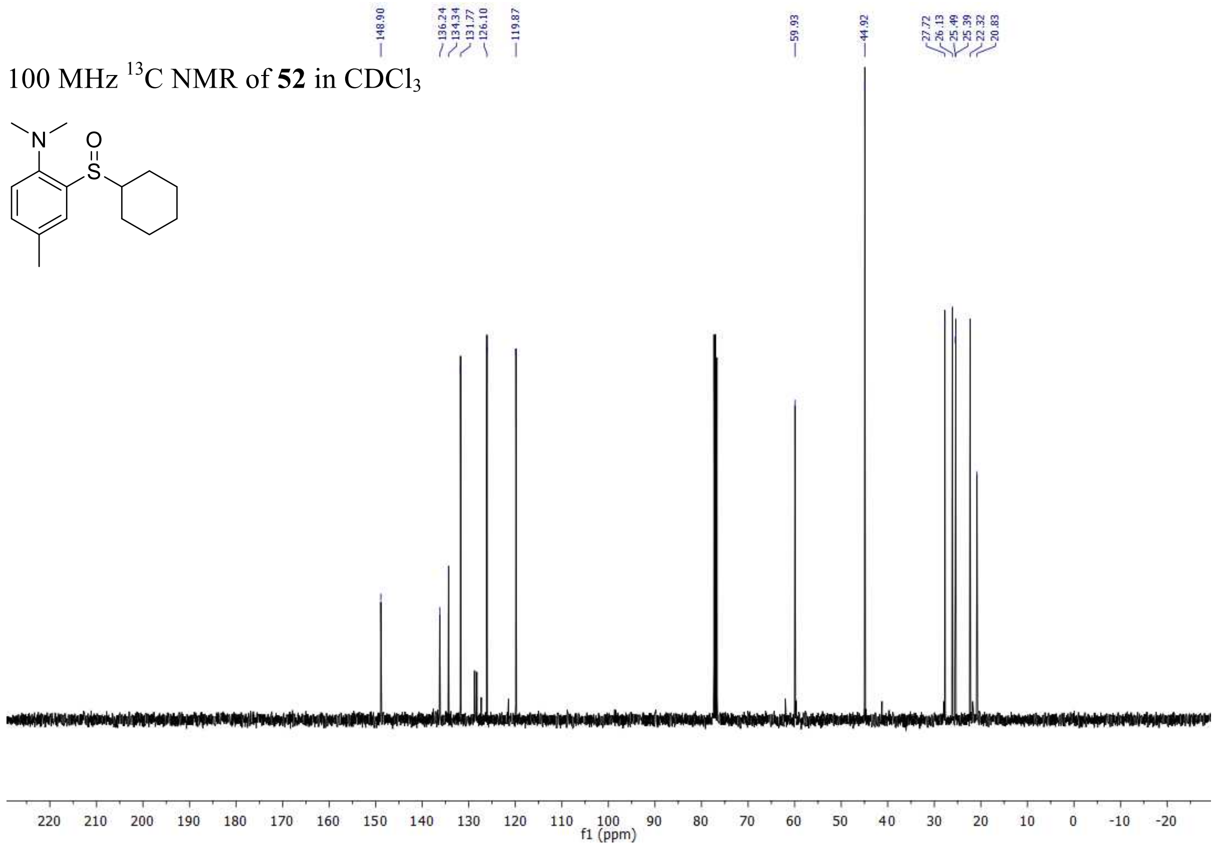

400 MHz ^{1}H NMR of **53** in $CDCl_3$

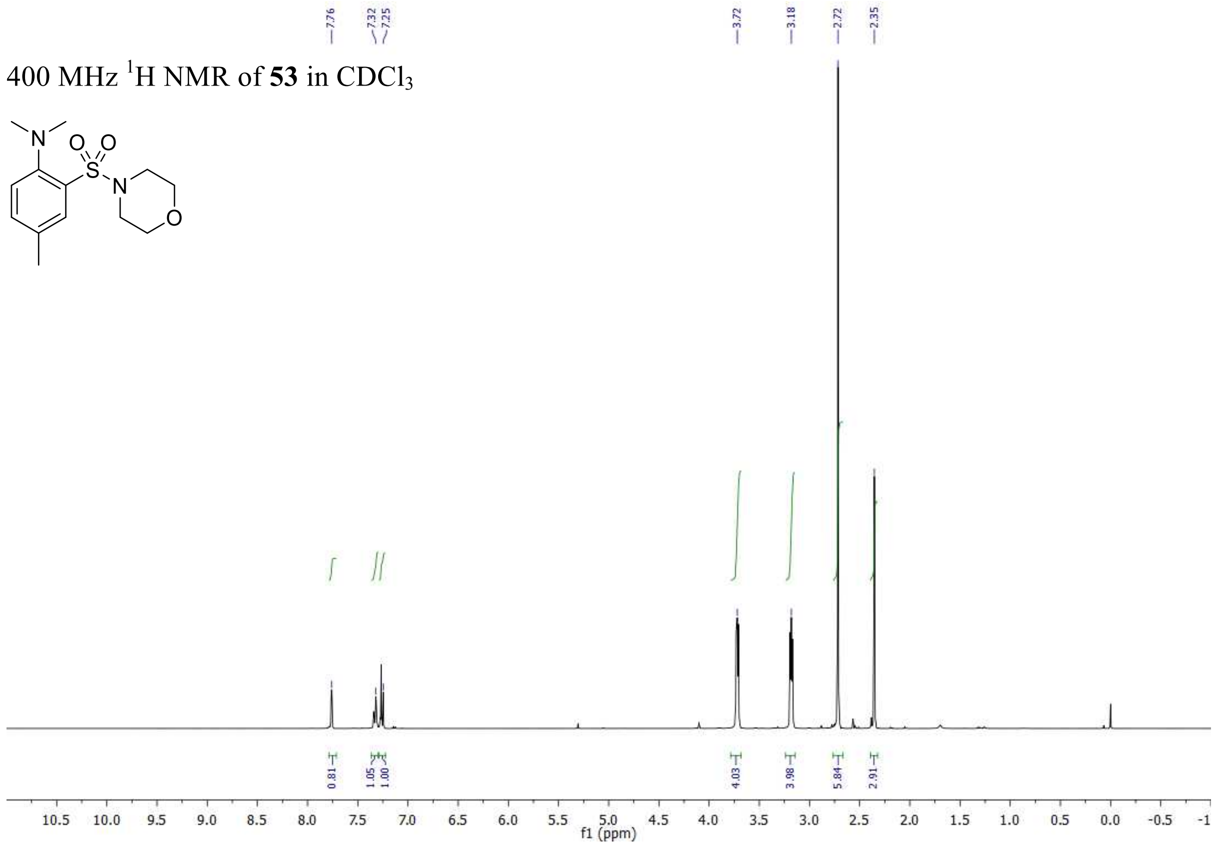

100 MHz ^{13}C NMR of **53** in $CDCl_3$

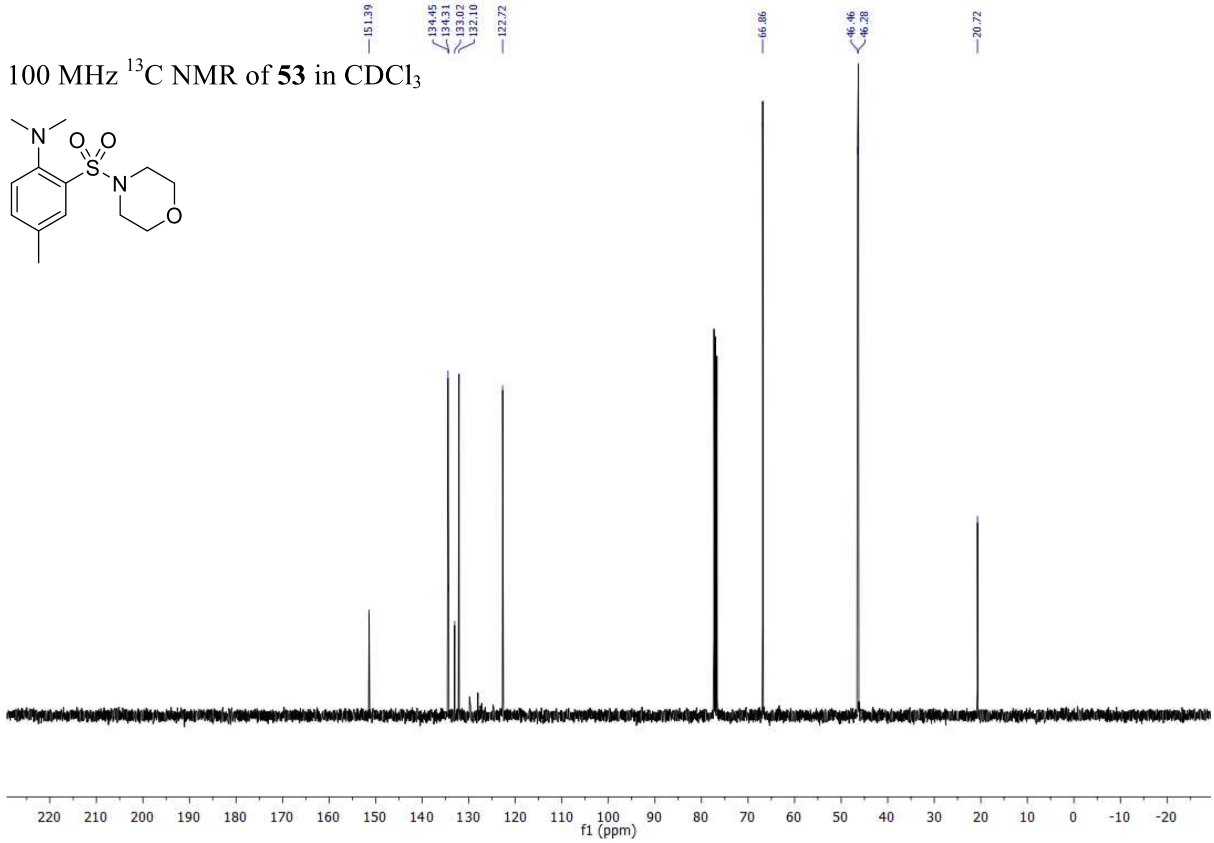

400 MHz ^{1}H NMR of **54** in CD_3OD

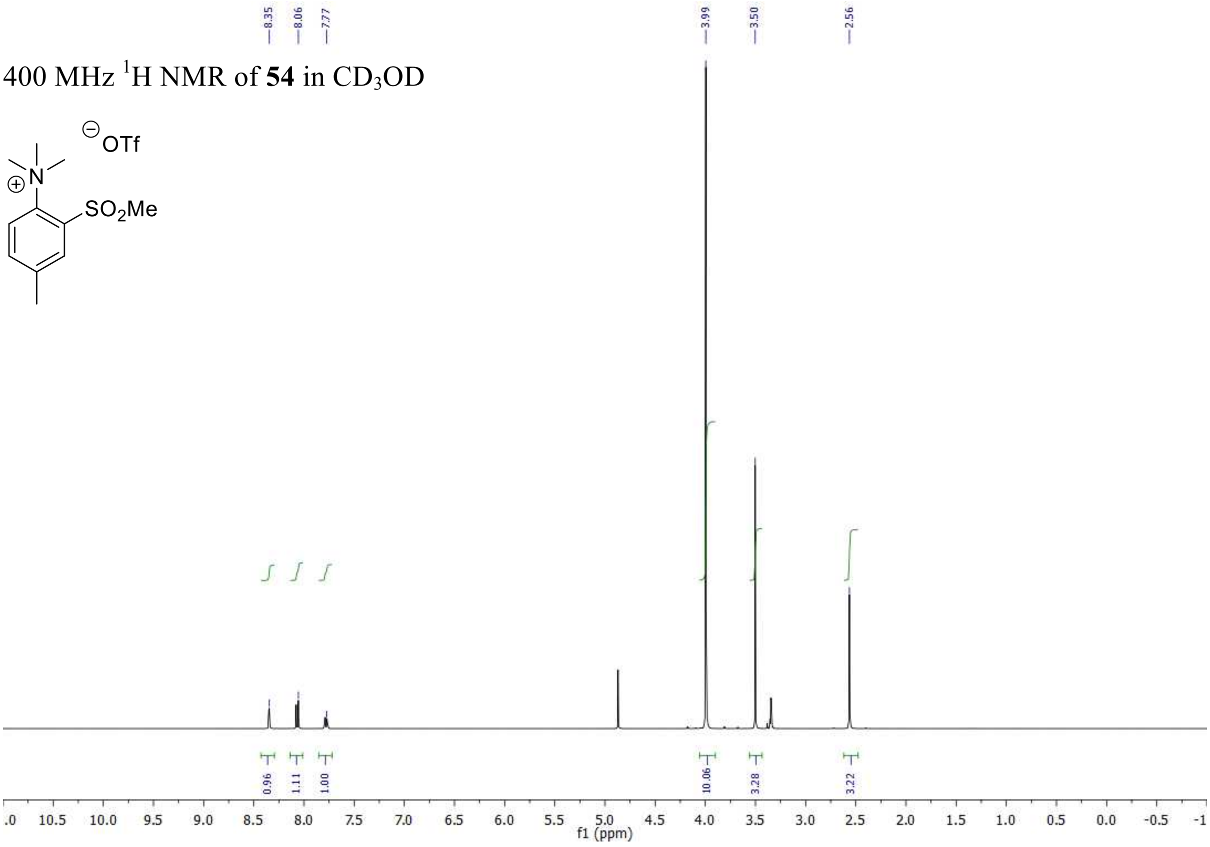

100 MHz ^{13}C NMR of **54** in CD_3OD

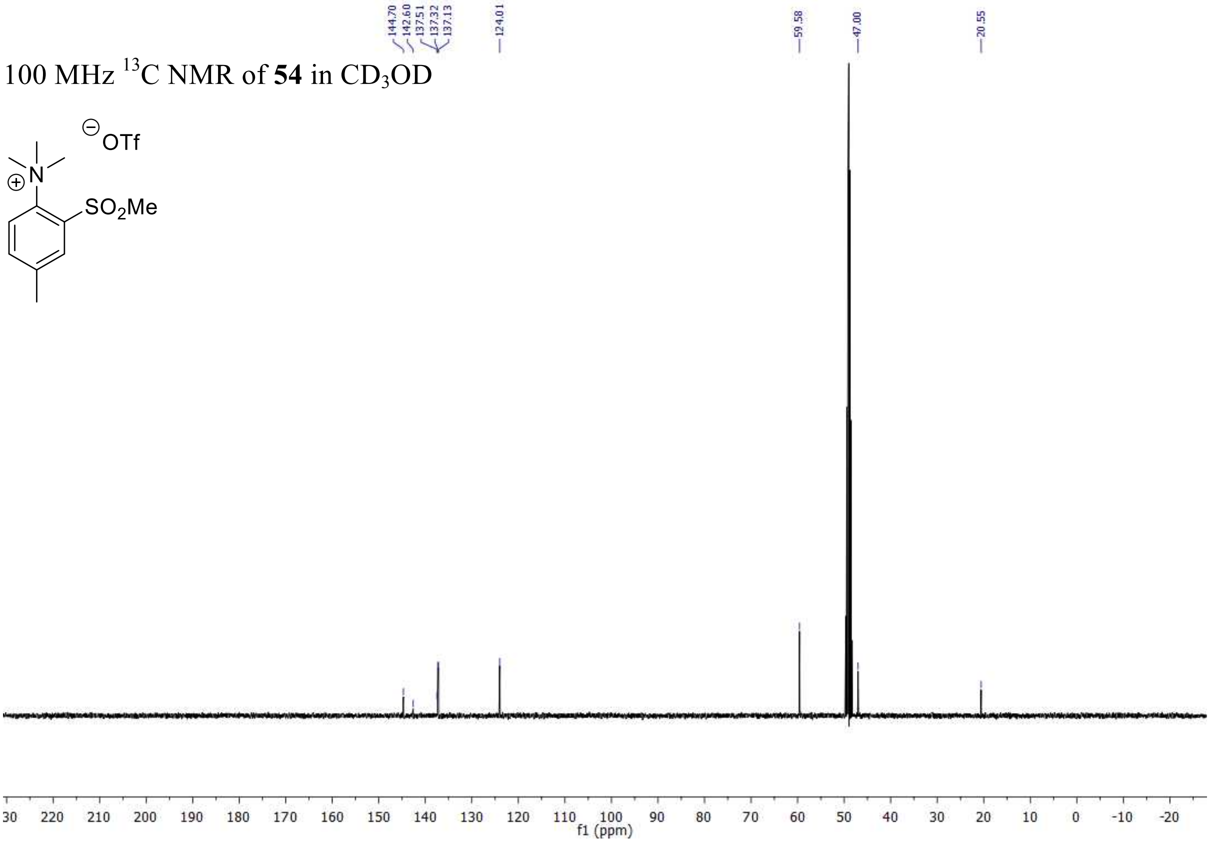

400 MHz ^{1}H NMR of **55** in $CDCl_3$

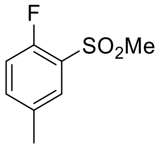

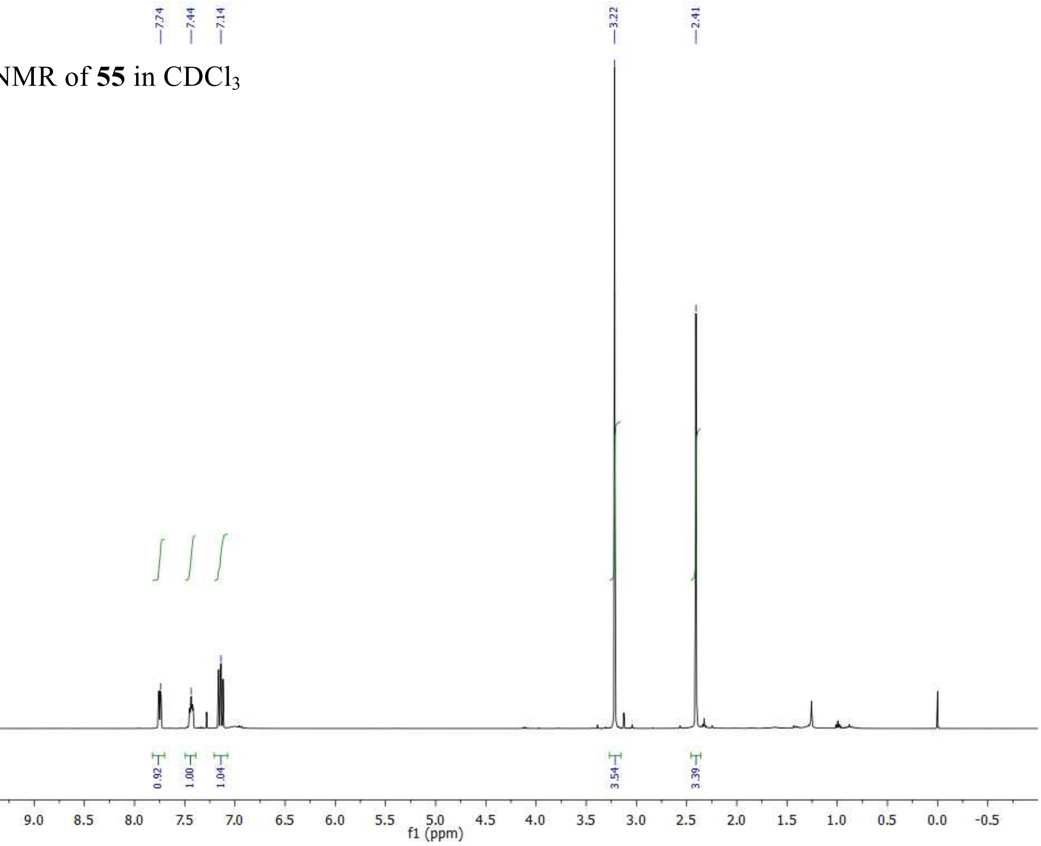

100 MHz ^{13}C NMR of **55** in $CDCl_3$

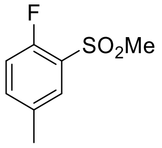

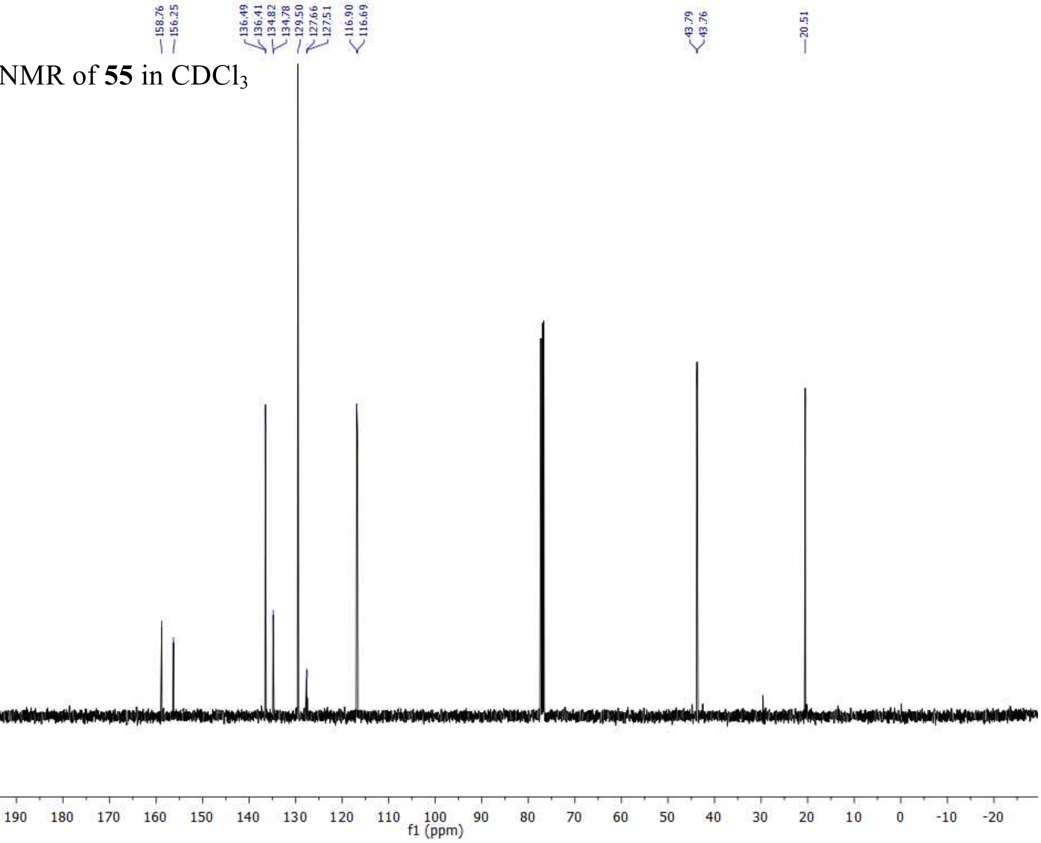

376.5 MHz ^{19}F NMR of **55** in $CDCl_3$

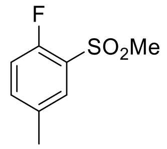

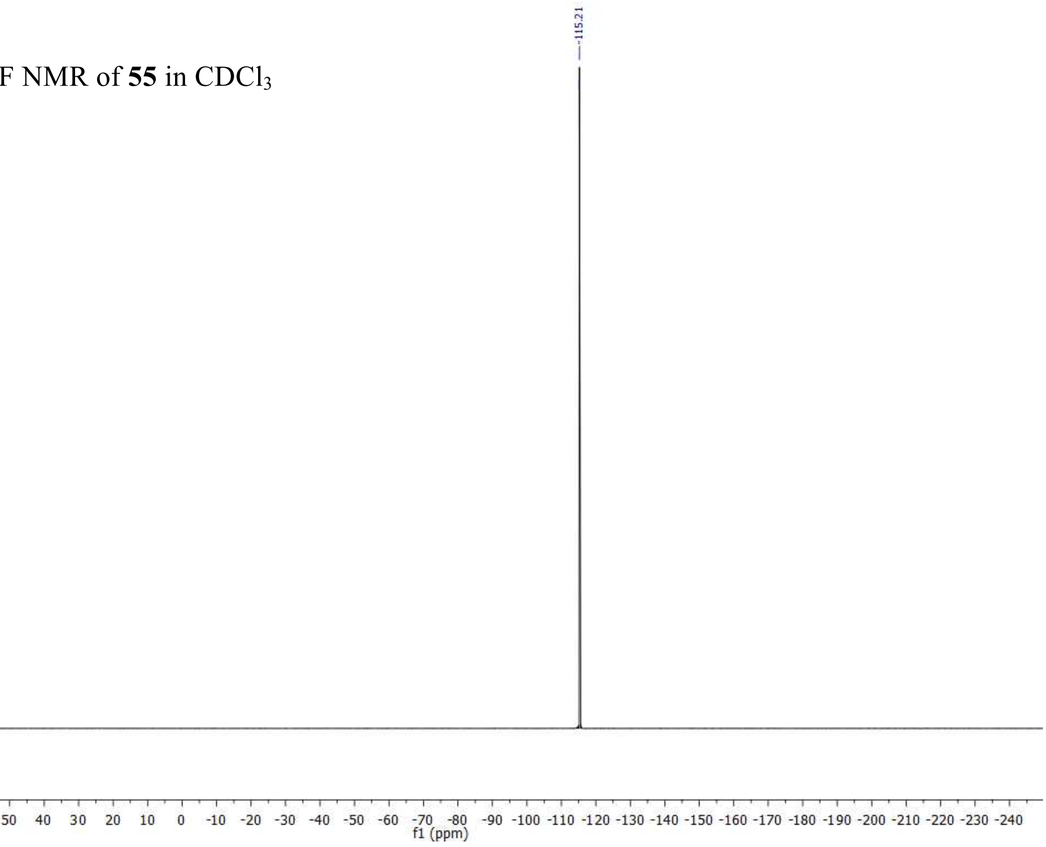

400 MHz ^{1}H NMR of **56** in $CDCl_3$

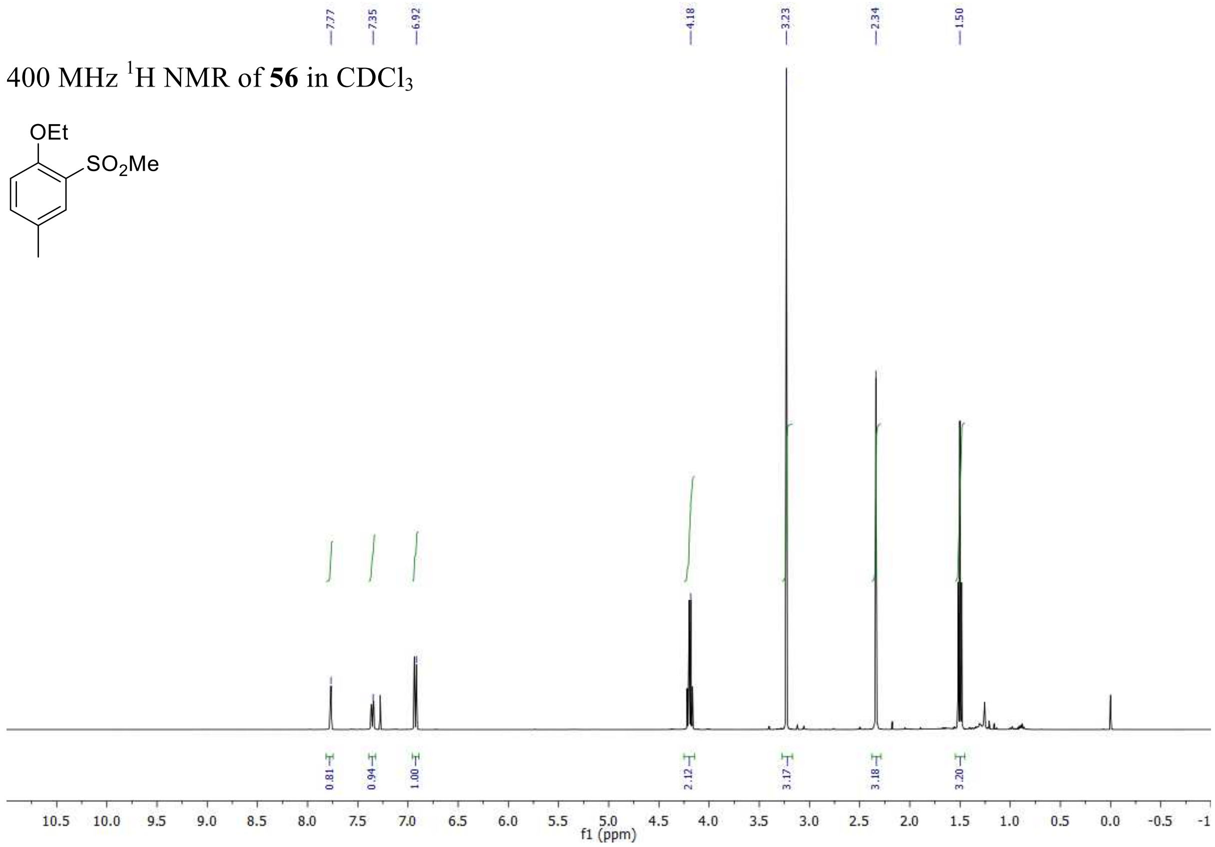

100 MHz ^{13}C NMR of **56** in $CDCl_3$

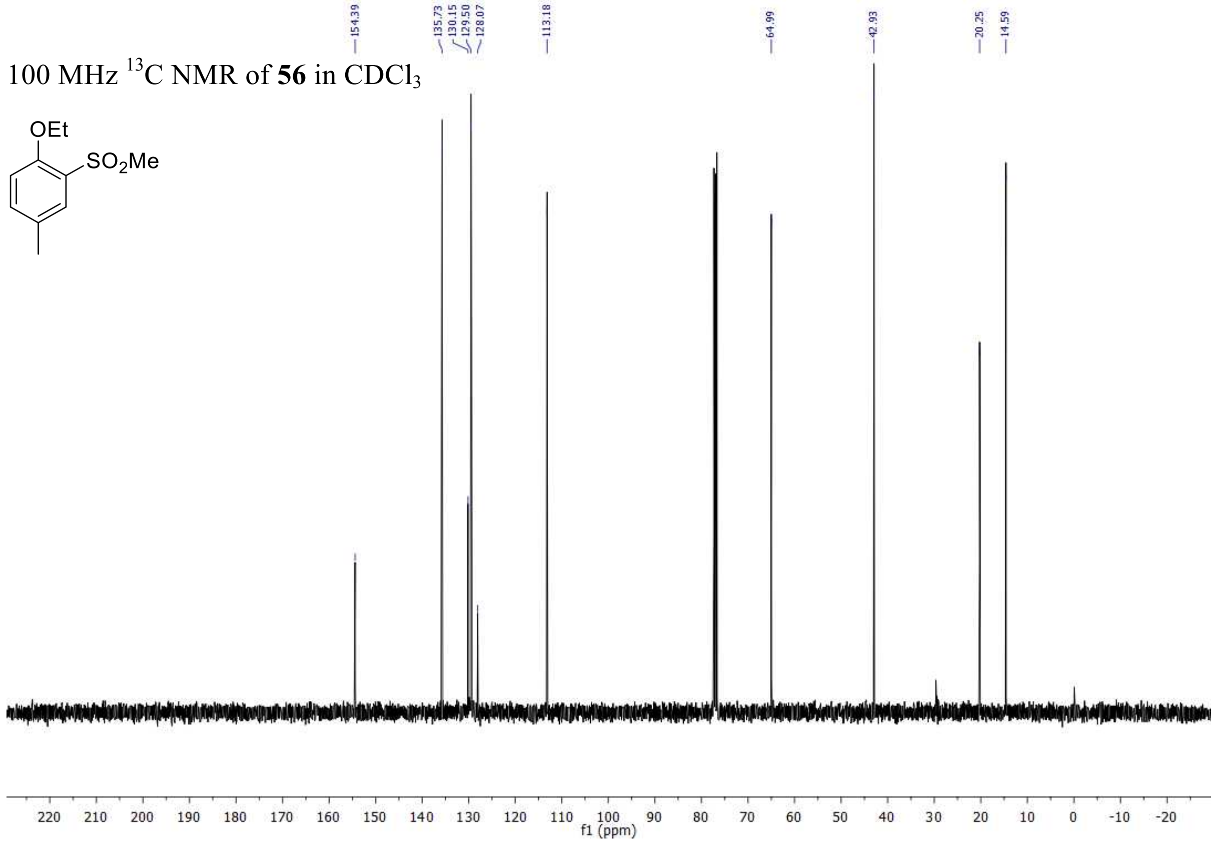

400 MHz ^{1}H NMR of **57** in $CDCl_3$

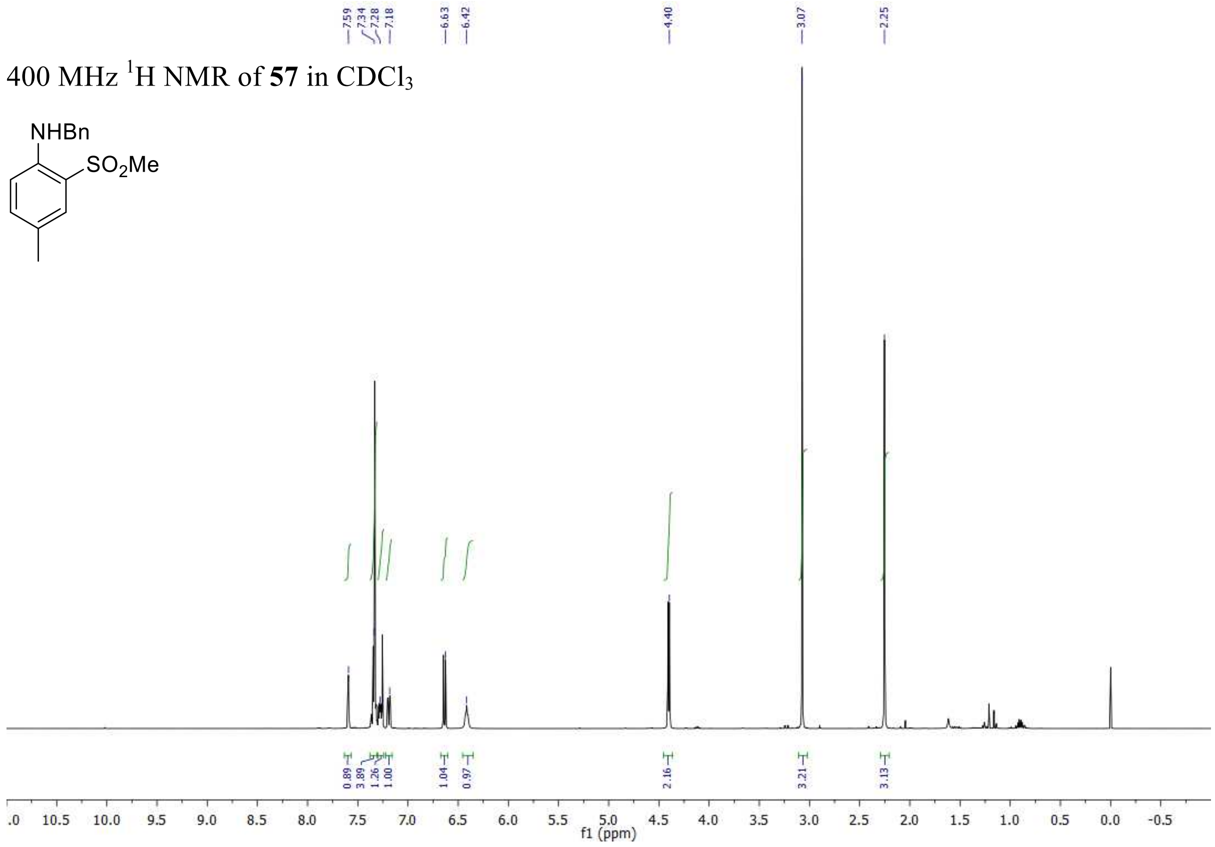

100 MHz ^{13}C NMR of **57** in $CDCl_3$

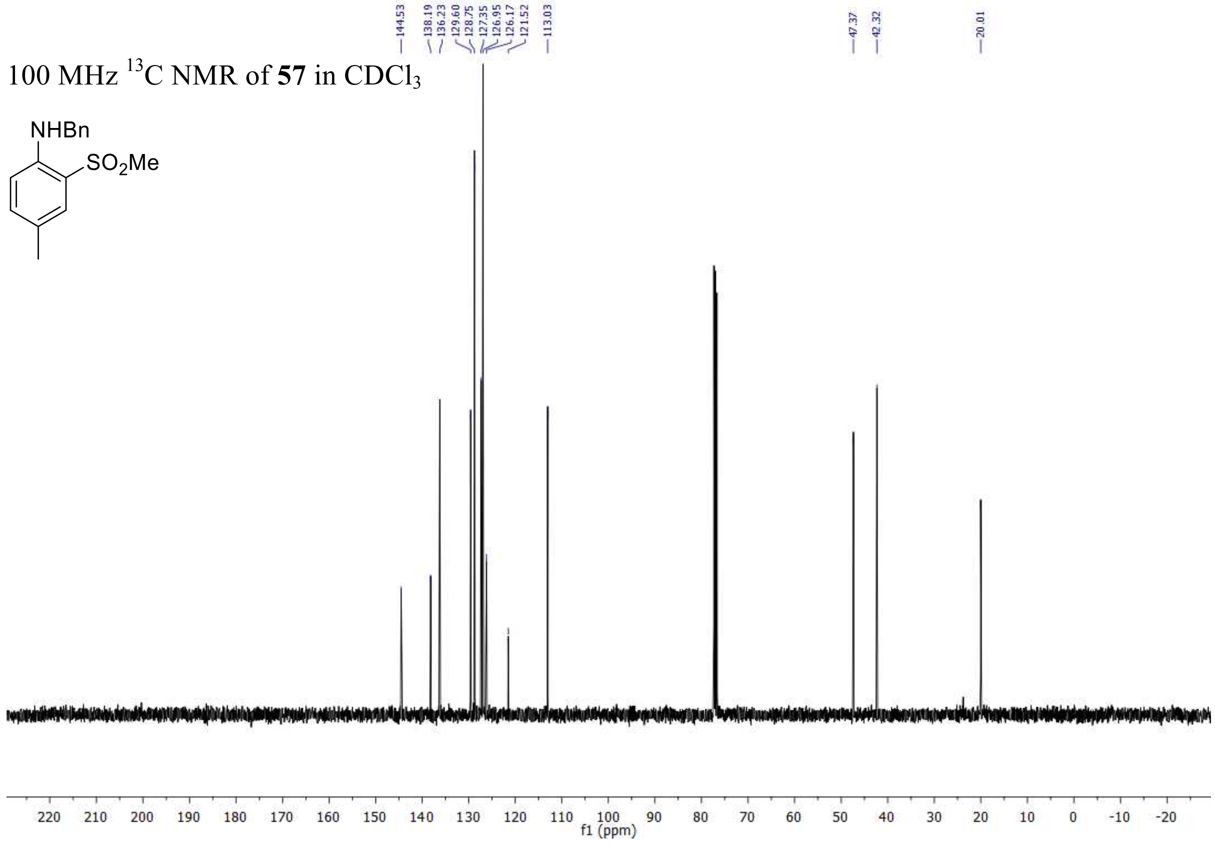

References

1. a) D. O. Jang, K. S. Moon, D. H. Cho, J. G. Kim, *Tetrahedron Lett.* **2006**, *47*, 6063–6066. b) S. Répichet, C. Le Roux, P. Hernandez, J. Dubac, *J. Org. Chem*, **1999**, 6479–6482. c) A. Alizadeh, M. M. Khodaei, E. Nazari, *Tetrahedron Lett.* **2007**, *48*, 6805–6808.

2. R. Gianatassio, S. Kawamura, C.L. Eprile, K. Foo, J. Ge, A.C. Burns, M.R. Collins, P.S. Baran, *Angew. Chem. Int. Ed*, **2014**, *53*, 9851-9855.

3. A.U. Meyer, K. Straková, T. Slanina, B. König, *Chem. Eur. J*, **2016**, *22*, 8694-8699.

4. T. Llamas, R. G. Arrayás, J. C. Carretero, *Org. Lett*, **2006**, *8*, 1795-1798.

5. E.E. Parent, C.S. Dence, C. Jenks, T.L. Sharp, M.J. Welch, J.A. Katzenellenbogen, *J. Med. Chem,* **2007**, *50*, 1028-1040.

6. D.R. Gauthier Jr, N. Yoshikawa, *Org. Lett,* **2016**, *18*, 5994-5997.

7. P. Katrun, S. Hongthong, S. Hlekhlai, M. Pohmakotr, V. Reutrakul, D. Soorukram, T. Jaipetch, C. Kuhakarn, *RSC Adv,* **2014**, *4*, 18933-18938.

8. E.F. Flegeau, J.M. Harrison, M.C. Willis, *Synlett*, **2016**, *27*, 101-105.
